# Potassium *N*-Iodo *p*-Toluenesulfonamide (TsNIK, Iodamine-T): A New Reagent for the Oxidation of Hydrazones to Diazo Compounds

**DOI:** 10.1002/chem.201304656

**Published:** 2014-03-11

**Authors:** Simon M Nicolle, Christopher J Moody

**Affiliations:** [a] School of Chemistry, University of NottinghamNottingham NG7 2RD (UK), Fax: (+44) 115-951-3564

**Keywords:** diazo compounds, hydrazones, iodine, oxidation

## Abstract

A new reagent for the oxidation of hydrazones to diazo compounds is described. *N*-Iodo *p*-toluenesulfonamide (TsNIK, iodamine-T) allows the preparation of α-diazoesters, α-diazoamides, α-diazoketones and α-diazophosphonates in good yield and in high purity after a simple extractive work-up. α-Diazoesters were also obtained in high yield from the corresponding ketones through a one-pot process of hydrazone formation/oxidation.

## Introduction

Diazo compounds are useful and versatile synthetic intermediates and their rich chemistry has attracted great interest since the first synthesis of ethyl diazoacetate by Curtius in 1883.[1] One of their main features is the generation of carbenes or metal carbenoids, reactive intermediates that undergo a wide range of synthetically useful reactions including C=H and X=H insertion reaction (X=N, O, S, P, Si),[2,3] cyclopropanation reactions and ylide formation.[4,5] With conservation of the diazo moiety, they also react as 1,3-dipoles in cycloaddition reactions to give heterocycles.[6]

The numerous routes available for the preparation of diazo compounds reflect not only over a century of research, but also the range of reactivity encountered amongst the members of this class. In fact, few other types of compounds present a stability trend that is so substitution dependent: from toxic and highly reactive diazoalkanes to well-behaved, bench-stable diazocarbonyl compounds, the stability of diazo compounds is strongly related to the substitution on the diazo-bearing carbon atom.[7,8] Stabilised diazo compounds are usually synthesized by the diazo-transfer method, using sulfonyl azides as the transfer reagent.[9] The main drawback of this method is the hazard associated with the use of potentially explosive sulfonyl azides, such as *p*-toluenesulfonyl azide and mesyl azide,[10] although safer alternative reagents such as *p*-dodecylbenzenesulfonyl azide[11] and *p*-acetamidobenzenesulfonyl azide[12] have been used. Possible alternative methods are the thermal decomposition of tosylhydrazones in the presence of a base (Bamford–Stevens reaction),[7,13,14] or the dehydrogenation of simple hydrazones. The latter has been classically carried out using heavy metal based reagents, such as HgO,[15–17] Ag_2_O,[18,19] Pb(OAc)_4_,[20] MnO_2,_[21,22] alumina-supported KMnO_4_,[23] Ni_2_O_3_,[24] CrO_2_,[25] but also Ca(OCl)_2_,[21] Oxone,[26] I_2_/peracid[27] and hypervalent iodine reagents.[28–30] More recently, activated DMSO[31] and NaOCl/TEMPO[32] have also been employed. However, the most widely used methods (HgO, MnO_2_, Pb(OAc)_4_ and Ag_2_O), despite the convenience of some protocols for lab-scale preparations, do not offer satisfactory conditions in terms of waste production and/or toxicity. Moreover, isolation of the diazo compound from the reaction media is often problematic owing to the sensitivity of the less stabilized compounds toward, for instance, purification by column chromatography (as is the case for diphenyldiazomethane). In several examples, the efficiency of the oxidation process is only determined by acid decomposition of the diazo compound and measurement of the quantity of evolved nitrogen[20,27,31] or isolation of the ester produced (decomposition by a carboxylic acid),[18,23–26,32] neither of which are worthwhile if one requires the diazo compound itself for further synthetic transformations. Notwithstanding the aforementioned examples, we set out to develop a new, user-friendly method for the dehydrogenation of hydrazones that offers a broad substrate scope and a simple purification of the diazo compound. We now report our results.

## Results and Discussion

**Scheme 1:**
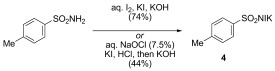
Preparation of *N*-iodo *p*-toluenesulfonamide potassium salt (TsNIK).

With the aforementioned objectives in mind, we began investigating iodine-based oxidants. As noted by Barton et al., the oxidation of simple hydrazones with molecular iodine leads, in the presence of a base, to iodinated species by the intermediacy of the diazo compound, or, as in the case of benzophenone hydrazone (**1**), to the corresponding diazo compound, diphenyldiazomethane (**2**).[33,34] Other methods, based on the combination of sub-stoichiometric iodine source and a stoichiometric oxidant, suggest the involvement of an iodine-based oxidant in these processes.[27,35] Using benzophenone hydrazone (**1**) to investigate oxidation conditions, we found that the use of *N*-iodosuccinimide at −78 °C, together with one equivalent of base, led to diphenyldiazomethane (**2**) in reasonable yield (Table 1, entries 1 to 3) but accompanied by up to 24 % of benzophenone azine **3**. Based on these results, we sought alternative methods involving a mild iodinating species capable of neutralizing the two proton equivalents generated during the oxidation of the hydrazone, as the less stabilized diazo compounds readily decompose in the presence of acid. The rarely investigated *N*-iodo potassium *p*-toluene-sulfonamide salt **4** (TsNIK, also known as iodamine-T), potassium salt and iodinated counterpart of the well-established chloramine-T, was selected on the basis of these criteria. This reagent is conveniently and rapidly prepared from inexpensive *p*-toluenesulfonamide in multi-gram quantity and good yield (74 %) by treatment with iodine in a mixture of aqueous KI and KOH, and simply collected by filtration.[36] Alternatively, because iodine is considered as an “at risk” element,[37] we developed a second protocol using KI and bleach solution to generate iodine, a protocol that gave the reagent in 44 % yield (Scheme 1).

**Table 1:** Oxidation of benzophenone hydrazone using TsNIK and related reagents.

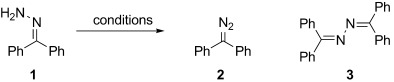				
		Entry	Conditions	Yield [%][a]
		1	2	3
1	NIS (1.0 equiv), TMG (1.0 equiv), THF −78 °C	nd	71	11
2	NIS (1.0 equiv), DBU (1.0 equiv), THF −78 °C	nd	64	24
3	NIS (1.0 equiv), NEt_3_ (1.0 equiv), THF −78 °C	nd	81	7
4	TsNIK (1.1 equiv), THF/H_2_O (23:2), rt	4	84	3
**5**	**TsNIK (1.1 equiv), THF/KOH (4:1), rt**	**4**	**94**	**<1**
6	aqueous “KOI” (3 equiv), H_2_O/Et_2_O, rt	79	20	nd
7	TsNIK (1.1 equiv), DMF, rt	11	88	nd
8	TsNIK (1.05 equiv), THF, 18-crown-6 (0.1 equiv), rt	6	90	2
9	TsNIK (1.1 equiv), MeOH/H_2_O (49:1), rt	53	27	nd
10	TsNClNa (1.1 equiv), THF, 18-crown-6 (0.1 equiv), rt	33	25	42
11	TsNClNa (1.1 equiv), DMF, 0 °C to rt	74	21	4

[a] Yields determined by analysis of the product isolated after an aqueous work-up. nd: not detected. NIS=*N*-iodo succinimide, DBU=1,8-diazabicyclo[5.4.0]undec-7-ene, TMG=1,1,3,3-tetramethylguanidine.

To solubilize the TsNIK reagent, a mixture of THF and water (ratio 23:2) was first chosen to carry out this reaction at room temperature (Table 1, entry 4). Diphenyldiazomethane (**2**) was obtained in good yield under these conditions using a slight excess of oxidant, while benzophenone azine **3** was produced as a side product. The use of a solution of potassium hydroxide (1 m) in a mixture with THF (ratio 4:1) led to a comparable yield but improved the purity of the final product obtained after a simple work-up (Table 1, entry 5). The reduction of TsNIK produces potassium iodide, which is in turn susceptible to oxidation to iodine. In the presence of potassium hydroxide, iodine gives the unstable hypoiodite KOI, which disproportionates at appreciable rates to give potassium iodide and iodate. In another experiment, we found that an excess of freshly prepared hypoiodite aqueous solution led to partial oxidation of a solution of hydrazone **1** in ether (Table 1, entry 6). Unfortunately, the yield was low, likely owing to the rapid decay of potassium hypoiodite and to inefficient mass transfers. In this regard, the potassium salt TsNIK might act as a stable hypoiodite equivalent.

The reaction can also be successfully carried out in dry dimethylformamide, giving 88 % conversion of the hydrazone **1** into diazo compound **2** (Table 1, entry 7). Reaction in dry THF was possible by addition of 18-crown-6 (0.1 equiv) to increase solubility of TsNIK (Table 1, entry 8). Reactions carried out in methanol/water led to poor conversions (Table 1, entry 9). Using the THF/KOH (1 m) solvent system, the sulfonamide resulting from the reduction of TsNIK was easily and quantitatively removed from the reaction media by washing with a solution of potassium hydroxide (1 m) and extraction in diethyl ether. Moreover, the product extracted in the organic phase was found to be of satisfactory purity (96 % by ^1^H NMR analysis), and further purification was not carried out. *p-*Toluenesulfonamide, the precursor to TsNIK, was recovered from the aqueous phase by an acidification/extraction sequence, and in principle can be recycled by treatment with I_2_/KI/KOH or KI/bleach. In contrast, the use of TsNClNa (chloramine-T) for hydrazine oxidation proved unsatisfactory under similar conditions (Table 1, entries 10 and 11).

Based on these encouraging results and in order to establish the scope of this oxidation, we set out to prepare a number of hydrazones from diverse ketone precursors. Various α-ketoester and α-ketoamide hydrazones were obtained by condensation of either commercially or readily available α-keto-esters or -amides with hydrazine hydrate in presence of a weak Brønsted acid, as previously reported (Table 2).[38,39] The reaction was carried out in methanol/water using acetic acid (conditions a, Table 2) or in THF using benzoic acid (conditions b, Table 2), and under both sets of conditions, hydrazones were obtained in high yields as separable (*E*)- and (*Z*)-isomers. However the latter set of conditions (b) offer several advantages allowing the reaction to take place within a few hours, at room temperature, using only one equivalent of acid and hydrazine hydrate, whereas the original conditions (a) required acetic acid as a co-solvent and an excess of hydrazine (2 equiv). Excellent conversions into the corresponding hydrazones were obtained for a range of α-ketoesters (Table 2, entries 1–10) and α-ketoamides (Table 2, entries 10, 11 and 16–18). Lower yields were obtained for hydrazones **6 m**–**o** (Table 2, entries 13–15) derived from heteroaromatic ketones that gave significant amounts of carbazide by displacement of the ester alkoxy group. The configuration of the C=N bond was determined on the basis of the N*H*_2_ signals in the^1^H NMR spectrum, and the IR carbonyl resonance frequencies, based on the existence of an intramolecular hydrogen bond in the (*Z*)-isomer.

**Table 2:** Hydrazone formation.[a]

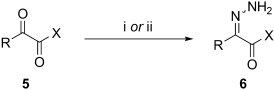						
Entry		Ketone		Hydrazone	Yield [%]	*E*/*Z* ratio
		R	X		(conditions)	
1	**5 a**	Ph	OEt	**6 a**	99 (a) 97 (b)[b]	23:77 26:74
2	**5 b**	4-MeO-C_6_H_4_	OEt	**6 b**	77 (a) 92 (b)[b]	22:78 23:77
3	**5 c**	2-MeO-C_6_H_4_	OEt	**6 c**	94 (a)	35:65
4	**5 d**	4-Br-C_6_H_4_	OEt	**6 d**	96 (b)	37:63
5	**5 e**	*i*Pr	OEt	**6 e**	92 (a) 92 (b)[b]	34:62 37:63
6	**5 f**	Me	OEt	**6 f**	91 (a)	100:0
7	**5 g**	PhCH_2_CH_2_	OEt	**6 g**	99 (a) 98 (b)[b]	87:13 94:6
8	**5 h**	Ph	OCH_2_CH=CH_2_	**6 h**	91 (a) 90 (b)[b]	24:76 28:72
9	**5 i**	Ph	O*c*-Hex	**6 i**	98 (a) 99 (b)[b]	18:82 30:70
10	**5 j**	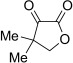	**6 j**	96 (b)[c]	88:12	
11	**5 k**	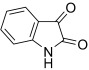	**6 k**	65 (b)[b]	89:11	
12	**5 l**	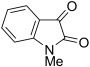	**6 l**	98 (b)[b]	85:15	
13	**5 m**	2-thienyl	OEt	**6 m**	36 (b)	41:59
14	**5 n**	2-pyridyl	OEt	**6 n**	68 (b)[b]	9:91
15	**5 o**	1-Boc-indol-3-yl	OMe	**6 o**	54 (b)	48:52
16	**5 p**	Ph	NHCH_2_CH=CH_2_	**6 p**	99 (b)	16:84
17	**5 q**	Ph	NH(2,5-(MeO)_2_C_6_H_3_	**6 q**	98 (b)	10:90
18	**5 r**	Ph	NHCH_2_(2-furyl)	**6 r**	98 (b)	12:88

[a] Reagents and conditions: (i) N_2_H_4_⋅H_2_O (2 equiv), acetic acid/methanol/water, rt, 1–17 h; (ii) N_2_H_4_⋅H_2_O (1 equiv), benzoic acid (1 equiv), THF, rt, 14–39 h.

[b] Hydrazone isomers were not separated.

[c] Hydrazine acetate was used instead of hydrazine hydrate and benzoic acid.

**Scheme 2:**
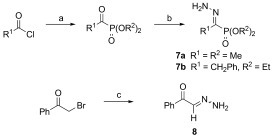
Formation of α-keto-phosphonate hydrazones 7 and α-keto-aldehyde hydrazones 8. Reagents and conditions: a) P(OR^2^)_3_, neat; b) N_2_H_4_⋅H_2_O (2 equiv for 7 a, 1 equiv for 7 b), acetic acid/R^2^OH, 7 a 59 %, 7 b 87 %; c) N_2_H_4_⋅H_2_O (3 equiv), MeOH reflux, 53 %.

α-Keto phosphonate hydrazones **7 a**–**b** were obtained by an adaptation of a known procedure,[40,41]from condensation of equimolar quantities of trialkyl phosphites with acyl chloride (Michaelis–Arbuzov reaction) followed by one-pot condensation with hydrazine hydrate in the presence of acetic acid (Scheme 2). Similarly to α-ketoesters and α-ketoamides, α-ketophosphonate hydrazone **7 b** exists as its separable (*E*)- and (*Z*)-isomers whilst hydrazone **7 a** was obtained as pure (*E*)-isomer. Hydrazone **8** was obtained by the method previously described by Hauptmann and co-workers starting from the corresponding α-bromo ketone (Scheme 2).[42]Benzil monohydrazone (**9**) was obtained by a literature procedure.[43]

The stability of the hydrazones **6**–**8** was variable. Although all of them could be kept at −5 °C under argon for several months, storage in air at room temperature was followed by partial decomposition for products **6 b**–**g**. On the contrary, hydrazone **6 a** was found to be stable for several months under the same storage conditions. Partial isomerizations during storage in isolated form and in solution were frequently observed (mostly in favour of the (*E*)-hydrazone).

The TsNIK oxidation conditions previously described were then applied to various hydrazones, including those prepared as described above (Table 3, conditions a). Unless specified, the product was obtained free of sulfonamide and in >95 % purity (determined by ^1^HNMR spectroscopic analysis of the final product) following a single extractive work-up. Benzil monohydrazone (**9**) gave a quantitative conversion into azibenzil (**10**; Table 3, entry 1). Both (*E*)- and (*Z*)-hydrazones of ethyl phenylglyoxylate **6 a** were oxidized with success and gave similar yields of diazoester **11 a** (Table 3, entry 2). 4-Methoxy-, 2-methoxyphenyl and 4-bromo- derivatives (**6 b**–**d**) were readily converted into the corresponding diazoesters, **11 b**–**d** (Table 3, entries 3–5). α-Ketoester hydrazones substituted by various alkyl groups, **6 e**–**g**, were also oxidized in excellent yields (Table 3, entries 6–8). Variation in the ester moiety of the α-ketoester hydrazone was also tolerated (allyl- in **6 i**, cyclohexyl- in **6 h**; Table 3, entries 9 and 10). Similarly, diazophosphonates **12 a**–**b** were obtained from α-keto phosphonate hydrazones **7 a**–**b** in good yield by using the same procedure (Table 3, entries 20 and 21). The yield for methyl diazophosphonate **12 a** was somewhat lowered owing to its enhanced water solubility, thus complicating the extractive work-up. The ketoaldehyde monohydrazone **8** was also oxidized to the corresponding diazoketone **13** in excellent yield (Table 3, entry 22). As noted by Hauptmann and co-workers,[42] this route for the preparation of diazoketones such as **13** represents an interesting alternative to the classical synthesis of this type of compound which involves the condensation of an acyl chloride with an excess of diazomethane.

**Table 3:** Oxidation of hydrazones to diazo compounds and a one-pot sequence from ketones to diazo compounds.[a]

	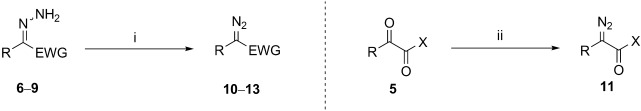											
Entry	Ketone/			Product	Yield [%]		Entry	Ketone/		Product		Yield [%]
	Hydrazone			i[b]	ii[c]			Hydrazone			i[b]	ii[c]
1	**9**	**10**	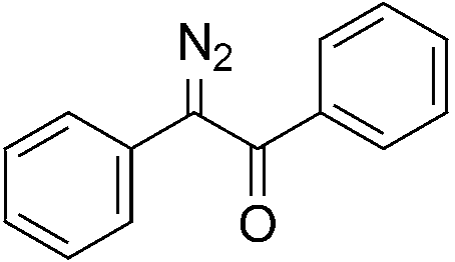	100	–		12	**5 k**	**11 k**	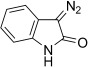	–	94[g]
2	**5 a 6 a**	**11 a**	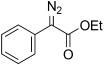	93[d] 95[e]	97		13	**5 l**	**11 l**	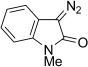	–	89
3	**5 b 6 b**	**11 b**	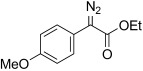	93[d]	87		14	**5 m 6 m**	**11 m**	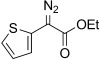	95^[f, g]^	–[h]
4	**6 c**	**11 c**	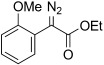	89[d]	–		15	**5 n**	**11 n**	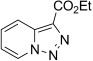	–	85[g]
5	**5 d**	**11 d**	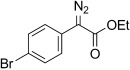	–	95		16	**5 o 6 o**	**11 o**	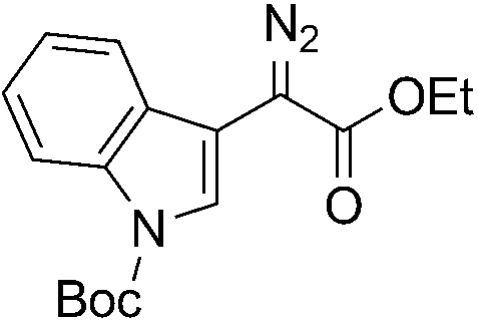	79[d]	–[h]
6	**5 e 6 e**	**11 e**	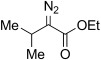	87[d]	87		17	**5 p**	**11 p**	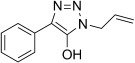	–	85[g]
7	**5 f 6 f**	**11 f**	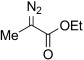	67[e]	96		18	**5 q**	**11 q**	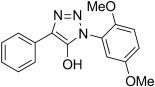	–	91
8	**5 g 6 g**	**11 g**	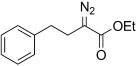	91[e]	84		19	**5 r**	**11 r**	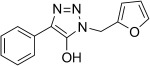	–	75[g]
9	**5 h 6 h**	**11 h**	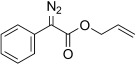	95[d]	88		20	**7 a**	**12 a**	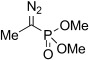	56[e]	–
10	**5 i 6 i**	**11 i**	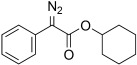	93[d]	99		21	**7 b**	**12 b**	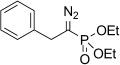	94[e]	–
11	**5 j**	**11 j**	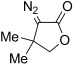	–	68		22	**8**	**13**	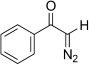	94[e]	–

[a] Reagents and conditions: (i) TsNIK (1.1 equiv), THF/aq. KOH (1 m). EWG: CO_2_R^1^, PO(OR^2^)_2_; (ii) N_2_H_4_⋅H_2_O (1.0 equiv), benzoic acid (1.0 equiv), THF, then aq. KOH (1 m), TsNIK (1.2–1.6 equiv).

[b] Yield based on starting hydrazone.

[c] Yield based on starting ketone.

[d] From (*Z*)-hydrazone.

[e] From (*E*)-hydrazone.

[f] From mixture of (*E*)- and (*Z*)-hydrazone.

[g] Purification by chromatography was necessary.

[h] A complex mixture was obtained containing the diazo compound in small quantity.

Because both hydrazone formation and oxidation steps could be carried out in the same solvent (THF), we envisioned the possibility of combining those two steps into a one-pot process. Thus, the hydrazone was first produced by using one equivalent of hydrazine hydrate; later addition of KOH (1 m) and TsNIK to the mixture gave, after extraction, the corresponding diazoester in pure form. α-Ketoesters gave diazo compounds in excellent yield by following this procedure (Table 3, conditions b), with yields comparable to those previously obtained through the two-step process. The enhanced yield obtained for diazoacetate **11 f** in the one-pot process, in comparison with the direct oxidation of hydrazone **6 f**, can be explained by the relative instability of the hydrazone precursor that was found to undergo decomposition upon storage. The failure of the one-pot process for ketones **5 m** and **5 o** (Table 3, entries 14 and 16) is likely to be due to the low efficiency of the hydrazone formation step, as noted previously for these compounds (Table 2, entries 13 and 15). Diazo compounds **11 m** and **11 o** were obtained in good yield starting from isolated hydrazones **6 m** and **6 o**.

The pyridinyl diazoacetate derived from ketone **5 n** was not observed as it undergoes a spontaneous 1,5-electrocyclization to give the previously reported triazolopyridine**11 n**.[44] A similar case of cyclization was observed in the attempted preparation of diazoamides from α-ketoamides **5 p**–**5 r** that resulted in isolation of 5-hydroxy-1,2,3-triazoles **11 p**–**11 r** in high yield. Such cyclizations of α-diazoamides into 1,2,3-triazol-5-ols were observed as early as 1904 by Dimroth and take place in the eponymous rearrangement of 5-amino-1,2,3-triazoles, during which diazo compounds are believed to be present as intermediates.[45–47]

## Conclusion

We have developed a new method for the dehydrogenation of hydrazones to diazo compounds. This method permits the preparation of stabilized diazo compounds such as diazoesters, diazoamides, diazoketones, diazophosphonates and diphenyldiazomethane in high yields. A range of diazoesters was obtained in a one-pot procedure starting from the corresponding ketones. In most cases, diazo compounds were obtained in high purity following a simple extractive work-up without the need for further purifications.

## Experimental Section

### General method for the synthesis of α-ketoester and α-ketoamide hydrazones 6

**Method A**:[39]Hydrazine hydrate (2 equiv) was slowly added to a mixture of glacial acetic acid (80 mL mol^−1^) and water (80 mL mol^−1^) cooled in an ice bath. The α-keto-ester or -amide (1 equiv) was added to the mixture at room temperature and methanol was added in order to obtain a homogeneous solution when necessary. The reaction mixture was stirred at room temperature until completion of the reaction as judged by TLC. The volatiles were removed under reduced pressure. Water (1.6 L mol^−1^) was added to the residue and the mixture was extracted with ethyl acetate (3.0 L mol^−1^). The combined organic phases were washed with saturated sodium hydrogen carbonate (1.0 L mol^−1^), saturated brine (0.5 L mol^−1^) and dried over MgSO_4_. The solvent was removed under reduced pressure to give a residue that was purified by column chromatography if necessary.

**Method B**: Hydrazine hydrate (1 equiv) was added to a solution of benzoic acid (1 equiv) and α-keto-ester or -amide (1 equiv) in THF (3 L mol^−1^), upon which the hydrazine benzoate precipitates in the solution. The mixture was stirred at room temperature during which the visible precipitate disappeared completely. The solution was poured on saturated sodium hydrogen carbonate solution (2.0 L mol^−1^) and extracted with ethyl acetate (6.0 L mol^−1^). The combined organic phases were washed with saturated brine (0.5 L mol^−1^) and dried over MgSO_4_. Evaporation of the solvent under reduced pressure gave a residue that was purified by column chromatography if necessary.

### Potassium N-iodo *p*-toluenesulfonamide 4 (TsNIK)

Prepared by a modification of a procedure previously described.[36] A solution of *p*-toluenesulfonamide (4.55 g, 26.6 mmol) in aqueous potassium hydroxide (10 %; 11.5 mL) was added to a solution of potassium iodide (18.0 g, 108 mmol) and iodine (9.00 g, 35.5 mmol) in water (20 mL). Aqueous potassium hydroxide (50 %; 6 mL) was added, upon which loss of the colouration owing to iodine occurred and a yellow precipitate appeared. The yellow solid was filtered, dried under suction and washed with ether (20 mL) to give the title compound as a yellow solid (6.57 g, 74 %). M.p.: 220 °C (decomposition; no m.p was reported in previously published preparation)[36]. ^1^H NMR (400 MHz; [D_6_]DMSO): *δ*=7.50 (2 H, d, *J*=8.0, ArH), 7.15 (2 H, d, *J*=8.0, ArH), 2.31 ppm (3 H, s, CH_3_); ^13^C NMR (100 MHz; [D_6_]DMSO): *δ*=144.0 (C), 138.1 (C), 128.0 (CH), 126.6 (CH), 20.8 ppm (CH_3_); IR(ATR): 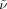
=1191, 1065, 959, 664, 625 cm^−1^; elemental analysis calcd (%) for C_7_H_7_INO_2_S: C 25.08, H 2.10, N 4.18; found: C 24.89, H 2.01, N 3.95. The product showed no signs of decomposition when stored in the dark at room temperature over several weeks but decomposes with iodine release when heated above 220 °C.

### TsNIK oxidation of hydrazone to the corresponding diazo compound: procedure A

A suspension of potassium *N*-iodo *p*-toluenesulfonamide (369 mg, 1.1 mmol) in a solution of hydrazone in THF (1 mmol in 4 mL) was prepared. For the hydrazones that were solid at room temperature, THF was added to a mixture of the hydrazone and potassium *N*-iodo *p*-toluenesulfonamide. Aqueous potassium hydroxide (1 m) was slowly added to the THF suspension (so that the final volume ratio KOH (1 m)/THF was 1:4). This caused dissolution of the potassium salt in the mixture and the appearance of a yellow to red colouration. In all cases the reaction was complete after stirring for 1 h at room temperature. The mixture was poured into aqueous potassium hydroxide (1 m; 5 mL) and extracted with ether (30 mL). The ethereal phase was washed with aqueous potassium hydroxide (1 m; 5 mL), saturated brine (5 mL) and dried over MgSO_4_. Removal of the solvent under reduced pressure gave diazo compounds which were >95 % pure as judged by ^1^H NMR spectroscopy.

### One-pot process hydrazone formation and oxidation, from α-ketoester to the corresponding diazo compound: procedure B

The starting α-ketoester (1.0 mmol) and benzoic acid (123 mg, 1.0 mmol) were dissolved in THF (4 mL) and hydrazine hydrate (49 μL, 1.0 mmol) was added. The mixture was stirred at room temperature for 16 h or until disappearance of the colourless hydrazine salt precipitate and completion of the reaction as judged by TLC. Aqueous potassium hydroxide (1 m; 1 mL) was then added at room temperature, followed by slow addition of potassium *N*-iodo *p*-toluenesulfonamide (402 mg, 1.2 mmol). The reaction progress was monitored by TLC. When required, additional quantities of TsNIK were added to the mixture (in 0.1 mmol portion). After completion, the reaction mixture was poured into aqueous potassium hydroxide (1 m; 5 mL) and extracted with ether (30 mL). The ethereal phase was washed with aqueous potassium hydroxide (1 m; 5 mL), saturated brine (5 mL) and dried over MgSO_4_. Removal of the solvent under reduced pressure gave diazo compounds that were over 95 % pure as judged by ^1^H NMR spectroscopy.

Further experimental detail, and characterization data for all compounds are given in the Supporting Information.

## References

[1] Curtius T (1883). Ber. Dtsch. Chem. Ges.

[2] Doyle MP, McKervey MA, Ye T (1998). Modern Catalytic Methods for Organic Synthesis with Diazo Compounds.

[3] Murphy GK, Stewart C, West FG (2013). Tetrahedron.

[4] Padwa A, Weingarten MD (1996). Chem. Rev.

[5] Gillingham D, Fei N (2013). Chem. Soc. Rev.

[6] Padwa A (1984). 1,3-Dipolar Cycloaddition Chemistry.

[7] Regitz M, Maas G (1986). Diazo Compounds, Properties and Synthesis.

[8] Maas G Angew. Chem.

[9] Regitz M (1967). Angew. Chem. Int. Ed. Engl.

[10] Bollinger FW, Tuma LD (1996). Synlett.

[11] Hazen GG, Weinstock LM, Connell R, Bollinger FW (1981). Synth. Commun.

[12] Baum JS, Shook DA, Davies HML, Smith HD (1987). Synth. Commun.

[13] Bamford WR, Stevens TS (1952). J. Chem. Soc.

[14] Bartrum HE, Blakemore DC, Moody CJ, Hayes CJ (2011). Chem. Eur. J.

[15] Curtius T (1889). Ber. Dtsch. Chem. Ges.

[16] Schönberg A, Awad WI, Latif N, Mustafa A, Goodman I, McIlroy RJ, Badcock WE, Pausacker KH, Ross IG, Hall DM, Mitchell RK, Albert A, Baker W, Coates GE, Glockling F, Atkinson RO, Poppelsdorf F (1951). J. Chem. Soc.

[17] Andrews SD, Day AC, Raymond P, Whiting MC (1970). Org. Synth.

[18] Schroeder W, Katz L (1954). J. Org. Chem.

[19] Heyns K, Heins A (1957). Liebigs Ann.

[20] Holton TL, Schechter H (1995). J. Org. Chem.

[21] Morrison H, Danishefsky S, Yates P (1961). J. Org. Chem.

[22] Kaufmann KD, Ruehlmann K (2010). Z. Chem.

[23] Lee K-H, Ko K-H (2006). Bull. Korean Chem. Soc.

[24] Nakagawa K, Onoue H, Minami K (1966). Chem. Commun.

[25] Ko K-Y, Kim J-Y (1999). Bull. Korean Chem. Soc.

[26] Curini M, Rosati O, Pisani E, Cabri W, Brusco S, Riscazzi M (1997). Tetrahedron Lett.

[27] Adamson JR, Bywood R, Eastlick DT, Gallagher G, Walker D, Wilson EM (1975). J. Chem. Soc. Perkin Trans. 1.

[28] Nicolaou KC, Mathison CJN, Montagnon T (2004). J. Am. Chem. Soc.

[29] Smith PAS, Bruckmann EM (1974). J. Org. Chem.

[30] Furrow ME, Myers AG (2004). J. Am. Chem. Soc.

[31] Javed MI, Brewer M (2007). Org. Lett.

[32] Perusquía-Hernández C, Lara-Issasi GR, Frontana-Uribe BA, Cuevas-Yañez E (2013). Tetrahedron Lett.

[33] Quiclet-Sire B, Zard SZ (2006). Chem. Commun.

[34] Barton DHR, O’Brien RE, Sternhell S (1962). J. Chem. Soc.

[35] Kawahara I, Sasaoka M, Wada I

[36] Roberts E (1923). J. Chem. Soc.

[38] Barton DHR, Jaszberenyi JC, Liu W, Shinada T (1996). Tetrahedron.

[39] Ciganek E (1970). J. Org. Chem.

[40] Yuan CY, Chen SJ, Xie RY, Feng HZ, Maier L (1995). Phosphorus Sulfur Silicon Relat. Elem.

[41] Ben Akacha A, Barkallah S, Baccar B (1992). Phosphorus Sulfur Silicon Relat. Elem.

[42] Hauptmann S, Kluge M, Seidig KD, Wilde H (1965). Angew. Chem.

[43] Alonazy HS, Al-Hazimi HMA, Korraa MMS (2009). Arab. J. Chem.

[44] Davies HML, Townsend RJ (2001). J. Org. Chem.

[45] Dimroth O (1910). Liebigs Ann.

[46] Dimroth O, Stahl H (1904). Liebigs Ann.

[47] Dimroth O (1909). Liebigs Ann.

