# Supplementary material for: Potassium N-Iodo p-Toluenesulfonamide (TsNIK, Iodamine-T): A New Reagent for the Oxidation of Hydrazones to Diazo Compounds
Source: Chemistry. 2014 Mar 11;20(15):4420–5. doi: 10.1002/chem.201304656 (PMC4164276; doi:10.1002/chem.201304656)

CHEMISTRY
A European Journal

Supporting Information

© Copyright Wiley-VCH Verlag GmbH & Co. KGaA, 69451 Weinheim, 2014

Potassium *N*-Iodo *p*-Toluenesulfonamide (TsNIK, Iodamine-T): A New Reagent for the Oxidation of Hydrazones to Diazo Compounds

Simon M. Nicolle and Christopher J. Moody*[a]

chem_201304656_sm_miscellaneous_information.pdf

GENERAL INFORMATION

Commercially available reagents were used throughout, without purification unless otherwise stated. Bleach solution was obtained from Alfa Aesar (14.5% available chlorine, 8.4% by iodometric titration). Tetrahydrofuran and dichloromethane were freshly distilled according to standard procedures; tetrahydrofuran was distilled from sodium benzophenone ketyl radical and dichloromethane from calcium hydride. When indicated, reactions were carried out in flame-dried vessels under an argon atmosphere using anhydrous solvents. Light petroleum refers to the fraction with bp 40-60 °C. Ether refers to diethyl ether. All aqueous solutions were prepared using deionized water. Saturated brine refers to aqueous saturated solution of sodium chloride.

Analytical thin layer chromatography was carried out on aluminium backed plates coated with Merck Kieselgel 60 GF_{254} and visualized under UV light at 254 and/or 360 nm. Chemical staining was also routinely used with either ethanolic vanillin or aqueous basic potassium permanganate. Flash chromatography was carried out using Davisil silica 60Å at medium pressure, with the eluent specified.

Infrared spectra were recorded in solution using a PerkinElmer 1600 series FT-IR spectrometer, using NaCl cells over the range 4000 - 600 cm^{-1}. For solid samples, infrared spectra were recorded using Nicolet Avatar 320 FTR-IR spectrometer equipped with an OMNI-Sampler™ Smart Accessory for HATR (Germanium crystal, DTGS detector) over the range 4000 - 600 cm^{-1}. NMR spectra were recorded at 298 K using a Bruker AV(III)500 instrument (500 MHz ^{1}H frequency, 125 MHz ^{13}C frequency), AV(III)400, AV400, DPX400 (400 MHz ^{1}H frequency, 100 MHz ^{13}C frequency, ^{31}P frequency 162 MHz) or DPX300 instrument (300 MHz ^{1}H frequency, 75 MHz ^{13}C frequency). Chemical shifts are quoted in parts per million (ppm), referenced to residual chloroform (7.26 ppm for ^{1}H NMR, 77.16 ppm

for ^{13}C NMR), dimethylsulfoxide (2.50 ppm for ^{1}H NMR, 39.51 ppm for ^{13}C NMR), acetone (2.05 ppm for ^{1}H NMR, 29.84 ppm for ^{13}C NMR) and methanol (3.31 ppm for ^{1}H NMR, 49.00 ppm for ^{13}C NMR) as internal standards and coupling constants, *J*, are quoted in Hz. Multiplicity of each signal is designated by the following abbreviations: s, singlet; d, doublet; t, triplet; q, quartet; m, multiplet; app, apparent; br, broad or combinations thereof. In the ^{13}CNMR spectra, signals corresponding to C, CH, CH_2 and CH_3 were assigned from DEPT experiments. Triphenylphosphine was used as a secondary standard for the calibration in ^{31}P NMR experiments (primary standard: phosphoric acid). Mass spectra were recorded on a Bruker MicroTOF 61 mass spectrometer using electrospray ionization (ESI). Melting points were measured on a Riechert-Kofler hot stage apparatus and are uncorrected. Elemental analyses were carried out using an Exeter Analytical CE-440 Elemental Analyser on dry homogeneous sample of material.

STARTING CARBONYL COMPOUNDS 5

ethyl phenyl-2-oxo-acetate **5a**, ethyl 3-methyl-2-oxo-butanoate**5e**, ethyl pyruvate**5f**, ethyl 2-oxo-4-phenylbutanaote**5g**, 4,4-dimethyldihydro-2,3-furandione **5j**, isatin**5k**, and *N*-methylisatin **5l**.

α-Ketoester synthesis

Ethyl 2-(4-methoxyphenyl)-2-oxoacetate**5b**, ethyl 2-(2-methoxyphenyl)-2-oxoacetate **5c**, ethyl 2-(4-bromophenyl)-2-oxoacetate**5d**and ethyl 2-oxo-2-(thiophen-2-yl)acetate**5m**were prepared by Friedel-Craft acylation of the corresponding aromatic compound with ethyl oxalyl chloride, as previously described.[1]*tert*-Butyl 3-(2-methoxy-2-oxoacetyl)indole-1-carboxylate**5o** was prepared by acylation of indole,[2] followed by Boc-protection.[3]Allyl 2-oxo-2-phenylacetate **5h** and cyclohexyl 2-oxo-2-phenylacetate**5i**were prepared according to a previously described procedures.[4]

Allyl 2-oxo-2-phenylacetate 5h

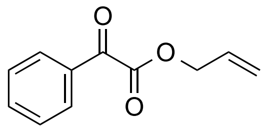

yellow liquid; Rf 0.4 (ethyl acetate : light petroleum, 1 : 24); (Found: M+Na$^+$, 213.0541. $C_{11}H_{10}NaO_3$ requires 231.0522); ν_{max} ($CHCl_3$)/cm^{-1} 3045, 1737, 1690, 1598, 1451, 1322, 1192, 1177, 1004, 988; δ_H (400 MHz; $CDCl_3$) 8.06-7.96 (2 H, m, ArH), 7.72-7.62 (1 H, m, ArH), 7.57-7.47 (2 H, m, ArH), 6.10-5.96 (1 H, ddt, *J* 17.1,10.3, 6.0, vinylic CH), 5.46 (1 H, dq, *J* 17.1, 1.5, vinylic CH_2), 5.35 (1 H, dq, *J* 10.3, 1.2, vinylic CH_2), 4.88 (2 H, td, *J* 1.4, 5.8, OCH_2);δ_C (100 MHz; $CDCl_3$) 186.2 (C), 163.6 (C), 135.1 (CH), 132.5 (CH), 130.9 (C), 130.1 (CH), 129.0 (CH), 120.1 (CH_2), 66.7 (CH_2).

Cyclohexyl 2-oxo-2-phenylacetate 5i

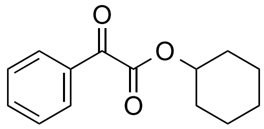

yellow liquid; Rf 0.6 (ethyl acetate : light petroleum, 1 : 24); (Found: M+Na$^+$, 255.0990. $C_{14}H_{16}NaO_3$ requires 255.0997); ν_{max} ($CHCl_3$)/cm^{-1} 3045, 2943, 2863, 1727, 1691, 1452, 1300, 1178, 1034, 1003; δ_H (400 MHz; $CDCl_3$) 8.01-7.97 (2 H, m, ArH), 7.65 (1 H, tt, *J* 7.3, 1.5, ArH), 7.51 (2 H, t, *J* 7.6, ArH), 5.14-5.06 (1 H, m, OCH), 2.07-1.94 (2 H, m, CH_2), 1.85-1.72 (2 H, m, CH_2), 1.67-1.53 (3 H, m, CH_2), 1.49-1.23 (3 H, m, CH_2); δ_C (100 MHz; $CDCl_3$) 186.9 (C), 163.8 (C), 134.9 (CH), 132.7 (C), 130.1 (CH), 129.0 (CH), 75.6 (CH), 31.6 (CH_2), 25.3 (CH_2), 23.8 (CH_2).

Ethyl 2-oxo-2-(pyridin-2-yl)acetate 5n

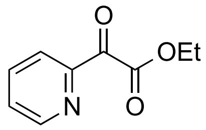

Following a procedure described in a patent,[5] a solution of 2-bromopyridine (2.0 mL, 21 mmol) in dry ether (50 mL) was prepared under anhydrous conditions and stirred at –78 °C under an argon atmosphere. Butyllithium (1.5 M; 14 mL, 21 mmol) was added over 5 min, upon which the solution showed an intense red colour. The mixture was stirred at this temperature for 30 min and subsequently transferred via a cannula to a solution of diethyl oxalate (10.0 mL, 73.6 mmol) in dry ether (100 mL) at 0 °C. The addition was completed after approximately 5 min, and the mixture was warmed to room temperature and stirred for 2 h. The reaction was quenched by slow addition of a saturated sodium hydrogen carbonate solution and the content of the reaction vessel was poured in saturated sodium hydrogen carbonate solution (60 mL). The combined organic phases were separated, washed with water (40 mL), saturated brine (20 mL) and dried over $MgSO_4$. Removal of the solvent under reduced pressure gave a liquid, principally constituted of excess diethyl oxalate. Purification by column chromatography (elution gradient ethyl acetate in light petroleum, 20% to 50%) gave the *title compound* as a red oil (781 mg, 21%); Rf 0.2 (ethyl acetate : light petroleum, 1 : 4); (Found: M+Na$^+$, 202.0480. $C_9H_9NNaO_3$ requires 202.0480); ν_{max} ($CHCl_3$)/cm^{-1} 2987, 1742, 1710, 1324, 1257, 1020; δ_H (400 MHz; $CDCl_3$) 8.72-8.78 (1 H, ddd, *J* 4.8, 1.5, 0.8, H-6 pyridine), 8.10 (1 H, dt, *J* 7.8, 1.1, H-3 pyridine), 7.90 (1 H, td, *J* 7.8, 1.5, H-4 pyridine), 7.54 (1 H, ddd, *J* 7.8, 4.8, 1.3, H-5 pyridine), 4.49 (2 H, q, *J* 7.3, C$\underline{H}_2$$CH_3$), 1.42 (3 H, t, *J* 7.3, CH_2C$\underline{H}_3$); δ_C (100 MHz; $CDCl_3$) 187.8 (C), 165.4 (C), 150.5 (C), 150.0 (CH), 137.3 (CH), 128.4 (CH), 123.5 (CH), 62.3 (CH_2), 14.2 (CH_3). The data are consistent with the literature.[5,6]

α-Ketoamide synthesis

2-Oxo-2-phenylacetyl chloride solution

Oxalyl chloride (3.0 mL, 35.0 mmol) was slowly added to a solution of phenylglyoxylic acid (5.26 g, 10.0 mmol) and dimethylformamide (6 drops) in dry dichloromethane (35 mL) under argon at 0 °C. After addition, the solution was warmed to room temperature and stirred for 4 h until the gaseous evolution has ceased. The mixture showed at this point a bright yellow coloration.

α-Ketoamide

A solution of 2-oxo-2-phenylacetyl chloride in dry dichloromethane (1M; 10 mL) was slowly added over 30 min to a solution of amine (10.0 mmol) and triethylamine (2.8 mL, 20.5 mmol) in dry dichloromethane (10 mL). The resulting mixture was warmed to room temperature and stirred for 14 h. The reaction was quenched by addition of water (5 mL) and extracted with dichloromethane (2 × 20 mL). The combined organic phases were washed with saturated brine (10 mL) and dried over $MgSO_4$. Removal of the solvent under reduced pressure gave a residue that was purified by column chromatography (ethyl acetate 10% in light petroleum ether) to give the pure α-keto amide.

***N*-Allyl-2-oxo-2-phenylacetamide 5p**

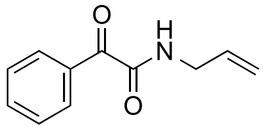

colourless solid (1.47 g, 78%); Rf 0.6 (ethyl acetate : light petroleum, 1 : 1); mp 59-60 °C (from light petroleum) (lit.,[7] mp 56-58 °C (from chloroform)); (Found: M+Na$^+$, 212.0693. $C_{11}H_{11}NNaO_2$ requires 212.0687); ν_{max} ($CHCl_3$)/cm^{-1}; 3415, 1671, 1598, 1519, 1449, 1179;δ_H (400 MHz; $CDCl_3$) 8.32 - 8.38 (2 H, m, ArH), 7.59 - 7.66 (1 H, m, ArH), 7.44 - 7.52 (2 H, m, ArH), 7.18 (1 H, br s, NH), 5.90 (1 H, ddt, *J* 17.1, 10.2, 5.7, vinylic CH), 5.28 (1 H, dq, *J*

17.1, 1.5, vinylic CH), 5.22 (1 H, dq, *J* 10.2, 1.5, vinylic CH), 4.02 (2 H, tt, *J* 5.7, 1.5, CH_2); δ_C (100 MHz; $CDCl_3$) 187.7 (C), 161.7 (C), 134.6 (CH), 133.4 (C), 133.1 (CH), 131.4 (CH), 128.6 (CH), 117.4 (CH_2), 41.9 (CH_2).

N-(2,5-Dimethoxyphenyl)-2-oxo-2-phenylacetamide 5q

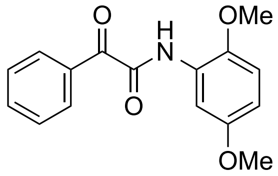

orange solid (2.56 g, 90%); Rf 0.6 (ethyl acetate : light petroleum, 1 : 1); mp 76-77 °C (from ethanol); (Found: C, 67.23; H, 5.29; N, 4.87. $C_{16}H_{15}NO_4$ requires C, 67.36; H, 5.30; N, 4.91%); (Found: M+Na$^+$, 308.0893. $C_{16}H_{15}NNaO_4$ requires 308.0893); ν_{max} ($CHCl_3$)/cm^{-1} 3374, 1674, 1532, 1486; δ_H (400 MHz; $CDCl_3$) 9.55 (1 H, br s, NH), 8.36 - 8.45 (2 H, m, ArH), 8.22 (1 H, d, *J* 3.1, ArH), 7.65 (1 H, t, *J* 7.6, ArH), 7.51 (2 H, t, *J* 7.7, ArH), 6.85 (1 H, d, *J* 8.9, ArH), 6.67 (1 H, dd, *J* 8.9, 3.1, ArH), 3.89 (3 H, s, OMe), 3.82 (3 H, s, OMe); δ_C (100 MHz; $CDCl_3$) 187.5 (C), 159.1 (C), 153.9 (C), 143.2 (C), 134.6 (CH), 133.4 (C), 131.5 (CH), 128.7 (CH), 127.1 (C), 111.1 (CH), 110.1 (CH), 106.1 (CH), 56.4 (CH_3), 56.0 (CH_3).

N-(2-Furylmethyl)-2-oxo-2-phenylacetamide 5r

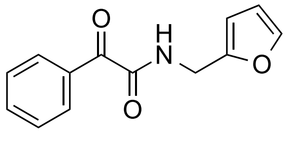

colourless solid (1.74 g, 76%); Rf 0.6 (ethyl acetate : light petroleum, 1 : 1); mp 83-84 °C (from ethanol); (Found: C, 67.97; H, 4.83; N, 6.09. $C_{13}H_{11}NO_3$ requires C, 68.11; H, 4.84; N, 6.11%);(Found: M+Na$^+$, 252.0628. $C_{13}H_{11}NNaO_3$ requires 252.0631);ν_{max} ($CHCl_3$)/cm^{-1} 3415, 1671, 1516; δ_H (400 MHz; $CDCl_3$) 8.30 - 8.38 (2 H, m, ArH), 7.61 (1 H, tt, *J* 7.5, 1.3, ArH), 7.40 - 7.53 (3 H, m, ArH and NH), 7.37 (1 H, dd, *J* 1.8, 0.8, furan H-4), 6.25 - 6.39 (2

H, m, furan H-3 and H-5), 4.56 (2 H, d, *J* 5.8, CH_2); δ_C (100 MHz; $CDCl_3$) 187.4 (C), 161.5 (C), 150.2 (C), 142.7 (CH), 134.6 (CH), 133.4 (C), 131.3 (CH), 128.6 (CH), 110.6 (CH), 108.2 (CH), 36.4 (CH_2).

SYNTHESISOF HYDRAZONES 6

3-Hydrazonoindolin-2-one **6k** (isatin hydrazone) was obtained by the method described below and the product obtained matched the previously reported data.[8]

2-Hydrazono-1-phenylethanone **8** was prepared following the procedure described by Hauptmann.[9]Benzil monohydrazone**9**was prepared following a known procedure.[10]

The stereochemistry of the C=N double bond in the hydrazones **6** was determined on the basis of their ^{1}HNMR chemical shifts and IR spectra. The data obtained for the pair of isomers were rationalized as followed: (a) the possibility of an intramolecular hydrogen bond in the (*Z*)-ketoester hydrazones (and also ketoamide and ketophosphonate) leads to a shift in the IR carbonyl resonance towards lower frequencies (in the range of 7-39 cm^{-1} lower than the (*E*)-isomer),(b) also in the (*Z*)-isomers there is deshielding of the NH_2 proton in the HNMR spectrum (1.25-2.40 ppm downfield compared to the (*E*)-isomer), and (c) a decreased capacity to form intermolecular hydrogen bond, which results in (*Z*)-hydrazone being often isolated as oils whilst (E)-hydrazone were obtained as crystalline solids. Similar shift of the $N\underline{H}_2$ ^{1}HNMR signal was observed for the ketophosphonate hydrazones and was used to determine the stereochemistry of the hydrazone observed.

General method for the synthesis of α-ketoester and α-ketoamide hydrazones

Method A:[11] Hydrazine hydrate (2 equiv) was slowly added to a mixture of glacial acetic acid (80 mL/mol) and water (80 mL/mol) cooled in an ice bath. The α-keto-ester or -amide (1 equiv) was added to the mixture at room temperature and methanol was added in order to

obtain a homogeneous solution when necessary. The reaction mixture was stirred at room temperature until completion of the reaction as judged by TLC. The volatiles were removed under reduced pressure. Water (1.6 L/mol) was added to the residue and the mixture was extracted with ethyl acetate (3.0 L/mol). The combined organic phases were washed with saturated sodium hydrogen carbonate (1.0 L/mol), saturated brine (0.5 L/mol) and dried over $MgSO_4$. The solvent was removed under reduced pressure to give a residue that was purified by to column chromatography if necessary.

Method B: Hydrazine hydrate (1 equiv) was added to a solution of benzoic acid (1 equiv) and α-keto-ester or -amide (1 equiv) in THF (3 L/mol), upon which the hydrazine benzoate precipitates in the solution. The mixture was stirred at room temperature during which the visible precipitate disappeared completely. The solution was poured into saturated sodium hydrogen carbonate solution (2.0 L/mol) and extracted with ethyl acetate (6.0 L/mol). The combined organic phases were washed with saturated brine (0.5 L/mol) and dried over $MgSO_4$. Evaporation of the solvent under reduced pressure gave a residue that was purified by column chromatography if necessary.

(*E*)- and (*Z*)-Ethyl phenylglyoxylate hydrazone 6a

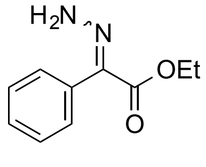

Method A, reaction time 24h;purificationelution gradient light petroleum-ethyl acetate, 7:1 to 1:1.**(*Z*)-Ethyl phenylglyoxylate hydrazone** yellow oil; Rf 0.5 (ethyl acetate : light petroleum, 2 : 23); (Found: $M+H^+$, 193.0984. $C_{10}H_{13}N_2O_2$ requires 193.0977); ν_{max} ($CHCl_3$)/cm^{-1} 3486, 3291, 3010, 1687, 1564, 1266, 1149, 1021; δ_H (400 MHz; $CDCl_3$) 8.40 (2 H, br s, NH_2), 7.54 - 7.49 (2 H, m, ArH), 7.38 - 7.26 (3 H, m, ArH), 4.31 (2 H, q, *J* 7.2, $C\underline{H}_2CH_3$), 1.33 (3 H, t, *J* 7.2, $CH_2C\underline{H}_3$); δ_C (100 MHz; $CDCl_3$) 163.0 (C), 136.8 (C), 131.3 (C), 128.3 (CH), 128.0

(CH), 127.6 (CH), 60.8 (CH_2), 14.3 (CH_3). **(*E*)-Ethyl phenylglyoxylate hydrazone** yellow solid; Rf 0.1 (ethyl acetate : light petroleum, 2 : 23); mp 96-97 °C (lit.,[12]no mp reported); (Found: M+Na$^+$, 215.0793. $C_{10}H_{12}N_2NaO_2$ requires 215.0796); ν_{max} ($CHCl_3$)/cm^{-1} 3474, 3317, 3011, 1710, 1573, 1330, 1137, 1047; δ_H (400 MHz; $CDCl_3$) 7.48 (2 H, m, ArH), 7.41 (1 H, t, *J* 7.3, ArH), 7.29 (2 H, d, *J* 7.2, ArH), 6.21 (2 H, br s, NH_2), 4.30 (2 H, q, *J* 7.2, C$\underline{H}_2$CH$_3$), 1.33 (3 H, t, *J* 7.2, CH$_2$C$\underline{H}_3$); δ_C (100 MHz; $CDCl_3$) 164.5 (C), 137.7 (C), 129.7 (C), 129.5 (CH), 129.3 (CH), 128.9 (CH), 61.4 (CH_2), 14.5 (CH_3). Data recorded are consistent with the literature.[12]

(*E*)- and (*Z*)-Ethyl 2-hydrazono-2-(4-methoxyphenyl)acetate 6b

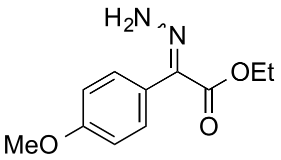

Method A, reaction time 3 h; purification elution gradient light petroleum-ethyl acetate, 19:1 to 1:1 ethyl acetate.**(*Z*)-Ethyl 2-hydrazono-2-(4-methoxyphenyl)acetate** yellow oil; Rf 0.7 (ethyl acetate : light petroleum, 1 : 4); (Found: M+Na$^+$, 245.0903. $C_{11}H_{14}N_2NaO_3$ requires 245.0902);ν_{max} ($CHCl_3$)/cm^{-1} 3485, 3293, 3008, 1688, 1609, 1565, 1513, 1300, 1268, 1250, 1177, 1147, 991, 835; δ_H (400 MHz; $CDCl_3$) 8.25 (2 H, br s, NH_2), 7.45 (2 H, d, *J* 8.8, ArH), 6.88 (2 H, d, *J* 8.8, ArH), 4.31 (2 H, q, *J* 7.2, C$\underline{H}_2$CH$_3$), 3.82 (3 H, s, OMe), 1.33 (3 H, t, *J* 7.2, CH$_2$C$\underline{H}_3$); δ_C (100 MHz; $CDCl_3$)163.1 (C), 159.3 (C), 131.5 (C), 129.5 (CH), 129.4 (C), 113.5 (CH), 60.8 (CH_2), 55.4 (CH_3), 14.3 (CH_3). **(*E*)-Ethyl 2-hydrazono-2-(4-methoxyphenyl)acetate** pale yellow solid; Rf 0.2 (ethyl acetate : light petroleum, 1 : 4); mp 91-92 °C; (Found: M+Na$^+$, 245.0899. $C_{11}H_{14}N_2NaO_3$ requires 245.0902);ν_{max} ($CHCl_3$)/cm^{-1}; 3472, 3314, 3008, 1709, 1610, 1575, 1511, 1328, 1292, 1250, 1177, 1047, 1030; δ_H (400 MHz; $CDCl_3$) 7.25 (2 H, d, *J* 8.8, ArH), 7.00 (2 H, d, *J* 8.8, ArH), 6.18 (2 H, br s, NH_2), 4.31 (2 H, q, *J* 7.2, C$\underline{H}_2$CH$_3$), 3.84 (3 H, s, OMe), 1.34 (3 H, t, *J* 7.2,CH$_2$C$\underline{H}_3$); δ_C (100 MHz;

$CDCl_3$) 164.7 (C), 137.5 (C), 130.4 (CH), 130.2 (C), 121.5 (C), 114.7 (CH), 61.4 (CH_2), 55.4 (CH_3), 14.4 (CH_3).

(*E*)- and (*Z*)-Ethyl 2-hydrazono-2-(2-methoxyphenyl)acetate 6c

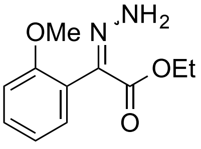

Method A,reaction time 3 h; purification elution gradient light petroleum-ethyl acetate, 19:1 to 1:1 ethyl acetate.**(*Z*)-Ethyl 2-hydrazono-2-(2-methoxyphenyl)acetate** yellow oil; Rf 0.6 (ethyl acetate : light petroleum, 1 : 4); (Found: M+Na$^+$, 245.0896. $C_{11}H_{14}N_2NaO_3$ requires 245.0902); ν_{max} ($CHCl_3$)/cm^{-1} 3484, 3299, 3007, 1693, 1570, 1493, 1465, 1269, 1243, 1147, 1113, 1029; δ_H (400 MHz; $CDCl_3$) 8.16 (2 H, br s, NH_2), 7.28-7.34 (2 H, m, ArH), 6.98 (1 H, td, *J* 7.4, 1.0, ArH), 6.87 (1 H, dd, *J* 8.7, 0.9, ArH), 4.22 (2 H, q, *J* 7.2, C$\underline{H}_2$$CH_3$), 3.78 (3 H, s, OMe), 1.19-1.25 (3 H, q, *J* 7.2, CH_2C$\underline{H}_3$); δ_C (100 MHz; $CDCl_3$) 163.1 (C), 157.8 (C), 130.7 (C), 130.1 (CH), 129.6 (CH), 126.6 (C), 120.8 (CH), 110.6 (CH), 60.4 (CH_2), 55.4(CH_3), 14.3 (CH_3). **(*E*)-Ethyl 2-hydrazono-2-(4-methoxyphenyl)acetate** yellow solid; Rf 0.1 (ethyl acetate : light petroleum, 1 : 4); mp 69-70 °C; (Found: M+Na$^+$, 245.0891. $C_{11}H_{14}N_2NaO_3$ requires 245.0902); ν_{max} ($CHCl_3$)/cm^{-1}: 3473, 3316, 3008, 1711, 1603, 1578, 1491, 1464, 1330, 1270, 1247, 1112, 1036; δ_H (400 MHz; $CDCl_3$) 7.37-7.44 (1 H, ddd, *J* 8.3, 7.5, 1.8, ArH), 7.17 (1 H, dd, *J* 7.5, 1.5, ArH), 7.05 (1 H, td, *J* 7.5, 0.9, ArH), 6.99 (1 H, d, *J* 8.3, ArH), 4.29 (2 H, q, *J* 7.2, C$\underline{H}_2$$CH_3$), 3.79 (3 H, s, OMe), 1.32 (3 H, t, *J* 7.2, CH_2C$\underline{H}_3$); δ_C (100 MHz; $CDCl_3$) 164.6 (C), 157.1 (C), 136.0 (C), 131.2 (CH), 130.3 (CH), 121.2 (CH), 118.7 (C), 111.7 (CH), 61.2 (CH_2), 55.8 (CH_3), 14.4 (CH_3).

(*E*) and (*Z*)-Ethyl 2-(4-bromophenyl)-2-hydrazonoacetate 6d

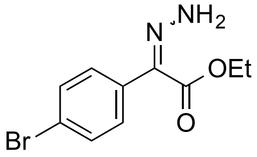

Method B, reaction time 14 h; purification elution gradient light petroleum-ethyl acetate, 9:1 to 1:1 ethyl acetate. **(*Z*)-Ethyl 2-(4-bromophenyl)-2-hydrazonoacetate** off-yellow solid; Rf 0.8 (ethyl acetate : light petroleum, 1 : 1); mp 51-52 °C; (Found: M+Na$^+$, 292.9891. $C_{10}H_{11}{}^{79}BrN_2NaO_2$ requires 292.9896); ν_{max} ($CHCl_3$)/cm^{-1} 3487, 3289, 1688, 1565, 1261, 1152; δ_H (400 MHz; $CDCl_3$) 8.52 (2 H, br s, NH_2), 7.46 (2 H, dt, *J* 8.8, 2.1, ArH), 7.40 (2 H, dt, *J* 8.8, 2.1, ArH), 4.30 (2 H, q, *J* 7.2, C$\underline{H}_2$CH$_3$), 1.32 (3 H, t, *J* 7.2, CH$_2$C$\underline{H}_3$); δ_C (100 MHz; $CDCl_3$) 162.7 (C), 135.8 (C), 131.0 (CH), 129.9 (CH), 129.7 (C), 121.6 (C), 60.9 (CH_2), 14.3 (CH_3).**(*E*)-Ethyl 2-(4-bromophenyl)-2-hydrazonoacetate** colourless solid; Rf 0.4 (ethyl acetate : light petroleum, 1 : 1); mp 106-107 °C; (Found: M+Na$^+$, 292.9893. $C_{10}H_{11}{}^{79}BrN_2NaO_2$ requires 292.9896); ν_{max} ($CHCl_3$)/cm^{-1} 3474, 3318, 3011, 1710; δ_H (400 MHz; DMSO-d_6) 7.74 (2 H, br s, NH_2), 7.62 - 7.67 (2 H, m, ArH), 7.13 - 7.20 (2 H, m, ArH), 4.11 (2 H, q, *J* 7.1, C$\underline{H}_2$CH$_3$), 1.19 (3 H, t, *J* 7.1, CH$_2$C$\underline{H}_3$); δ_C (100 MHz; DMSO-d_6) 164.3 (C), 131.7 (CH), 131.4 (CH), 130.7 (C), 130.2 (C), 121.7 (C), 59.8 (CH_2), 14.2 (CH_3). The (*E*)-hydrazone was found to undergo isomerization to the (*Z*)-hydrazone at room temperature in $CDCl_3$.

***(E*)- and (*Z*)-Ethyl 3-methyl-2-oxobutanoate hydrazone 6e**

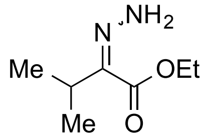

Method A, reaction time 23 h; purification elution gradient light petroleum-ethyl acetate, 7:1 to 1:1 ethyl acetate; **(*Z*)-Ethyl 3-methyl-2-oxobutanoate hydrazone** yellow oil; Rf 0.8 (ethyl acetate : light petroleum, 1 : 4); (Found: M+Na$^+$, 181.0956. $C_7H_{14}N_2NaO_2$ requires 181.0953);

ν_{max} ($CHCl_3$)/cm^{-1} 3480, 3298, 2972, 2937, 1688, 1570, 1469, 1369, 1270, 1159, 1084, 1026; δ_H (400 MHz; $CDCl_3$) 7.93 (2 H, br s, NH_2), 4.24 (2 H, q, *J* 7.1, C<u>H</u>$_2$CH$_3$), 2.90 (1 H, hept, *J* 6.8, C<u>H</u>(CH_3)$_2$), 1.33 (3 H, t, *J* 7.1, CH$_2$C<u>H</u>$_3$), 1.08 (6 H, d, *J* 6.8, CH(C<u>H</u>$_3$)$_2$); δ_C (100 MHz; $CDCl_3$) 163.0 (C), 136.4 (C), 60.2 (CH_2), 31.0 (CH), 21.1 (CH_3), 14.3 (CH_3). **(*E*)-Ethyl 3-methyl-2-oxobutanoate hydrazone** colourless solid; Rf 0.1 (ethyl acetate : light petroleum, 1 : 4); mp 80-81 °C (lit.,[13] mp 89-91°C); (Found: M+Na$^+$, 181.0969. $C_7H_{14}N_2NaO_2$ requires 181.0953); ν_{max} ($CHCl_3$)/cm^{-1} 3462, 3003, 2939, 1710, 1591, 1373, 1323, 1186, 1148, 1038; δ_H (400 MHz; $CDCl_3$) 5.97 (2 H, br s, NH_2), 4.25 (2 H, q, *J* 7.1, C<u>H</u>$_2$CH$_3$), 2.97 (1 H, hept, *J* 7.0, C<u>H</u>(CH_3)$_2$), 1.33 (3 H, t, *J* 7.1, CH$_2$C<u>H</u>$_3$), 1.25 (6 H, d, *J* 7.0, CH(C<u>H</u>$_3$)$_2$); δ_C (100 MHz; $CDCl_3$) 164.4 (C), 144.6(C), 60.9 (CH_2), 24.6 (CH), 18.4 (CH_3), 14.4 (CH_3).

(*E*)-Ethyl pyruvate hydrazone 6f

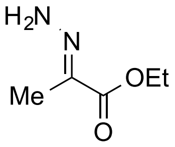

Method A, reaction time 17 h; purification elution gradient light petroleum-ethyl acetate, 1:1 to 100% ethyl acetate; colourless solid; Rf 0.1 (ethyl acetate); mp 49-50 °C; (Found: M+Na$^+$, 153.0634. $C_5H_{10}N_2NaO_2$ requires 153.0640); ν_{max} ($CHCl_3$)/cm^{-1} 3467, 3335, 3010, 1707, 1596, 1370, 1327, 1261, 1135, 1025; δ_H (400 MHz; $CDCl_3$) 5.95 (2 H, br s, NH_2), 4.26 (2 H, q, *J* 7.2, C<u>H</u>$_2$CH$_3$), 1.94 (3 H, s, CH_3), 1.32 (3 H, t, *J* 7.2, CH$_2$C<u>H</u>$_3$); δ_C (100 MHz; $CDCl_3$) 165.1 (C), 137.1 (C), 61.4 (CH_2), 14.4 (CH_3), 9.6 (CH_3).

(*E*)- and (*Z*)-Ethyl 2-oxo-4-phenylbutanoate hydrazone 6g

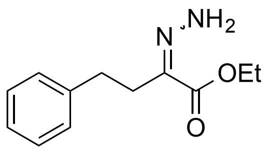

Method A, reaction time 1 h; purification elution gradient light petroleum-ethyl acetate, 7:1 to 1:1 ethyl acetate;**(*Z*)-Ethyl 2-oxo-4-phenylbutanoate hydrazone** yellow oil; Rf 0.8 (ethyl acetate : light petroleum, 2 : 3); (Found: M+Na$^+$, 243.1094. $C_{12}H_{16}N_2NaO_2$ requires 243.1109); ν_{max} ($CHCl_3$)/cm^{-1} 3483, 3298, 2986, 2937, 1690, 1570, 1303, 1179, 1120; δ_H (400 MHz; $CDCl_3$) 8.09 (2 H, br s, NH_2), 7.32-7.26 (2 H, m, ArH), 7.24-7.16 (3 H, m, ArH), 4.22 (2 H, q, *J* 7.2, $C\underline{H}_2CH_3$), 2.87-2.79 (2 H, m, CH_2), 2.73-2.66 (2 H, m, CH_2), 1.32 (3 H, t, *J* 7.2, $CH_2C\underline{H}_3$); δ_C (100 MHz; $CDCl_3$) 163.0 (C), 142.1 (C), 130.8 (C), 128.6 (CH), 128.5 (CH), 126.0 (CH), 60.4 (CH_2), 35.3 (CH_2), 34.4 (CH_2), 14.3 (CH_3). **(*E*)-Ethyl 2-oxo-4-phenylbutanoate hydrazone** yellow oil; Rf 0.4 (ethyl acetate : light petroleum, 2 : 3); (Found: C, 65.32; H, 7.35; N, 12.42. $C_{12}H_{16}N_2NaO_2$ requires C, 65.43; H, 7.32; N, 12.72%); (Found: M+Na$^+$, 243.1082. $C_{12}H_{16}N_2NaO_2$ requires 243.1109); ν_{max} ($CHCl_3$)/cm^{-1} 3463, 3328, 3010, 1705, 1596, 1376, 1327, 1257, 1176, 1097, 1068; δ_H (400 MHz; $CDCl_3$) 7.36-7.29 (2 H, m, ArH), 7.28-7.21 (3 H, m, ArH), 5.79 (2 H, br s, NH_2), 4.31 (2 H, q, *J* 7.2, $C\underline{H}_2CH_3$), 2.88-2.75 (4 H, m, CH_2), 1.37 (3 H, t, *J* 7.2, $CH_2C\underline{H}_3$); δ_C (100 MHz; $CDCl_3$) 164.9 (C), 141.0 (C), 139.8 (C) 128.9 (CH), 128.4 (CH), 126.6 (CH), 61.3 (CH_2), 31.4 (CH_2), 26.4 (CH_2), 14.4 (CH_3).

(*E*)- and (*Z*)-Allyl 2-hydrazono-2-phenylacetate 6h

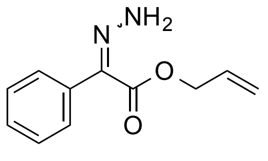

Method A; reaction time 5 h; purification elution gradient light petroleum-ethyl acetate, 19:1 to 1:1 ethyl acetate.**(*Z*)-Allyl 2-hydrazono-2-phenylacetate** yellow oil; Rf 0.4 (ethyl acetate : light petroleum, 1 : 24); (Found: M+Na$^+$, 227.0793. $C_{11}H_{12}N_2NaO_2$ requires 227.0796); ν_{max} ($CHCl_3$)/cm^{-1} 3486, 3292, 3012, 1688, 1566, 1295, 1265, 1149, 1006, 937;δ_H (400 MHz; $CDCl_3$) 8.45 (2 H, br s, NH_2), 7.49-7.55 (2 H, m, ArH), 7.27-7.38 (3 H, m, ArH), 5.98 (1 H,

ddt, J 17.2, 10.6, 5.8, vinylic CH), 5.32 (1 H, dd, J 17.2, 1.3, vinylic CH), 5.25 (1 H, dd, J 10.6, 1.3, vinylic CH), 4.75 (2 H, dt, J 5.8, 1.3, CH_2); δ_C (100 MHz; $CDCl_3$) 162.6 (C), 136.7 (C), 131.7 (CH), 130.8 (C), 128.4 (CH), 128.0 (CH), 127.7 (CH), 118.8 (CH_2), 65.3 (CH_2).

(*E*)-Allyl 2-hydrazono-2-phenylacetate yellow oil; Rf 0.1 (ethyl acetate : light petroleum, 1 : 24); (Found: M+Na$^+$, 227.0795. $C_{11}H_{12}N_2NaO_2$ requires 227.0796); ν_{max} ($CHCl_3$)/cm^{-1} 3475, 3317, 3009, 1712, 1572, 1370, 1325, 1310, 1240, 1138, 1042, 1020; δ_H (400 MHz; $CDCl_3$) 7.46-7.53 (2 H, m, ArH), 7.42 (1 H, t, J 7.3, ArH), 7.28-7.33 (2 H, m, ArH), 6.27 (2 H, br s, NH_2), 5.91-6.04 (1 H, ddt, J 17.2, 10.3, 5.8, vinylic CH), 5.31 (1 H, dq, J 17.2, 1.4, vinylic CH), 5.23 (1 H, dq, J 10.3, 1.4, vinylic CH), 4.74 (2 H, dt, J 5.8, 1.4, CH_2); δ_C (100 MHz; $CDCl_3$) 164.2 (C), 137.4 (C), 132.2 (CH), 129.6 (C) 129.5 (CH), 129.3 (CH), 129.0 (CH), 118.8 (CH_2), 66.0 (CH_2).

(*E*)- and (*Z*)-cyclohexyl 2-hydrazono-2-phenylacetate 6i

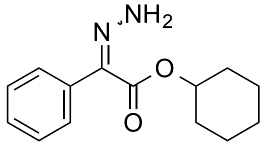

Method A,reaction time 5 h; purification elution gradient light petroleum-ethyl acetate, 19:1 to 1:1 ethyl acetate.**(*Z*)-Cyclohexyl 2-hydrazono-2-phenylacetate** colourless solid; Rf 0.4 (ethyl acetate : light petroleum, 1 : 24); mp 57-58 °C; (Found: M+Na$^+$, 269.1256. $C_{14}H_{18}N_2NaO_2$ requires 269.1266); ν_{max} ($CHCl_3$)/cm^{-1} 3485, 3288, 3009, 2941, 2862, 1681, 1562, 1293, 1263, 1162, 1150, 1121; δ_H (400 MHz; $CDCl_3$) 8.34 (2 H, br s, NH_2), 7.50-7.56 (2 H, m, ArH), 7.26-7.38 (3 H, m, ArH), 4.96-5.04 (1 H, m, OC<u>H</u>), 1.83-1.94 (2 H, m, CH_2), 1.63 - 1.75 (2 H, m, CH_2), 1.22-1.59 (6 H, m, CH_2); δ_C (100 MHz; $CDCl_3$) 162.5 (C), 136.9 (C), 132.0 (C), 128.3 (CH), 127.9 (CH), 127.5 (CH), 73.4 (CH), 31.6 (CH_2), 25.4 (CH_2), 23.7 (CH_2). **(*E*)-Cyclohexyl 2-hydrazono-2-phenylacetate** colourless solid; Rf 0.1 (ethyl acetate : light petroleum, 1 : 24); mp 117-118 °C; (Found: M+Na$^+$, 269.1251. $C_{14}H_{18}N_2NaO_2$ requires

269.1266); ν_{max} ($CHCl_3$)/cm^{-1} 3473, 3316, 3008, 2940, 2862, 1703, 2573, 1338, 1304, 1240, 1138, 1042, 1018;δ_H (400 MHz; $CDCl_3$) 7.44-7.49 (2 H, m, ArH), 7.37-7.44 (1 H, m, ArH), 7.30 (2 H, dd, *J* 8.3, 1.3, ArH), 6.19 (br s, 2 H, NH_2), 4.84-4.96 (1 H, m, OC<u>H</u>), 1.90 (2 H, dd, *J* 8.5, 4.0, CH_2), 1.68 (2 H, dd, *J* 8.4, 4.1, CH_2), 1.14-1.58 (6 H, m, CH_2); δ_C (100 MHz; $CDCl_3$) 164.0 (C), 138.3 (C), 130.0 (C), 129.3 (CH), 129.2 (CH), 128.9 (CH), 73.8 (CH), 31.7 (CH_2), 25.5 (CH_2), 24.0 (CH_2).

(*Z*)- and (*E*)-3-Hydrazono-4,4-dimethyldihydrofuran-2(*3H*)-one 6j

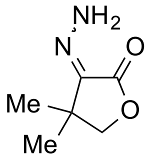

Method B, reaction time 20 h; purification: elution gradient, ethyl acetate : light petroleum ether 1:1 to 9:1. **(*Z*)-3-Hydrazono-4,4-dimethyldihydrofuran-2(*3H*)-one** yellow oil; Rf 0.4 (ethyl acetate : light petroleum, 1 : 1); (Found: M+Na^+, 165.0637. $C_6H_{10}N_2NaO_2$ requires 165.0634); ν_{max} ($CHCl_3$)/cm^{-1} 3485, 1732, 1593, 1124, 1010; δ_H (400 MHz; $CDCl_3$) 7.98 (2 H, br s, NH_2), 4.14 (2 H, s, CH_2), 1.25 (6 H, s, CH_3); δ_C (100 MHz; $CDCl_3$) 165.0 (C), 134.7 (C), 79.5 (CH_2), 37.9 (C), 26.2 (CH_3). **(*E*)-3-Hydrazono-4,4-dimethyldihydrofuran-2(*3H*)-one** colourless solid; Rf 0.2 (ethyl acetate); mp 159-160 °C; (Found: M+Na^+, 165.0637. $C_6H_{10}N_2NaO_2$ requires 165.0634); ν_{max} ($CHCl_3$)/cm^{-1} 3476, 3356, 1771, 1610; δ_H (400 MHz; DMSO-d_6) 7.97 (2 H, s, NH_2), 3.94 (2 H, s, CH_2), 1.32 (6 H, s, CH_3); δ_C (100 MHz; DMSO-d_6) 167.5 (C), 132.9 (C), 77.3 (CH_2), 36.4 (C), 21.3 (CH_3).

(*E*) and (*Z*)-3-hydrazonoindolin-2-one 6k

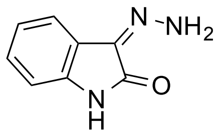

Method B, reaction time 3 h; obtained as a mixture of (*E*) and (*Z*)-isomers after an aqueous work-up (ratio E/Z: 89/11, isomerizes upon standing in favour of the (*Z*)-isomer). Orange solid; Rf 0.4 and 0.2 (ethyl acetate : light petroleum, 1 : 1); (Found: M+Na$^+$, 184.0480. $C_8H_7N_3NaO$ requires 184.0487); ν_{max} (ATR)/cm^{-1} 1686, 1587, 1555, 1463, 1204; δ_H (400 MHz; DMSO-d_6) (*E*)-isomer: 10.35 (1 H, s, NH), 8.76 (2 H, br s, NH_2), 7.90 (1 H, d, *J*7.5, ArH), 7.20 (1 H, td, *J* 7.8, 1.0, ArH), 6.96 (1 H, td, J 7.5, 0.9, ArH), 6.83 (1 H, d, *J* 7.8, ArH), (*Z*)-isomer: 10.68 (1 H, s, NH), 10.53 (1 H, d, *J* 14.0, NH_2), 9.54 (1 H, d, *J* 14.0, NH_2), 7.35 (1 H, d, *J* 7.5, ArH), 7.15 (1 H, td, *J* 7.8, 1.2, ArH), 6.97 (1 H, dt, *J* 7.5, 1.0, ArH), 6.83 - 6.87 (1 H, m, ArH overlapping with isomer signal); δ_C (100 MHz; DMSO-d_6) (*E*)-isomer: 162.8 (C), 138.7 (C), 127.1 (CH), 126.2 (C), 122.3 (C), 121.4 (CH), 117.5 (CH), 110.0 (CH); (*Z*)-isomer: 165.8 (C), 140.5 (C), 128.7 (CH), 128.5 (C), 122.7 (CH),121.0 (CH), 116.9 (C), 109.6 (CH).

Following a previously described procedure,[8] the (*Z*)-isomer was obtained as a yellow solid; Rf 0.4 (ethyl acetate : light petroleum, 1 : 1); mp 210 °C (decomp) (lit.,[8] mp 226 °C); (Found: M+Na$^+$, 184.0481. $C_8H_7N_3NaO$ requires 184.0487); ν_{max} (ATR)/cm^{-1} 3134, 1683, 1656, 1586, 1548, 1465, 1191, 978, 746, 678;δ_H (300 MHz; DMSO-d_6) 10.68 (1 H, s, NH), 10.54 (1 H, d, *J* 14.0, NH_2), 9.54 (1 H, d, *J* 14.0, NH_2), 7.35 (1 H, d, *J* 7.5, ArH), 7.15 (1 H, dt, *J* 7.8, 1.2, ArH), 6.97 (1 H, dt, *J* 7.5, 1.0, ArH), 6.86 (1 H, dt, *J* 7.8, 1.0, ArH); δ_C (75 MHz; DMSO-d_6) 162.8 (C), 138.6 (C), 127.0 (CH), 126.2 (C), 122.2 (C), 121.3 (CH), 117.4 (CH), 110.0 (CH).

(*E*)- and (*Z*)-3-Hydrazono-1-methylindolin-2-one 6l

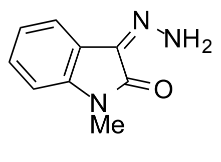

Method B, reaction time 1 h; obtained as a mixture of (*E*) and (*Z*)-isomers after an aqueous work-up (ratio E/Z : 85/15). Red solid; Rf 0.6 and 0.1 (ethyl acetate : light petroleum, 1 : 1); (Found: M+Na$^+$, 198.0644. $C_9H_9N_3NaO$ requires 198.0638); ν_{max} (ATR)/cm^{-1} 1691, 1675, 1607, 1588, 1489, 1468;δ_H (400 MHz; DMSO-*d*$_6$) (*E*)-isomer: 8.87 (2 H, br s, NH_2), 7.95 (1 H, d, *J* 7.5, ArH), 7.29 (1 H, t, *J* 7.8, ArH), 7.04 (1 H, d, *J* 7.5, ArH), 7.01 (1 H, *J* 7.8, ArH), 3.15 (3 H, s, CH_3), (*Z*)-isomer: 10.51 (1 H, d, *J* 14.3, NH_2), 9.62 (1 H, d, *J* 14.3, NH_2), 7.40 (1 H, d, *J* 7.7, ArH), 7.24 (1 H, t, *J* 7.5, ArH), 6.94 - 7.10 (2 H, m, ArH, overlapping with isomeric signals); δ_C (100 MHz; DMSO-*d*$_6$) (*E*)-isomer : 164.4 (C), 141.6 (C), 128.6 (CH), 127.6 (C), 122.3 (CH), 121.5 (CH), 116.1 (C), 108.2 (CH), 25.6 (CH_3); (*Z*)-isomer: 160.8 (C), 140.0 (C), 127.0 (CH), 125.4 (C), 121.9 (CH), 121.3 (C), 117.2 (CH), 108.6 (CH), 25.1 (CH_3).

(*E*)- and (*Z*)-Ethyl 2-hydrazono-2-(thienyl)acetate 6m

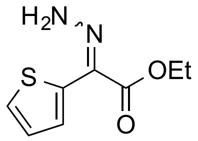

Method B, reaction time 39 h, purification: elution gradient 10 to 40% ethyl acetate in light petroleum ether. **(*Z*)-Ethyl 2-hydrazono-2-(thienyl)acetate** yellow solid; Rf 0.6 (ethyl acetate : light petroleum, 1 : 1); mp 45 - 46 °C;(Found: M+Na$^+$, 221.0352. $C_8H_{10}N_2NaO_2S$ requires 221.0355); ν_{max} ($CHCl_3$)/cm^{-1} 3485, 3011, 1688, 1287, 1155; δ_H (400 MHz; $CDCl_3$) 8.48 (2 H, br s, NH_2), 7.40 (1 H, dd, *J* 3.7, 1.1, H-5 thiophene), 7.17 (1 H, dd, *J* 5.1,1.0, H-3 thiophene), 6.98 (1 H, dd, *J* 5.1, 3.7, H-4 thiophene), 4.37 (2 H, q, *J* 7.2, $C\underline{H}_2CH_3$), 1.42 (3 H, t, *J* 7.2, $CH_2C\underline{H}_3$); δ_C (100 MHz; $CDCl_3$) 162.0 (C), 140.9 (C), 127.2 (CH), 126.2 (C), 125.2 (CH), 124.7 (CH), 61.1 (CH_2), 14.3 (CH_3).**(*E*)-Ethyl 2-hydrazono-2-(thienyl)acetate**: yellow solid; Rf 0.4 (ethyl acetate : light petroleum, 1 : 1); mp 63 – 64 °C; (Found: M+Na$^+$, 221.0352. $C_8H_{10}N_2NaO_2S$ requires 221.0355); ν_{max} ($CHCl_3$)/cm^{-1} 1711, 1239; δ_H (400 MHz; $CDCl_3$)7.53 (1 H, dd, *J* 5.1, 1.1, H-3 thiophene), 7.32 (1 H, dd, *J* 3.6, 1.1, H-5 thiophene),

7.15 (1 H, dd, *J* 5.1, 3.6, H-4 thiophene), 6.75 (2 H, br s, NH_2), 4.33 (2 H, q, *J* 7.2, C$\underline{H}_2$$CH_3$), 1.36 (3H, t, *J* 7.2, CH_2C$\underline{H}_3$); δ_C (100 MHz; $CDCl_3$)164.3 (C), 130.5 (C), 129.2 (CH), 128.7 (C), 128.6 (CH), 127.0 (CH), 61.7 (CH_2), 14.4 (CH_3).

(*E*)- and (*Z*)-Ethyl 2-hydrazono-2-(pyridin-2-yl)acetate 6n

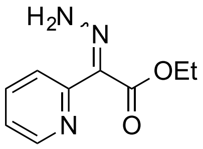

Method B, reaction time 12 h; obtained as a mixture of (*E*) and (*Z*)-isomers after an aqueous work-up (ratio E/Z: 9/91). Yellow solid; Rf 0.4 and 0.2 (ethyl acetate : light petroleum, 1 : 1); (Found: M+Na^+, 216.0745. $C_9H_{11}N_3NaO_2$ requires 216.0743); ν_{max} ($CHCl_3$)/cm^{-1} 3470, 3006, 1703, 1571, 1267; δ_H (400 MHz; $CDCl_3$) (*Z*)-isomer: 9.51 (2 H, s, NH_2) 8.55 (1 H, ddd, *J*4.9, 1.9, 1.0, pyridine H-6), 8.02 (1 H, dt, *J* 8.3, 1.0, pyridine H-3), 7.77 (1 H, ddd, *J* 8.3, 7.6, 1.9, pyridine H-4), 7.21 (1 H, ddd, *J* 7.6, 4.9, 1.0, pyridine H-5), 4.35 (2 H, q, *J* 7.2, C$\underline{H}_2$$CH_3$), 1.38 (3 H, t, *J* 7.2, CH_2C$\underline{H}_3$), (*E*)-isomer: 8.57 - 8.60 (1 H, m, pyridine H-6), 8.46(2 H, s, NH_2), 7.66 (2 H, td, *J* 7.5, 1.8, pyridine H-3), 7.55 (1 H, ddd, *J* 7.9, 7.3, 1.1, pyridine H-4), 7.17 (1 H, ddd, *J* 7.5, 4.9, 1.4, pyridine H-5), 4.32 (2 H, d, *J* 7.2,C$\underline{H}_2$$CH_3$), 1.29 (3 H, t, *J* 7.2, CH_2C$\underline{H}_3$);δ_C (100 MHz; $CDCl_3$) (*Z*)-isomer: 165.4 (C), 152.5 (C), 146.8 (CH), 136.7 (CH), 127.9 (C), 124.4 (CH), 122.4 (CH), 61.1 (CH_2), 14.5 (CH_3); (*E*)-isomer: 162.9 (C), 149.0 (CH), 136.1 (CH), 124.2 (C), 123.0 (CH), 122.3 (CH), 60.9 (CH_2), 14.2 (CH_3), one quaternary carbon was not detected for the (*E*)-isomer.

(*E*)- and (*Z*)-*tert*-Butyl 3-(1-hydrazono-2-methoxy-2-oxoethyl)indole-1-carboxylate 6o

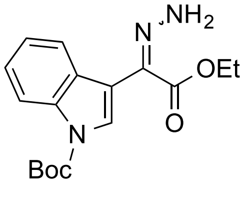

Method B,reaction time 20 h; purification: ethyl acetate 25% in light petroleum ether.**(*Z*)-*tert*-Butyl 3-(1-hydrazono-2-methoxy-2-oxoethyl)indole-1-carboxylate** yellow oil; Rf 0.7 (ethyl acetate : light petroleum, 1 : 1); (Found: M+Na$^+$, 340.1265. $C_{16}H_{19}N_3NaO_4$ requires 340.1268); ν_{max} ($CHCl_3$)/cm^{-1} 1729, 1699, 1452, 1381, 1251, 1157, 1102; δ_H (400 MHz; $CDCl_3$) 8.54 (2 H, br s, NH_2), 8.14 (1 H, d, *J* 8.0, ArH), 8.08 (1 H, d, *J* 7.9, ArH), 7.92 (1 H, s, ArH indole H-2), 7.29 - 7.36 (1 H, m, ArH), 7.21 - 7.29 (1 H, m, ArH), 3.89 (3 H, s, OMe), 1.69 (9 H, s, *t*-Bu); δ_C (100 MHz; $CDCl_3$) 163.2 (C), 149.9 (C), 135.3 (C), 129.1 (C), 125.6 (C), 125.4 (CH), 124.6 (CH), 123.0 (CH), 122.2 (CH), 116.4 (C), 115.0 (CH), 83.9 (C), 51.7 (CH_3), 28.3 (CH_3). **(*E*)-*tert*-Butyl 3-(1-hydrazono-2-methoxy-2-oxoethyl)indole-1-carboxylate** yellow solid; Rf 0.4 (ethyl acetate : light petroleum, 1 : 1); mp 132-134 °C;(Found: M+Na$^+$, 340.1272. $C_{16}H_{19}N_3NaO_4$ requires 340.1268); ν_{max} ($CHCl_3$)/cm^{-1} 1734, 1374, 1154, 1102; δ_H (400 MHz; $CDCl_3$) 8.21 (1 H, d, *J* 8.3, ArH), 7.76 (1 H, s, ArH indole H-2), 7.32 - 7.39 (2 H, m, ArH), 7.22 - 7.28 (1 H, m, ArH), 6.41 (2 H, s, NH_2), 3.86 (3 H, s, OMe), 1.68 (9 H, s, *t*-Bu); δ_C (100 MHz; $CDCl_3$) 165.2 (C), 149.3 (C), 135.4 (C), 130.7 (C), 127.4 (C), 127.1 (CH), 125.2 (CH), 123.2 (CH), 120.8 (CH), 115.8 (CH), 108.8 (C), 84.7 (C), 52.6 (CH_3), 28.3 (CH_3).

(*E*)- and (*Z*)-*N*-Allyl-2-hydrazono-2-phenylacetamide 6p

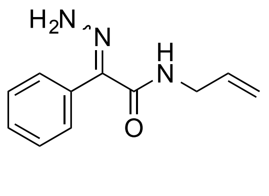

Method B, reaction time 20 h; purification: ethyl acetate 30% in light petroleum ether. **(*Z*)-*N*-Allyl-2-hydrazono-2-phenylacetamide** slightly yellow solid; Rf 0.3 (ethyl acetate : light petroleum, 2 : 3); mp 45-46 °C; (Found: M+Na$^+$, 226.0949. $C_{11}H_{13}N_3NaO$ requires 226.0951); ν_{max} ($CHCl_3$)/cm^{-1} 3431, 3003, 1656, 1556, 1511, 1168; δ_H (400 MHz; $CDCl_3$) 8.05 (2 H, br s, NH_2), 7.42 - 7.49 (2 H, m, ArH), 7.29 - 7.41 (3 H, m, ArH), 5.87 (1 H, br s,

NH), 5.80 (1 H, ddt, *J* 17.1, 10.3, 5.7, vinylic CH), 5.09 - 5.19 (2 H, m, vinylic CH), 3.91 (2 H, tt, *J* 5.7, 1.5, CH_2); δ_C (100 MHz; $CDCl_3$) 163.7 (C), 136.6 (C), 135.3 (C), 133.6 (CH), 128.9 (CH), 128.5 (CH), 128.1 (CH), 116.8 (CH_2), 41.4 (CH_2). **(*E*)-*N*-Allyl-2-hydrazono-2-phenylacetamide** yellow oil; Rf 0.1 (ethyl acetate : light petroleum, 2 : 3); (Found: M+Na^+, 226.0950. $C_{11}H_{13}N_3NaO$ requires 226.0951); ν_{max} ($CHCl_3$)/cm^{-1} 3419,3006, 1663, 1516; δ_H (400 MHz; $CDCl_3$) 7.42 - 7.49 (2 H, m, ArH), 7.35 - 7.41 (1 H, m, ArH), 7.28 - 7.33 (2 H, m, ArH), 7.08 (1 H, br s, NH), 5.86 - 5.95 (3 H, m, vinylic CH and N<u>H</u>$_2$), 5.23 (1 H, dq, *J* 17.1, 1.6, vinylic CH), 5.14 (1 H, dq, *J* 10.2, 1.4, vinylic CH), 3.96 (2 H, tt, *J* 5.8, 1.6, CH_2); δ_C (100 MHz; $CDCl_3$) 164.2 (C), 140.8 (C), 134.7 (CH), 129.3 (CH), 129.1 (CH), 129.0 (CH), 116.2 (CH_2), 41.8 (CH_2), one quaternary carbon signal was not observed.

(*E*)- and (*Z*)-*N*-(2,5-Dimethoxyphenyl)-2-hydrazono-2-phenylacetamide 6q

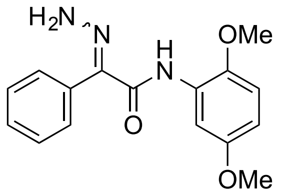

Method B, reaction time 20 h; purification: ethyl acetate 25% in light petroleum ether.**(*Z*)-*N*-(2,5-Dimethoxyphenyl)-2-hydrazono-2-phenylacetamide** yellow solid; Rf 0.6 (ethyl acetate : light petroleum, 1 : 1); mp 114-115 °C; (Found: M+Na^+, 322.1158. $C_{16}H_{17}N_3NaO_3$ requires 322.1162); ν_{max} ($CHCl_3$)/cm^{-1} 3481, 3389, 1660, 1601, 1530, 1482; δ_H (400 MHz; $CDCl_3$) 8.36 (2 H, br s, NH_2), 8.16 (1 H, br s, NH), 8.14 (1 H, d, *J* 2.9, ArH), 7.55 (2 H, dd, *J* 8.0, 1.3, ArH), 7.35 - 7.48 (3 H, m, ArH), 6.73 (2 H, d, *J* 8.9, ArH), 6.59 (1 H, dd, *J* 8.9, 2.9, ArH), 3.81 (3 H, s, OMe), 3.63 (3 H, s, OMe); δ_C (100 MHz; $CDCl_3$) 161.8 (C), 153.9 (C), 142.7 (C), 136.5 (C), 134.9 (C), 128.9 (CH), 128.6 (CH), 128.5 (CH), 127.7 (C), 111.1 (CH), 109.2 (CH), 106.3 (CH), 56.4 (CH_3), 55.9 (CH_3). **(*E*)-*N*-(2,5-Dimethoxyphenyl)-2-hydrazono-2-phenylacetamide** off-yellow solid; Rf 0.5 (ethyl acetate : light petroleum, 1 : 1); mp 115-116 °C; (Found: M+Na^+, 322.1155. $C_{16}H_{17}N_3NaO_3$ requires

322.1162); ν_{max} ($CHCl_3$)/cm^{-1} 3467, 3374, 1671, 1601, 1531, 1486; δ_H (400 MHz; $CDCl_3$) 9.61 (1 H, br s, NH), 8.22 (1 H, d, *J* 3.0, ArH), 7.48 - 7.54 (2 H, m, ArH), 7.40 - 7.46 (1 H, m, ArH), 7.33 - 7.38 (2 H, m, ArH), 6.82 (1 H, d, *J* 8.9, ArH), 6.57 (1 H, dd, *J* 8.9, 3.0, ArH), 5.98 (2 H, br s, NH_2), 3.89 (3 H, s, OMe), 3.75 (3 H, s, OMe); δ_C (100 MHz; $CDCl_3$) 162.2 (C), 154.0 (C), 142.6 (C), 141.2 (C), 129.5 (CH), 129.2 (CH), 129.1 (CH), 128.9 (C), 128.7 (C), 111.1 (CH), 108.8 (CH), 105.0 (CH), 56.5 (CH_3), 55.9 (CH_3).

(*E*)- and (*Z*)-*N*-(2-Furylmethyl)-2-hydrazono-2-phenylacetamide 6r

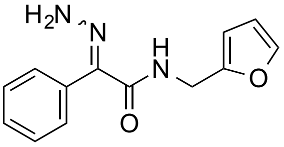

Method B, reaction time 20 h; purification: ethyl acetate 40% in light petroleum ether; **(*Z*)-*N*-(2-Furylmethyl)-2-hydrazono-2-phenylacetamide** yellow oil; Rf 0.5 (ethyl acetate : light petroleum, 1 : 1); (Found: M+Na$^+$, 266.0894. $C_{13}H_{13}N_3NaO_2$ requires 266.0900); ν_{max} ($CHCl_3$)/cm^{-1} 3480, 3431, 3011, 1656, 1514; δ_H (400 MHz; $CDCl_3$) 8.02 (2 H, br s, NH_2), 7.39 - 7.47 (2 H, m, ArH), 7.28 - 7.39 (4 H, m, ArH and furan H-3), 6.29 (1 H, dd, *J* 3.1, 1.9, furan H-4), 6.20 (1 H, d, *J* 3.1, furan H-5), 6.16 (1 H, br s, NH), 4.45 (2 H, d, *J* 5.8, CH_2); δ_C (100 MHz; $CDCl_3$) 163.6 (C), 150.7 (C), 142.3 (CH), 136.4 (C), 135.1 (C), 128.8 (CH), 128.4 (CH), 128.0 (CH), 110.5 (CH), 107.6 (CH), 35.9 (CH_2). **(*E*)-*N*-(2-Furylmethyl)-2-hydrazono-2-phenylacetamide** pale yellow solid; Rf 0.4 (ethyl acetate : light petroleum, 1 : 1); mp 92-93 °C (Found: M+Na$^+$, 266.0888. $C_{13}H_{13}N_3NaO_2$ requires 266.0900); ν_{max} ($CHCl_3$)/cm^{-1}3420, 3011, 1663, 1514; δ_H (400 MHz; $CDCl_3$) 7.42 - 7.50 (2 H, m, ArH), 7.27 - 7.42 (5 H, m, ArH and NH), 6.33 (1 H, dd, *J* 3.1, 1.9, ArH), 6.26 (1 H, dd, *J* 3.1, 0.6, ArH), 5.85 (2 H, br s, NH_2), 4.52 (2 H, d, *J* 5.8, CH_2); δ_C (100 MHz; $CDCl_3$) 164.2 (C), 151.8 (C), 142.2 (CH), 140.8 (C), 129.4 (CH), 129.2 (CH), 129.03 (CH), 128.99 (C), 110.5 (CH), 107.4 (CH), 36.5 (CH_2).

(*E*)-Dimethyl 1-hydrazonoethylphosphonate 7a

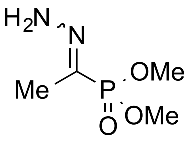

Trimethylphosphite (3.9 mL, 33.4 mmol) was slowly added to acetyl chloride (2.4 mL, 33.4 mmol) over 30 min under an atmosphere of argon at 0 °C and the mixture was heated at 65 °C for 1 h. After cooling, methanol (40 ml) was added to the mixture and a solution of hydrazine hydrate (3.2 mL, 65.9 mmol) in methanol (60 mL) and glacial acetic acid (6 mL) was added over 20 min. The mixture was then stirred for 14 h at room temperature. The solvent was removed under reduced pressure to give a residue that was subjected to column chromatography (8% methanol in ethyl acetate) to give *the title compound*as a colourless solid (3.31 g, 59%), Rf 0.4 (methanol : ethyl acetate, 2 : 23); mp 68-69 °C, (Found: M+Na$^+$, 189.0407. $C_4H_{11}N_2NaO_3P$ requires 189.0405); ν_{max} ($CHCl_3$)/cm^{-1} 3463, 3006, 2956, 1250, 1037, 837; δ_H (400 MHz; $CDCl_3$) 6.02 (2 H, br s, NH_2), 3.75 (6 H, dt, *J* 10.8, 0.9, OMe), 1.88 (3 H, dt, *J* 11.0 0.9, CH_3); δ_C (100 MHz; $CDCl_3$) 137.7 (d, J_{CP} 238, C), 53.0 (d, J_{CP} 5.8, CH_3), 11.4 (d, J_{CP} 22, CH_3);δ_P (162 MHz; $CDCl_3$) 14.8 (m).

***(E*)- and (*Z*)-Diethyl 1-hydrazono-2-phenylethylphosphonate 7b**

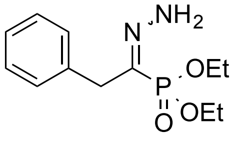

Triethylphosphite (1.71 mL, 10 mmol) was added to 2-phenylacetyl chloride (1.33 mL, 10 mmol) over 5 min at 0 °C under an argon atmosphere. The mixture was subsequently stirred at room temperature for 2 h, upon which absolute ethanol (10 mL) was added. The solution obtained was added at 0 °C to a solution of hydrazine hydrate (0.49 mL, 10 mmol) in a mixture of ethanol and acetic acid (10:1, 11 mL). The resulting mixture was then stirred at

room temperature for 20 h, poured into water (20 mL), extracted with dichloromethane (3 × 50 mL), washed with saturated brine (15 mL) and dried over $MgSO_4$. Removal of the solvent under reduced pressure gave an oil which was purified by column chromatography (elution gradient ethyl acetate in light petroleum ether 50% to 100%) to give the (*Z*)- and (*E*)-hydrazones in order of elution. **(*Z*)-Diethyl 1-hydrazono-2-phenylethylphosphonate** yellow liquid (472 mg, 17%); Rf 0.5 (ethyl acetate); (Found: M+Na$^+$, 293.1012. $C_{12}H_{19}N_2NaO_3P$ requires 293.1026); ν_{max} ($CHCl_3$)/cm^{-1} 2999, 1240, 1046, 1022, 975; δ_H (400 MHz; $CDCl_3$) 7.45 (2 H, br s, NH_2), 7.15 - 7.33 (5 H, m, ArH), 3.92 - 4.04 (2 H, m, CH_2), 3.73 - 3.85 (4 H, m, $C\underline{H}_2CH_3$), 3.62 (2 H, d, *J* 10.7, CH_2), 1.18 (6 H, t, *J* 7.0, $CH_2C\underline{H}_3$); δ_C (100 MHz; $CDCl_3$) 138.4 (C), 129.2 (CH), 128.4 (CH), 126.6 (CH), 62.2 (d, J_{CP} 5.1, CH_2), 41.0 (d, J_{CP} 26.0, CH_2), 16.2 (d, J_{CP} 6.6, CH_3), one quaternary carbon signal was not observed; δ_P (162 MHz; $CDCl_3$) 8.2 (m).**(*E*)-Diethyl 1-hydrazono-2-phenylethylphosphonate** yellow solid (1.893 g, 70%); Rf 0.1 (ethyl acetate); mp 42-43 °C; (Found: M+Na$^+$, 293.1012. $C_{12}H_{19}N_2NaO_3P$ requires 293.1026); ν_{max} ($CHCl_3$)/cm^{-1} 2998, 1246, 1049, 1027, 972 δ_H (400 MHz; $CDCl_3$) 7.16 - 7.32 (5 H, m, ArH), 6.19 (2 H, br s, NH_2), 4.01 - 4.18 (4 H, m, $C\underline{H}_2CH_3$), 3.73 (2 H, d, *J* 12.0, CH_2), 1.27 (6 H, t, *J* 7.0, $CH_2C\underline{H}_3$); δ_C (100 MHz; $CDCl_3$) 134.7 (d, J_{CP} 3.7, C), 129.2 (CH), 128.4 (CH), 127.2 (CH), 62.6 (d, J_{CP} 5.9, CH_2), 32.8 (d, J_{CP} 22.7, CH_2), 16.5 (d, J_{CP} 5.9, CH_3), one quaternary carbon signal was not observed; δ_P (162 MHz; $CDCl_3$) 12.2 (m).

OXIDATION OF HYDRAZONES 6

Potassium *N*-iodo *p*-toluenesulfonamide 4 (TsNIK)

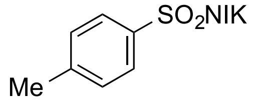

Prepared by a modification of a procedure previously described.[14] A solution of *p*-toluenesulfonamide (4.55 g, 26.6 mmol) in aqueous potassium hydroxide (10%; 11.5 mL) was added to a solution of potassium iodide (18.0 g, 108 mmol) and iodine (9.00 g, 35.5 mmol) in

water (20 mL). Aqueous potassium hydroxide (50%; 6 mL) was added, upon which loss of the colouration due to iodine occurred and a yellow precipitate appeared. The yellow solid was filtered, dried under suction and washed with ether (20 mL) to give the *title compound* as a yellow solid (6.57 g, 74%); mp 220 °C (decomp.) (lit.,[14]no mp reported); (Found: C, 24.89; H, 2.01; N, 3.95. $C_7H_7INO_2S$ requires C, 25.08; H, 2.10; N, 4.18%); ν_{max} (ATR)/cm^{-1} 1191, 1065, 959, 664, 625; δ_H (400 MHz; DMSO-*d*$_6$) 7.50 (2 H, d, *J* 8.0, ArH), 7.15 (2 H, d, *J* 8.0, ArH), 2.31 (3 H, s, CH_3); δ_C (100 MHz; DMSO-*d*$_6$) 144.0 (C), 138.1 (C), 128.0 (CH), 126.6 (CH), 20.8 (CH_3). The productshowed no signs of decomposition when stored in the dark at room temperature over several weeks but decomposes with iodine release when heated above 220 °C.

Alternatively, compound **4** was obtained from potassium iodide using bleach solution to generate iodine using the following procedure: A suspension of potassium iodide (6.70 g, 40 mmol) in concentrated hydrochloric acid (3 mL) was prepared at 0 °C. Bleach solution (8.4% available chlorine by titration, 30 mL) was then slowly added. The resulting mixture was stirred 5 min at room temperature and added to a solution of *p*-toluenesulfonamide (3.43 g, 20 mmol) dissolved in a mixture of concentrated potassium hydroxide solution (50%; 5 mL) and distilled water (5 mL) at 0 °C. After addition, the mixture was stirred for 5 min at 0 °C and the pale yellow precipitate was filtered off and dried to give the *title compound* as a pale yellow solid (2.98 g, 44%).

TsNIK oxidation of hydrazone to the corresponding diazo compound: Procedure A

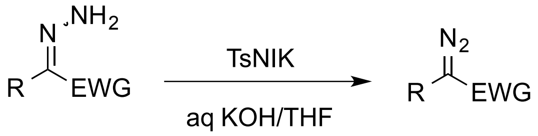

A suspension of potassium *N*-iodo *p*-toluenesulfonamide (369 mg, 1.1 mmol) in a solution of hydrazone in THF (1 mmol in 4 mL) was prepared. For the hydrazones that were solid at room temperature, THF was added to a mixture of the hydrazone and potassium *N*-iodo *p*-toluenesulfonamide. Aqueous potassium hydroxide (1 M) was slowly added to the THF suspension (so that the final volume ratio KOH (1 M) : THF was equal to 1:4). This caused dissolution of the potassium salt in the mixture and the appearance of a yellow to red colouration. In all cases the reaction was complete after stirring for 1 h at room temperature. The mixture was poured into aqueous potassium hydroxide (1 M; 5 mL) and extracted with ether (30 mL). The ethereal phase was washed with aqueous potassium hydroxide (1 M; 5 mL), saturated brine (5 mL) and dried over $MgSO_4$. Removal of the solvent under reduced pressure gave diazo compounds which were >95% pure as judged by ^{1}HNMR spectroscopy.

One-pot process hydrazone formation and oxidation, from α-ketoester to the corresponding diazo compound: Procedure B

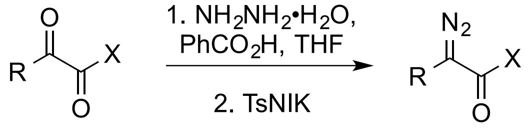

The starting α-ketoester (1.0 mmol) and benzoic acid (123 mg, 1.0 mmol) were dissolved in THF (4 mL) and hydrazine hydrate (49 µL, 1.0 mmol) was added. The mixture was stirred at room temperature for 16 h or until disappearance of the colourless hydrazine salt precipitate and completion of the reaction as judged by TLC. Aqueous potassium hydroxide (1 M; 1 mL) was then added at room temperature, followed by slow addition of potassium *N*-iodo *p*-toluenesulfonamide (402 mg, 1.2 mmol). The reaction progress was monitored by TLC. When required, additional quantities of TsNIK were added to the mixture (in 0.1 mmol portion). After completion, the reaction mixture was poured into aqueous potassium hydroxide (1 M; 5 mL) and extracted with ether (30 mL). The ethereal phase was washed with aqueous

potassium hydroxide (1 M; 5 mL), saturated brine (5 mL) and dried over $MgSO_4$. Removal of the solvent under reduced pressure gave diazo compounds that were over 95% pure as judged by ^{1}HNMR spectroscopy.

The following diazo compounds were prepared.

Diphenyldiazomethane 2

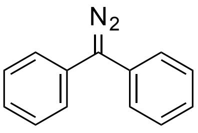

Obtained from **1** (196 mg, 1.0 mmol) by Procedure A (193 mg, 94%, purity 95% as judged by ^{1}HNMR). Purple solid; Rf 0.8 (ethyl acetate : light petroleum, 1 : 9); mp 29-30 °C (lit.,[15] mp 30 °C); ν_{max} ($CHCl_3$)/cm^{-1} 3063, 3011, 2959, 2045 (CN_2), 1595, 1496, 651; δ_H (400 MHz; $CDCl_3$) 7.40 (2 H, m, ArH), 7.31 (2 H, d, *J* 7.5, ArH), 7.19 (1 H, t, *J* 7.5, ArH); δ_C (100 MHz; CD_3OD) 130.7 (C), 130.3 (CH), 126.8 (CH), 126.2 (CH), the signal due to $\underline{C}N_2$ was not observed. The data are consistent with the literature.[15-17]

2-Diazo-1,2-diphenylethanone 10

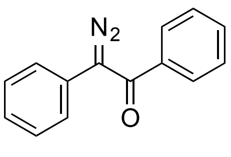

Obtained from **9** (224 mg, 1.0 mmol) by Procedure A (222 mg, 100%). Yellow solid; Rf 0.8 (ethyl acetate : light petroleum, 1 : 9); mp 73-74 °C (from light petroleum) (lit.,[17] mp 79 °C; lit.,[18] mp 66-67 °C), ν_{max} ($CHCl_3$)/cm^{-1} 3011, 2078 (CN_2), 1622, 1352, 1283, 850, 646; δ_H (400 MHz; $CDCl_3$) 7.59 - 7.65 (2 H, m, ArH), 7.36 - 7.53 (7 H, m, ArH), 7.26 (1 H, t, *J* 77, ArH); δ_C (100 MHz; acetone-d_6) 188.5 (C), 139.3 (C), 132.4 (CH), 129.7 (CH), 129.4 (CH),

128.4 (CH), 127.5 (CH), 126.6 (CH), the signals due to <u>C</u>$N_2$and one other quaternary carbon were not observed. The data are consistent with the literature.[17,18]

Ethyl 2-diazo-2-phenylacetate 11a

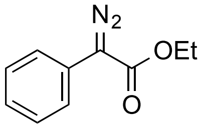

Obtained from **6a** (97 mg, 0.5 mmol) by Procedure A(92 mg, 95%) and from **5a**(100µL, 0.62 mmol) by Procedure B (116 mg, 97%). Orange oil; Rf 0.7 (ethyl acetate : light petroleum, 1 : 9); ν_{max} ($CHCl_3$)/cm^{-1} 2985, 2090 (CN_2), 1698, 1248, 1174;δ_H (400 MHz; $CDCl_3$) 7.49 (2H, d, *J* 7.3, ArH), 7.41-7.35 (2H, m, ArH), 7.18 (1 H, td, *J* 1.3, 7.4, ArH), 4.34 (2 H, q, *J* 7.2, C<u>H</u>$_2$$CH_3$), 1.34 (3 H, t, *J* 7.2, CH_2C<u>H</u>$_3$); δ_C (100 MHz; $CDCl_3$) 165.4 (C), 129.1 (CH), 125.9 (CH), 125.8 (C), 124.1 (CH), 61.1 (CH_2), 14.6 (CH_3), the signal due to <u>C</u>N_2 was not observed. The data are consistent with the literature.[19]

Ethyl 2-diazo-2-(4-methoxyphenyl)acetate 11b

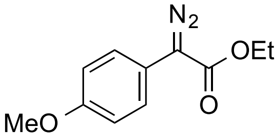

Obtained from **6b** (105 mg, 0.47 mmol) by Procedure A (97 mg, 93%)and from **5b** (103 mg, 0.5 mmol) by Procedure B(95 mg, 87%). Red solid; Rf 0.7 (ethyl acetate : light petroleum, 1 : 9); mp 43 °C (lit.,[20]no mp reported); ν_{max} ($CHCl_3$)/cm^{-1} 3009, 2985, 2087 (CN_2), 1695, 1514, 1257, 1173;δ_H (400 MHz; $CDCl_3$) 7.97 (2 H, d, *J* 9.0, ArH), 6.95 (2 H, d, *J* 9.0, ArH), 4.41 (2 H, q, *J* 7.2, C<u>H</u>$_2$$CH_3$), 3.86 (3 H, s, OMe), 1.39 (3 H, t, *J* 7.2, CH_2C<u>H</u>$_3$); δ_C (100 MHz; $CDCl_3$) 165.9 (C), 158.2 (C), 126.1 (CH), 117.2 (C), 114.7 (CH), 61.1 (CH_2), 55.5 (CH_3), 14.7 (CH_3), signal due to <u>C</u>N_2 not observed. The data are consistent with the literature.[20]

Ethyl 2-diazo-2-(2-methoxyphenyl)acetate 11c

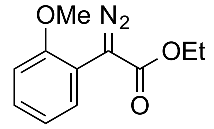

Obtained from **6c** (140 mg, 0.63 mmol) by Procedure A (123 mg, 89%). Yellow oil; Rf 0.6 (ethyl acetate : light petroleum, 1 : 9); ν_{max} ($CHCl_3$)/cm^{-1} 3011, 2984, 2102 (CN_2), 1689, 1498, 1255, 1028;δ_H (400 MHz; $CDCl_3$) 7.56 (1 H, dd, *J* 7.8, 1.5, ArH), 7.23 - 7.28 (1 H, m, ArH), 7.02 (1 H, td, *J* 7.6, 1.1, ArH), 6.90 (1 H, dd, *J* 8.3, 1.0, ArH), 4.30 (2 H, q, *J* 7.2, $C\underline{H}_2CH_3$), 3.86 (3 H, s, OMe), 1.32 (3 H, t, *J* 7.2, $CH_2C\underline{H}_3$); δ_C (100 MHz; $CDCl_3$) 166.4 (C), 155.6 (C), 130.3 (CH), 128.7 (CH), 121.3 (CH), 114.0 (C), 111.0 (CH), 60.8 (CH_2), 55.7 (CH_3), 14.7 (CH_3), the signal due to $\underline{C}N_2$ was not observed.

Ethyl 2-(4-bromophenyl)-2-diazoacetate 11d

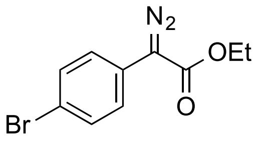

Obtained from **5d** (257 mg, 1.0 mmol) by Procedure B (255 mg, 95%).Orange solid; Rf 0.8 (ethyl acetate : light petroleum, 1 : 4); mp 48-49 °C (lit.,[21]mp 54 °C); ν_{max} ($CHCl_3$)/cm^{-1} 2091 (CN_2), 1699;δ_H (400 MHz; $CDCl_3$) 7.46 - 7.52 (2 H, m, ArH), 7.33 - 7.39 (2 H, m, ArH), 4.33 (2 H, q, *J* 7.1, $C\underline{H}_2CH_3$), 1.34 (3 H, t, *J* 7.1, $CH_2C\underline{H}_3$); δ_C (100 MHz; $CDCl_3$) 165.0 (C), 132.1 (CH), 125.5 (CH), 125.0 (C), 119.4 (C), 61.3 (CH_2), 14.6 (CH_3). The signal for $\underline{C}N_2$ was not observed.The data are consistent with the literature.[21]

Ethyl 2-diazo-3-methylbutanoate 11e

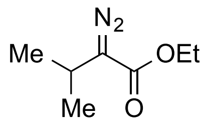

Obtained from **6e** (144 mg, 0.91 mmol) by Procedure A (124 mg, 87%) and from **5e** (113 mg, 0.8 mmol) by Procedure B (107 mg, 87%). Yellow oil; Rf 0.8 (ethyl acetate : light petroleum, 1 : 9); ν_{max} ($CHCl_3$)/cm^{-1} 2979, 2084 (CN_2), 1683, 1390, 1270, 1092; δ_H (400 MHz; $CDCl_3$) 4.21 (2 H, q, *J* 7.2, C$\underline{H}_2$$CH_3$), 2.75 (1 H, spt, *J* 6.9, CH), 1.27 (3 H, t, *J* 7.2, CH_2C$\underline{H}_3$), 1.14 (6 H, d, *J* 6.9, CH_3); δ_C (100 MHz; $CDCl_3$) 60.7 (CH_2), 23.3 (CH), 20.7 (CH_3), 14.7 (CH_3), the signals due to $\underline{C}$O and $\underline{C}N_2$ were not observed. The data are consistent with the literature.[4]

Ethyl 2-diazopropanoate 11f

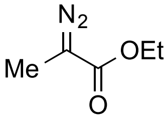

Obtained from **6f** (80 mg, 0.6 mmol) by Procedure A (53 mg, 67%) and from **5f** (96 µL, 0.8 mmol) by Procedure B (106 mg, 96%). Yellow liquid (lit.,[22] bp 50 °C/20 mmHg); Rf 0.7 (ethyl acetate : light petroleum, 1 : 9); ν_{max} ($CHCl_3$)/cm^{-1} 2985, 2086 (CN_2), 1682, 1328, 1310, 1140; δ_H (400 MHz; $CDCl_3$) 4.22 (q, *J* 7.1, 2 H, C$\underline{H}_2$$CH_3$), 1.96 (s, 3 H, Me), 1.27 (t, *J* 7.2, 3 H, CH_2C$\underline{H}_3$); δ_C (100 MHz; $CDCl_3$) 60.9 (CH_2), 14.7 (CH_3), 8.6 (CH_3), the signals due to $\underline{C}$O and $\underline{C}N_2$ were not observed. The data are consistent with the literature.[23]

Ethyl 2-diazo-4-phenylbutanoate 11g

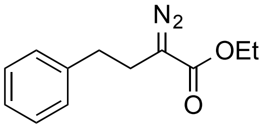

Obtained from **6g** (112 mg, 0.5 mmol) by Procedure A (101 mg, 91%) and from **5g** (95 µL, 0.5 mmol) by Procedure B (92 mg, 84%). Yellow oil; Rf 0.8 (ethyl acetate : light petroleum, 1 : 9); ν_{max} ($CHCl_3$)/cm^{-1} 3008, 2985, 2087 (CN_2), 1682, 1373, 1315, 1173, 1115; δ_H (400 MHz; $CDCl_3$) 7.27 - 7.34 (2 H, m, ArH), 7.17 - 7.25 (3 H, m, ArH), 4.22 (2 H, q, *J* 7.2, C$\underline{H}_2$$CH_3$), 2.84 (2 H, t, *J* 7.8, CH_2), 2.61 (2 H, t, *J* 7.8, CH_2), 1.27 (3 H, t, *J* 7.2, CH_2C$\underline{H}_3$); δ_C

(100 MHz; $CDCl_3$) 140.2 (C), 128.6 (CH), 128.5 (CH), 126.4 (CH), 60.9 (CH_2), 34.0 (CH_2), 25.4 (CH_2), 14.5 (CH_3), the signals due to $\underline{C}O$ and $\underline{C}N_2$ were not observed. The data are consistent with the literature.[4]

Allyl 2-diazo-2-phenylacetate 11h

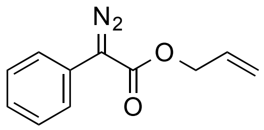

Obtained from **6h** (101 mg, 0.5 mmol) by Procedure A (95 mg, 95%) and from **5h** (96 mg, 0.5 mmol) by Procedure B (91 mg, 88%). Orange oil; Rf 0.6 (ethyl acetate : light petroleum, 1 : 9); ν_{max} ($CHCl_3$)/cm^{-1}; 2951, 2091 (CN_2), 1699, 1247, 1156; δ_H (400 MHz; $CDCl_3$) 7.46 - 7.53 (2 H, m, ArH), 7.35 - 7.43 (2 H, m, ArH), 7.19 (1 H, tt, *J* 7.3, 1.3, ArH), 5.99 (1 H, ddt, *J* 17.2, 10.5, 5.6, vinylic CH), 5.37 (1 H, dq, *J* 17.2, 1.4, vinylic CH), 5.28 (1 H, dq, *J* 10.5, 1.4, vinylic CH), 4.78 (2 H, dt, *J* 5.6, 1.4, CH_2); δ_C (100 MHz; $CDCl_3$) 164.9 (C), 132.2 (CH), 129.1 (CH), 126.0 (CH), 125.6 (C), 124.1 (CH), 118.5 (CH_2), 65.6 (CH_2), the signal due to $\underline{C}N_2$ was not observed. The data are consistent with the literature.[4]

Cyclohexyl 2-diazo-2-phenylacetate 11i

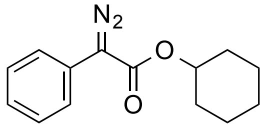

Obtained from **6i** (113 mg, 0.46 mmol) by Procedure A (105 mg, 93%) and from **5i** (116 mg, 0.5 mmol) by Procedure B (121 mg, 99%). Orange oil; Rf 0.6 (ethyl acetate : light petroleum, 1 : 9); (Found: $M+Na^+$, 267.1104. $C_{14}H_{16}N_2NaO_2$ requires 267.1104); ν_{max} ($CHCl_3$)/cm^{-1} 2941, 2090 (CN_2), 1693, 1246, 1169;δ_H (400 MHz; $CDCl_3$) 7.47 - 7.52 (2 H, m, ArH), 7.35 - 7.41 (2H, m, ArH), 7.17 (1 H, t, *J* 7.3, ArH), 4.94 - 5.03 (1 H, m, $OC\underline{H}$), 1.86 - 1.97 (2 H, m, CH_2), 1.74 (2 H, m, CH_2), 1.24 - 1.61 (6 H, m, CH_2); δ_C (100 MHz; $CDCl_3$) 164.8 (C), 128.9

(CH), 125.9 (C), 125.7 (CH), 124.0 (CH), 73.3 (CH), 31.8 (CH_2), 25.4 (CH_2), 23.6 (CH), the signal due to $\underline{C}N_2$ was not observed. The data are consistent with the literature.[4]

3-Diazo-4,4-dimethyldihydrofuran-2(*3H*)-one 11j

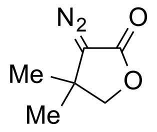

Obtained from **5j** (103 mg, 0.8 mmol) by Procedure B (76 mg, 68%). Yellow oil; Rf 0.5 (ethyl acetate : light petroleum, 1 : 4); ν_{max} ($CHCl_3$)/cm^{-1} 2970, 2099 (CN_2), 1732, 1377, 1146, 1058, 1015; δ_H (300 MHz; $CDCl_3$) 4.05 (2 H, s, CH_2), 1.41 (6 H, s, Me); δ_C (75 MHz; $CDCl_3$) 169.3 (C), 78.7 (CH_2), 39.0 (C), 26.0 (CH_3), the signal due to $\underline{C}N_2$ was not observed. The data are consistent with the literature.[24]

3-Diazoindolin-2-one 11k

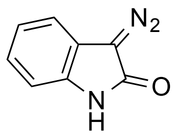

Obtained from **5k** (119 mg, 0.8 mmol) by Procedure B (120 mg, 94%) with modified work-up conditions: ethyl acetate was used instead of ether for the extraction step. The product was purified by column chromatography (30 % ethyl acetate in light petroleum). Red solid; Rf 0.3 (ethyl acetate : light petroleum, 1 : 1); mp 161 °C (decomp) (from ethyl acetate/cyclohexane) (lit.,[25] mp 168 °C (decomp)); (Found: M+Na$^+$, 182.0331. $C_8H_5N_3NaO$ requires 182.0330); ν_{max} ($CHCl_3$)/cm^{-1} 3449, 2118, 2099 (CN_2), 1693, 1468, 1402, 1191; δ_H (400 MHz; CD_3OD) 7.27-7.30 (1 H, m, ArH), 7.13 (1 H, td, *J* 7.6, 1.2, ArH), 7.06 (1 H, dd, *J* 7.6, 1.0, ArH), 6.95 - 6.98 (1 H, m, ArH); δ_C (100 MHz; CD_3OD) 169.4 (C), 132.3 (C), 125.1 (CH), 121.7 (CH), 118.3 (CH), 117.2 (C), 110.1 (CH). The signal due to $\underline{C}N_2$ was not observed. The data are consistent with the literature.[25]

3-Diazo-1-methylindolin-2-one 11l

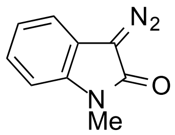

Obtained from **5l** (161 mg, 1.0 mmol) by Procedure B (154 mg, 89%). Red solid; Rf 0.4 (ethyl acetate : light petroleum, 1 : 1); mp 84-85°C (lit.,[26] mp 87-89 °C);(Found: M+Na$^+$, 196.0486. $C_9H_7N_3NaO$requires 196.0481); ν_{max} ($CHCl_3$)/cm^{-1} 3011, 2105 (CN_2), 2095, 1678; δ_H (400 MHz; $CDCl_3$) 7.16 - 7.23 (2 H, m, ArH), 7.08 (1 H, tq, *J* 7.6, 0.9, ArH), 6.91 (1 H, d, *J* 7.6, ArH), 3.32 (3 H, s, Me); δ_C (100 MHz; $CDCl_3$) 166.9 (C), 134.6 (C), 125.6 (CH), 122.2 (CH), 118.3 (CH), 116.8 (C), 108.7 (CH), 26.9 (Me). The signal for $\underline{C}N_2$ was not observed.

Ethyl 2-diazo-2-(2-thienyl)acetate 11m

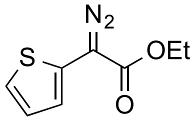

Obtained from **6m** (mixture of (*E*)- and (*Z*)-hydrazones100 mg, 0.5 mmol) by Procedure A (93 mg, 95%). Deep red oil; Rf 0.8 (ethyl acetate : light petroleum, 1 : 1); (Found: M+Na$^+$, 219.0202. $C_8H_8N_2NaO_2S$ requires 219.0199); ν_{max} ($CHCl_3$)/cm^{-1} 2087 (CN_2), 1695, 1289; δ_H (400 MHz; $CDCl_3$) 7.31 (1 H, dd, *J* 5.1, 1.2, ArH), 7.03 (1 H, dd, *J* 5.1, 3.7, ArH), 6.82 (1 H, dd, *J* 3.7, 1.2, ArH), 4.35 (2 H, q, *J* 7.2, $C\underline{H_2}CH_3$), 1.34 (3 H, t, *J* 7.1, $CH_2C\underline{H_3}$); δ_C (100 MHz; $CDCl_3$) 165.2 (C), 127.0 (CH), 126.0 (C), 125.7 (CH), 121.1 (CH), 61.7 (CH_2), 14.6 (CH_3), the signal due to $\underline{C}N_2$ was not observed.

Ethyl [1,2,3]triazolo[1,5-*a*]pyridine-3-carboxylate 11n

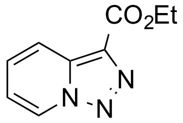

Obtained from **5n** (89 mg, 0.5 mmol) by Procedure B (80 mg, 84%) with modified work-up conditions: ethyl acetate was used instead of ether for the extraction step. The product was purified by column chromatography using 40% ethyl acetate in light petroleum. Colourless solid; Rf 0.2 (ethyl acetate : light petroleum, 1 : 1); mp 105-106 °C; (Found: M+Na$^+$, 214.0589. $C_9H_9N_3NaO_2$ requires 214.0592); ν_{max} ($CHCl_3$)/cm^{-1} 1724, 1707, 1545, 1270, 1069; δ_H (400 MHz; $CDCl_3$) 8.79 - 8.86 (1 H, m, ArH), 8.28 (1 H, dd, *J* 8.8, 1.1, ArH), 7.54 (1 H, ddd, *J* 8.8, 6.8, 0.9, ArH), 7.15 (1 H, td, *J* 6.8, 1.1, ArH), 4.52 (2 H, q, *J* 7.2, $C\underline{H}_2CH_3$), 1.48 (3 H, t, *J* 7.2, $CH_2C\underline{H}_3$); δ_C (100 MHz; $CDCl_3$) 161.5 (C), 135.2 (C), 129.7 (C), 129.2 (CH), 126.0 (CH), 119.5 (CH), 116.5 (CH), 60.5 (CH_2), 14.6 (CH_3). The data are consistent with the literature.[27]

***tert*-Butyl 3-(1-diazo-2-methoxy-2-oxoethyl)indole-1-carboxylate 11o**

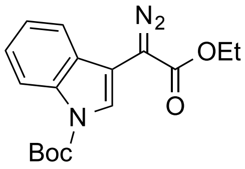

Obtained from **6o** (158 mg, 0.5 mmol) by Procedure A (130 mg, 79%). Orange solid; Rf 0.8 (ethyl acetate : light petroleum, 1 : 1); mp 84-85 °C (decomp) (lit.,[28] mp 86-87 °C); (Found: M+Na$^+$, 338.1110. $C_{16}H_{17}N_3NaO_4$ requires 338.1111); ν_{max} ($CDCl_3$)/cm^{-1} 2089 (CN_2), 1732, 1703, 1372, 1246, 1155; δ_H (400 MHz; $CDCl_3$) 8.22 (1 H, d, *J* 8.2, ArH), 7.87 (1 H, s, ArH), 7.49 (1 H, d, *J* 7.9, ArH), 7.32 - 7.41 (1 H, m, ArH), 7.22 - 7.30 (1 H, m, ArH), 3.89 (3 H, s, OMe), 1.68 (9 H, s, *t*-Bu); δ_C (100 MHz; $CDCl_3$) 149.4 (C), 135.4 (C), 127.6 (C), 125.1 (CH), 124.0 (CH), 123.1 (CH), 118.5 (CH), 115.8 (CH), 103.1 (C), 84.3 (C), 52.4 (CH_3), 28.3 (CH_3), the signals due to $\underline{C}N_2$ and one other quaternary carbon were not observed. The data are consistent with the literature.[28]

1-Allyl-4-phenyl-*1H*-1,2,3-triazol-5-ol 11p

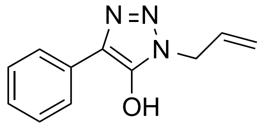

Obtained from **5p** (95 mg, 0.5 mmol) by Procedure B(85 mg, 85%), using the following work-up conditions: after completion of the reaction, the mixture was poured in aqueous potassium hydroxide (1 M; 5 mL) and agitated vigorously. An aqueous solution of acetic acid (10%) was then slowly added until pH = 5. The mixture was extracted with ethyl acetate (4 × 20 mL). The combined organic phases were washed with saturated brine (15 mL) and dried on $MgSO_4$. Removal of the solvent under reduced pressure gave a solid residue that was purified by column chromatography (elution gradient methanol in dichloromethane, 5% to 10%) to give 1-allyl-4-phenyl-*1H*-1,2,3-triazol-5-ol as a colourless solid; Rf 0.3 (methanol : dichloromethane, 1 : 9); mp162-164 °C (decomp); (Found: M+Na^+, 224.0772. $C_{11}H_{11}N_3NaO$ requires 224.0794); ν_{max} (ATR)/cm^{-1} 1613, 1568, 814, 767; δ_H (400 MHz; DMSO-d_6) 7.88 (2 H, d, *J* 7.6, ArH), 7.40 (2 H, t, *J* 7.6, ArH), 7.23 (1 H, t, *J* 7.6, ArH), 6.00 (1 H, ddt, *J* 17.0, 10.5, 5.6, vinylic CH), 5.22 (1 H, dd, *J* 10.5, 1.5, vinylic CH), 5.10 (1 H, dd, *J* 17.0, 1.5, vinylic CH), 4.80 (2 H, d, *J* 5.6, CH_2); δ_C (100 MHz; DMSO-d_6) 133.1 (CH), 131.6 (C), 129.0 (CH), 126.0 (CH), 124.5 (CH), 118.0 (CH_2), 47.7 (CH_2), two quaternary carbon signals were not observed.

1-(2,5-Dimethoxyphenyl)-4-phenyl-1H-1,2,3-triazol-5-ol 11q

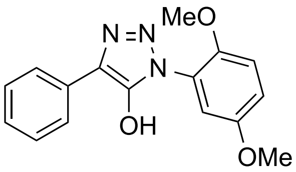

Obtained from **5q** (286 mg, 1.0 mmol) Procedure B(271 mg, 91%), using the following work-up conditions: after completion of the reaction, the mixture was poured in aqueous potassium hydroxide (1 M; 5 mL) and agitated vigorously. An aqueous solution of aqueous acetic acid

(10%) was then slowly added until pH = 5, followed by addition of a saturated solution of sodium thiosulfate (2 mL). The precipitate formed was filtrated, washed with dichloromethane (20 mL) and dried to give the *title compound* as an off-white solid; Rf 0.3 (methanol : dichloromethane, 1 : 9); mp 173-174°C (decomp); (Found: M+Na$^+$, 320.1009. $C_{16}H_{15}N_3NaO_3$ requires 320.1006); ν_{max} (ATR)/cm^{-1} 1605, 1507, 1227, 774, 649; δ_H (400 MHz; $CDCl_3$) 11.65 (1 H, br s, OH), 7.90 (2 H, d, *J* 7.5, ArH), 7.44 (2 H, t, *J* 7.5, ArH), 7.27 (1 H, t, *J* 7.5, ArH), 7.22 (1 H, d, *J* 9.1, ArH), 7.15 (1 H, dd, *J* 9.1,2.8, ArH), 7.07 (1 H, d, *J* 2.8, ArH), 3.77 (3 H, s, OMe), 3.74 (3 H, s, OMe); δ_C (100 MHz; $CDCl_3$) 152.9 (C), 148.5 (C), 131.1 (C), 128.6 (CH), 126.5 (CH), 124.3 (CH), 123.6 (C), 116.7 (CH), 114.2 (CH), 113.8 (CH), 56.3 (CH_3), 55.8 (CH_3), two quaternary carbon signals were not observed.

1-(2-Furylmethyl)-4-phenyl-1H-1,2,3-triazol-5-ol 11r

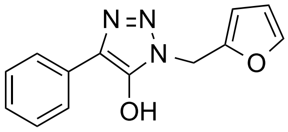

Obtained from **5r** (229 mg, 1.0 mmol) Procedure B(181 mg, 75%), using the following work-up conditions: after completion of the reaction, the mixture was poured in aqueous potassium hydroxide (1 M; 5 mL) and agitated vigorously. An aqueous solution of aqueous acetic acid (10%) was then slowly added until pH = 5, followed by addition of a saturated solution of sodium thiosulfate (2 mL). The precipitate formed was filtrated and washed with ether (20 mL). The resulting solid was further purified by column chromatography using the elution gradient: methanol in dichloromethane, 5% to 10% to give the *title compound* as a colourless solid; Rf 0.3 (methanol : dichloromethane, 1 : 9); mp 194-195 °C (decomp); (Found: M+Na$^+$, 264.0740. $C_{13}H_{11}N_3NaO_2$ requires 264.0743); ν_{max} (ATR)/cm^{-1} 1604, 1575, 762, 649; δ_H (400 MHz; DMSO-d_6) 11.78 (1 H, br s, OH), 7.85 (2 H, d, *J* 7.4, ArH), 7.64 (1 H, dd, *J* 1.8, 0.8, furan H-5), 7.42 (2 H, t, *J* 7.4, ArH), 7.25 (1 H, t, *J* 7.4, ArH), 6.47 (1 H, d, *J* 3.1, furan H-3),

6.45 (1 H, dd, *J* 3.1, 1.8, furan H-4), 5.40 (2 H, s, CH_2); δ_C (100 MHz; DMSO-d_6) 148.7 (C), 143.3 (CH), 128.6 (CH), 126.6 (CH), 124.2 (CH), 110.8 (CH), 109.2 (CH), 41.7 (CH_2), three quaternary carbon signals were not observed.

Dimethyl 1-diazoethylphosphonate 12a

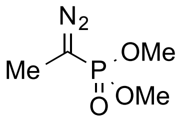

Obtainedfrom **7a** (104 mg, 1.0 mmol) byProcedure A (57 mg, 56%). Yellow liquid; Rf 0.2 (ethyl acetate : light petroleum, 1 : 1); ν_{max} ($CHCl_3$)/cm^{-1} 3009, 2954, 2852, 2087 (CN_2), 1459, 1253, 1030, 832; δ_H (400 MHz; CD_3OD) 3.76 (6 H, d, J_{PH}11.5, OMe), 1.85 (3 H, d, J_{PH} 9.8, CH_3); δ_C (100 MHz; CD_3OD) 53.6 (d, J_{CP}5.4, OMe), 8.6 (d, J_{CP}7.7, CH_3), the signal due to CN_2 was not observed; δ_P (162 MHz; CD_3OD) 26.6 (m). The data are consistent with the literature.[29,30]

Diethyl 1-diazo-2-phenylethylphosphonate 12b

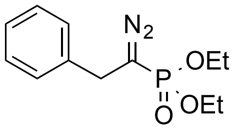

Obtainedfrom **7b** (135 mg, 0.5 mmol) byProcedure A (127 mg, 94%). Yellow liquid; Rf 0.2 (ethyl acetate : light petroleum, 1 : 1); ν_{max} ($CHCl_3$)/cm^{-1} 3001, 2082 (CN_2), 1248, 1025, 1050, 972; δ_H (400 MHz; $CDCl_3$) 7.30 - 7.36 (2 H, m, ArH), 7.22 - 7.29 (3 H, m, ArH), 3.96 - 4.17 (4 H, m, $C\underline{H}_2CH_3$), 3.44 (2 H, d, *J* 9.8, CH_2), 1.30 (6 H, td, *J* 7.0, 0.3, $CH_2C\underline{H}_3$); δ_C (100 MHz; $CDCl_3$) 137.5 (d, J_{CP} 2.9, C), 128.9 (CH), 128.5 (CH), 127.3 (CH), 62.6 (d, J_{CP} 5.9, CH_2), 30.3 (d, J_{CP} 8.8,CH_2), 16.3 (d, J_{CP} 6.6,CH_3); δ_P (162 MHz; $CDCl_3$) 21.0 (m), the signal due to $\underline{C}N_2$ was not observed.

2-Diazo-1-phenylethanone 13

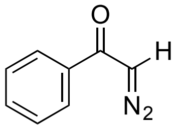

Obtainedfrom **8** (119 mg, 0.8 mmol) byProcedure A (110 mg, 94%). Orange solid; Rf 0.6 (ethyl acetate : light petroleum, 1 : 1); mp 43-44 °C (from ether) (lit.,[9] mp 49°C); (Found: 2M+Na$^+$, 315.0849. $C_{16}H_{12}N_4NaO_2$ requires 315.0852); ν_{max} ($CHCl_3$)/cm^{-1} 2111 (CN_2), 1624, 1364; δ_H (400 MHz; $CDCl_3$) 7.72 - 7.79 (2 H, m, ArH), 7.50 - 7.56 (1 H, m, ArH), 7.39 - 7.47 (2 H, m, ArH), 5.91 (1 H, s, CH); δ_C (100 MHz; $CDCl_3$) 186.4 (C), 136.7 (C), 132.8 (CH), 128.7 (CH), 126.8 (CH), 54.3 (CH). The data are consistent with the literature.[9]

References

[1] Q. Meng, Y. Sun, V. Ratovelomanana-Vidal, J. P. Genêt, Z. Zhang, *J. Org. Chem.* **2008**, *73*, 3842-3847.

[2] O. Ilovich, H. Billauer, S. Dotan, E. Mishani, *Bioorg. Med. Chem.* **2010**, *18*, 612-620.

[3] H. Wang, T.-S. Zhu, M.-H. Xu, *Org. Biomol. Chem.* **2012**, *10*, 9158-9164.

[4] H. E. Bartrum, D. C. Blakemore, C. J. Moody, C. J. Hayes, *Chem. Eur. J.* **2011**, *17*, 9586-9589.

[5] J. C. Barrow, C. Coburn, H. G. Selnick, P. L. Ngo, US20020193398A1, 2002.

[6] Y. Yamamoto, H. Ochi, T. Tanaka, *Chem. Pharm. Bull.* **1995**, *43*, 1028-1030.

[7] F. Heaney, J. Fenlon, P. McArdle, D. Cunningham, *Org. Biomol. Chem.* **2003**, *1*, 1122-1132.

[8] B. Murukan, B. S. Kumari, K. Mohanan, *J. Coord. Chem.* **2007**, *60*, 1607-1617.

[9] S. Hauptmann, H. Wilde, *J. Prakt. Chem.* **1969**, *311*, 604-613.

[10] H. S. Alonazy, H. M. A. Al-Hazimi, M. M. S. Korraa, *Arab. J. Chem.* **2009**, *2*, 101-108.

[11] E. Ciganek, *J. Org. Chem.* **1970**, *35*, 862-864.

[12] D. H. R. Barton, J. C. Jaszberenyi, W. Liu, T. Shinada, *Tetrahedron* **1996**, *52*, 14673-14688.

[13] N. Takamura, S. Yamada, *Chem. Pharm. Bull.* **1976**, *24*, 800-803.

[14] E. Roberts, *J. Chem. Soc.* **1923**, 849-853.

[15] R. Baltzly, N. B. Mehta, P. B. Russell, R. E. Brooks, E. M. Grivsky, A. M. Steinberg, *J. Org. Chem.* **1961**, *26*, 3669-3676.

[16] B. P. Giri, G. Prasad, K. N. Mehrotra, *Can. J. Chem.* **1979**, *57*, 1157-1161.

[17] C. D. Nenitzescu, E. Solomonica, *Org. Synth.* **1935**, *15*.

[18] E. A. Lund, I. A. Kennedy, A. G. Fallis, *Can. J. Chem.* **1996**, *74*, 2401-2412.

[19] Q.-H. Deng, H.-W. Xu, A. W.-H. Yuen, Z.-J. Xu, C.-M. Che, *Org. Lett.* **2008**, *10*, 1529-1532.

[20] C. Peng, G. Yan, Y. Wang, Y. Jiang, Y. Zhang, J. Wang, *Synthesis* **2010**, 4154-4168.

[21] N. D. Hahn, M. Nieger, K. H. Doetz, *J. Organomet. Chem.* **2004**, *689*, 2662-2673.

[22] D. Daniil, U. Merkle, H. Meier, *Synthesis* **1978**, 535-536.

[23] L. Huang, W. D. Wulff, *J. Am. Chem. Soc.* **2011**, *133*, 8892-8895.

[24] R. T. Buck, D. M. Coe, M. J. Drysdale, L. Ferris, D. Haigh, C. J. Moody, N. D. Pearson, J. B. Sanghera, *Tetrahedron: Asymmetry* **2003**, *14*, 791-816.

[25] C. Marti, E. M. Carreira, *J. Am. Chem. Soc.* **2005**, *127*, 11505-11515.

[26] M. Colonna, P. Bruni, G. Guerra, *Gazz. Chim. Ital.* **1969**, *99*, 3-13.

[27] B. Abarca, D. J. Hayles, G. Jones, D. R. Sliskovic, *J. Chem. Res., Synop.* **1983**, 144.

[28] H. M. L. Davies, R. J. Townsend, *J. Org. Chem.* **2001**, *66*, 6595-6603.

[29] D. Seyferth, P. Hilbert, R. S. Marmor, *J. Am. Chem. Soc.* **1967**, *89*, 4811-4812.

[30] D. Seyferth, R. S. Marmor, *J. Org. Chem.* **1971**, *36*, 128-136.

Potassium *N*-iodo *p*-toluenesulfonamide4 (TsNIK)

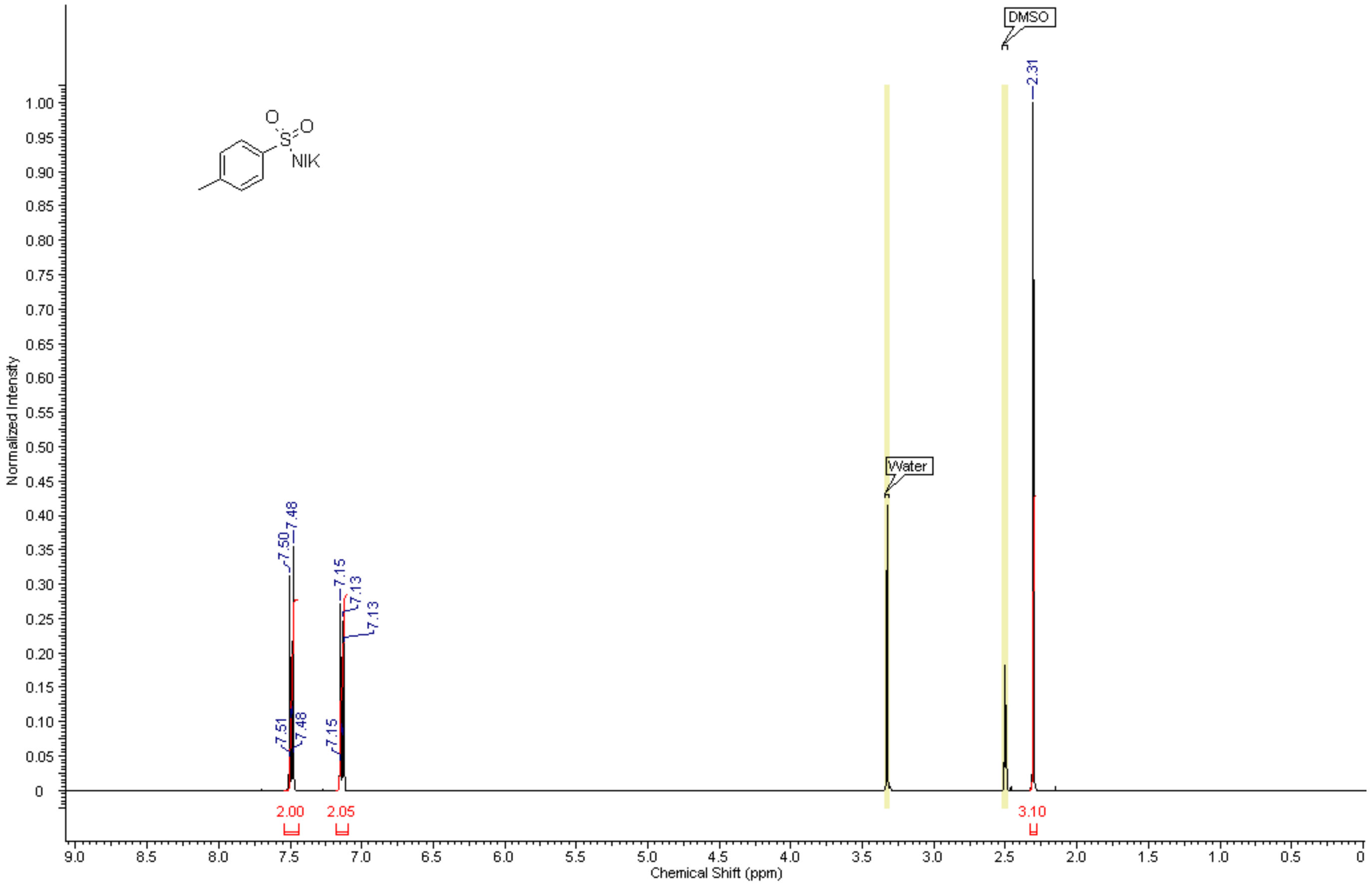

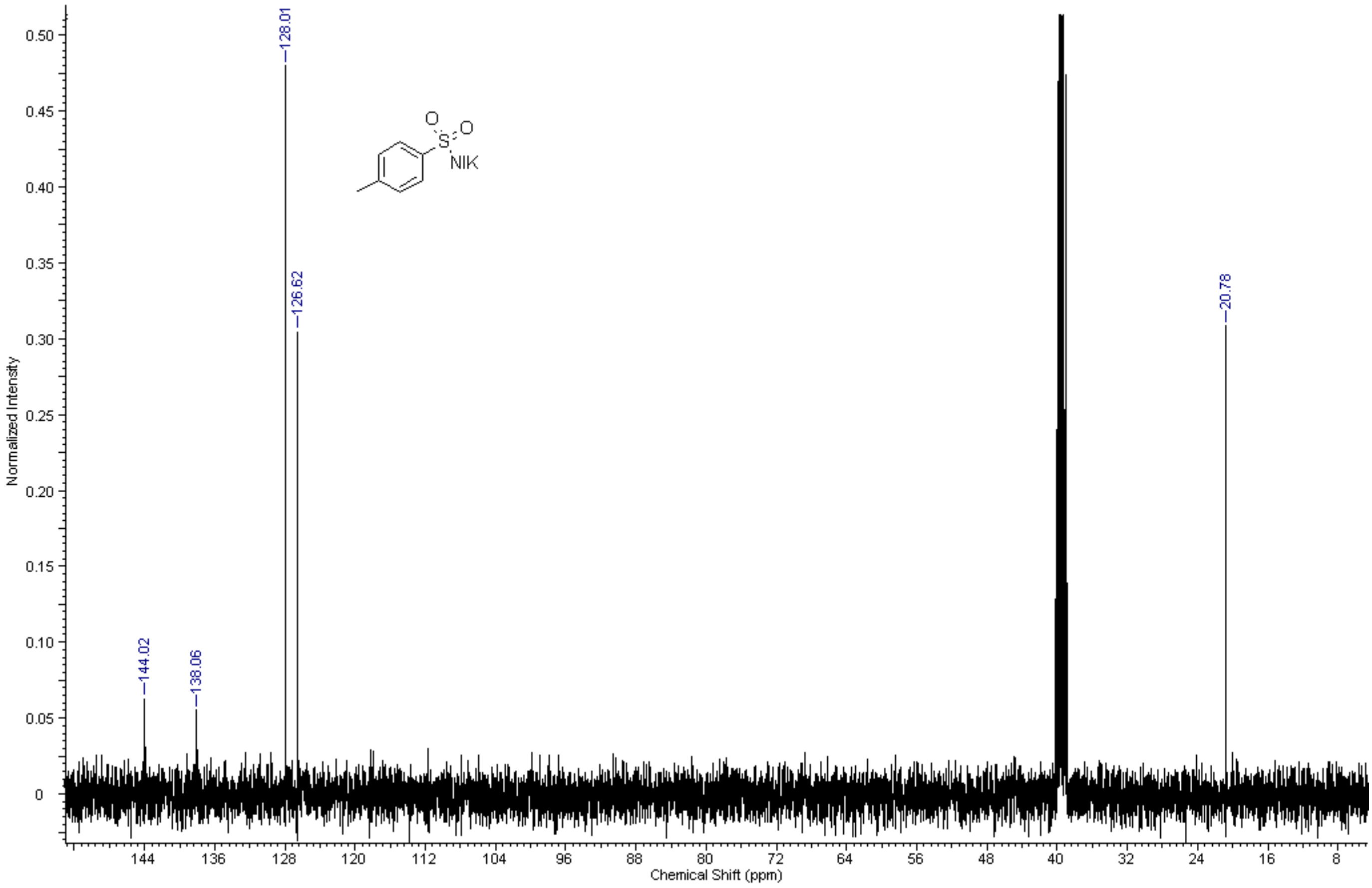

Allyl 2-oxo-2-phenylacetate 5h

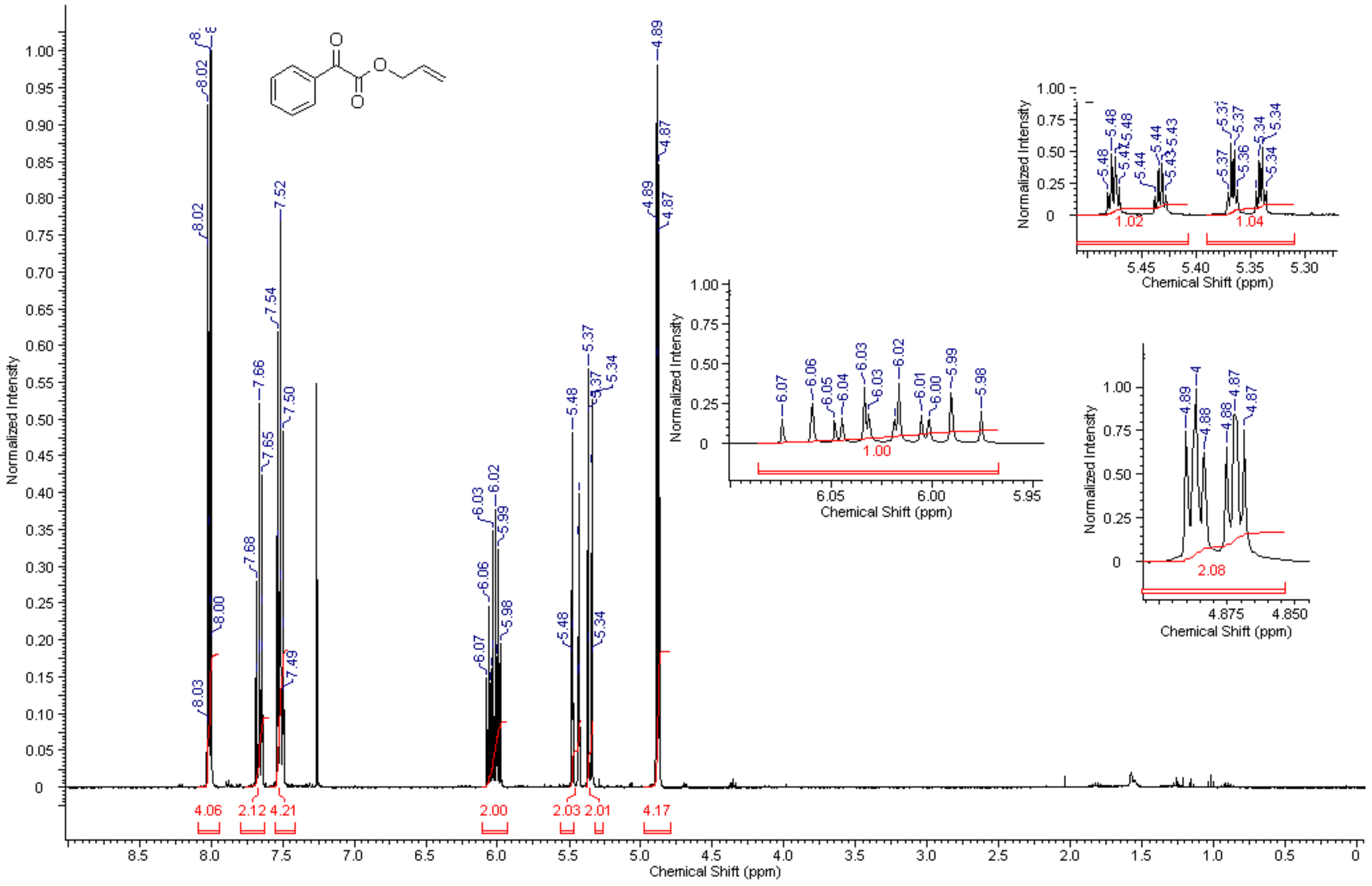

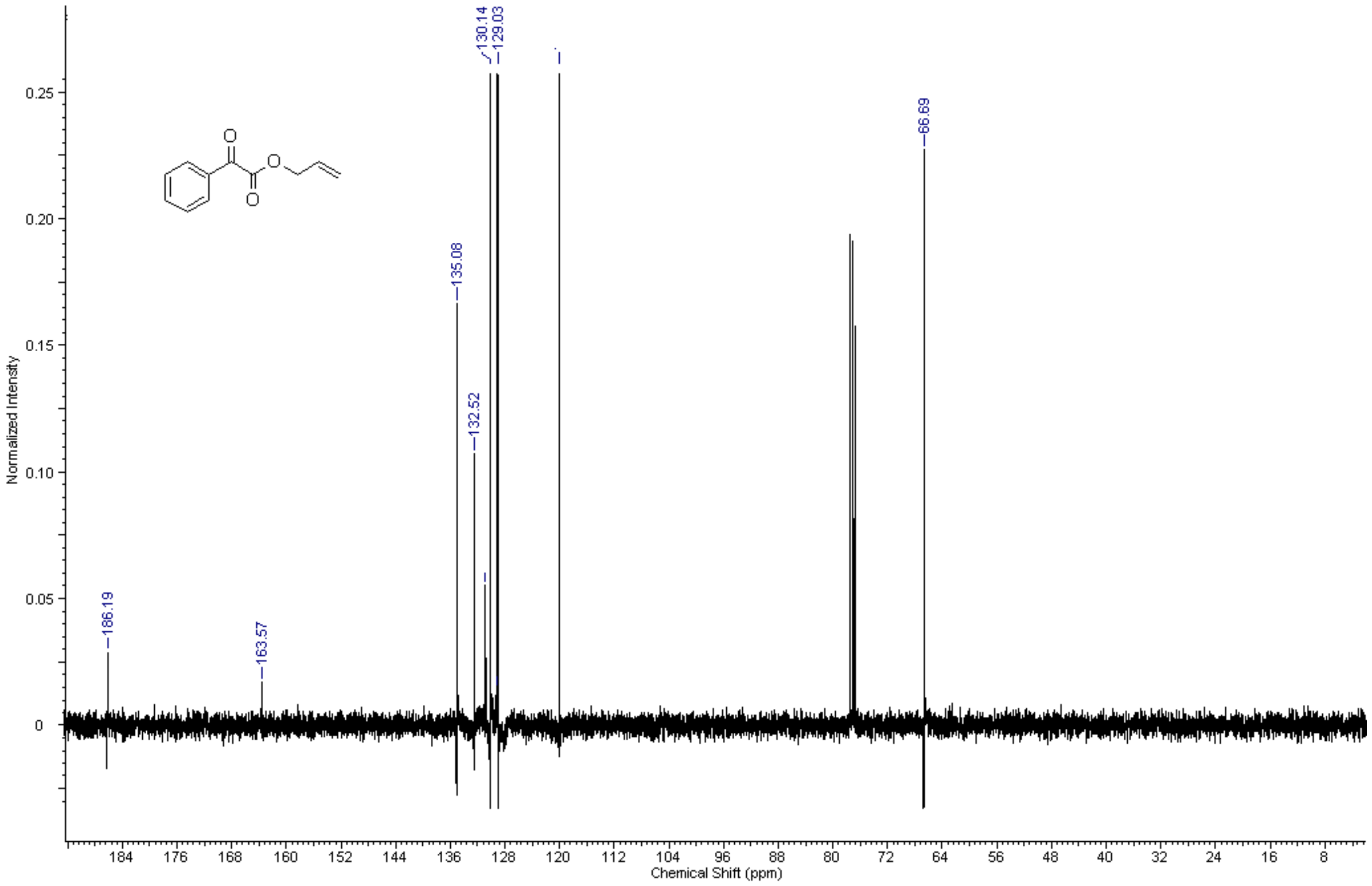

Cyclohexyl 2-oxo-2-phenylacetate 5i

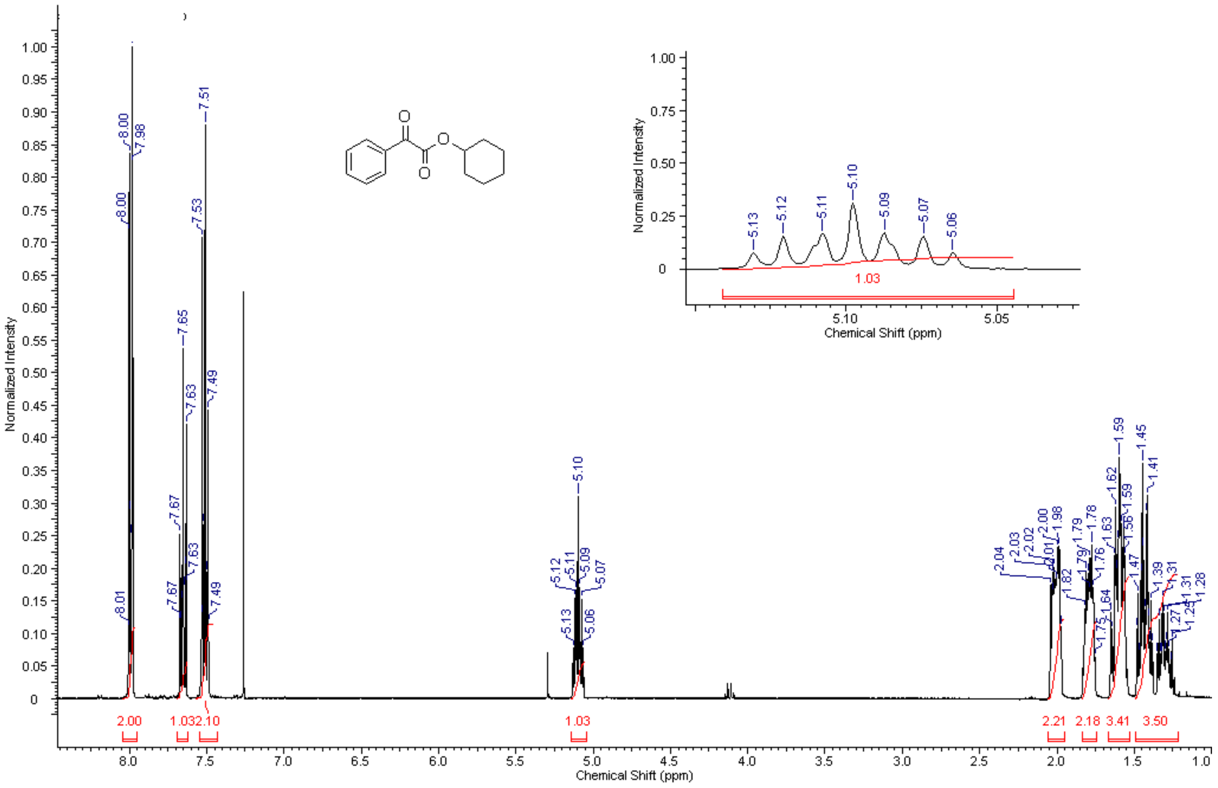

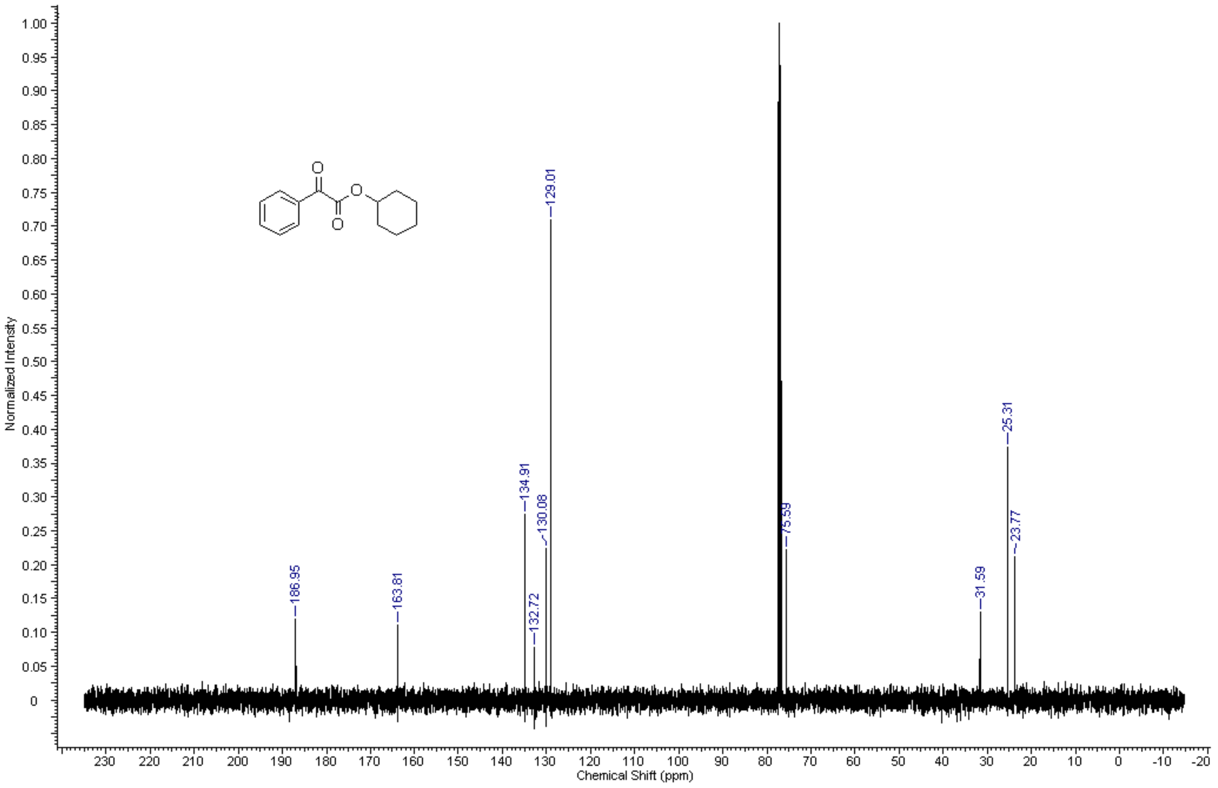

Ethyl 2-oxo-2-(pyridin-2-yl)acetate 5n

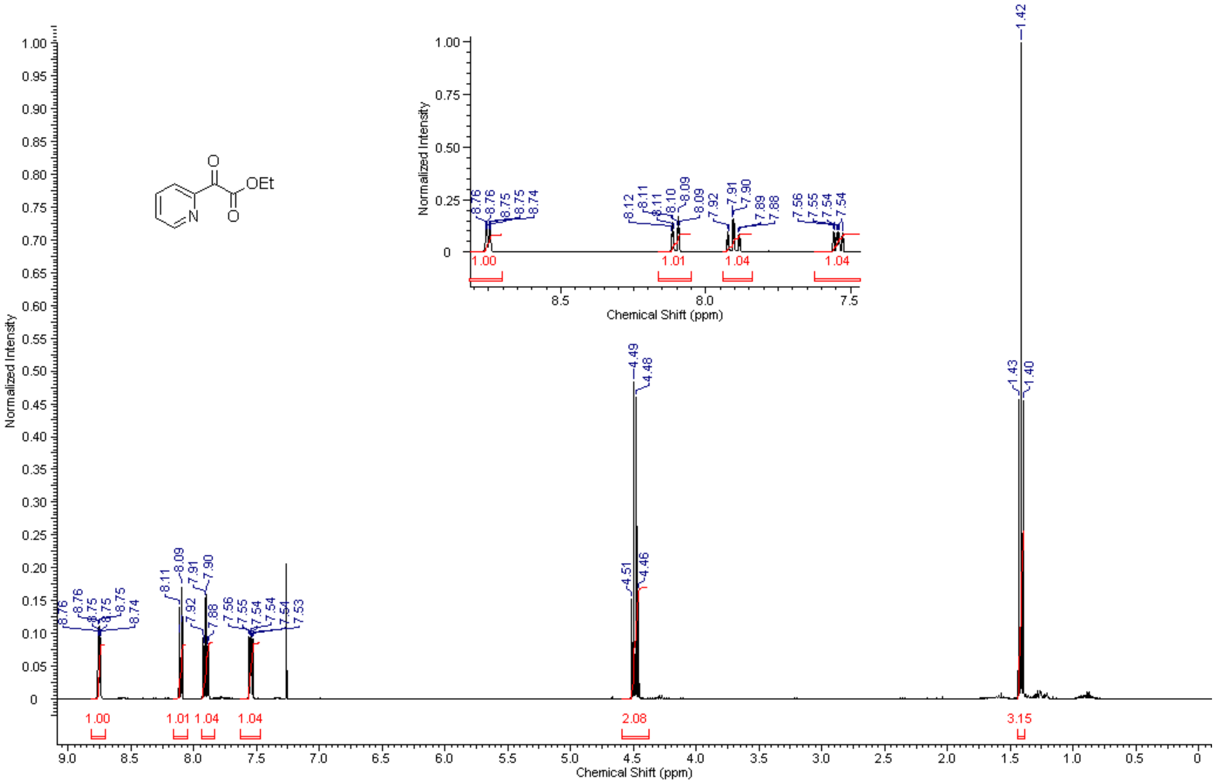

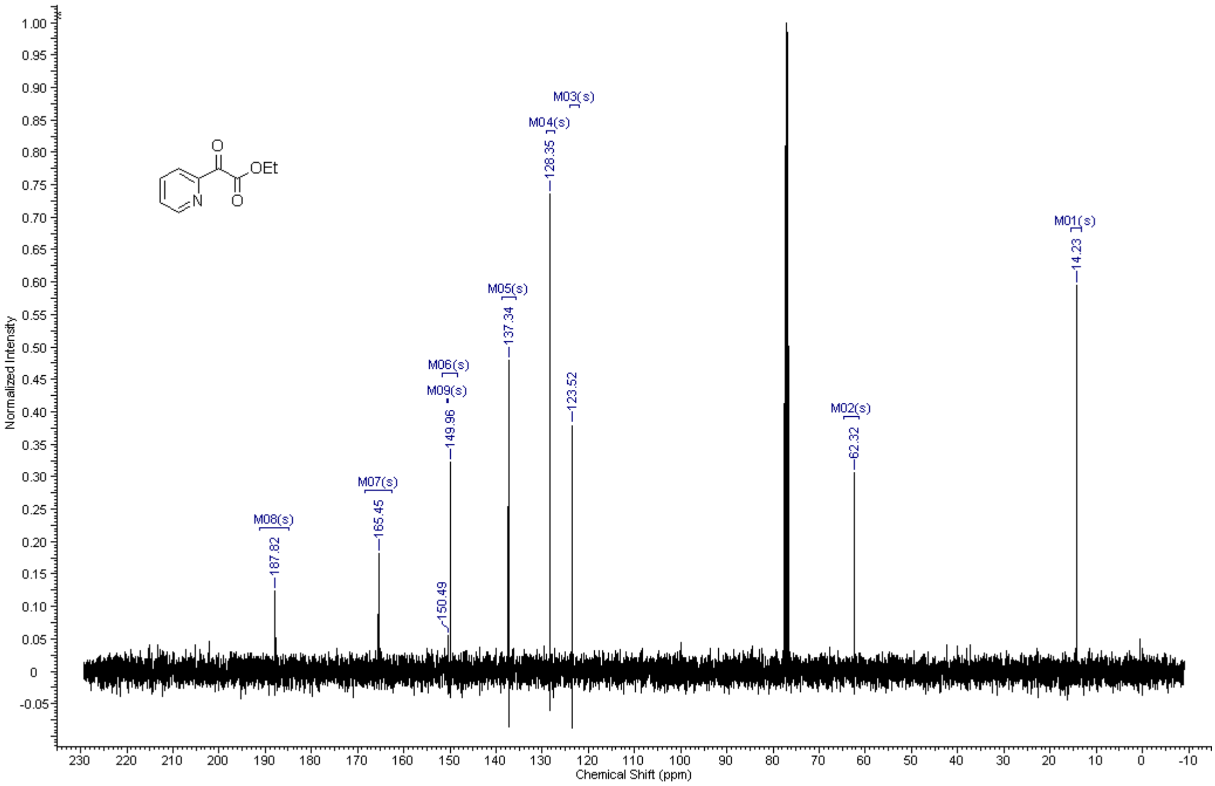

N-Allyl-2-oxo-2-phenylacetamide 5p

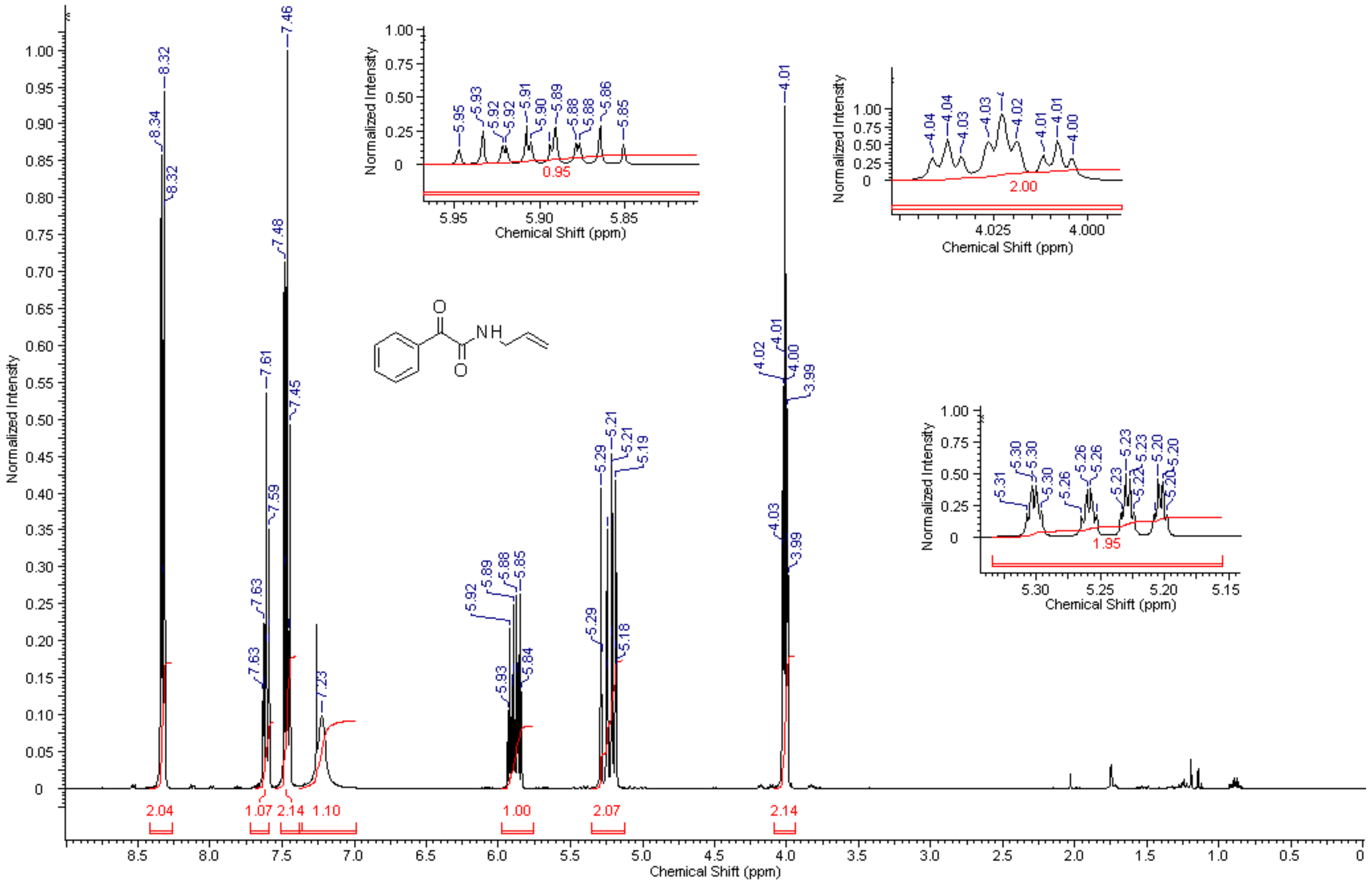

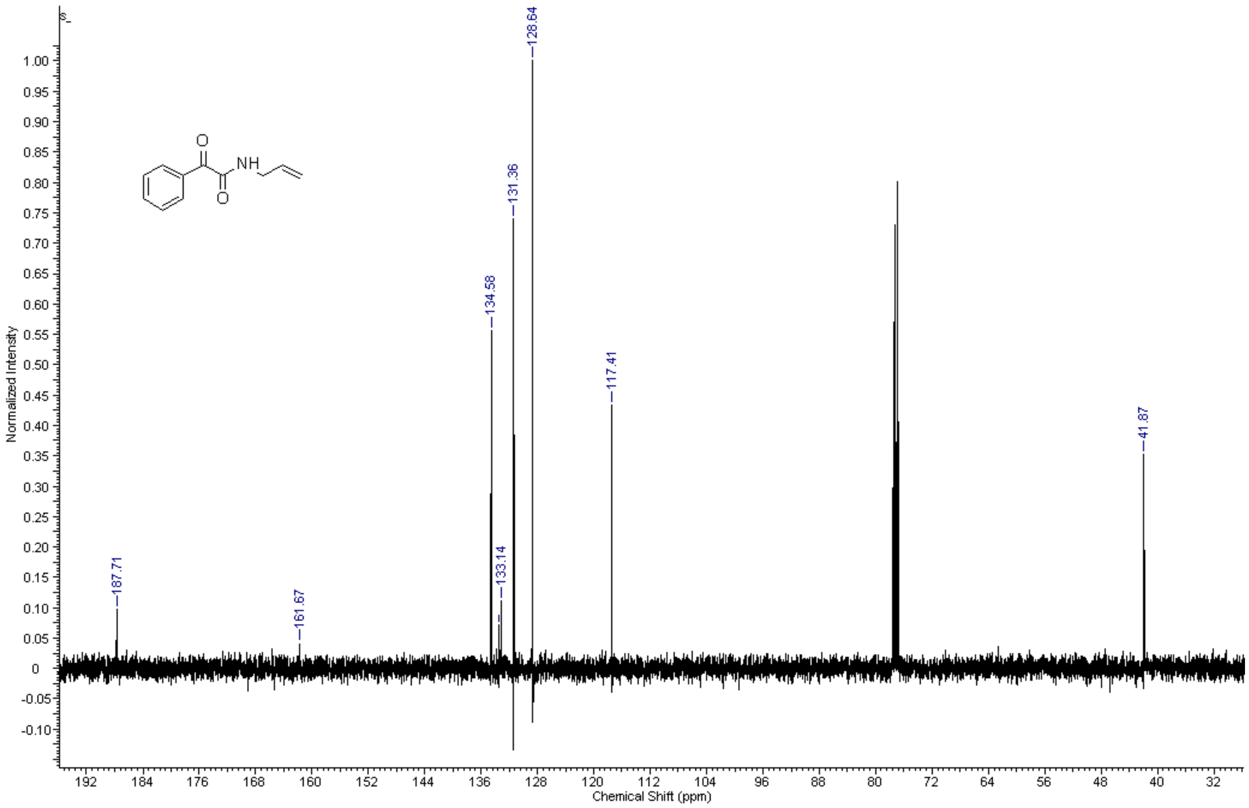

_N_-(2,5-Dimethoxyphenyl)-2-oxo-2-phenylacetamide 5q

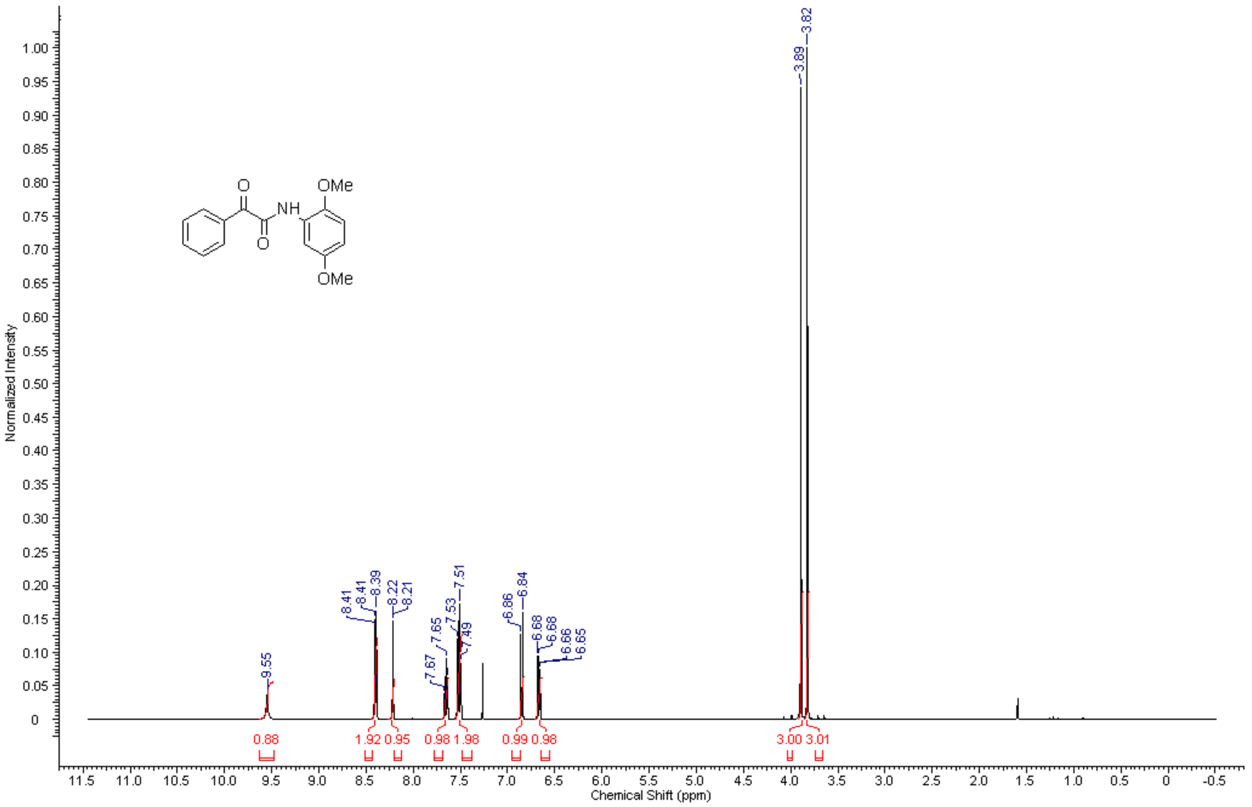

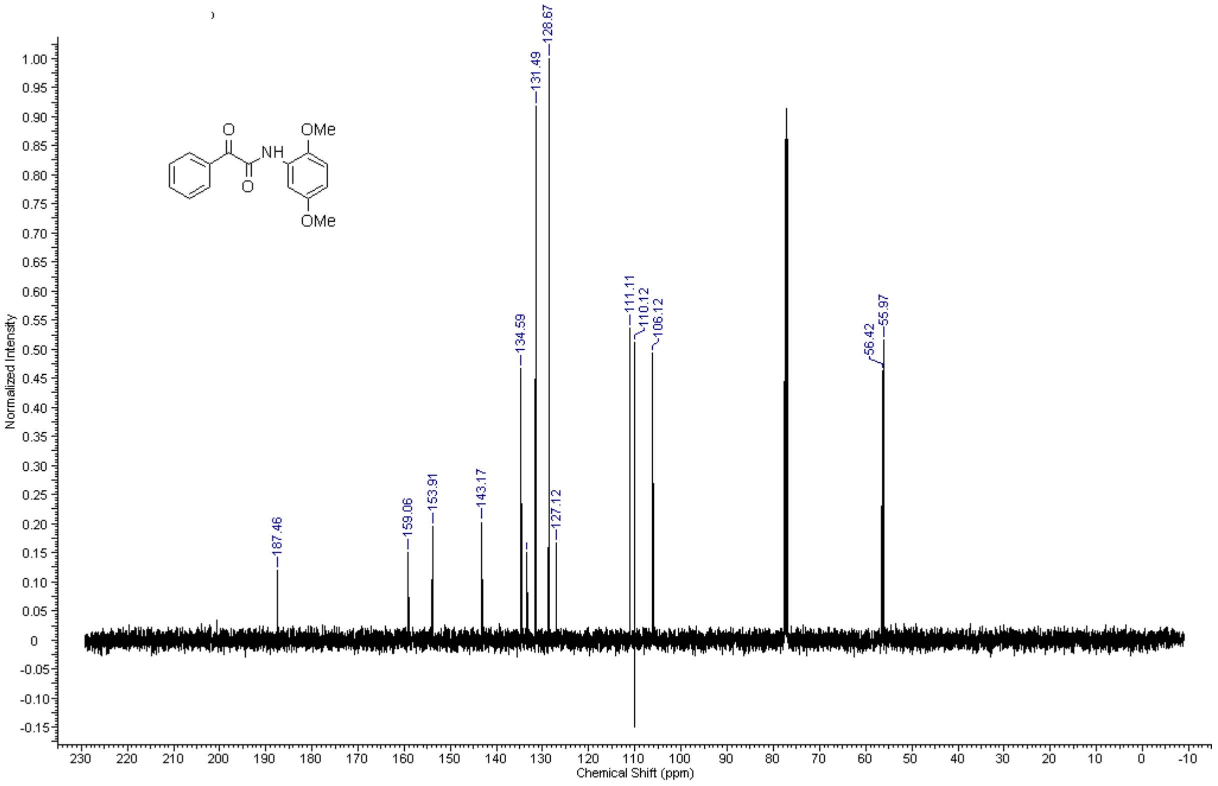

***N*-(2-Furylmethyl)-2-oxo-2-phenylacetamide 5r**

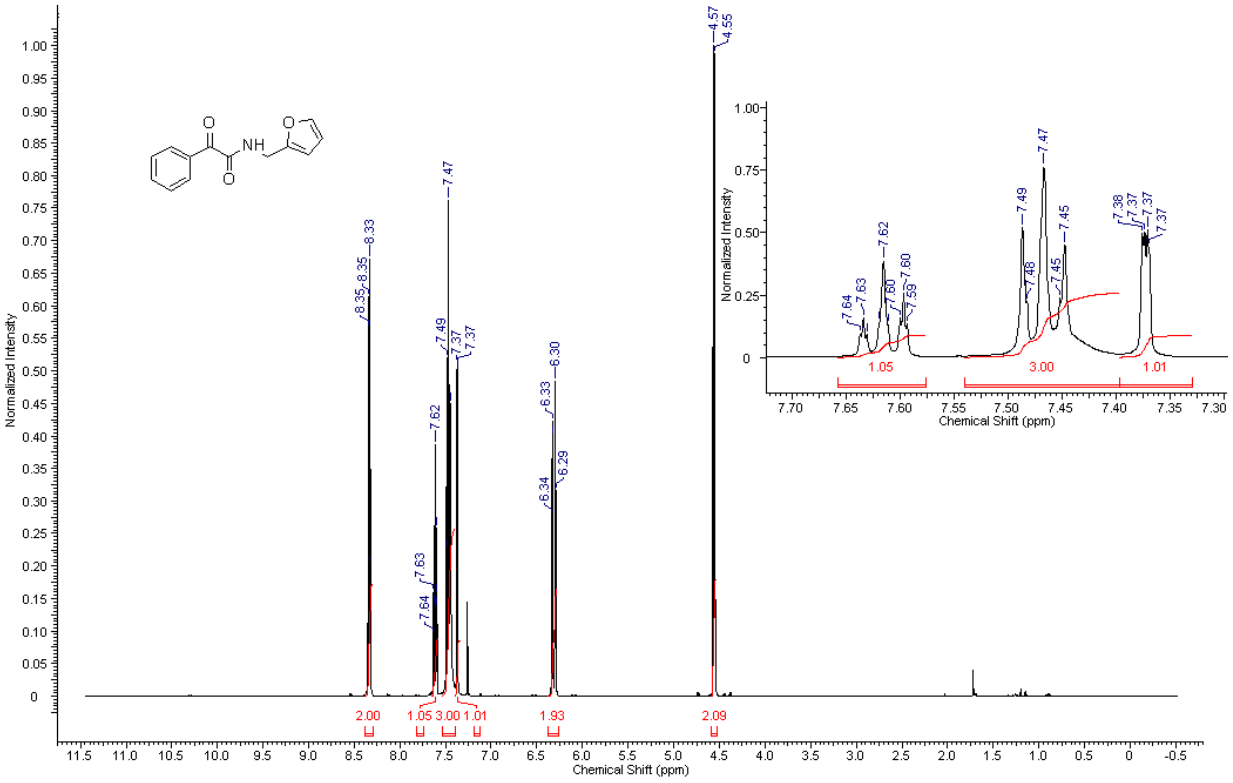

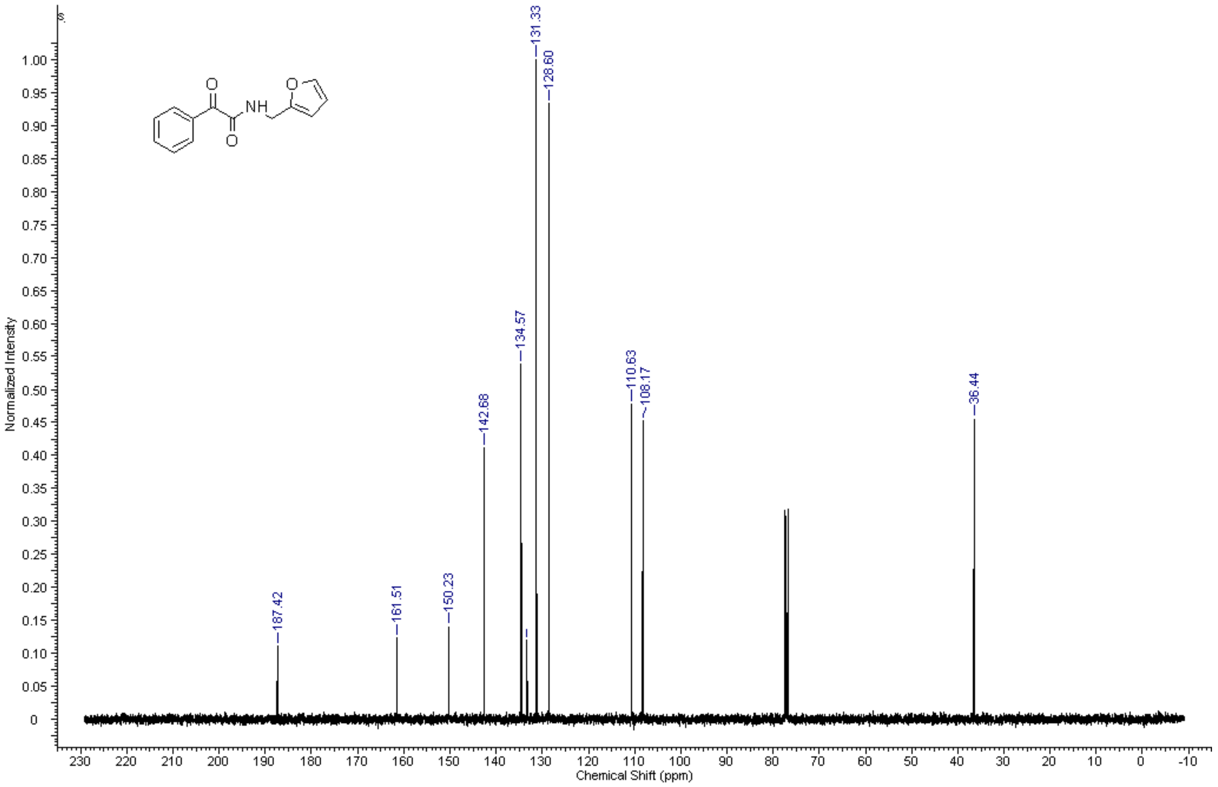

(*E*)- and (*Z*)-Ethyl phenylglyoxylate hydrazone 6a

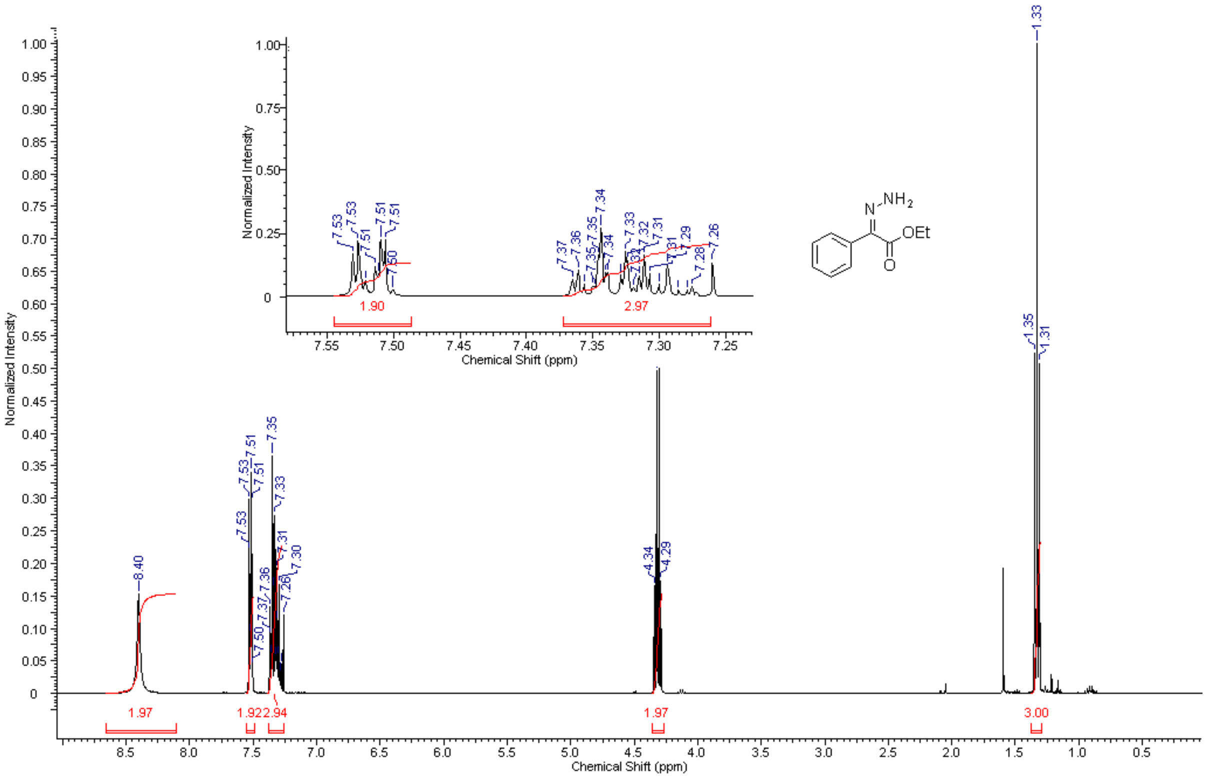

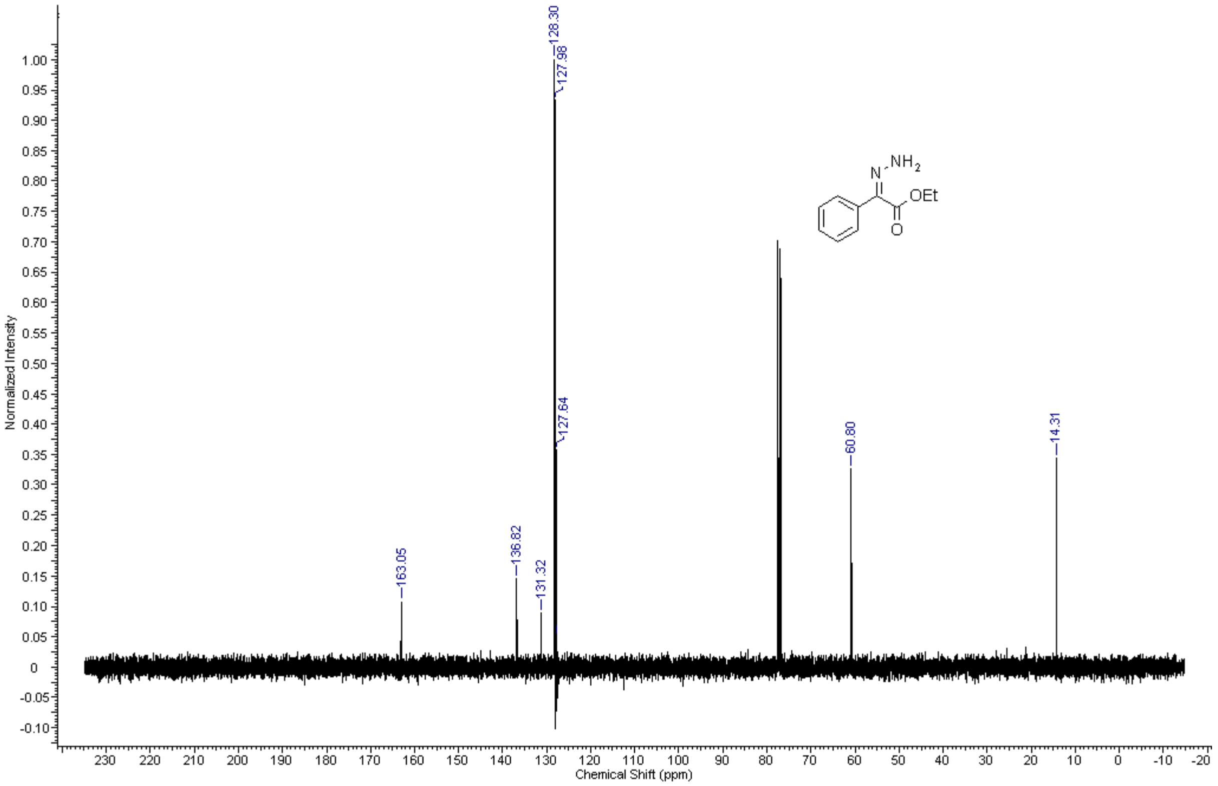

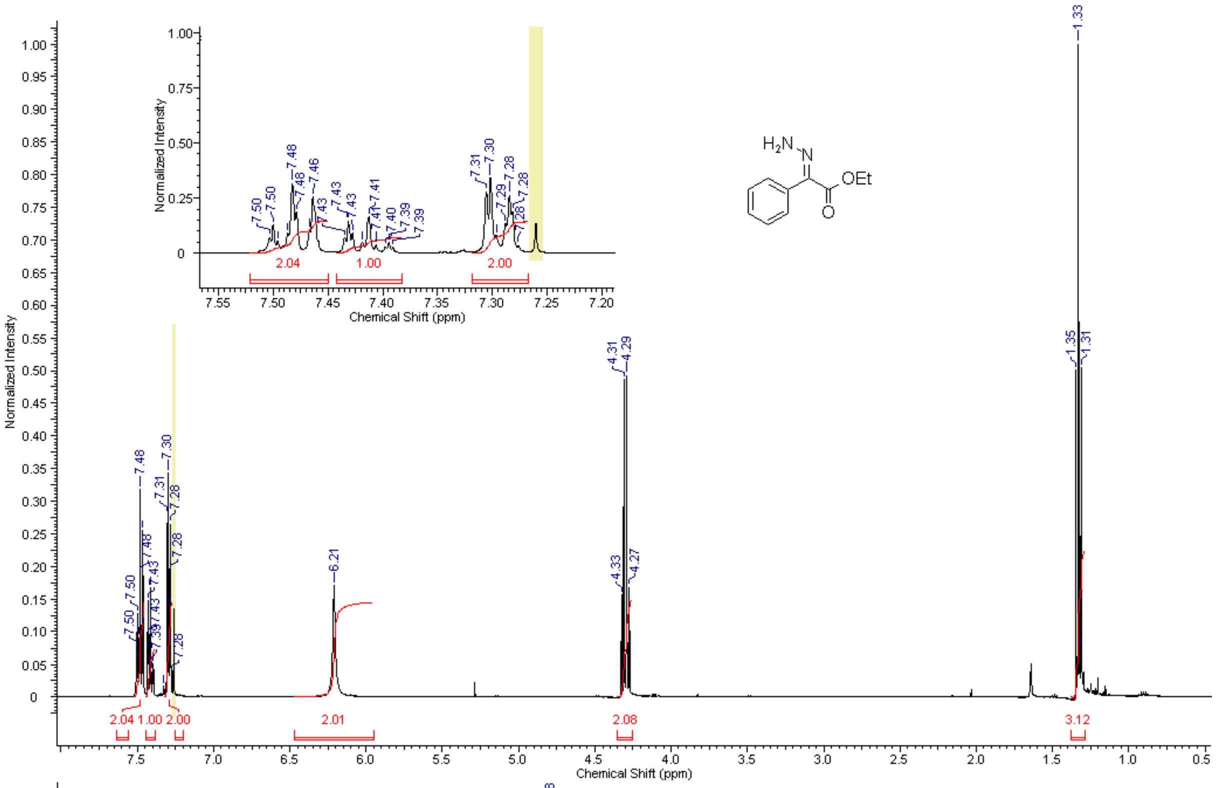
H2N
N
OEt
O
Normalized Intensity
Chemical Shift (ppm)
7.50
7.50
7.48
7.48
7.46
7.43
7.43
7.43
7.41
7.41
7.40
7.39
7.39
7.31
7.30
7.29
7.28
7.28
7.28
6.21
4.33
4.31
4.29
4.27
1.35
1.33
1.31
2.04
1.00
2.00
2.01
2.08
3.12

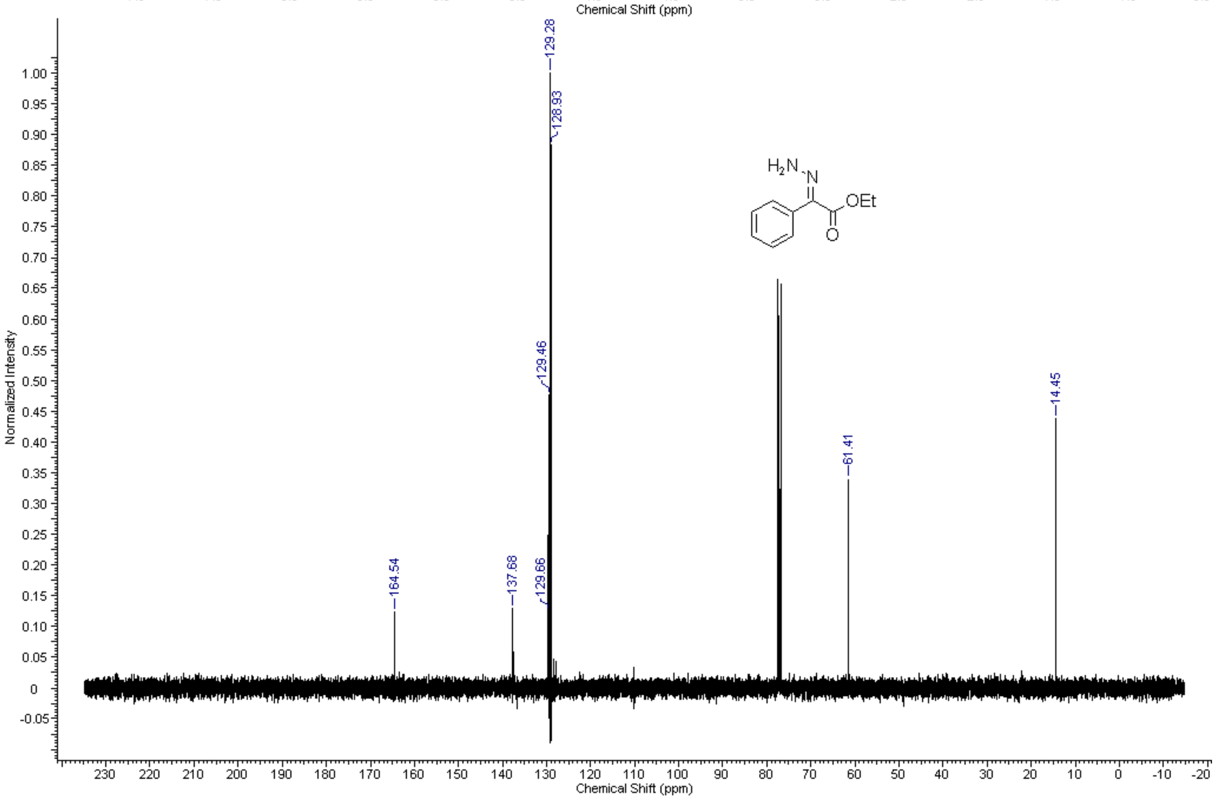
H2N
N
OEt
O
Normalized Intensity
Chemical Shift (ppm)
164.54
137.68
129.66
129.46
129.28
128.93
61.41
14.45

(*E*)- and (*Z*)-Ethyl 2-hydrazono-2-(4-methoxyphenyl)acetate 6b

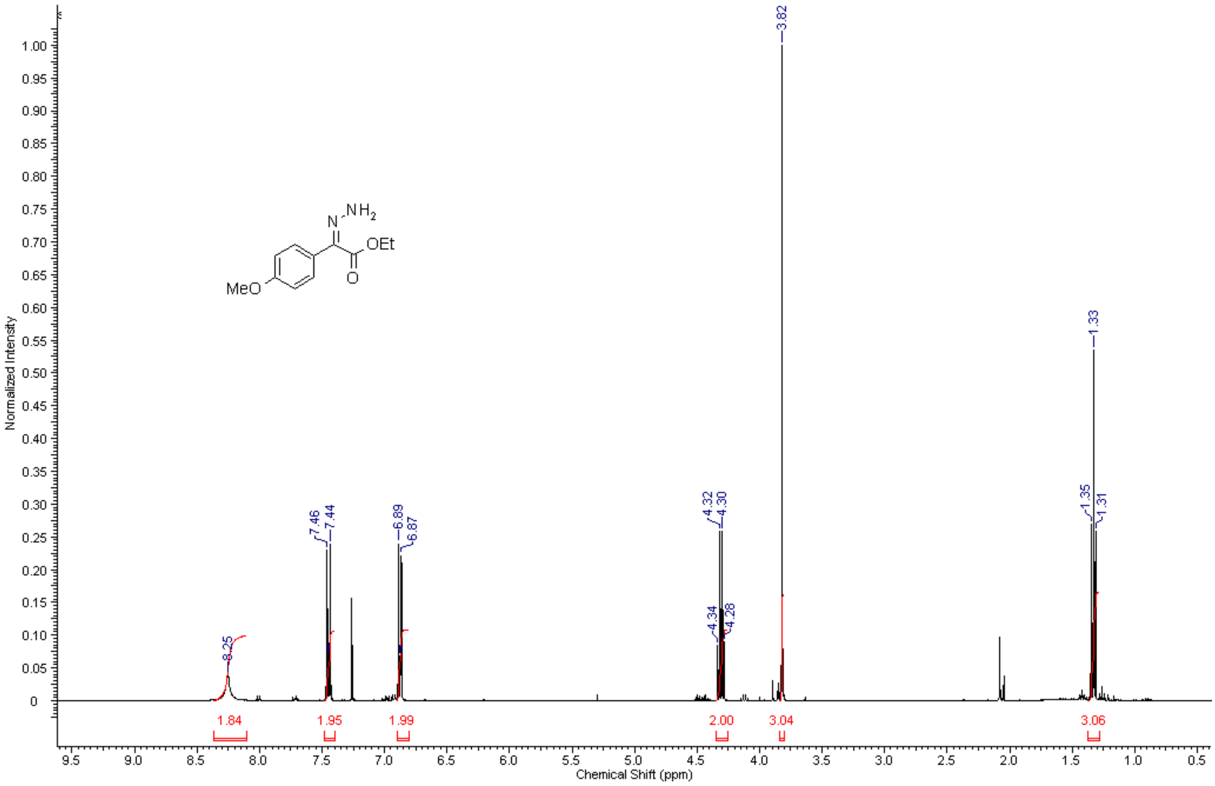

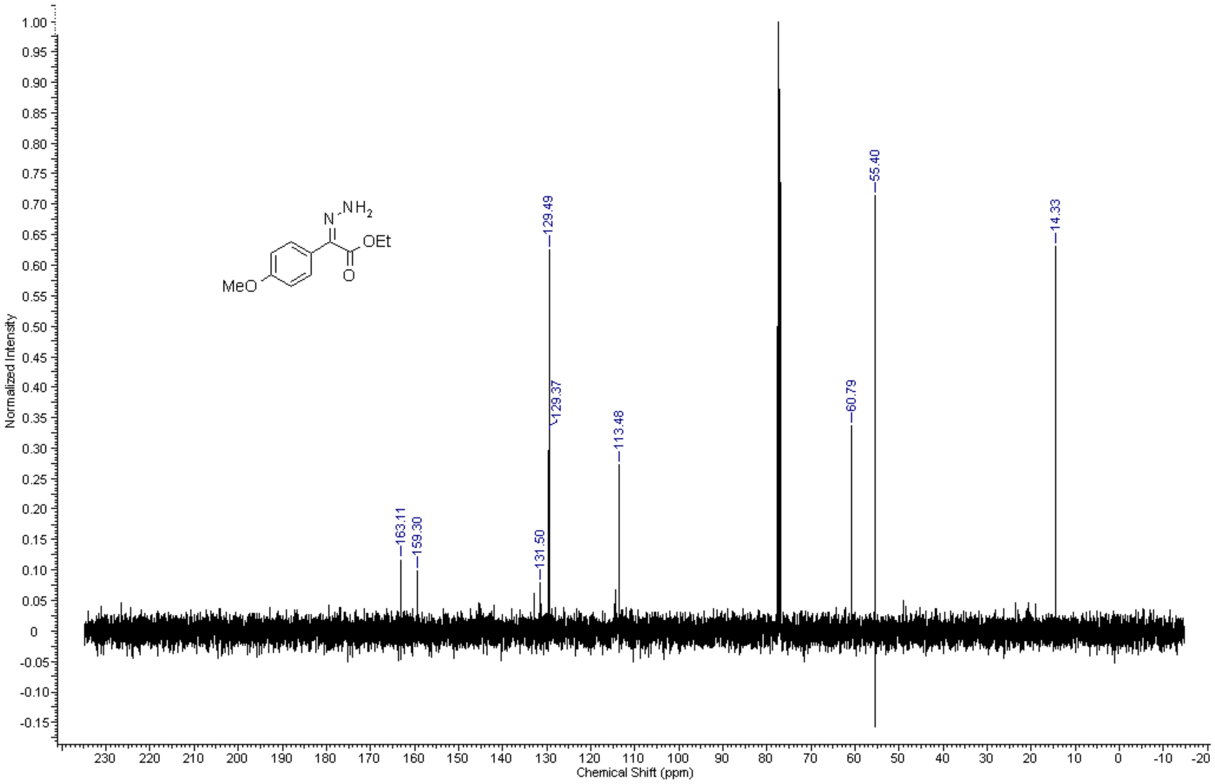

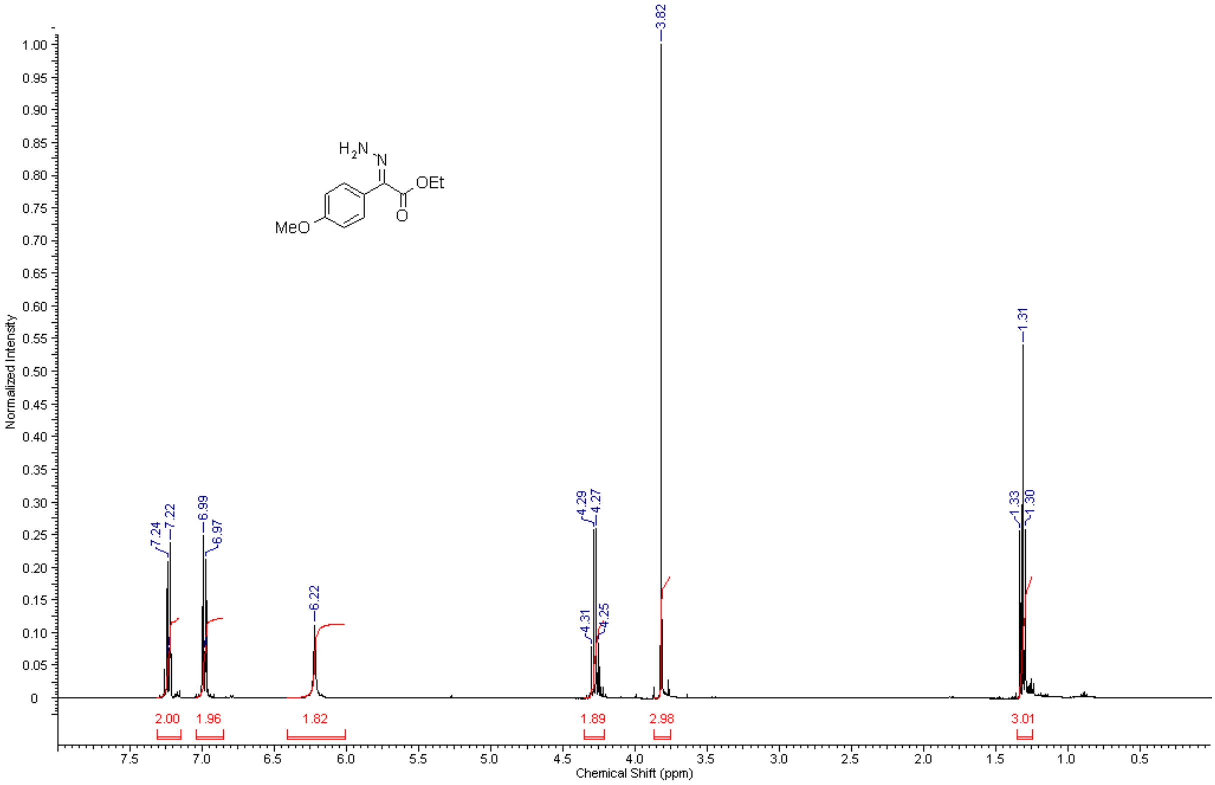
H2N
N
OEt
O
MeO
7.24
7.22
6.99
6.97
6.22
4.31
4.29
4.27
4.25
3.82
1.33
1.31
1.30
2.00
1.96
1.82
1.89
2.98
3.01
Normalized Intensity
Chemical Shift (ppm)

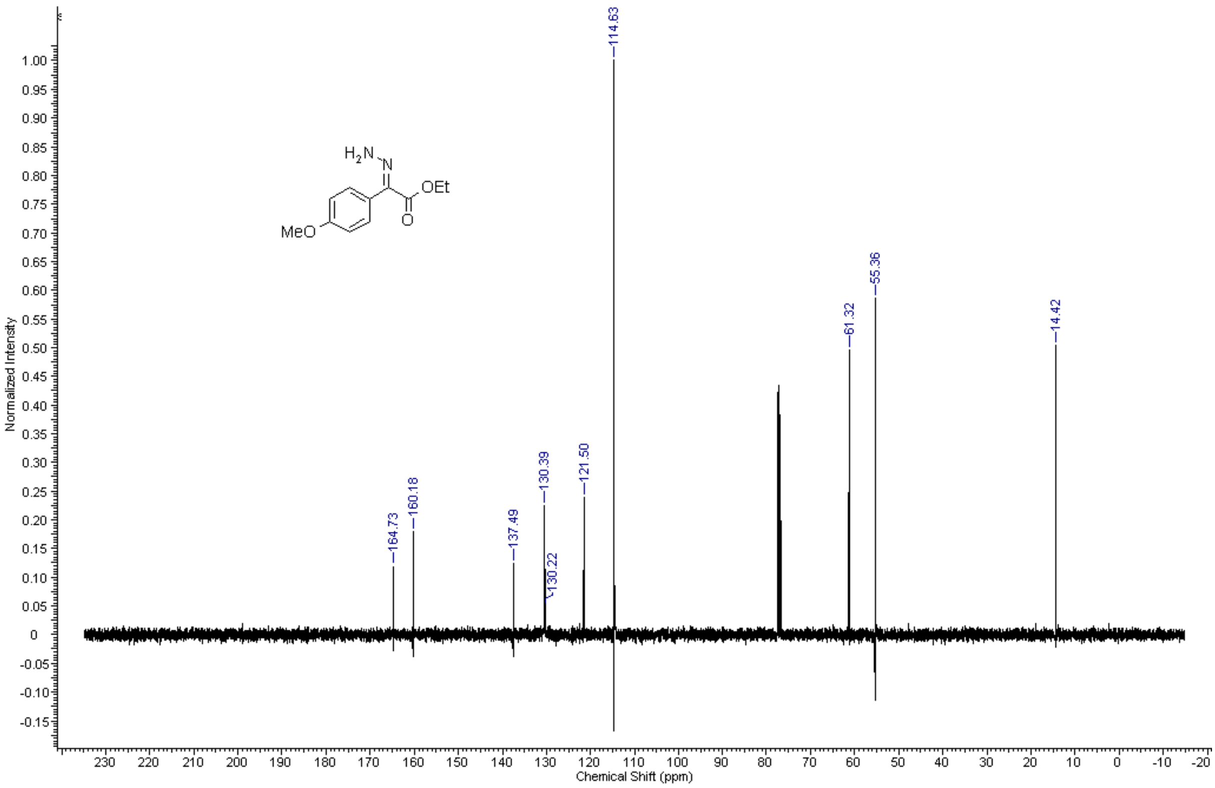
H2N
N
OEt
O
MeO
164.73
160.18
137.49
130.39
130.22
121.50
114.63
61.32
55.36
14.42
Normalized Intensity
Chemical Shift (ppm)

(E)- and (*Z*)-Ethyl 2-hydrazono-2-(2-methoxyphenyl)acetate 6c

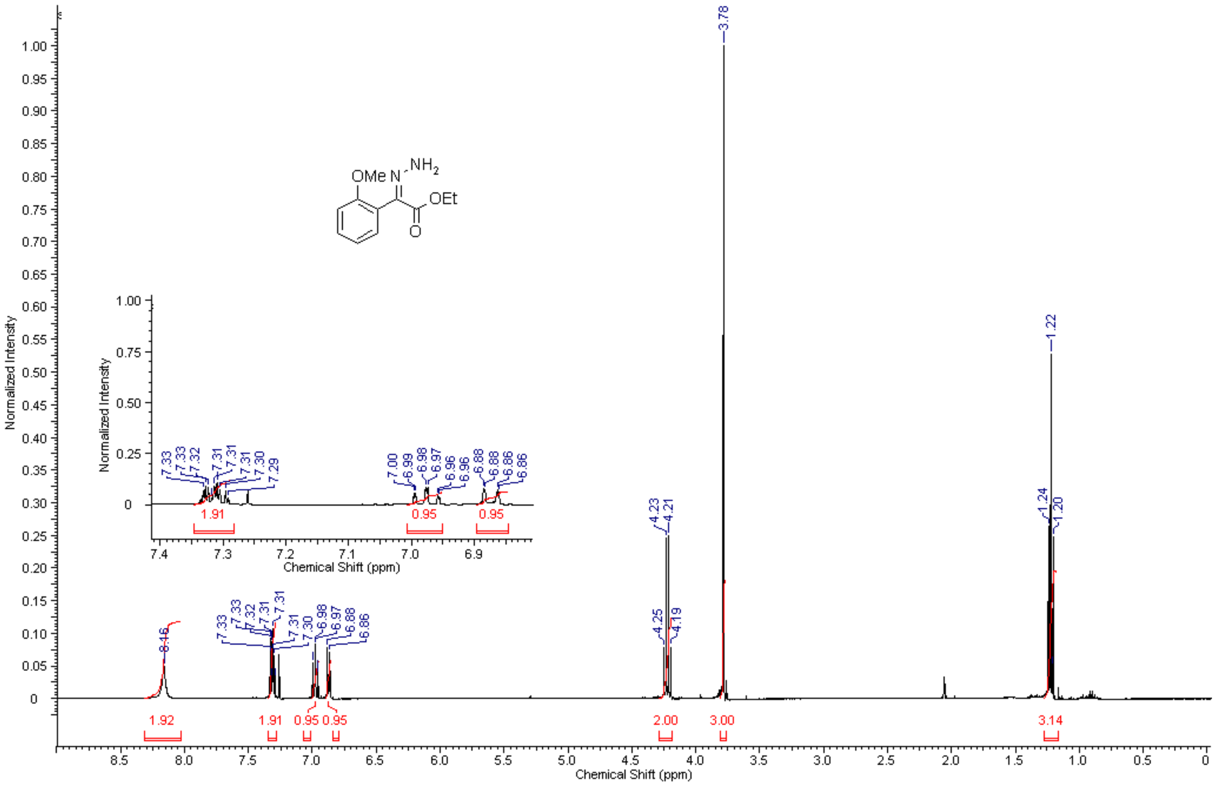

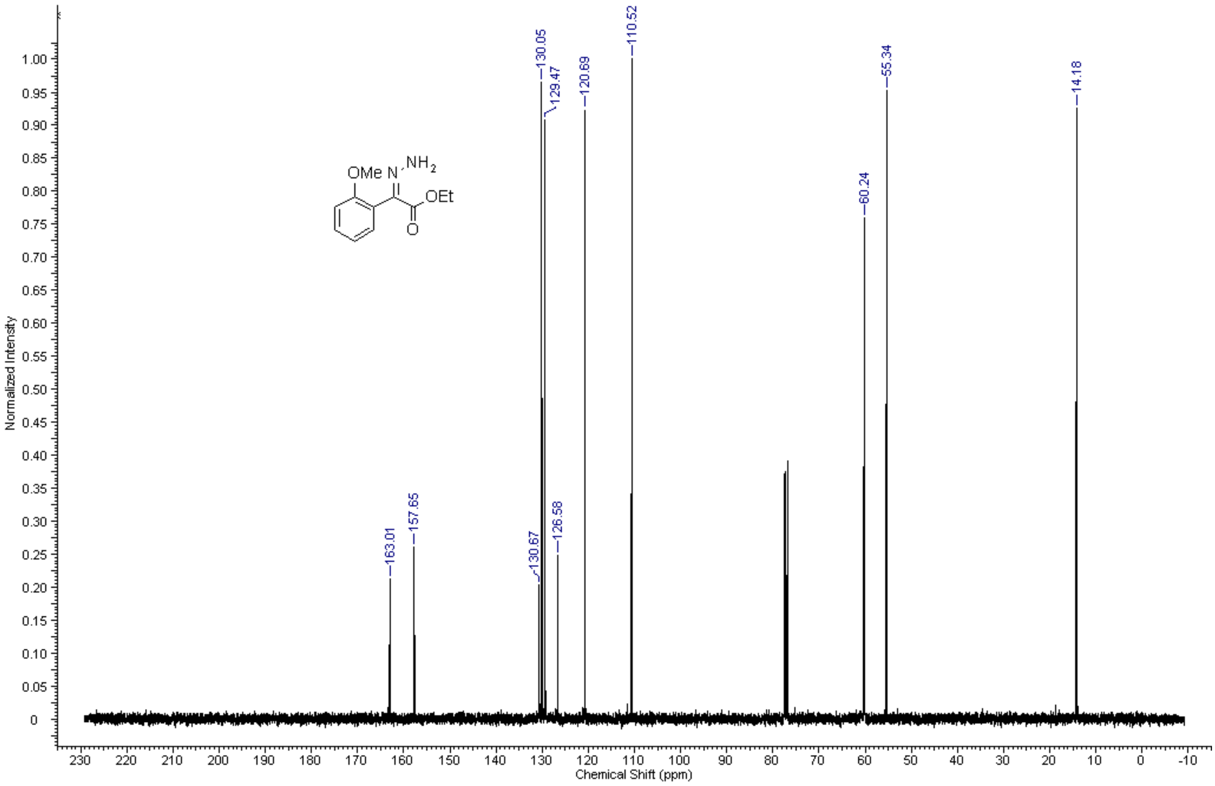

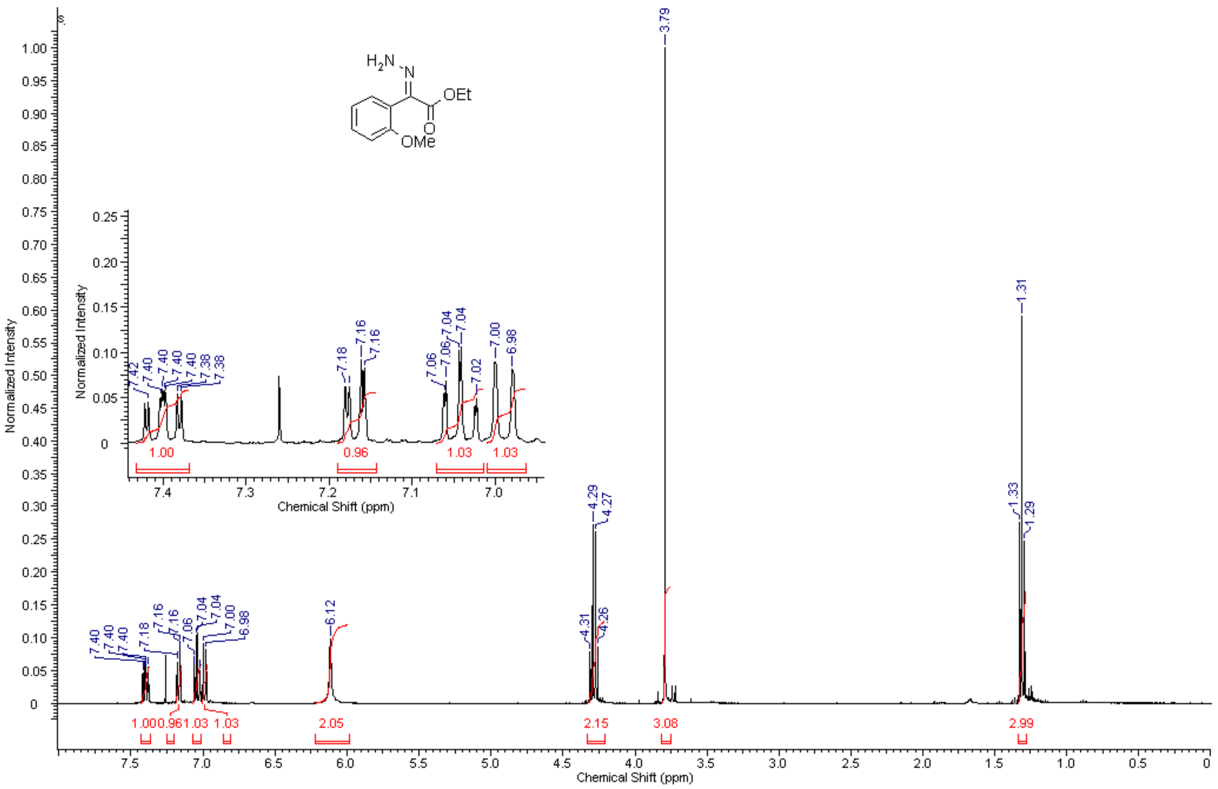
H2N N
OEt
O
OMe
Normalized Intensity
Chemical Shift (ppm)
3.79
1.31
1.33
1.29
4.29
4.27
4.31
4.26
6.12
7.40
7.40
7.40
7.18
7.16
7.16
7.06
7.04
7.04
7.00
6.98
1.00
0.96
1.03
1.03
2.05
2.15
3.08
2.99

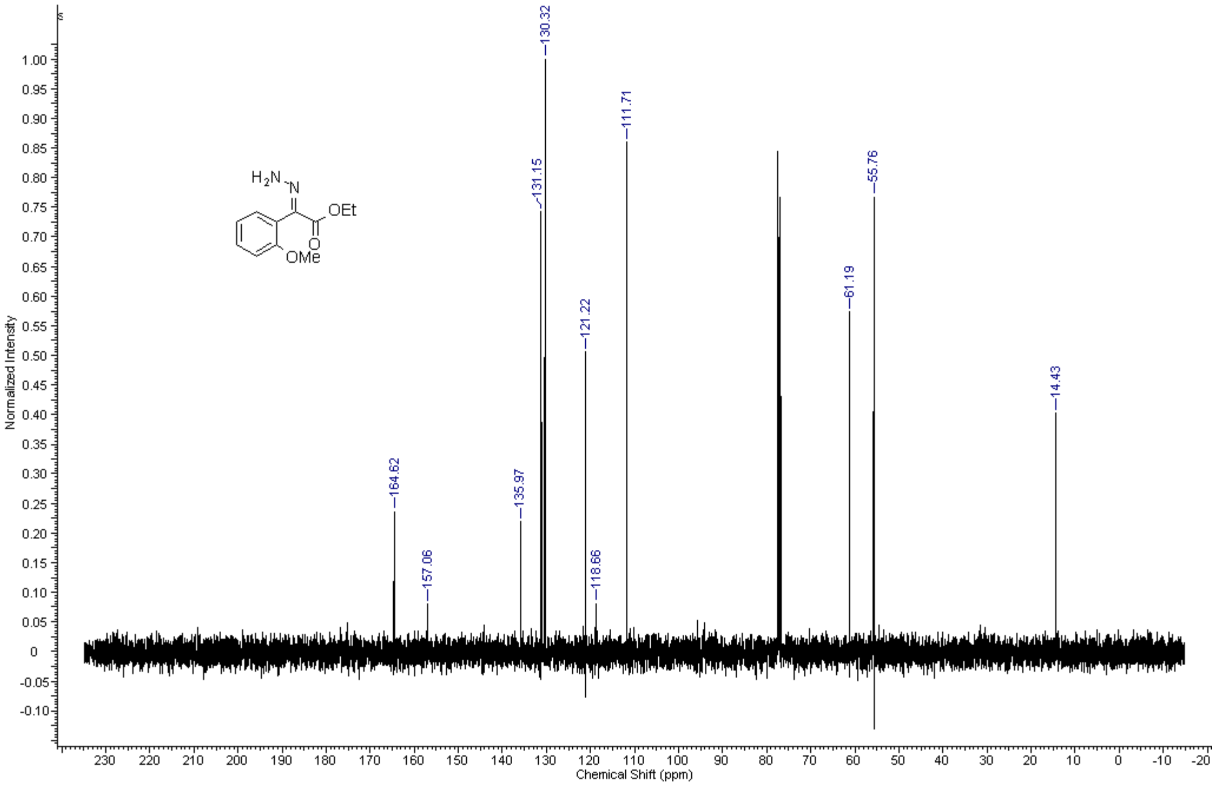
H2N N
OEt
O
OMe
Normalized Intensity
Chemical Shift (ppm)
130.32
131.15
111.71
121.22
118.66
135.97
164.62
157.06
61.19
55.76
14.43

(E) and (Z)-Ethyl 2-(4-bromophenyl)-2-hydrazonoacetate 6d

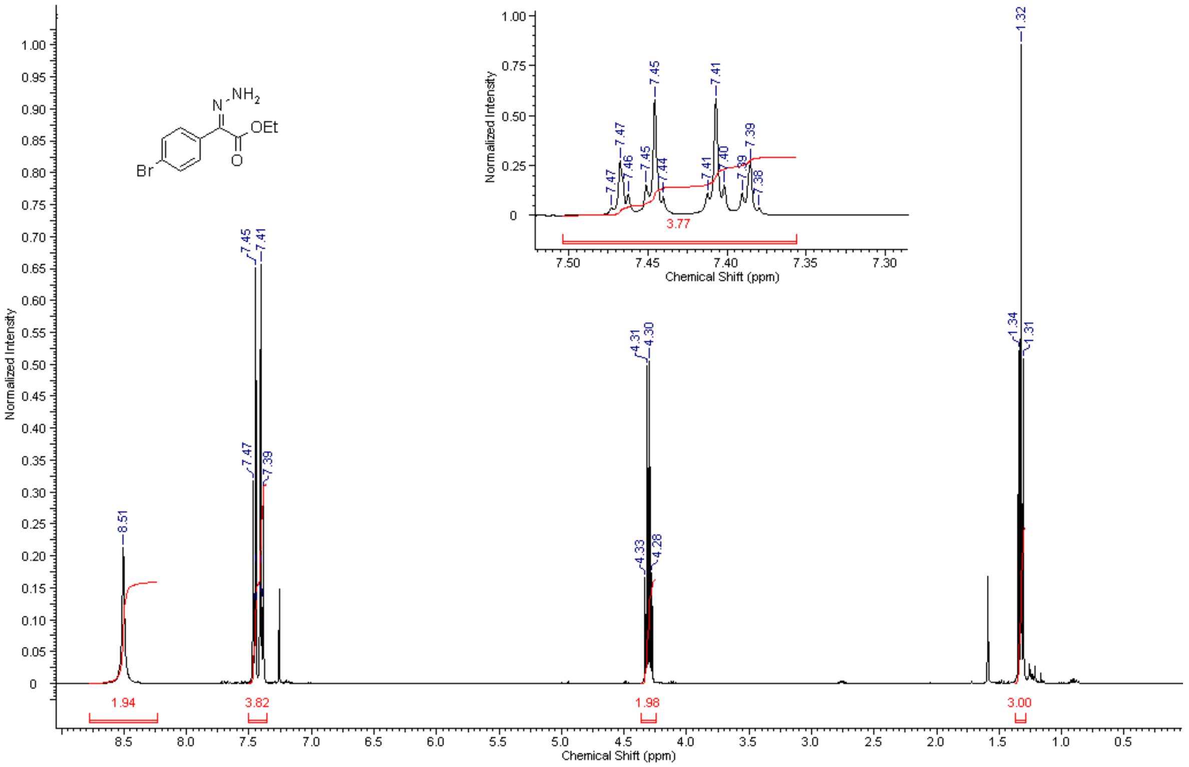

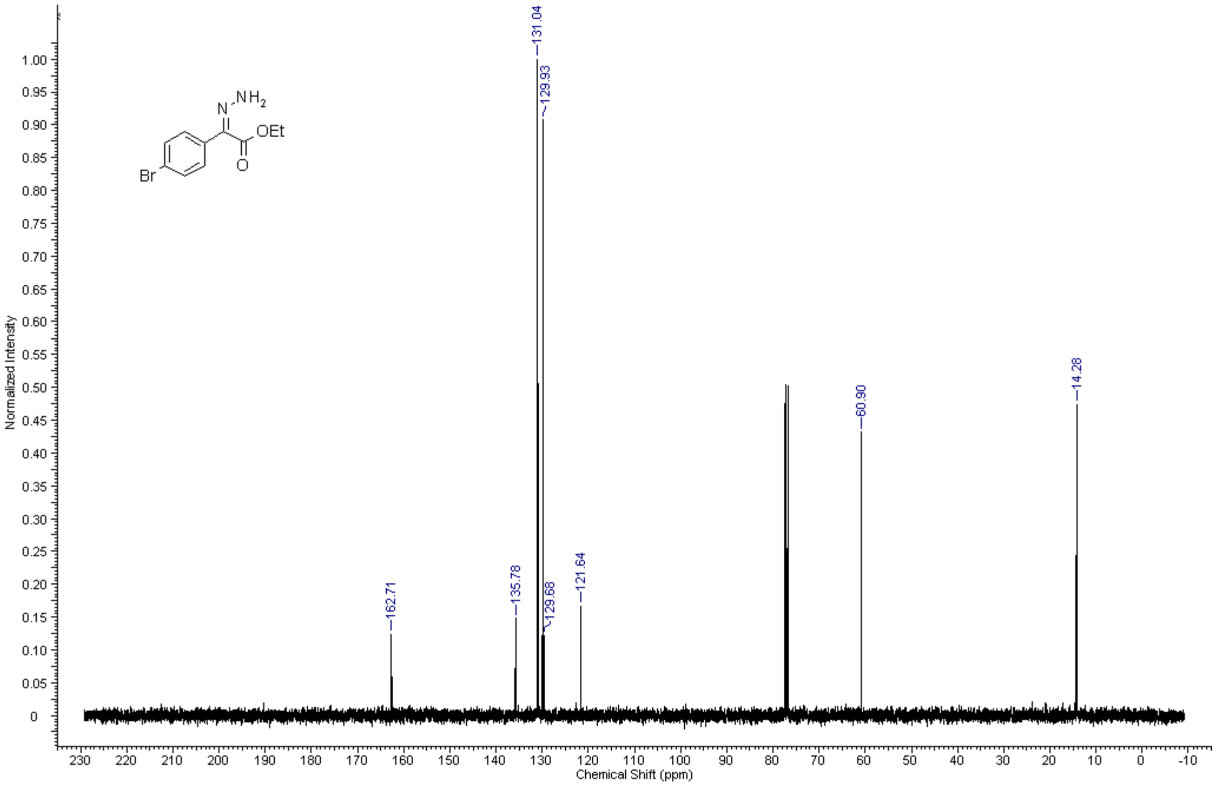

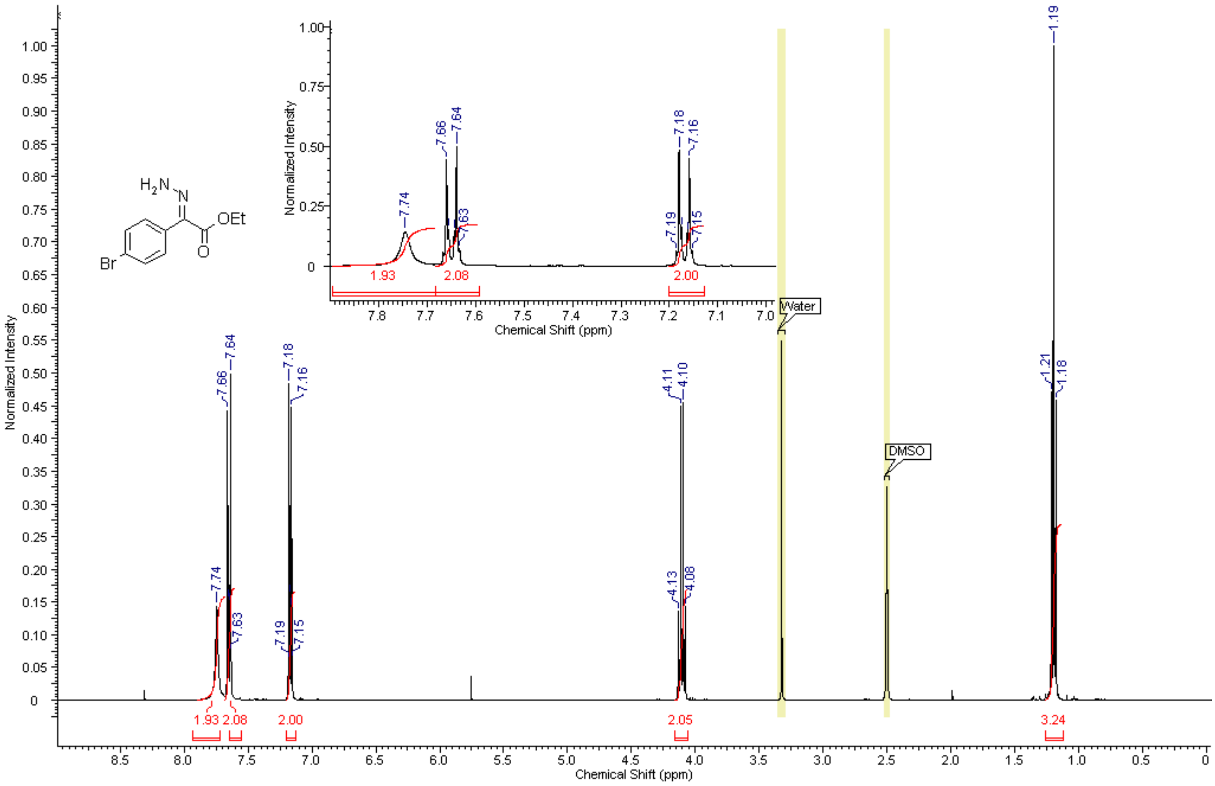
H2N
N
OEt
O
Br
Normalized Intensity
Chemical Shift (ppm)
7.74
7.66
7.64
7.63
7.19
7.18
7.16
7.15
4.13
4.11
4.10
4.08
Water
DMSO
1.21
1.19
1.18
1.93
2.08
2.00
2.05
3.24

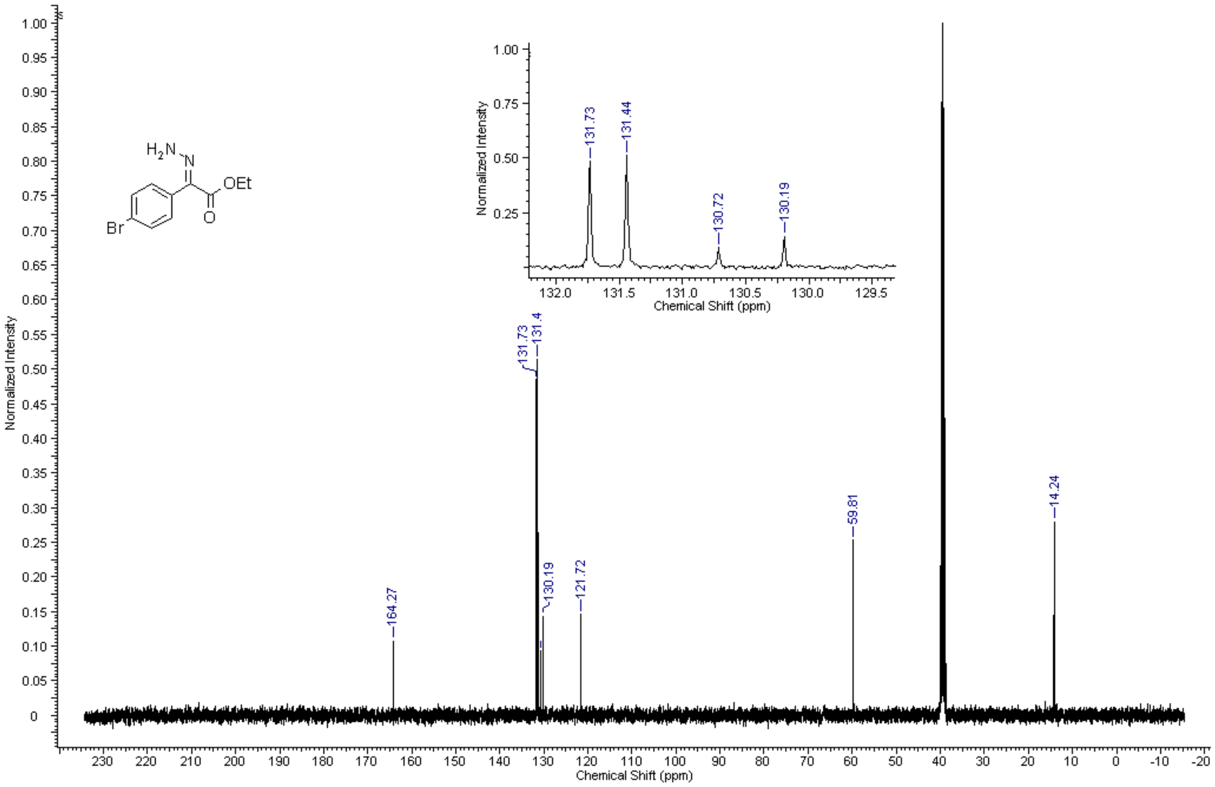
H2N
N
OEt
O
Br
Normalized Intensity
Chemical Shift (ppm)
164.27
131.73
131.4
131.44
130.72
130.19
121.72
59.81
14.24

***(E)*- and *(Z)*-Ethyl 3-methyl-2-oxobutanoate hydrazone 6e**

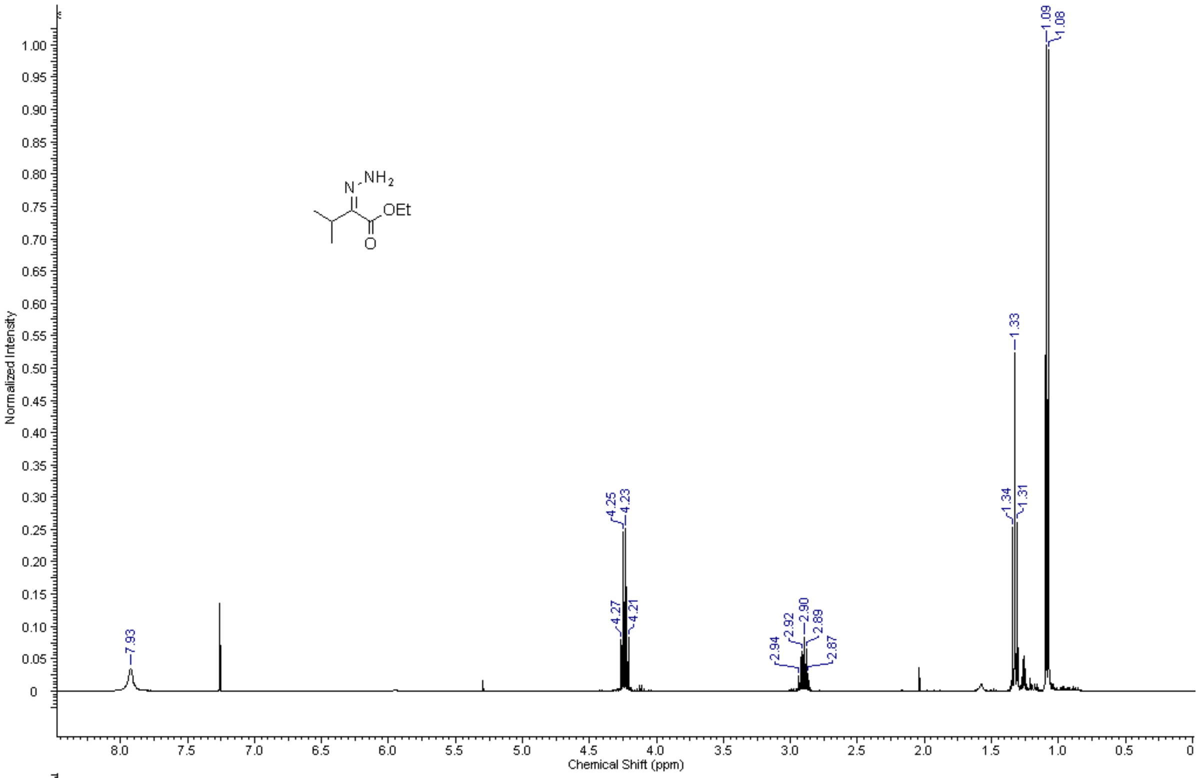

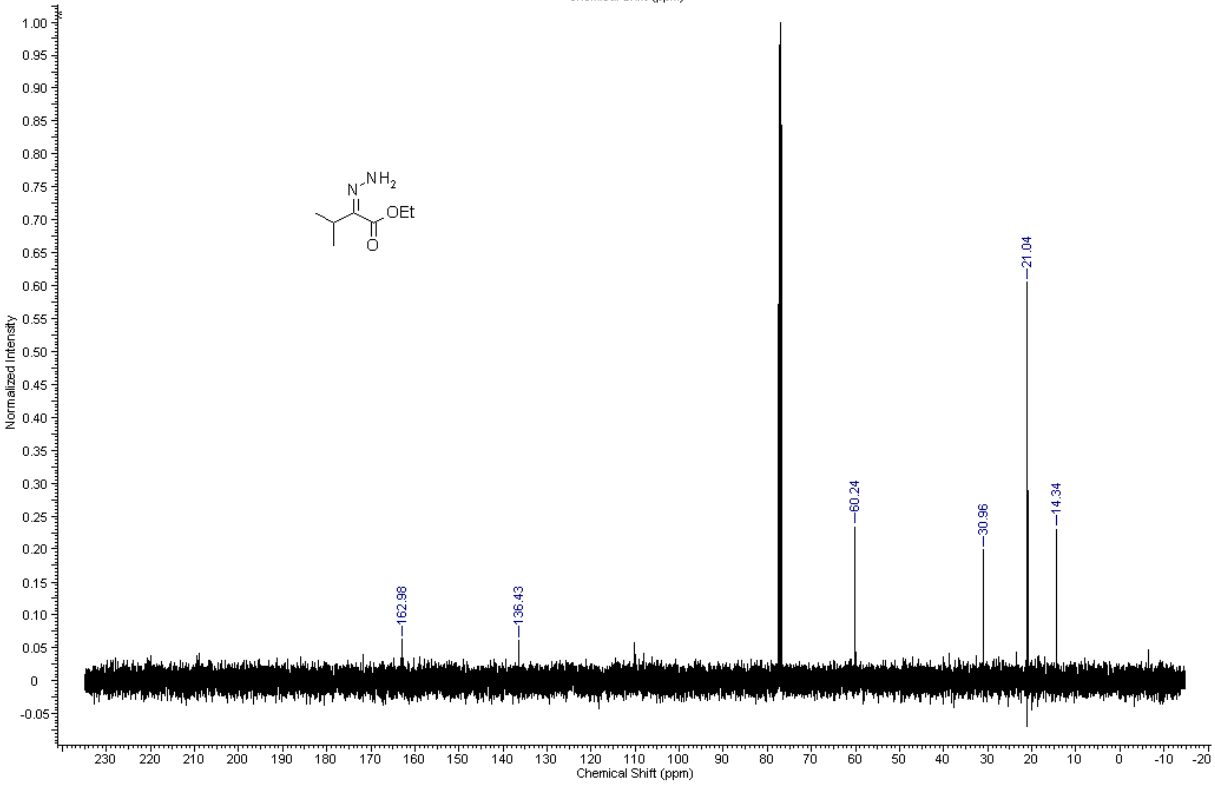

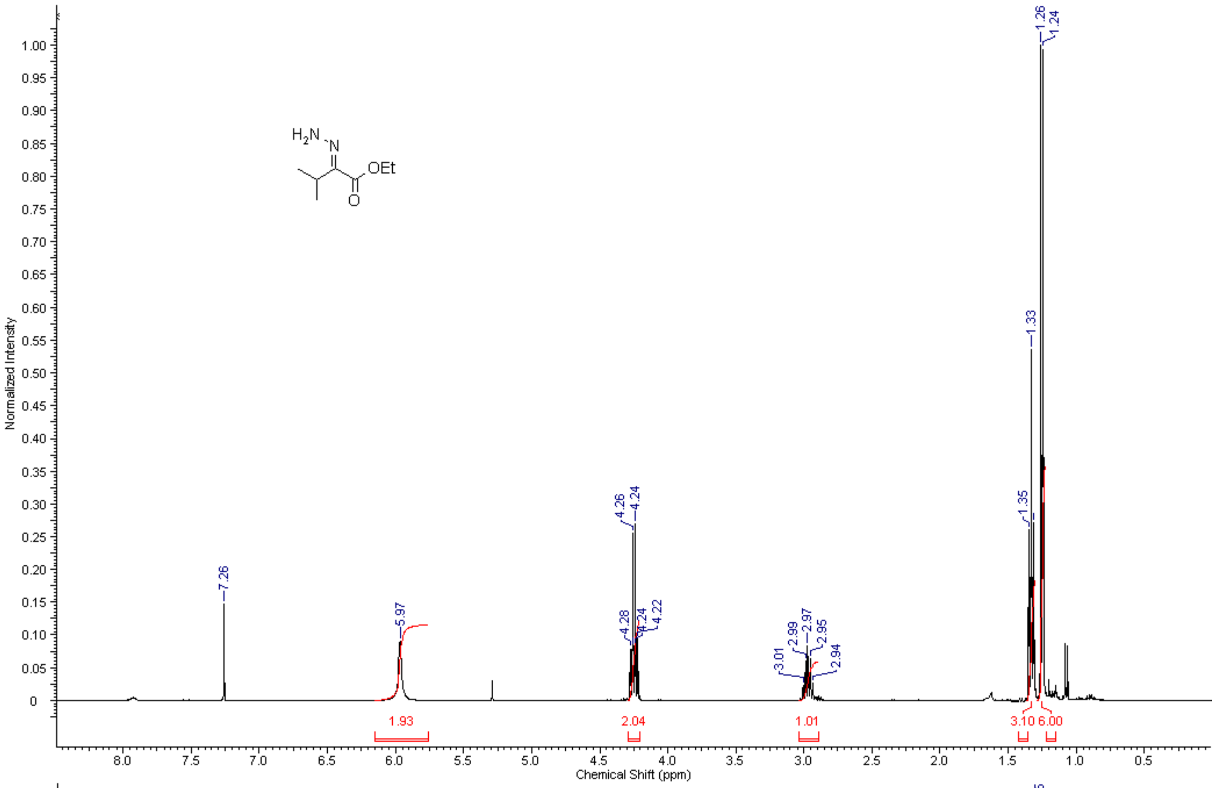
H2N
N
OEt
O
Normalized Intensity
Chemical Shift (ppm)
7.26
5.97
4.26
4.24
4.28
4.24
4.22
3.01
2.99
2.97
2.95
2.94
1.35
1.33
1.26
1.24
1.93
2.04
1.01
3.10
6.00

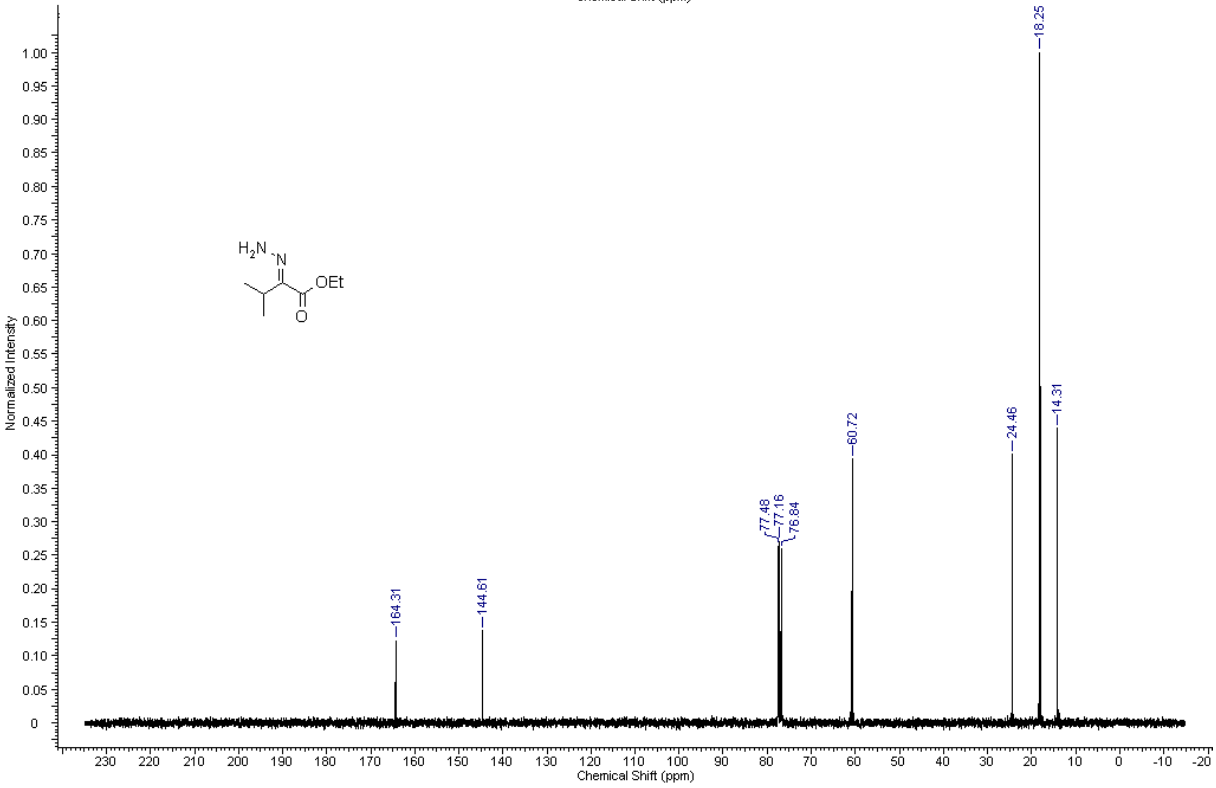
H2N
N
OEt
O
Normalized Intensity
Chemical Shift (ppm)
164.31
144.61
77.48
77.16
76.84
60.72
24.46
18.25
14.31

(*E*)-Ethyl pyruvate hydrazone 6f

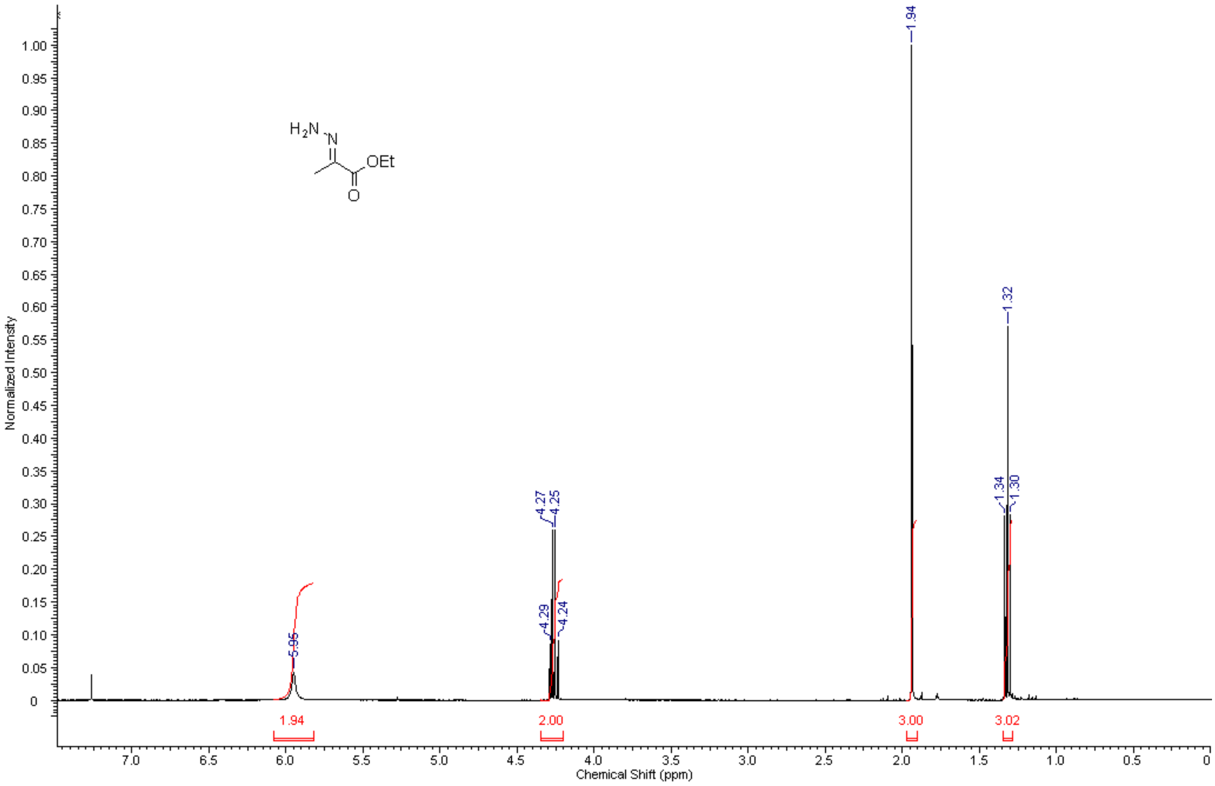

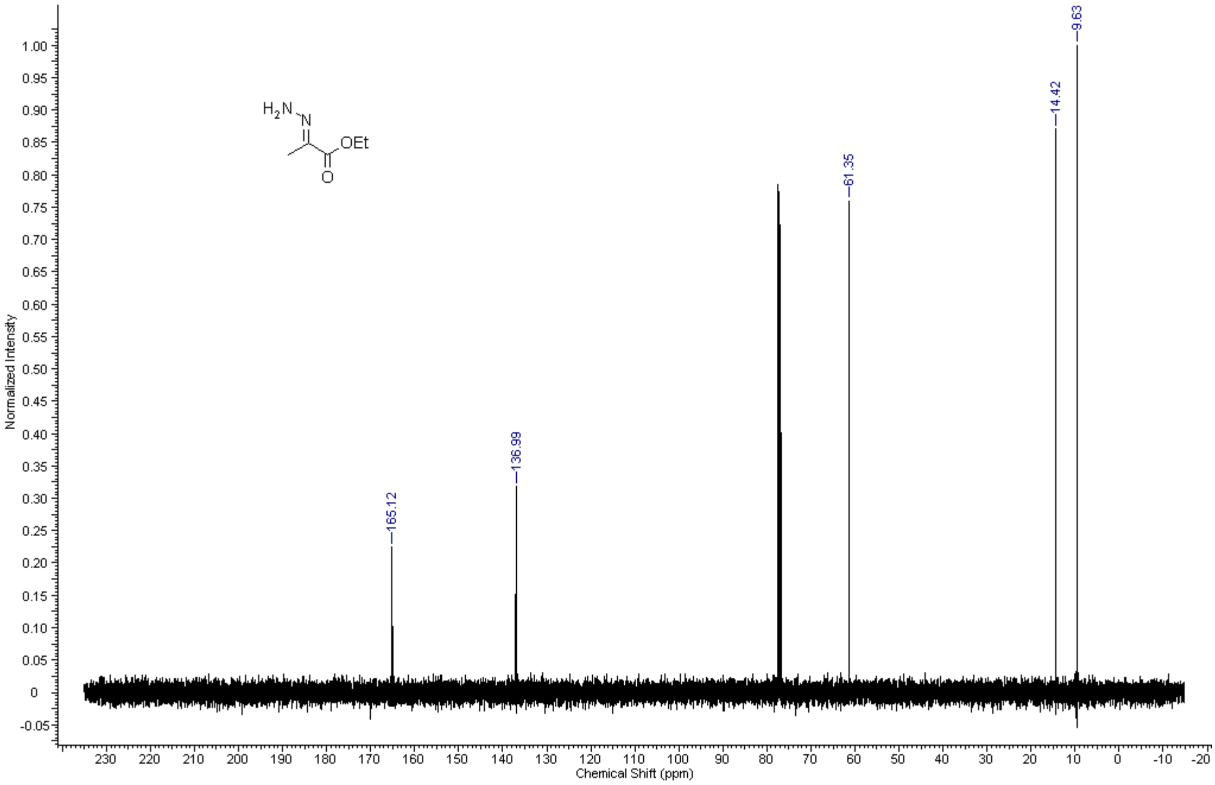

(*E*)- and (*Z*)-Ethyl 2-oxo-4-phenylbutanoate hydrazone 6g

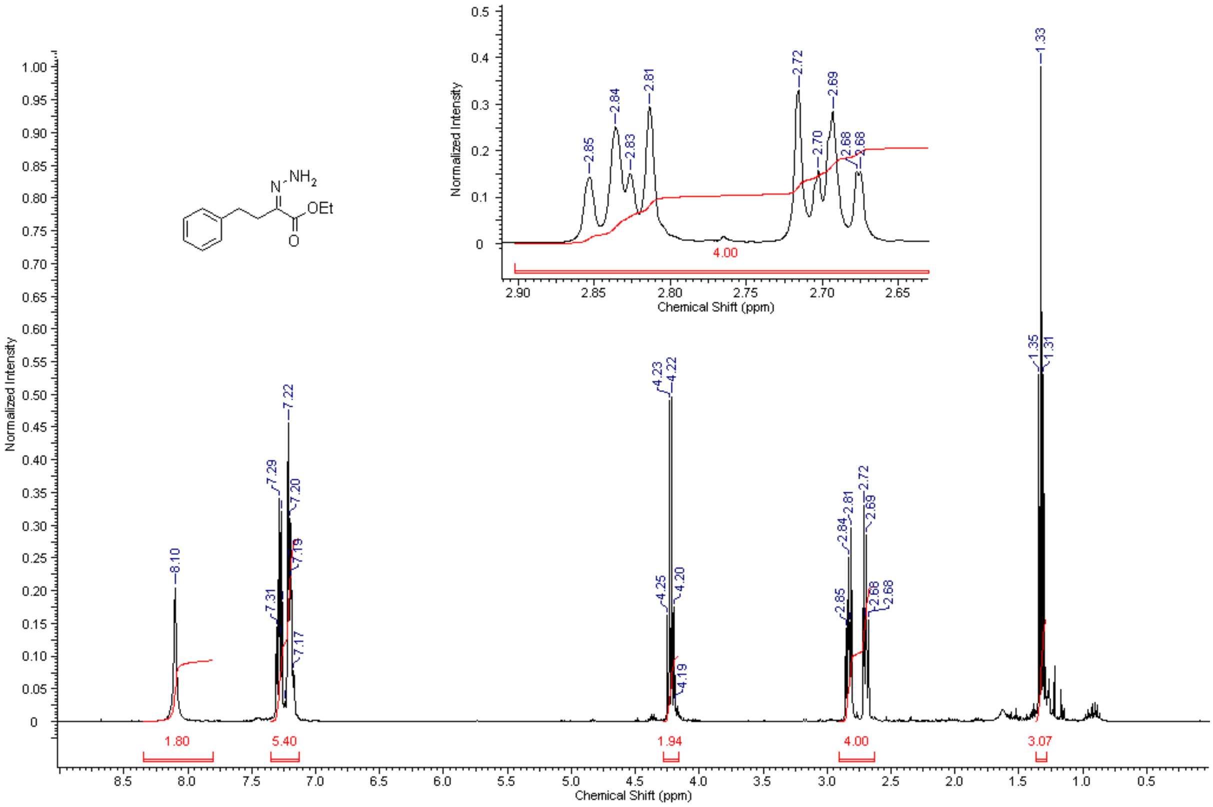

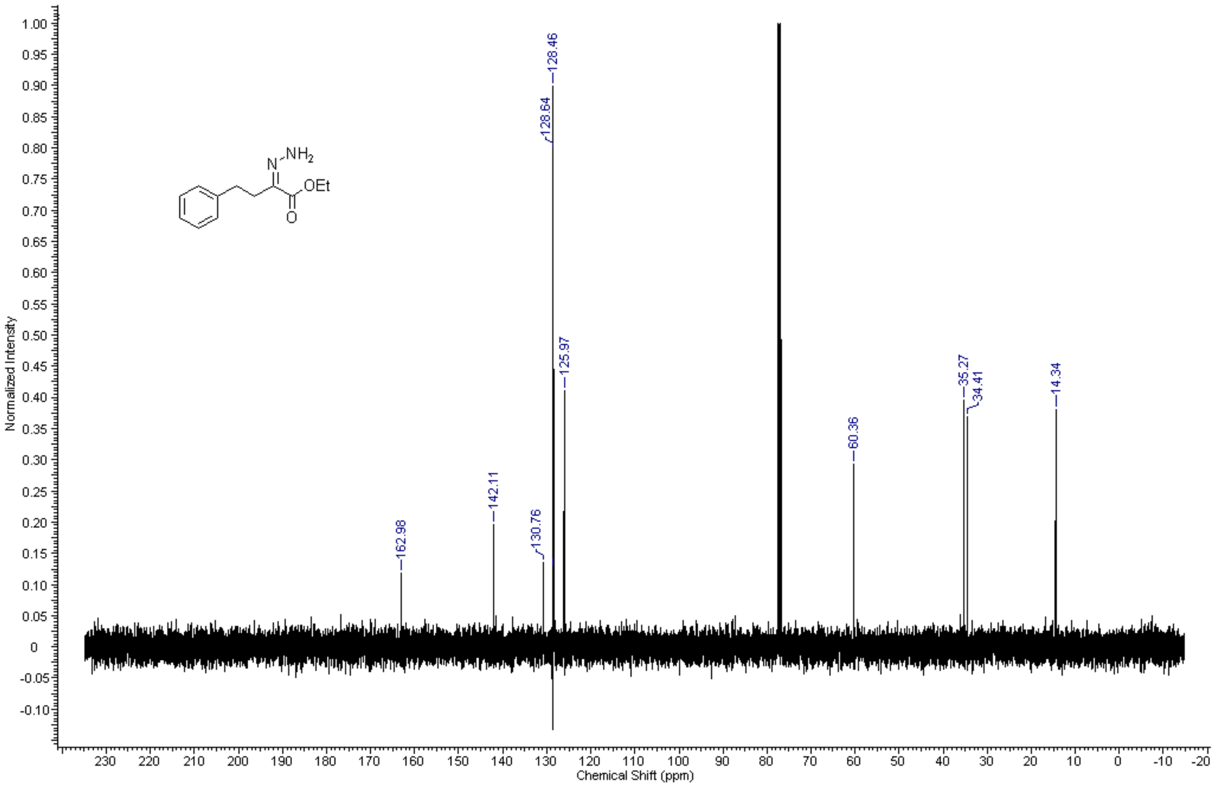

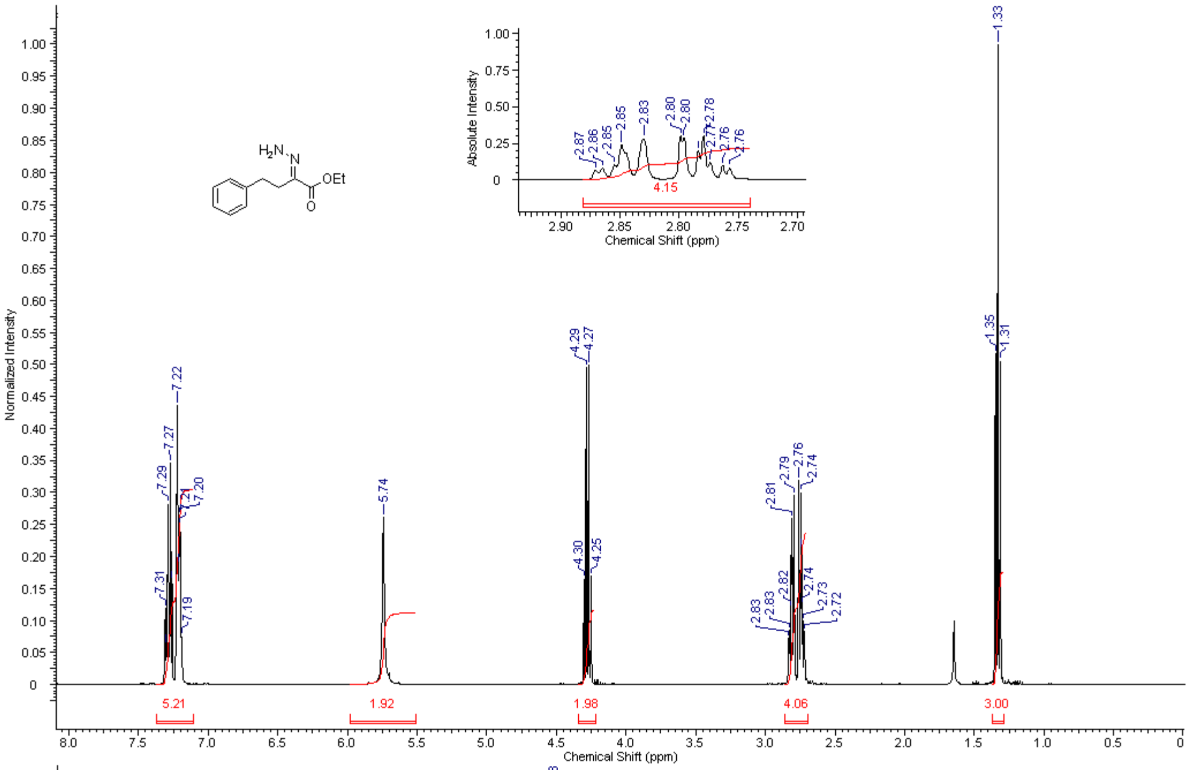

H2N
N
OEt
O
Normalized Intensity
Absolute Intensity
Chemical Shift (ppm)
4.15
5.21
1.92
1.98
4.06
3.00

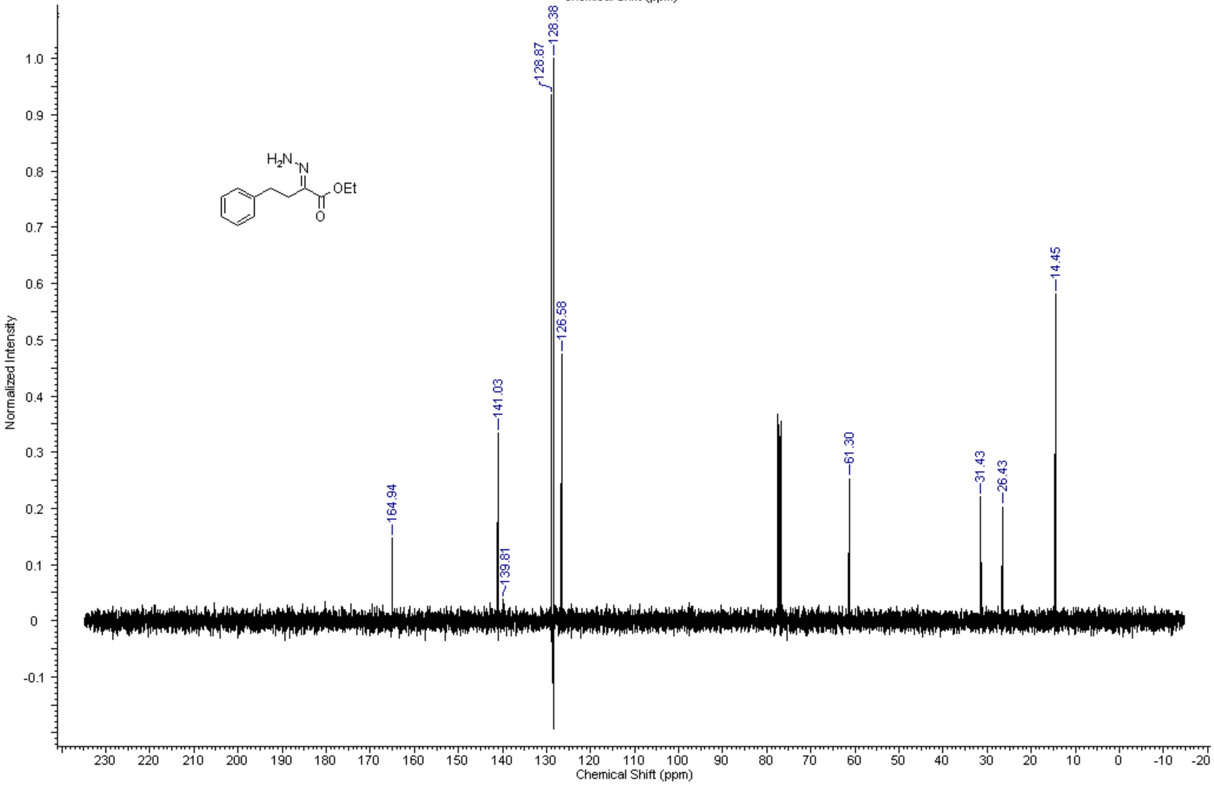

H2N
N
OEt
O
Normalized Intensity
Chemical Shift (ppm)
164.94
141.03
139.81
128.87
128.38
126.58
61.30
31.43
26.43
14.45

(*E*)- and (*Z*)-Allyl 2-hydrazono-2-phenylacetate 6h

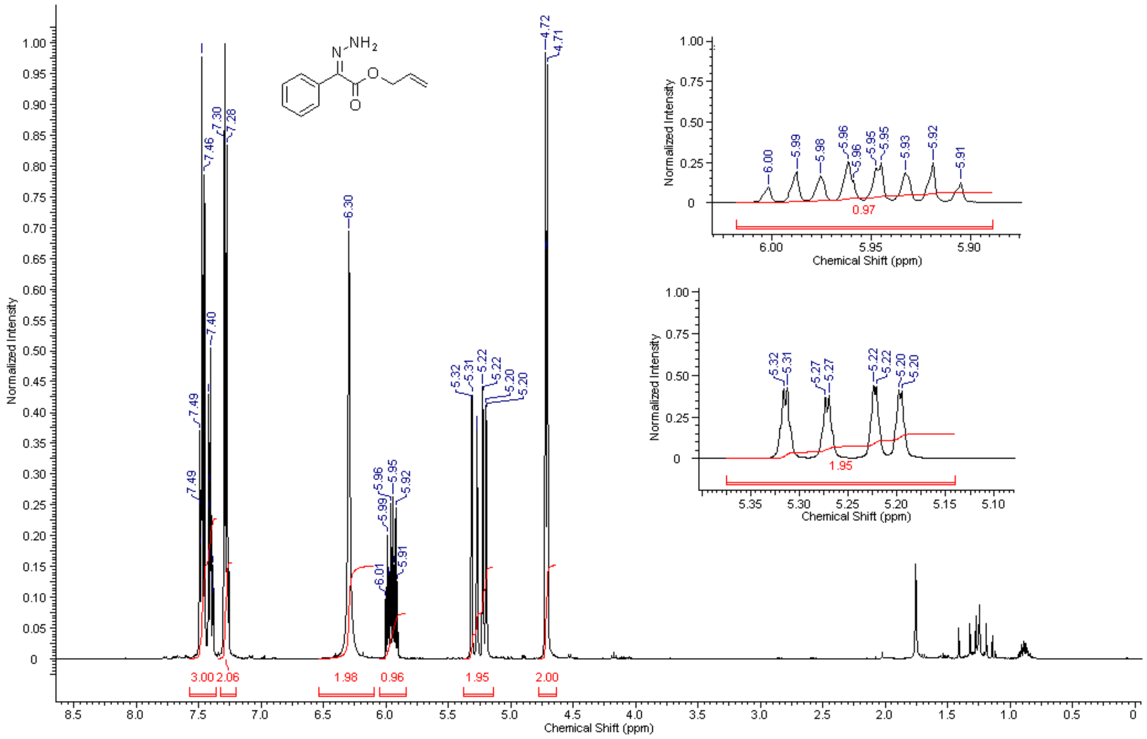

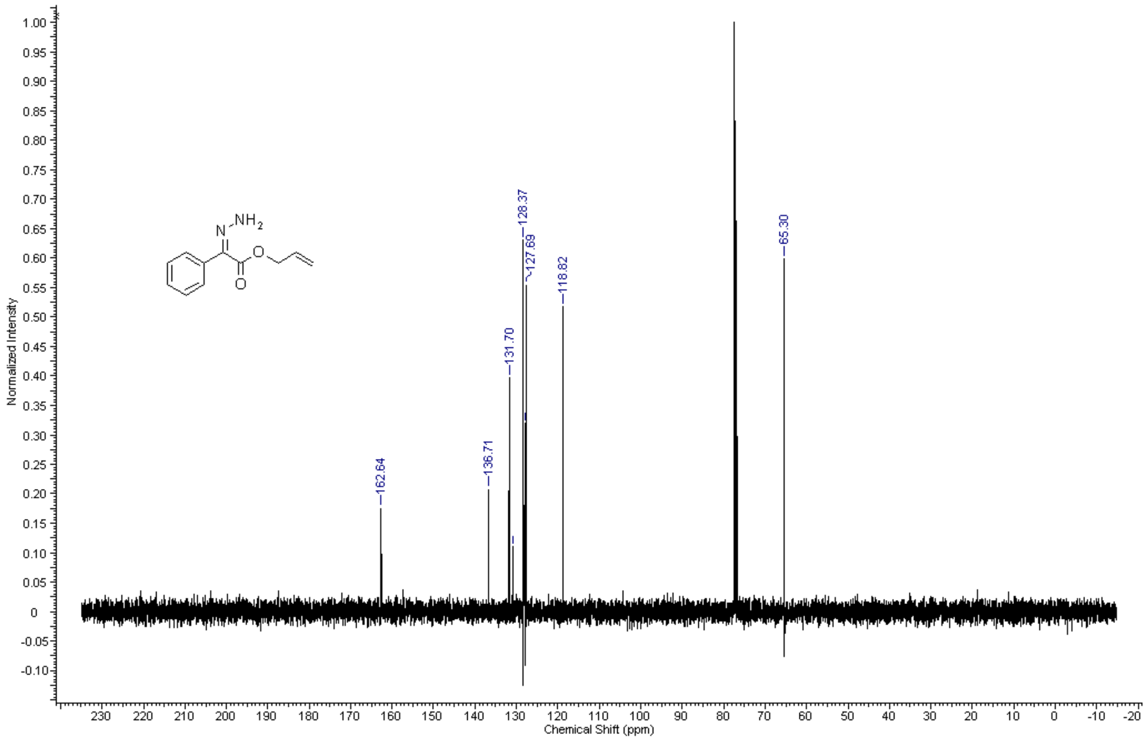

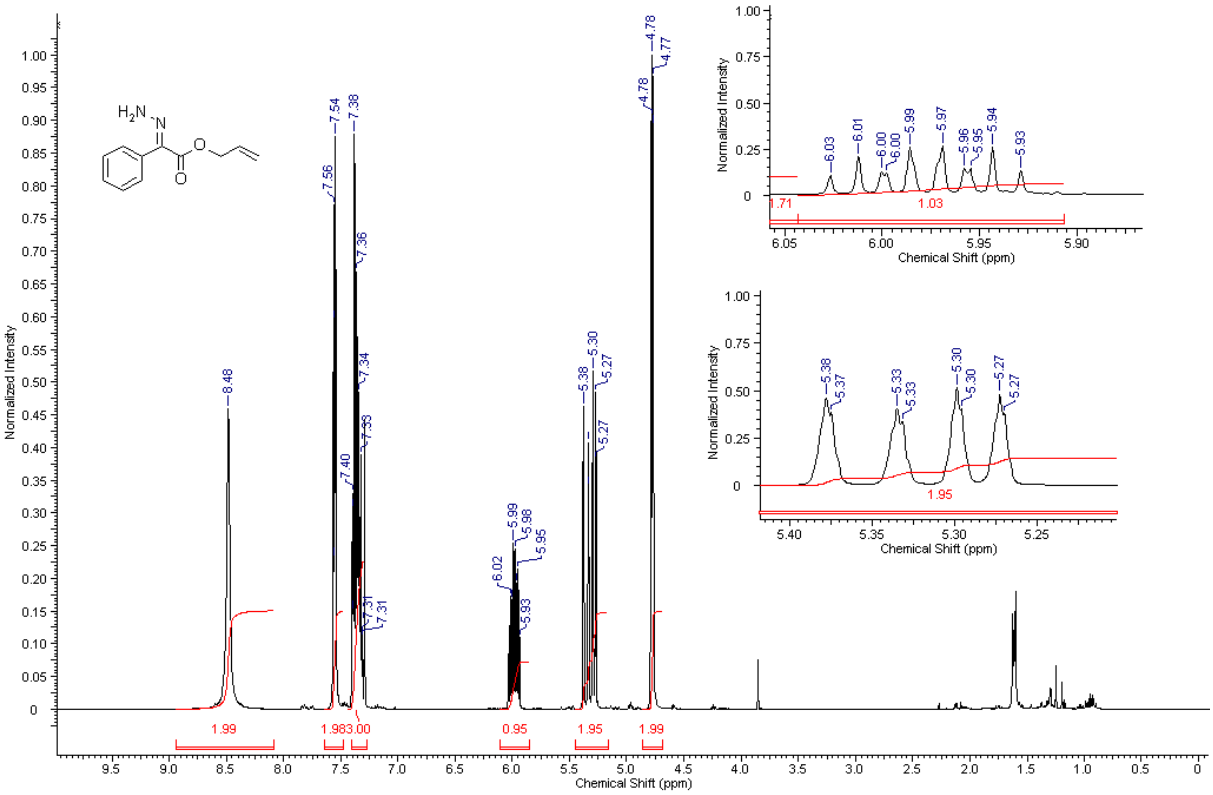

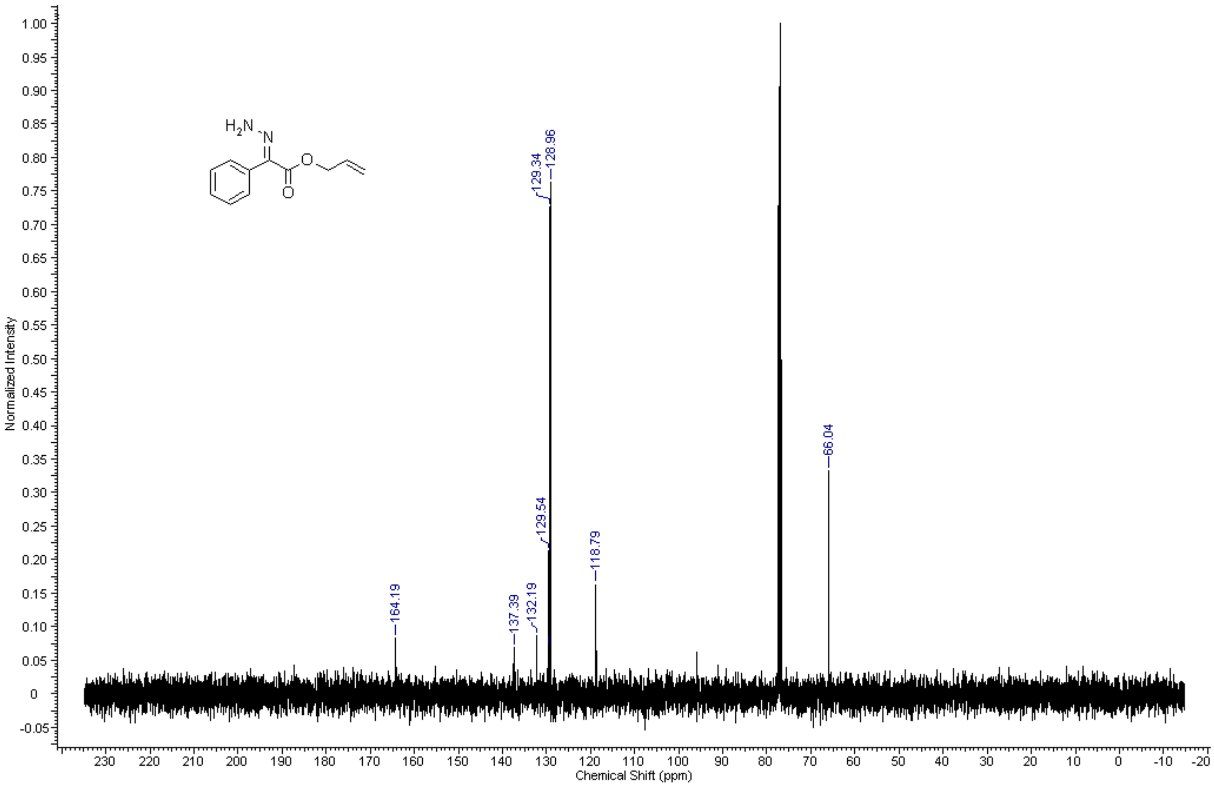

(*E*)- and (*Z*)-cyclohexyl 2-hydrazono-2-phenylacetate 6i

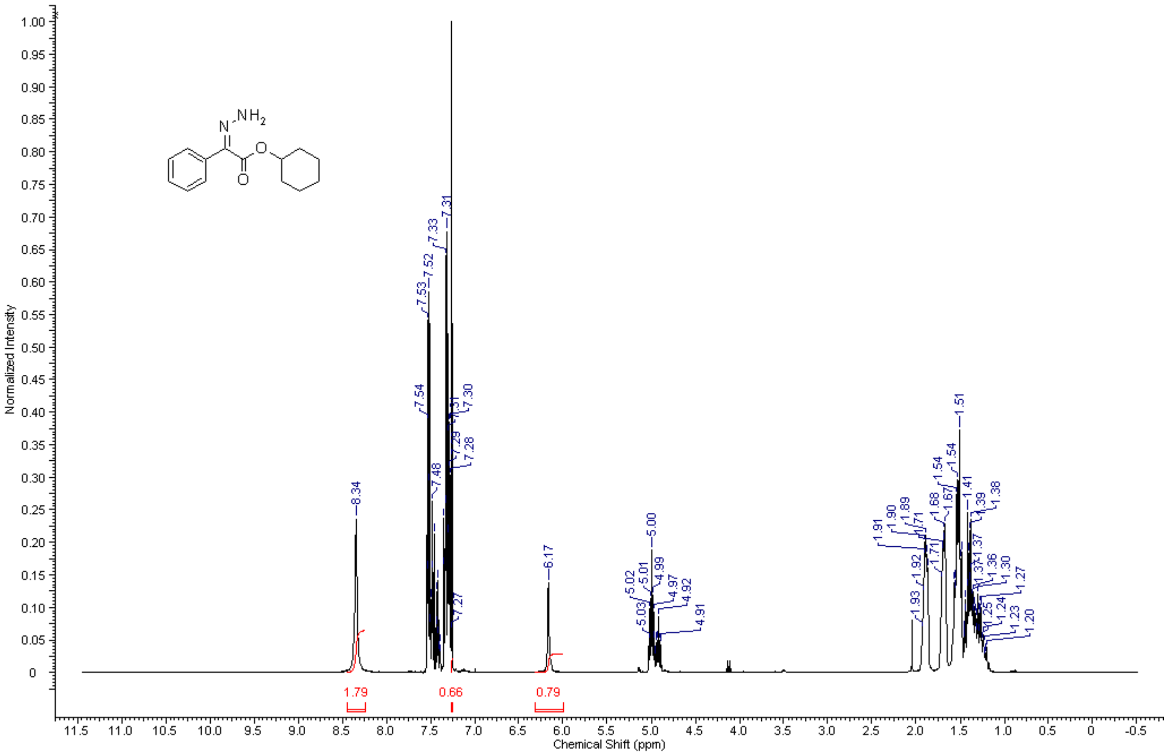

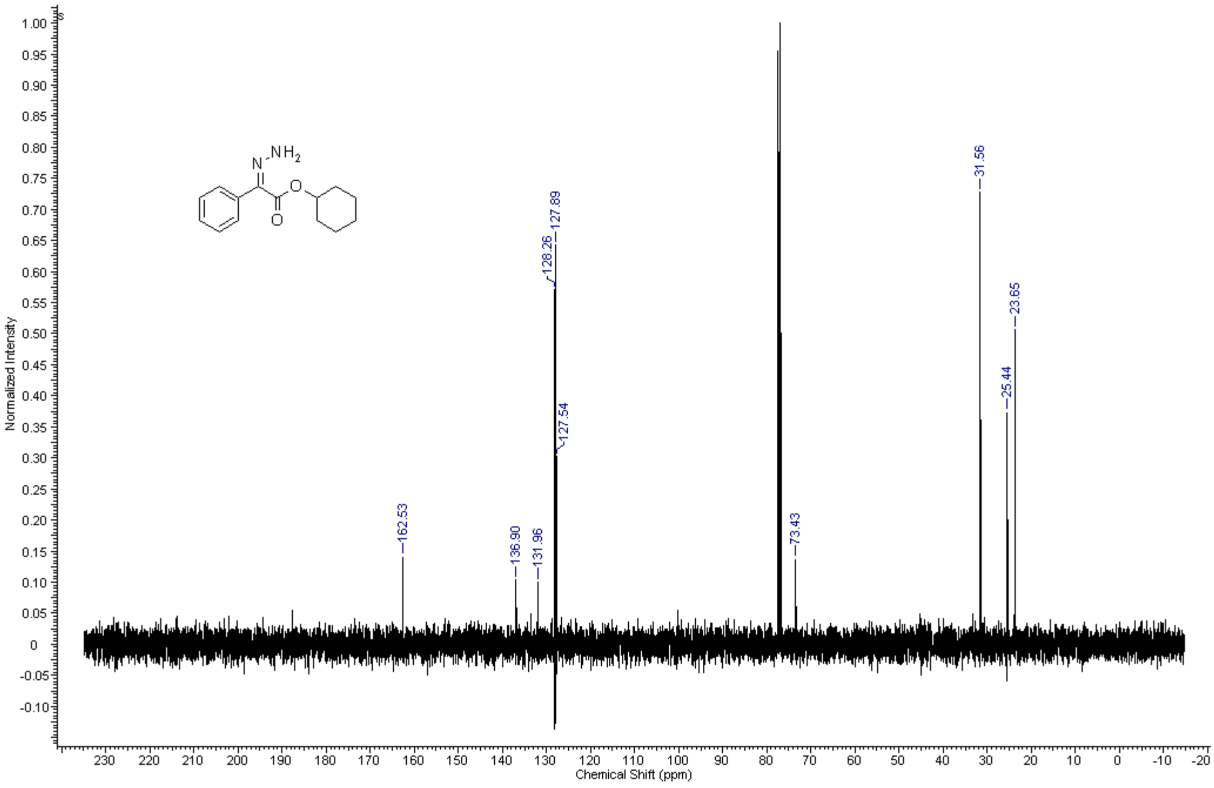

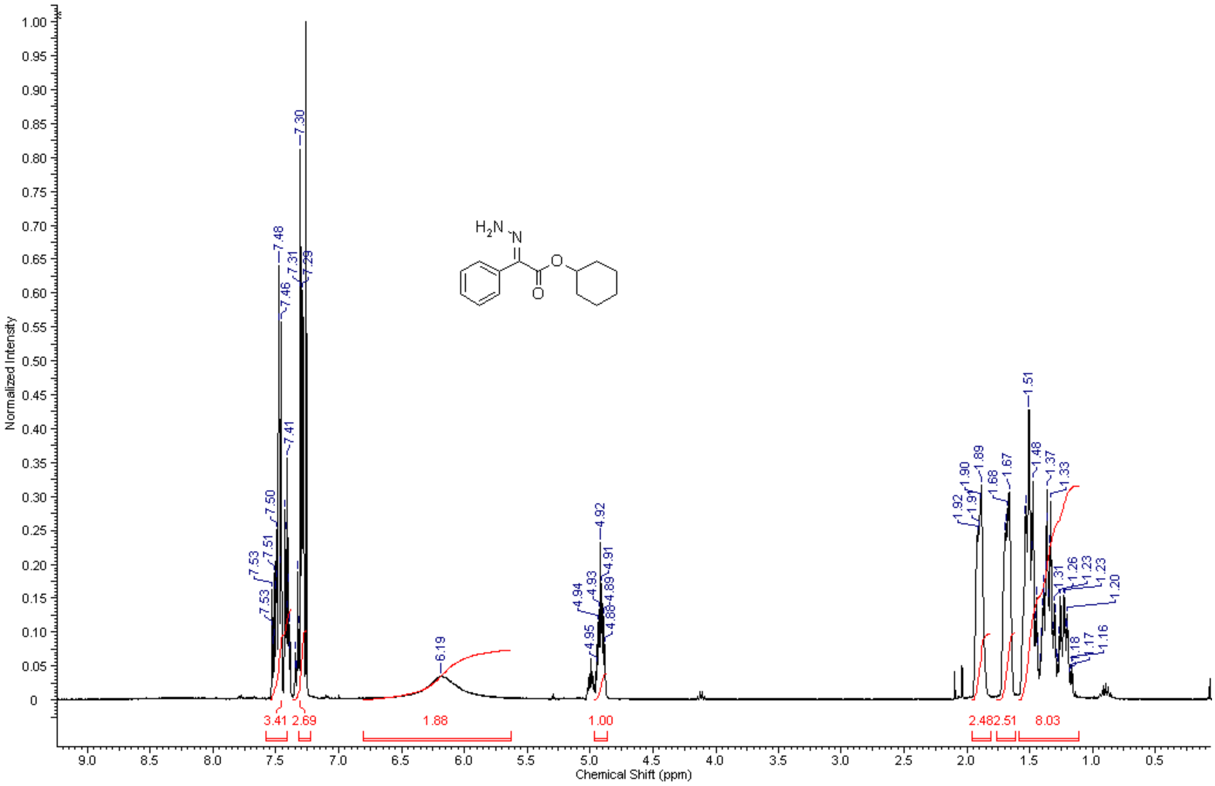

H_2N
N
O
O
Normalized Intensity
Chemical Shift (ppm)
7.30
7.48
7.46
7.31
7.29
7.41
7.50
7.51
7.53
7.53
6.19
4.92
4.91
4.94
4.93
4.89
4.88
4.95
1.51
1.48
1.37
1.33
1.90
1.89
1.92
1.91
1.68
1.67
1.31
1.26
1.23
1.23
1.20
1.18
1.17
1.16
3.41
2.69
1.88
1.00
2.48
2.51
8.03

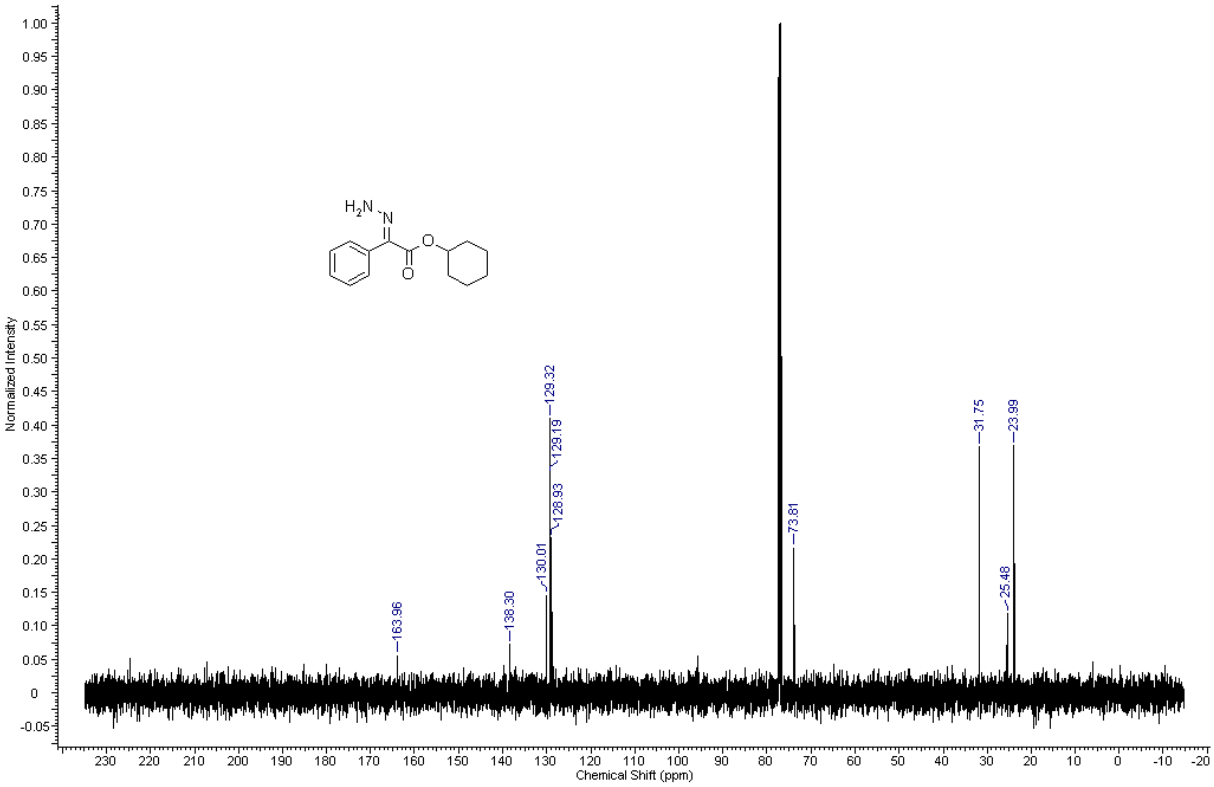

H_2N
N
O
O
Normalized Intensity
Chemical Shift (ppm)
163.96
138.30
130.01
129.32
129.19
128.93
73.81
31.75
25.48
23.99

(*E*)- and (*Z*)-3-Hydrazono-4,4-dimethyldihydrofuran-2(*3H*)-one 6j

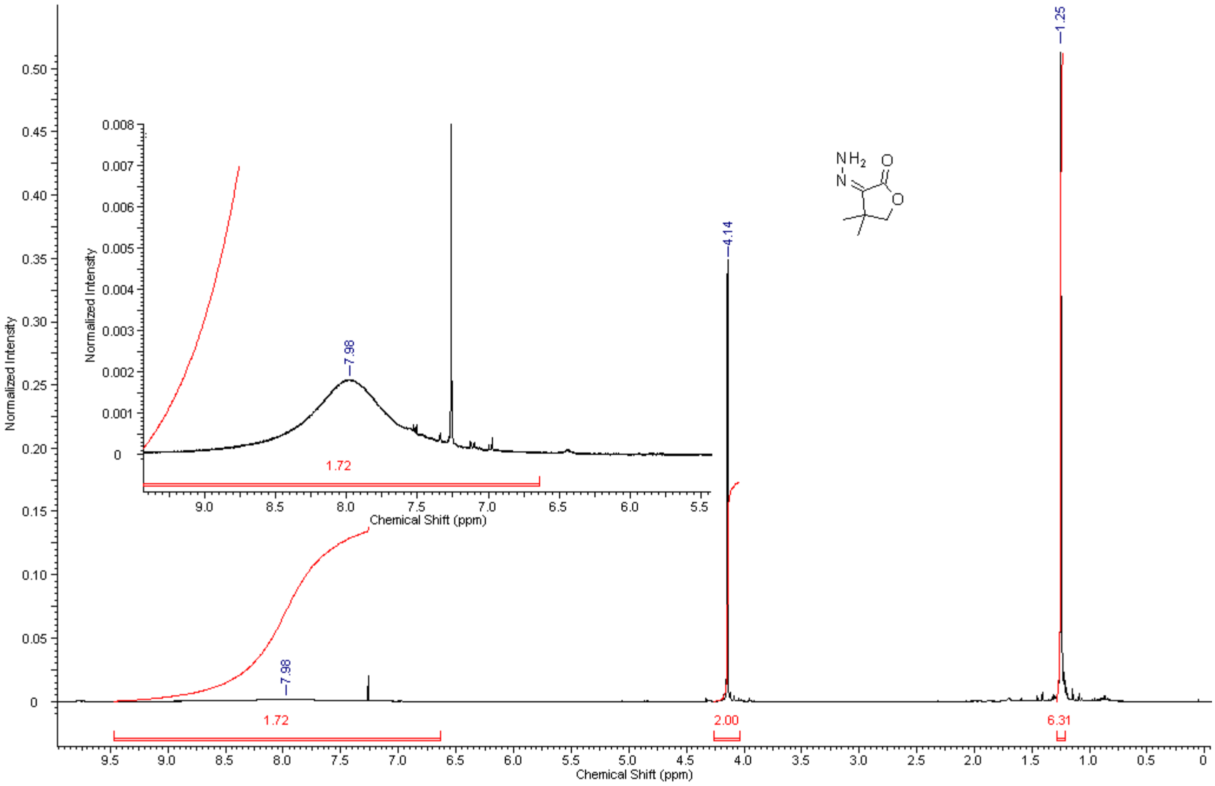

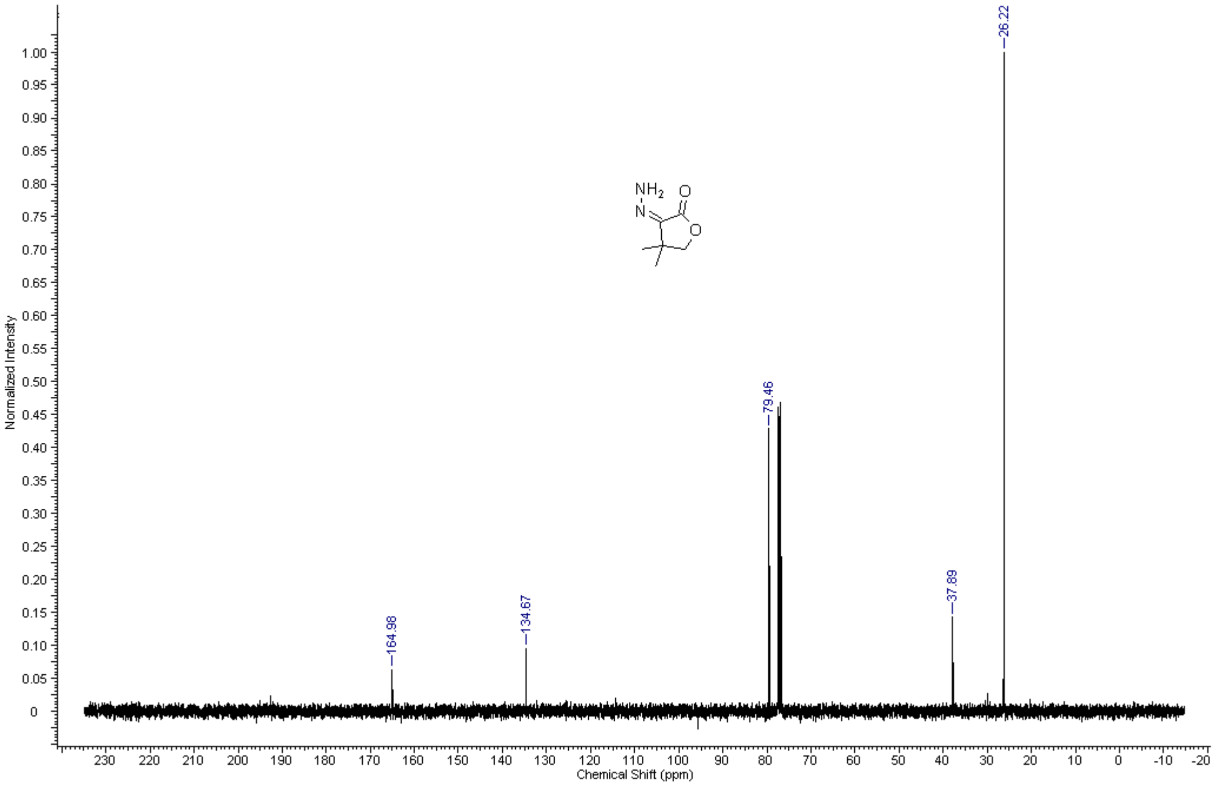

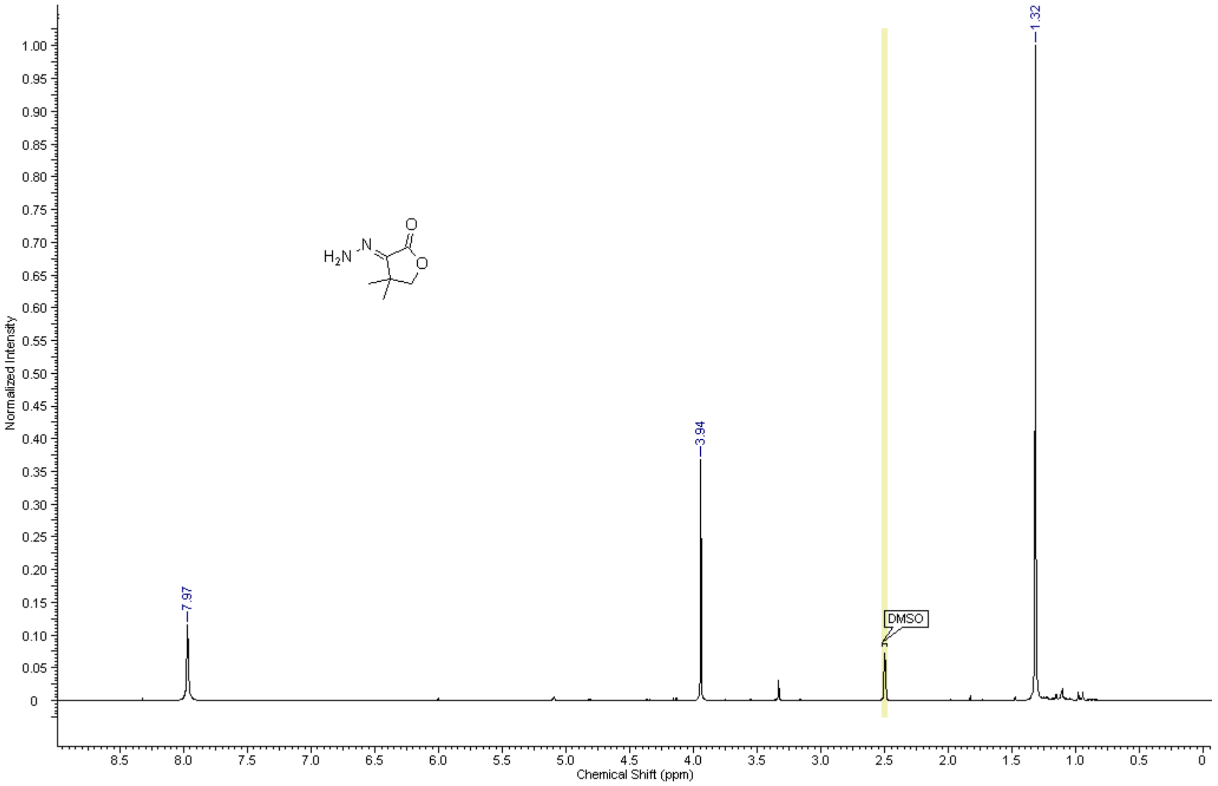
7.97
3.94
1.32
DMSO
Normalized Intensity
Chemical Shift (ppm)

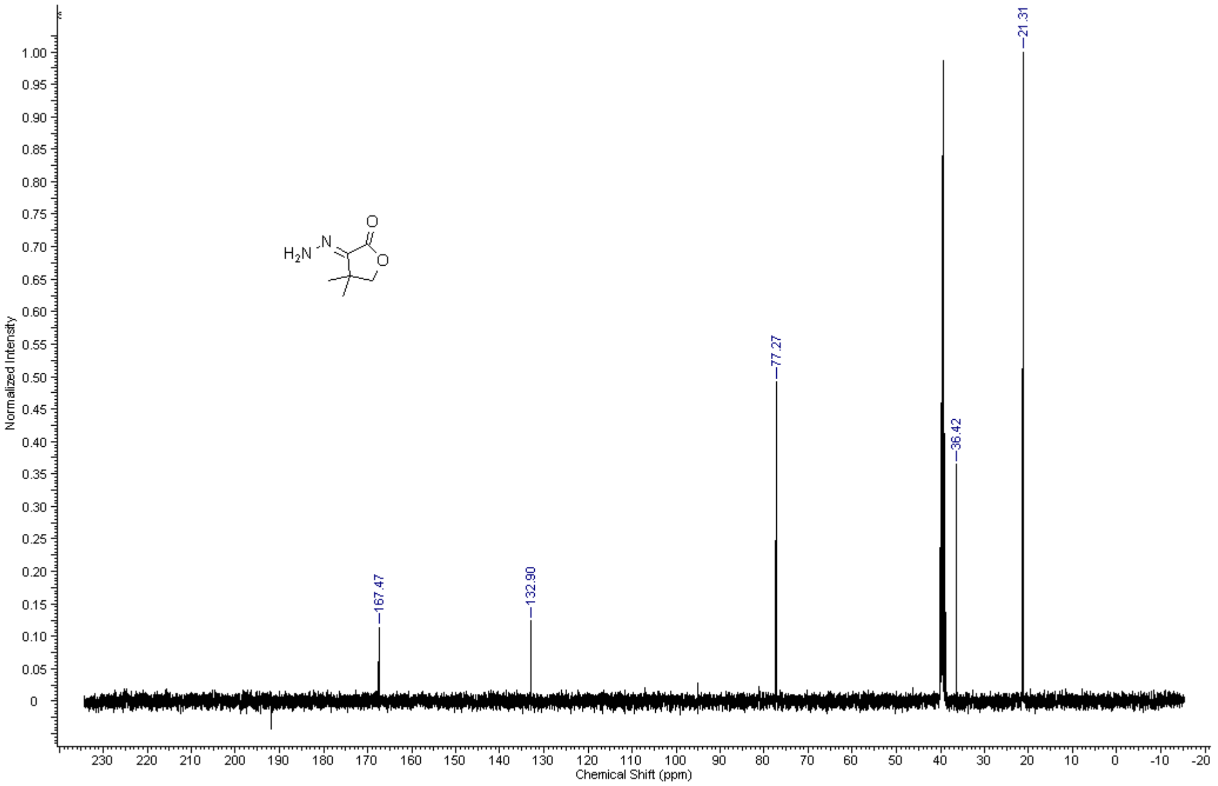
167.47
132.90
77.27
36.42
21.31
Normalized Intensity
Chemical Shift (ppm)

Mixture of (*E*) and (*Z*)-3-hydrazonoindolin-2-one 6k

(initial ratio: E/Z = 89/11, isomerisation occurred upon standing in favour of the (*Z*)- isomer)

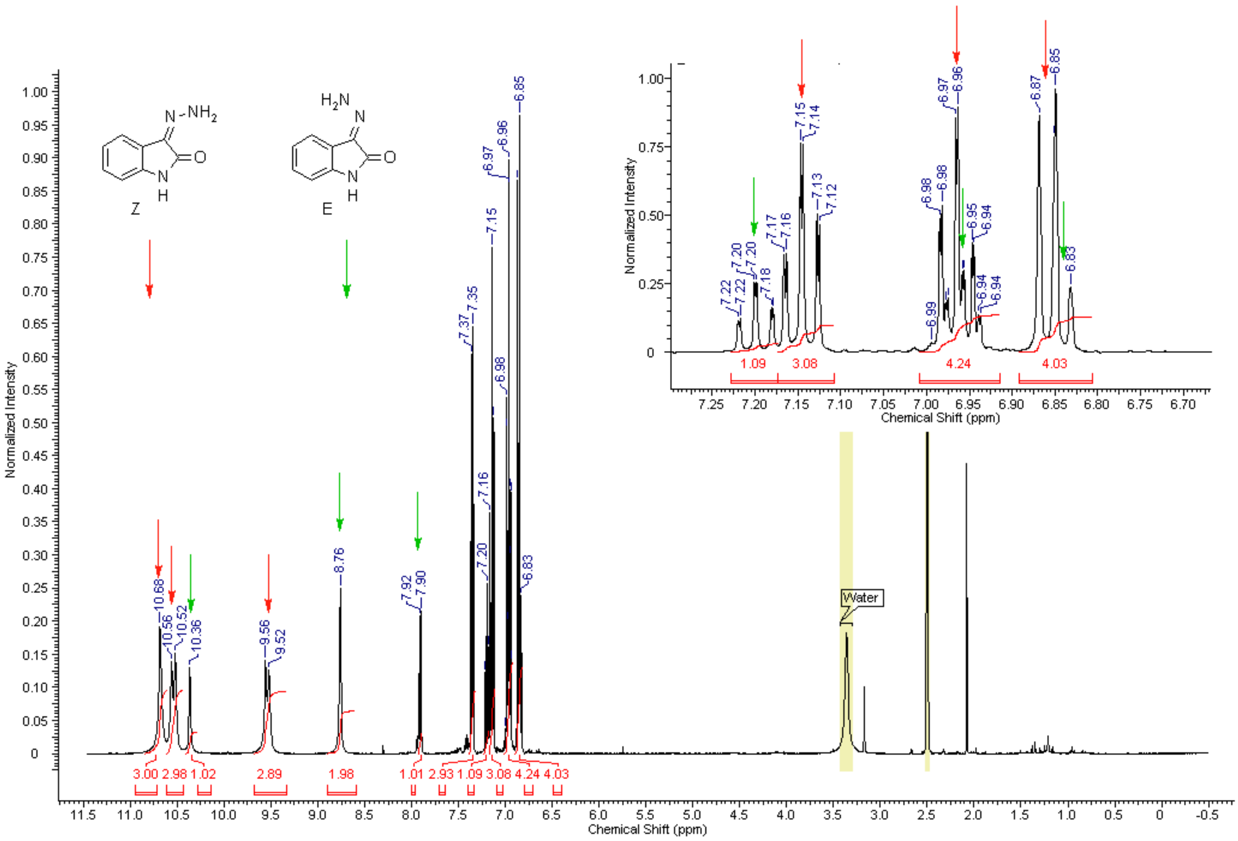

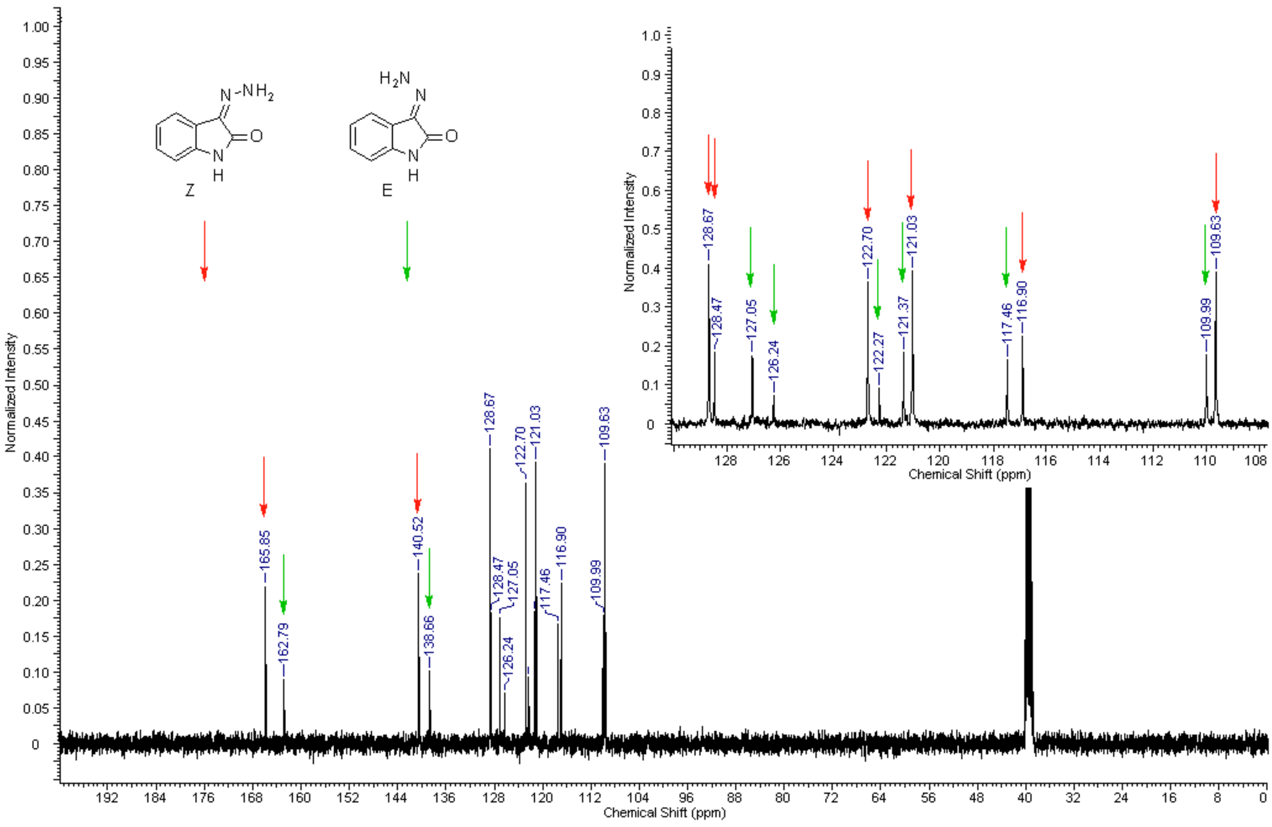

Mixture of 3-hydrazono-1-methylindolin-2-one 6l

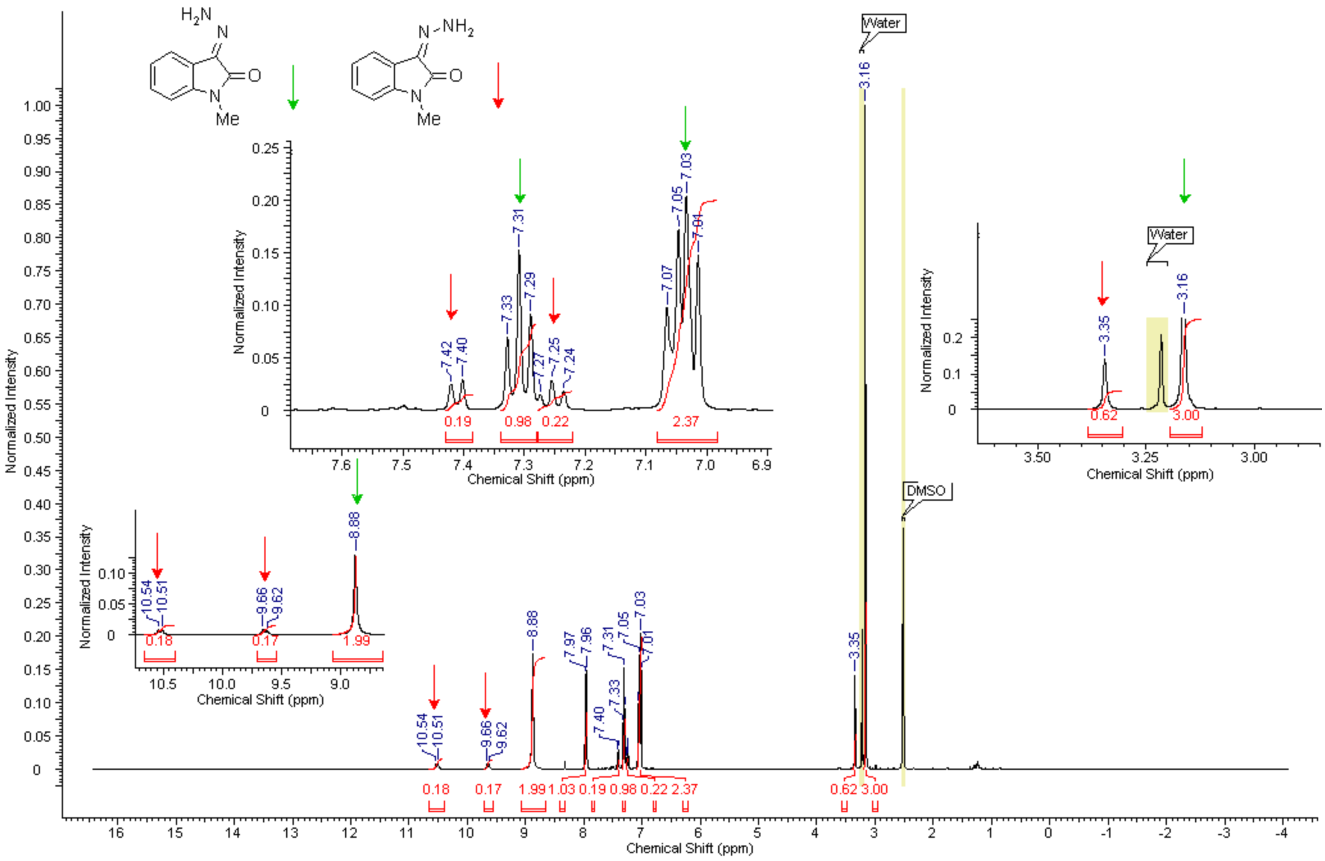

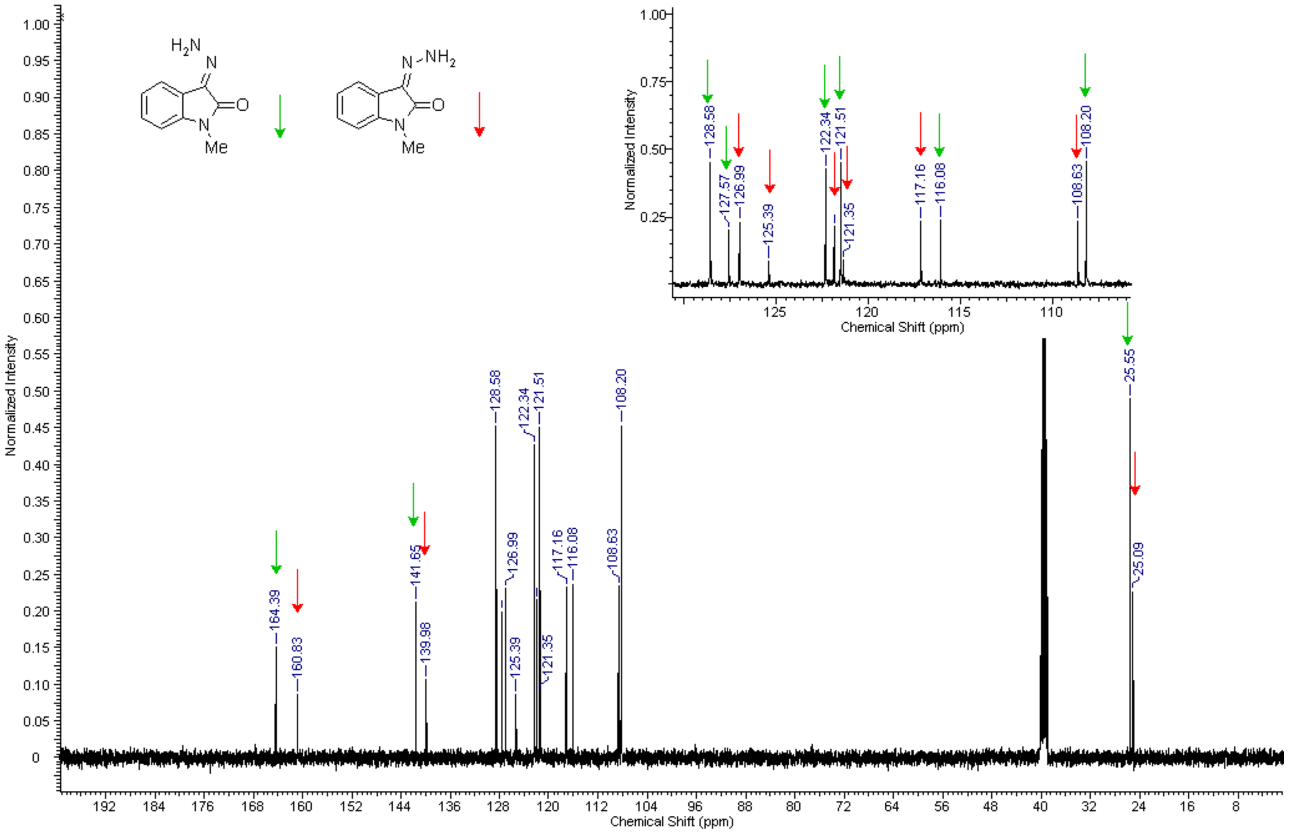

(*E*)- and (*Z*)-Ethyl 2-hydrazono-2-(thienyl)acetate 6m

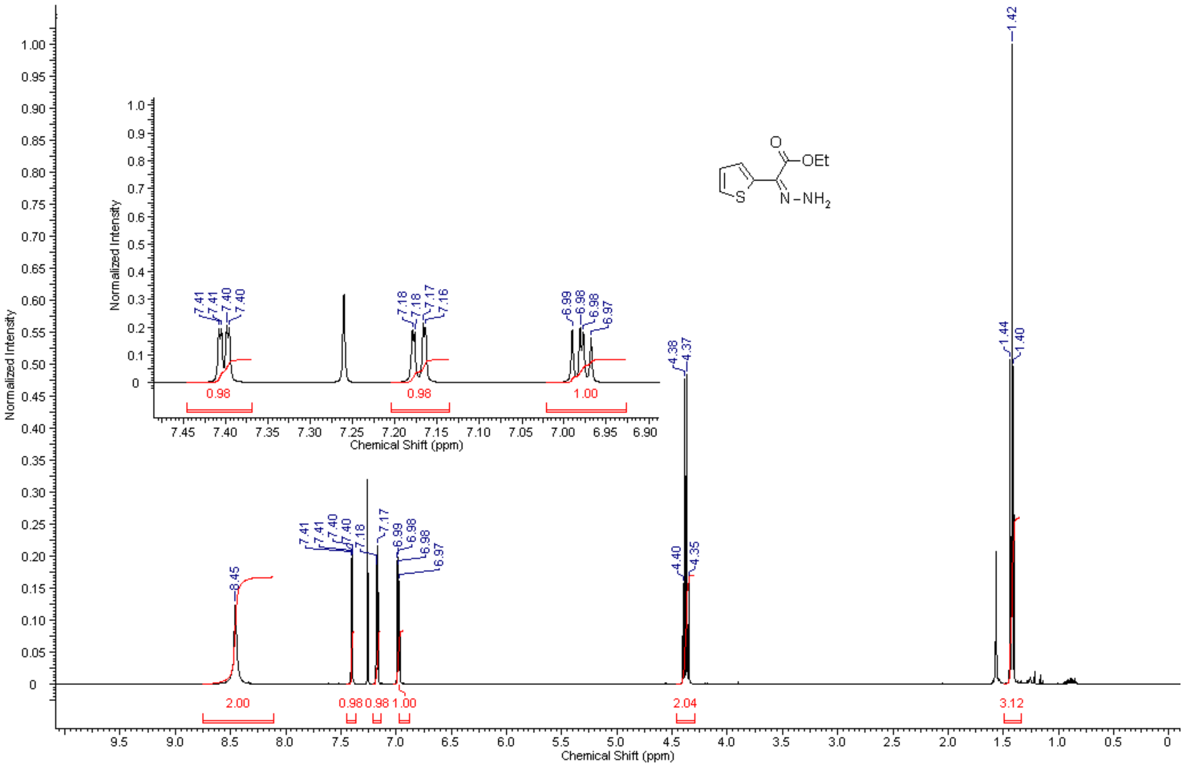

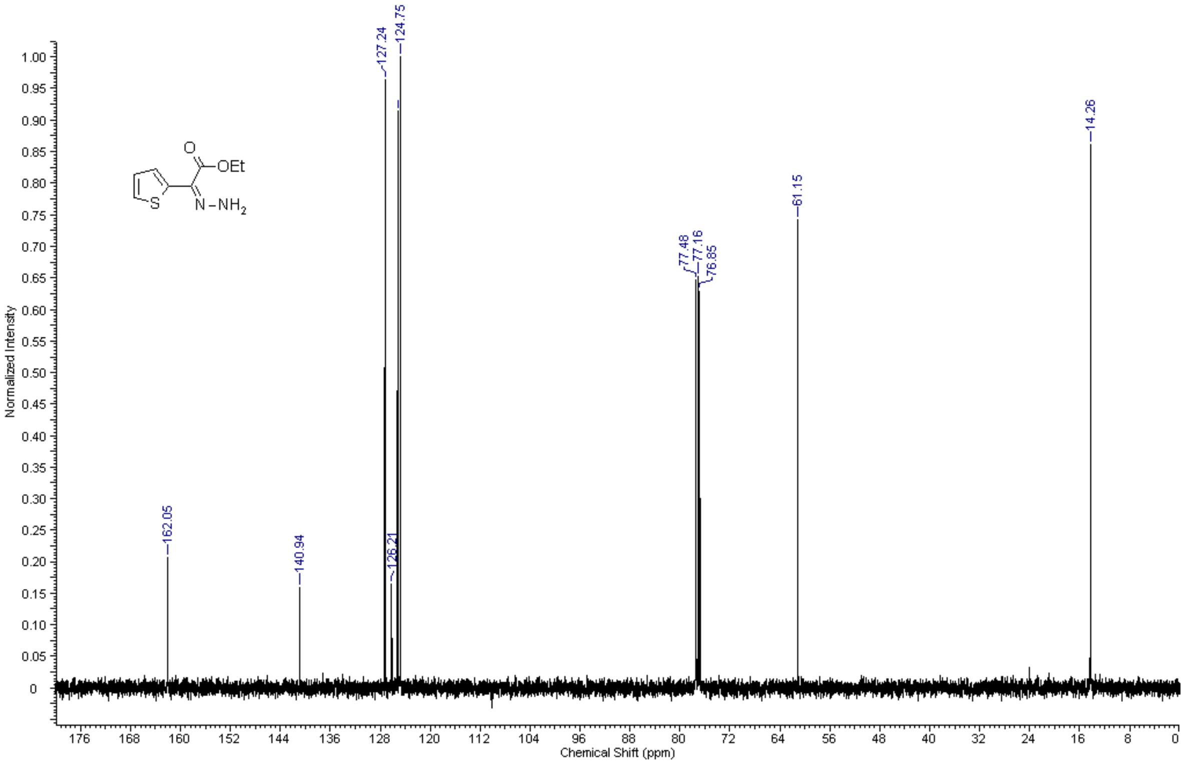

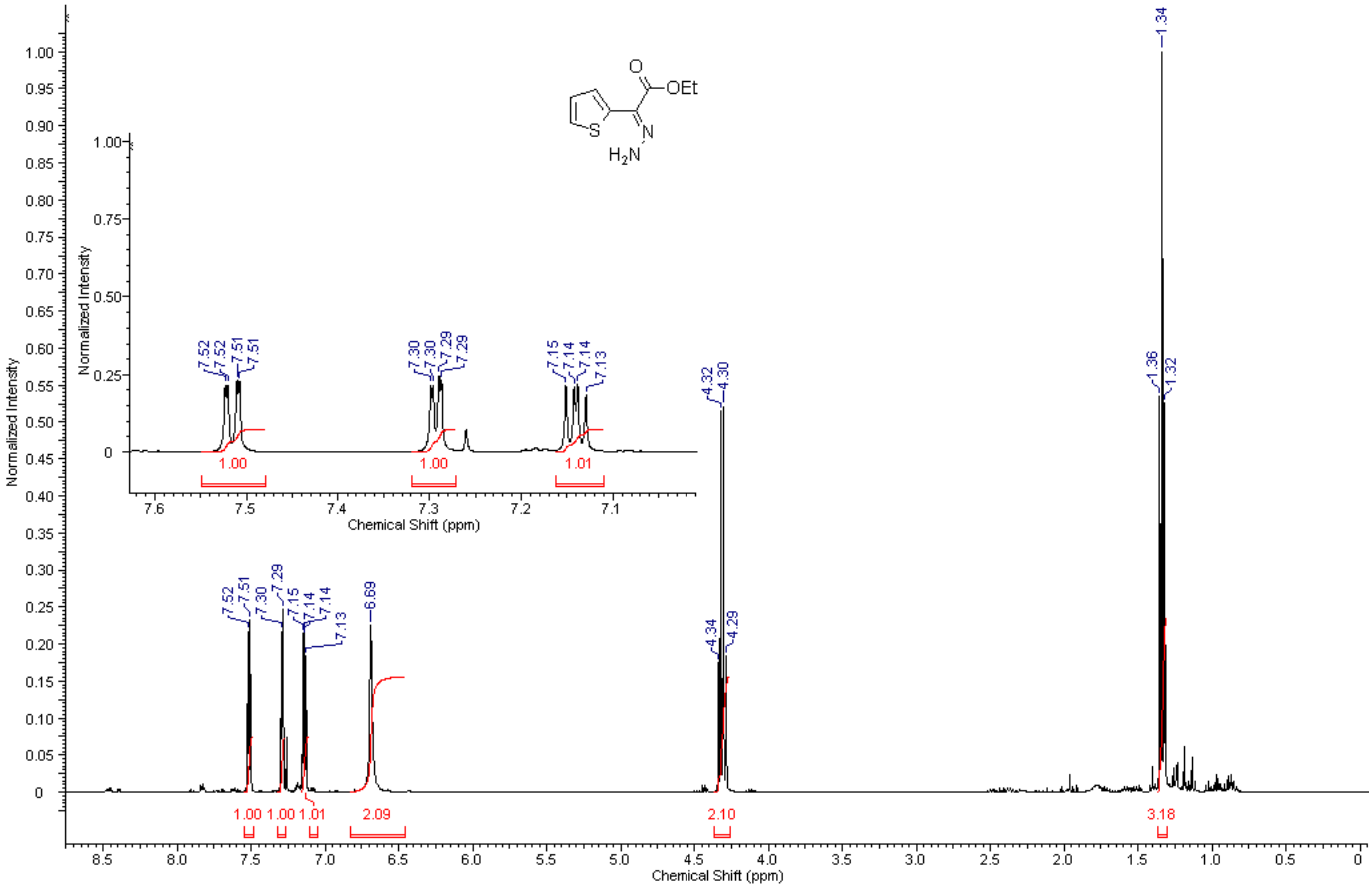

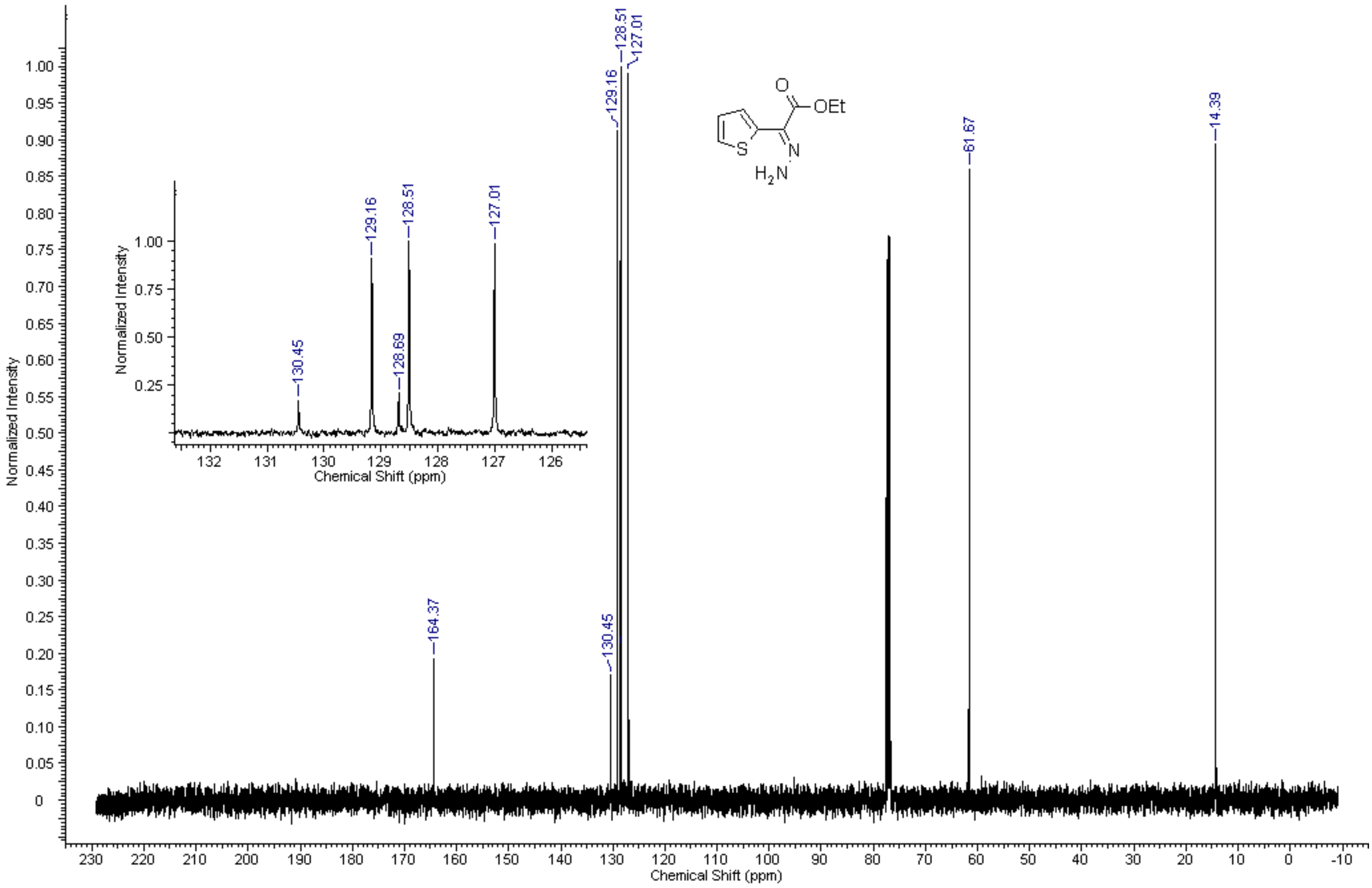

Ethyl 2-hydrazono-2-(pyridin-2-yl)acetate 6n

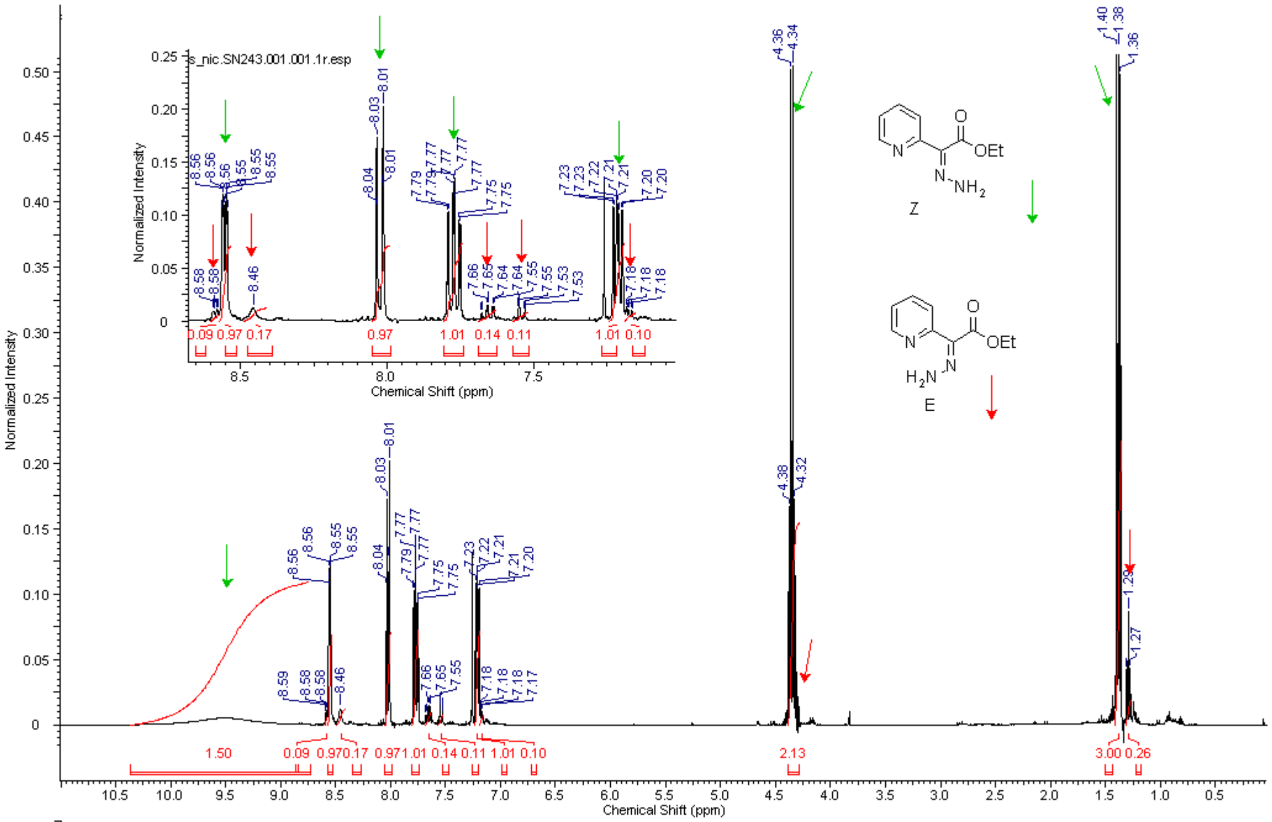

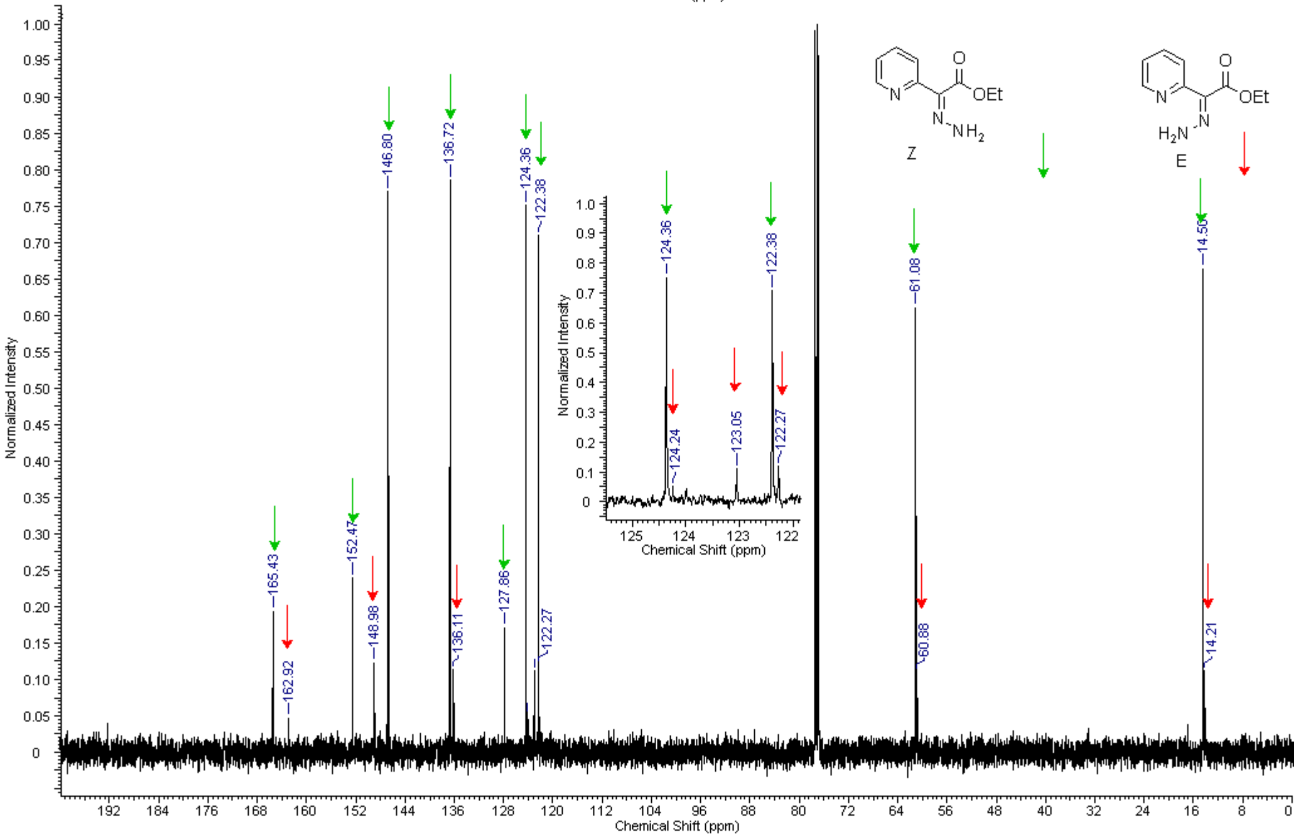

(*E*)- and (*Z*)-*tert*-Butyl 3-(1-hydrazono-2-methoxy-2-oxoethyl)-1H-indole-1-carboxylate 6o

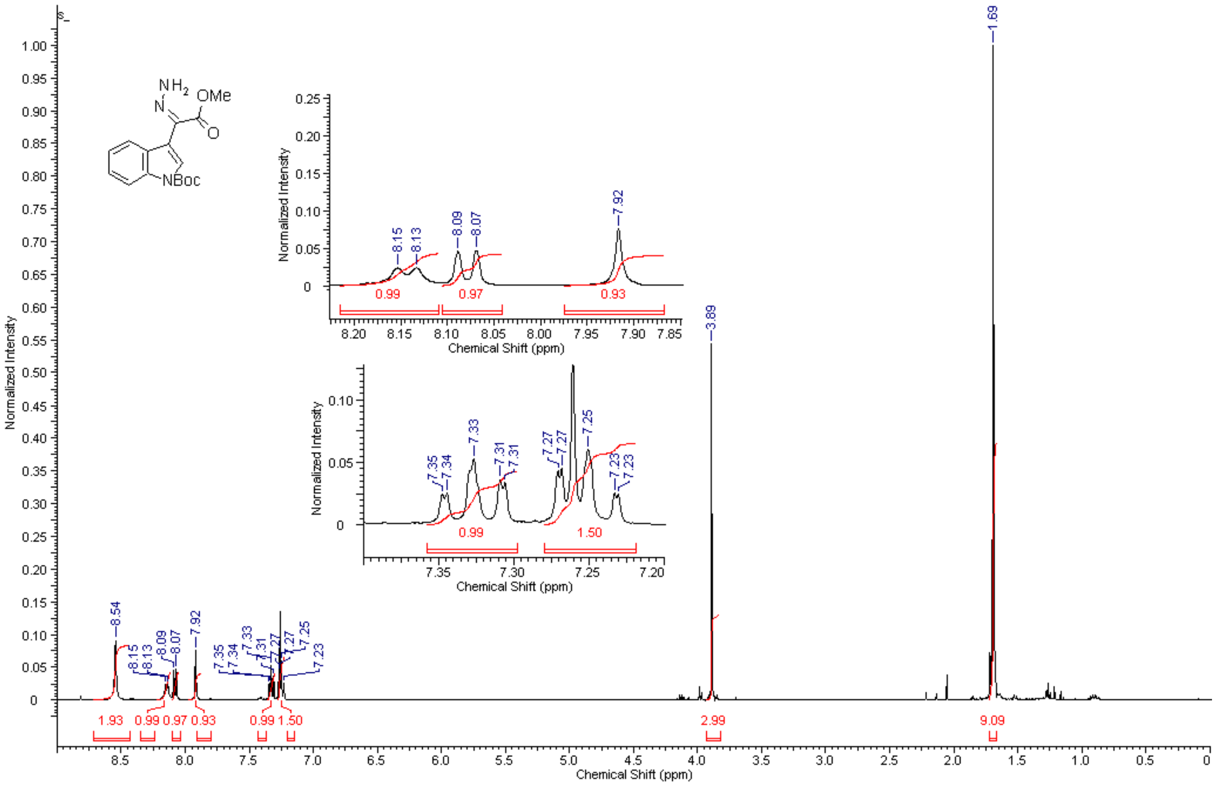

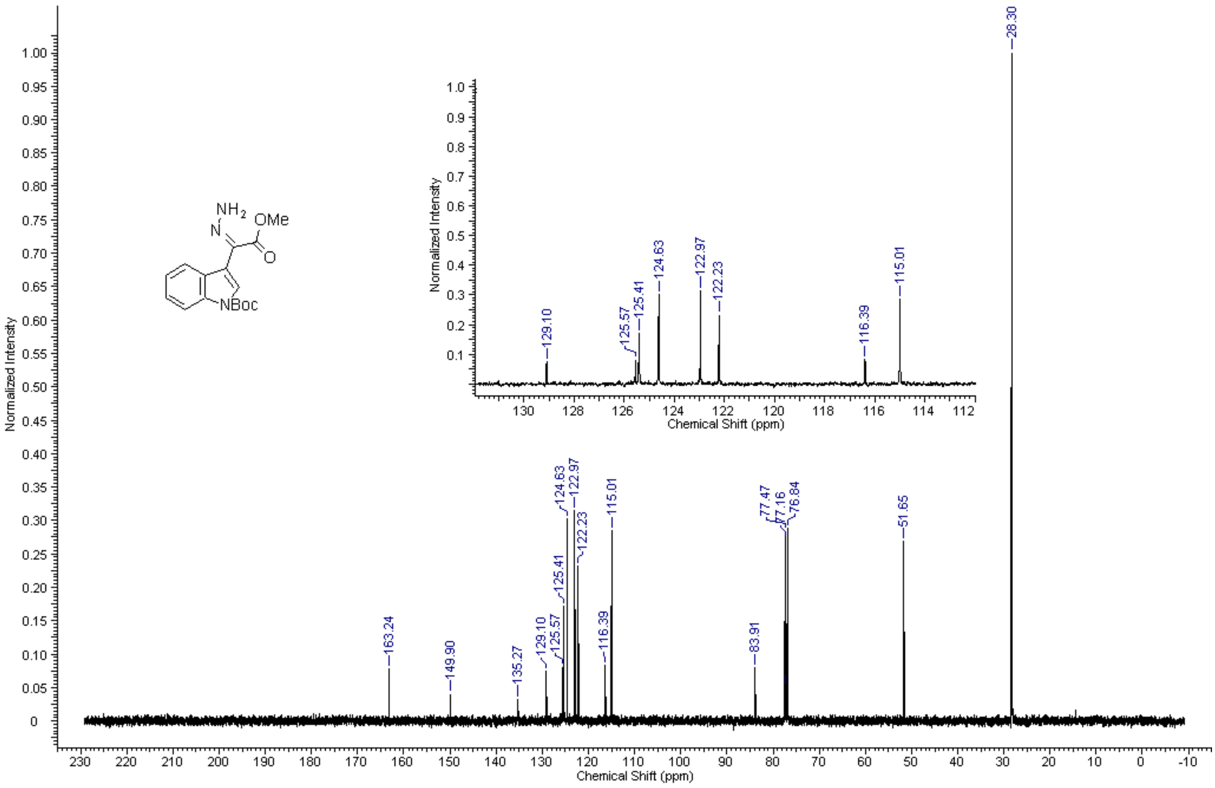

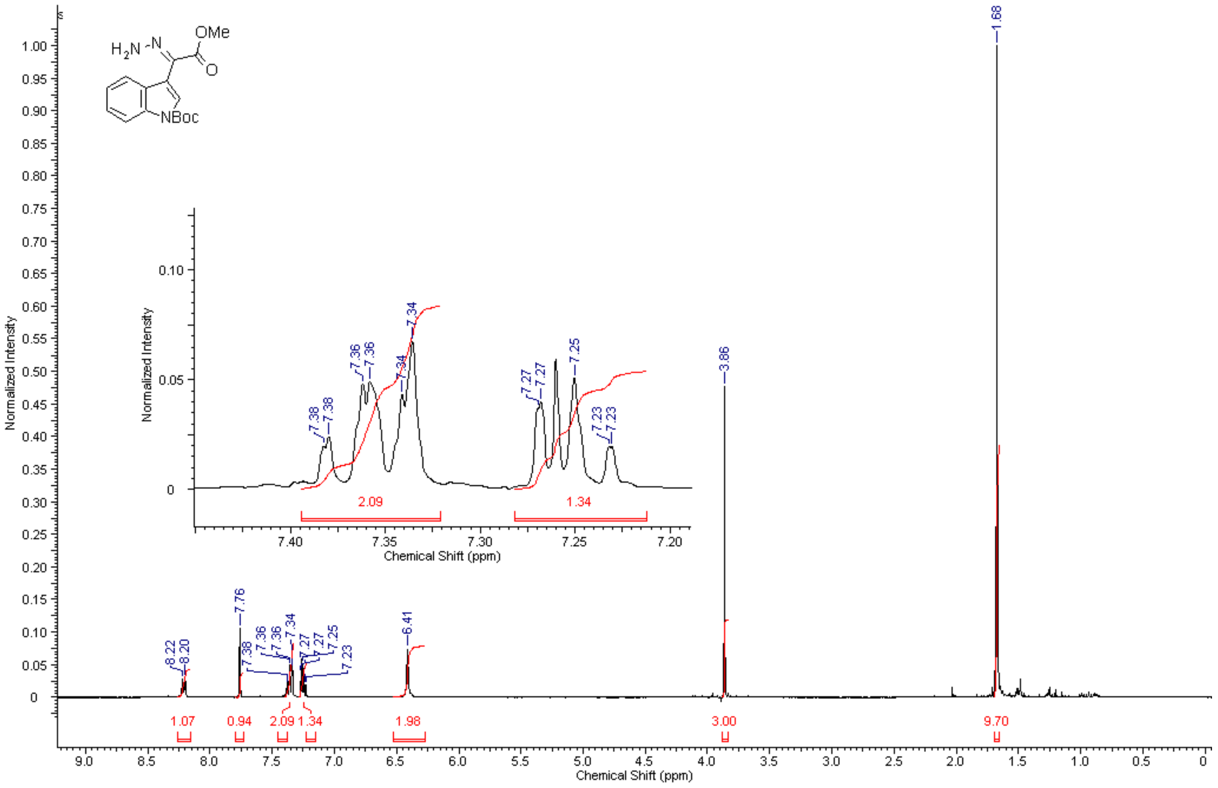
H2N-N
OMe
O
NBoc
Normalized Intensity
Chemical Shift (ppm)
2.09
1.34
1.07
0.94
3.00
1.98
9.70
1.68
3.86
6.41
7.76
8.22
8.20

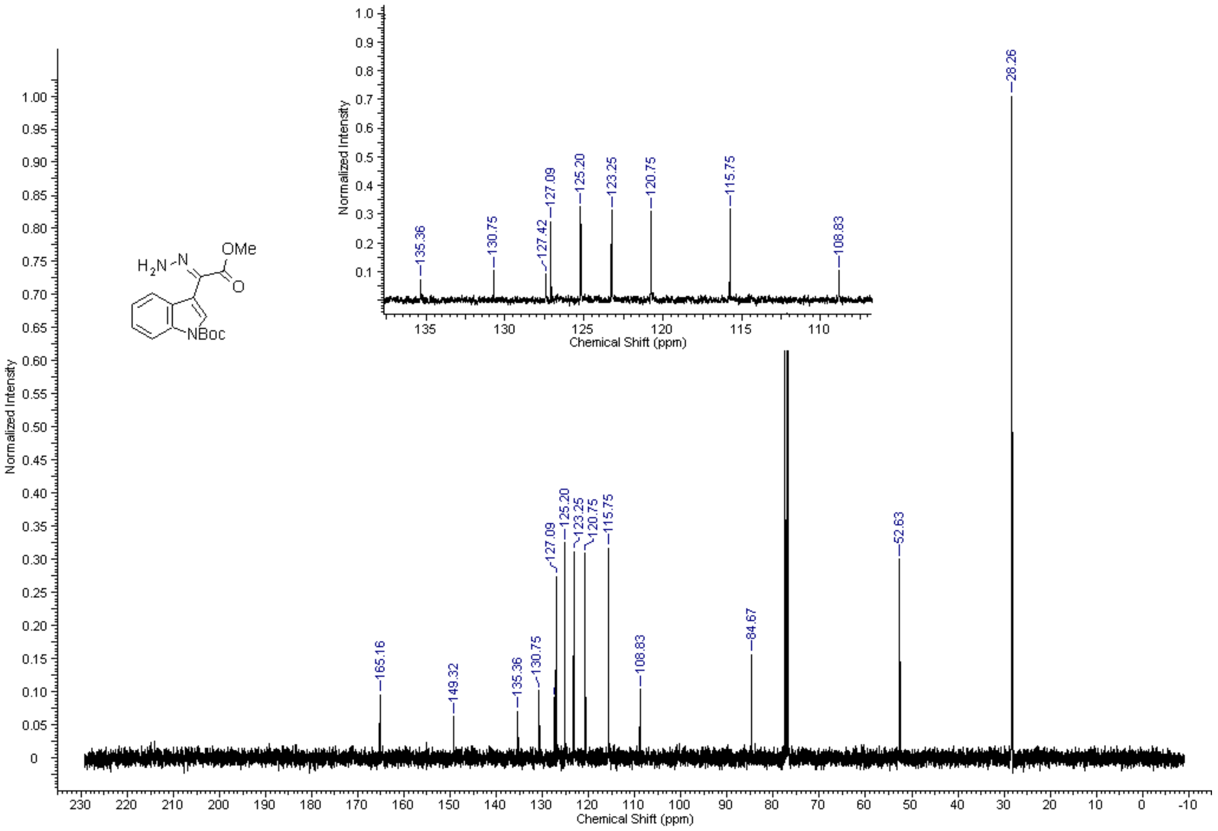
H2N-N
OMe
O
NBoc
Normalized Intensity
Chemical Shift (ppm)
165.16
149.32
135.36
130.75
127.42
127.09
125.20
123.25
120.75
115.75
108.83
84.67
52.63
28.26

(*E*)- and (*Z*)-*N*-Allyl-2-hydrazono-2-phenylacetamide 6p

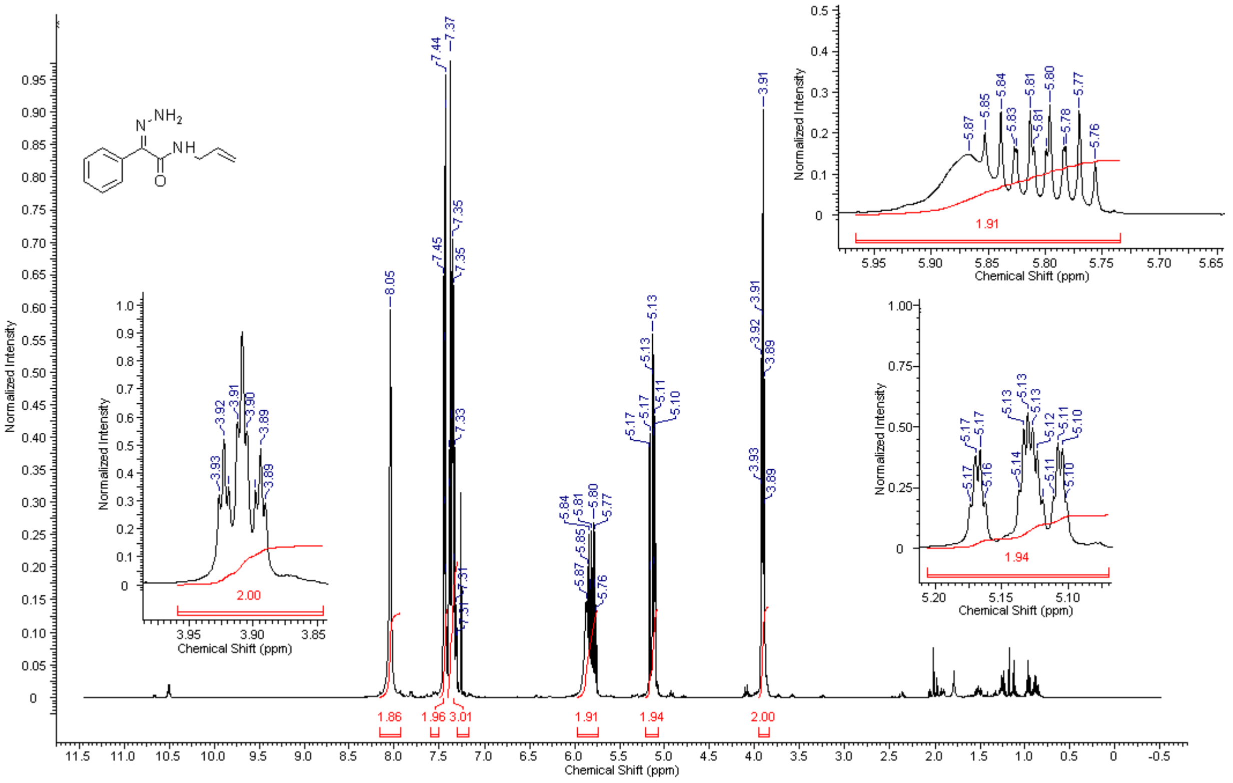

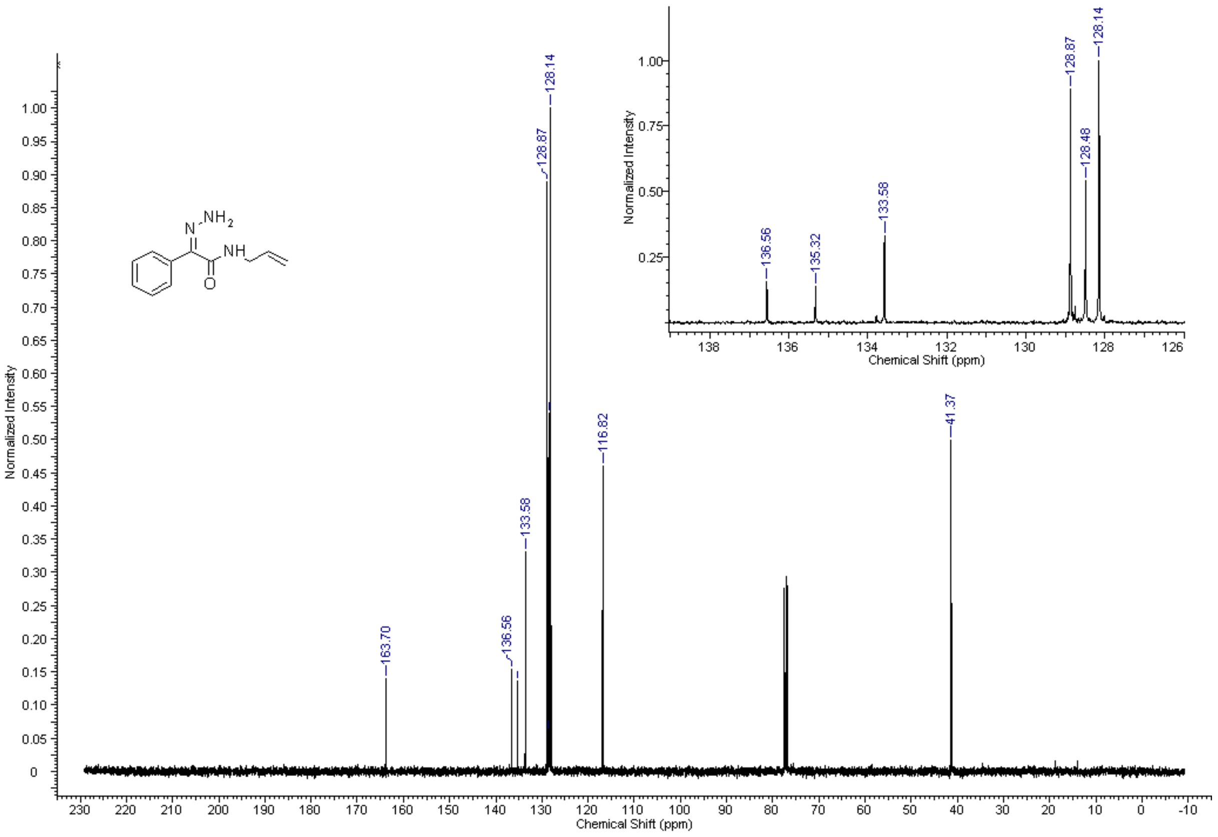

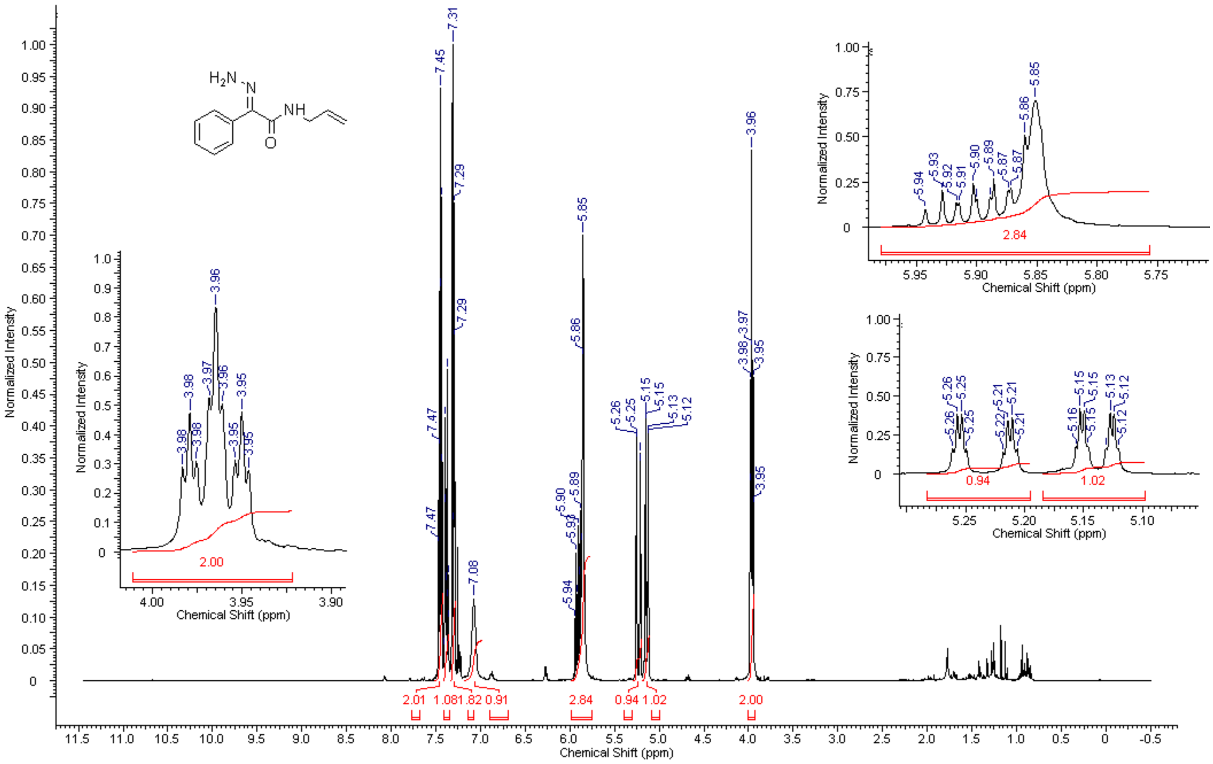

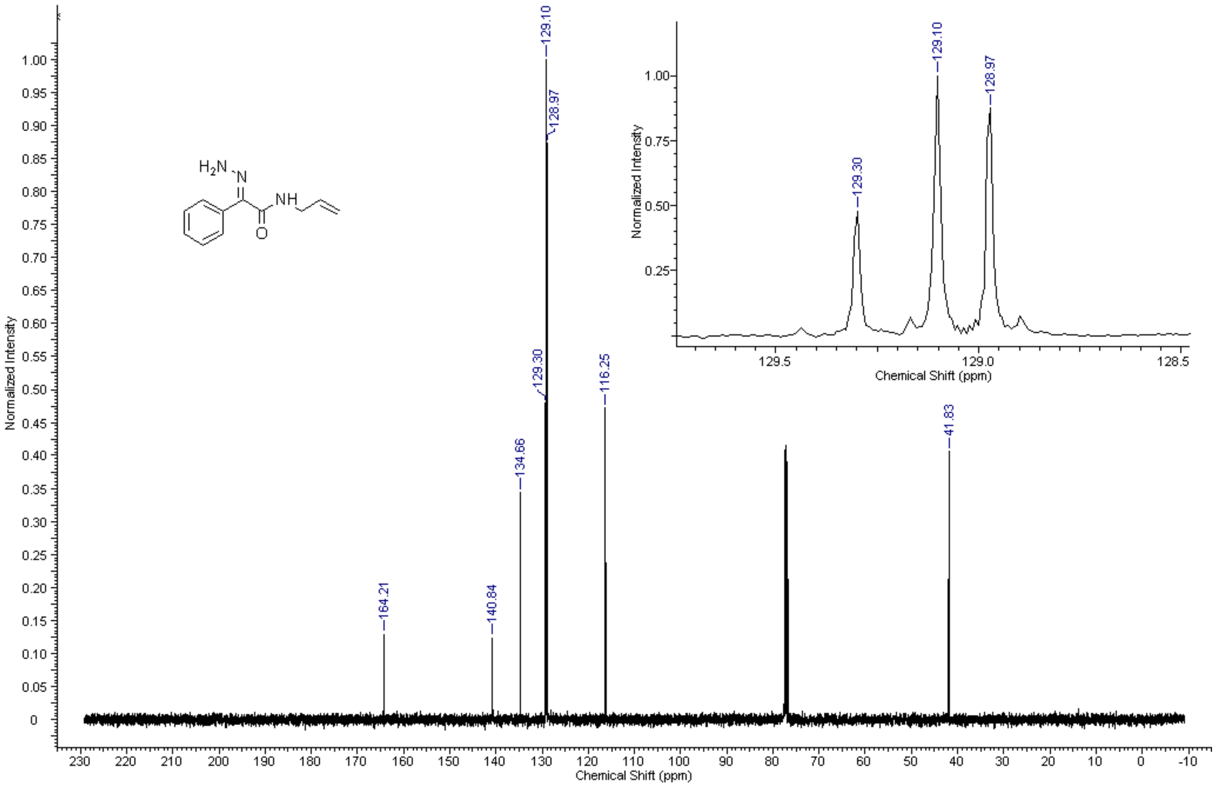

(*E*)- and (*Z*)-*N*-(2,5-Dimethoxyphenyl)-2-hydrazono-2-phenylacetamide 6q

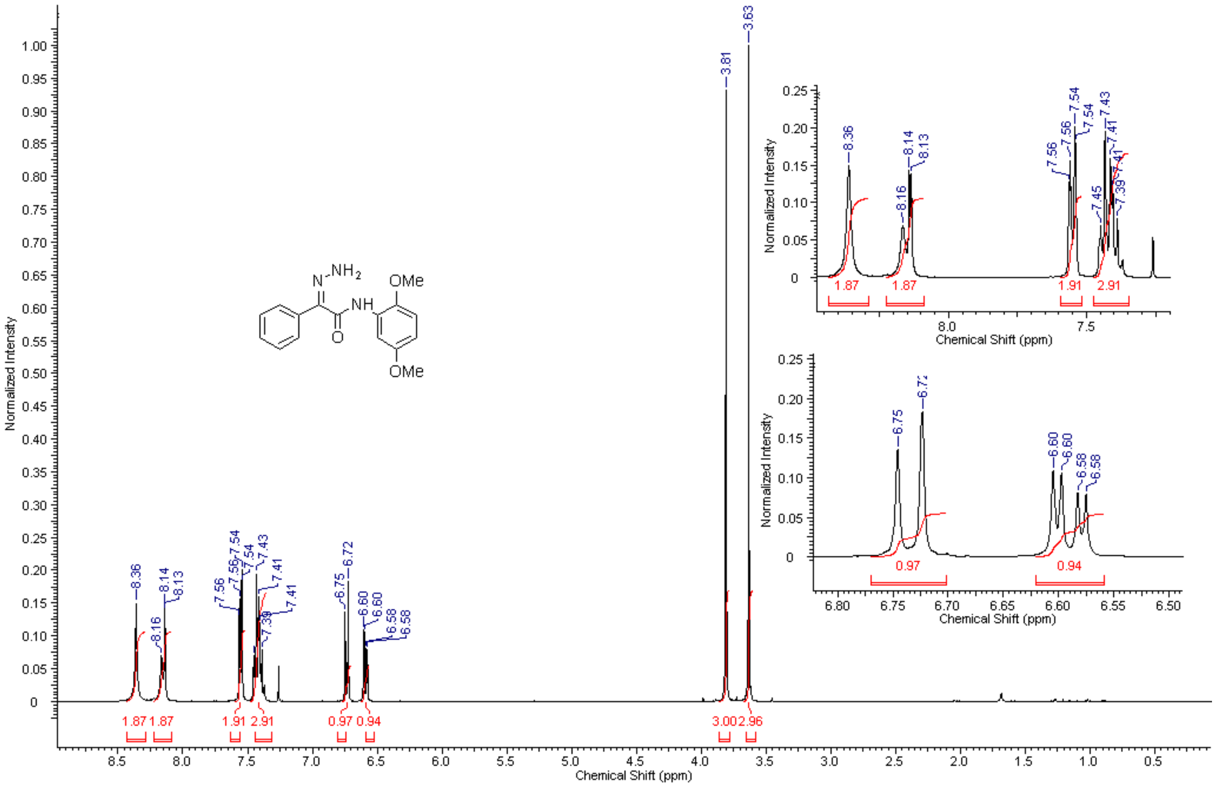

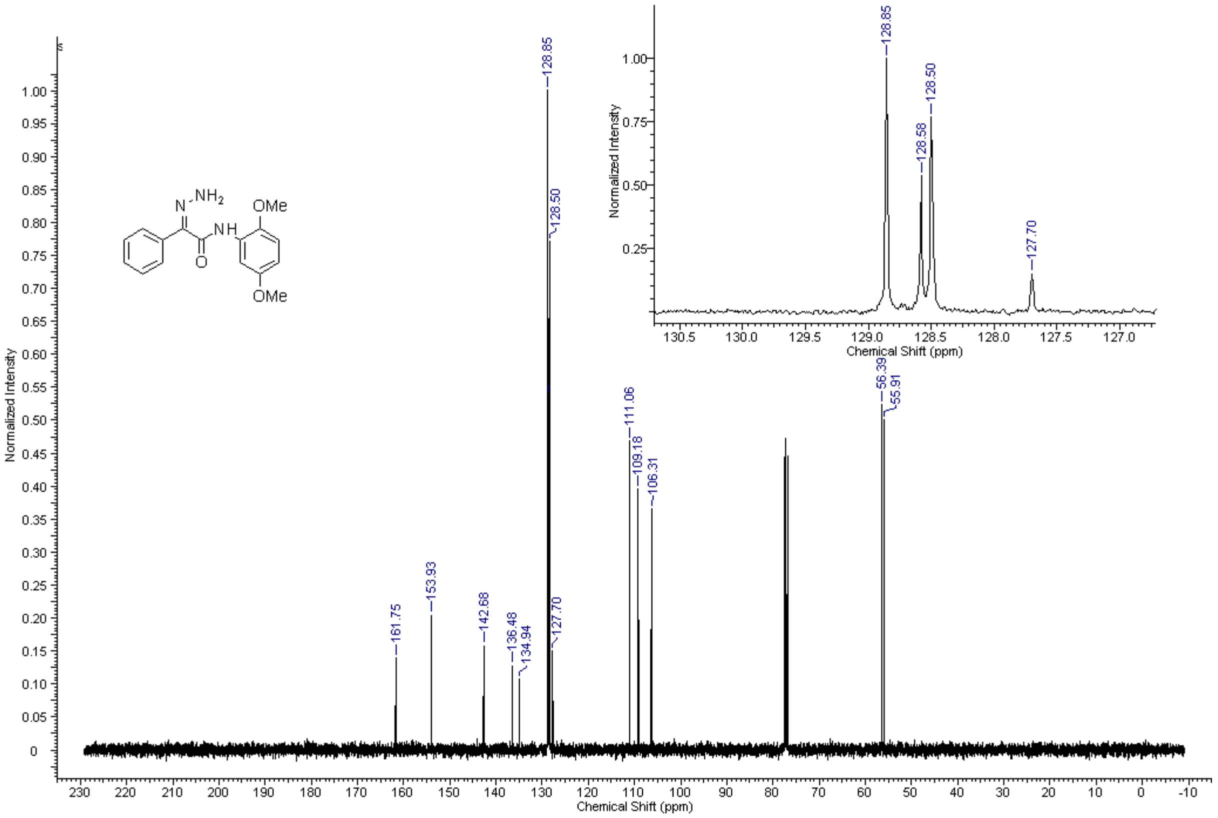

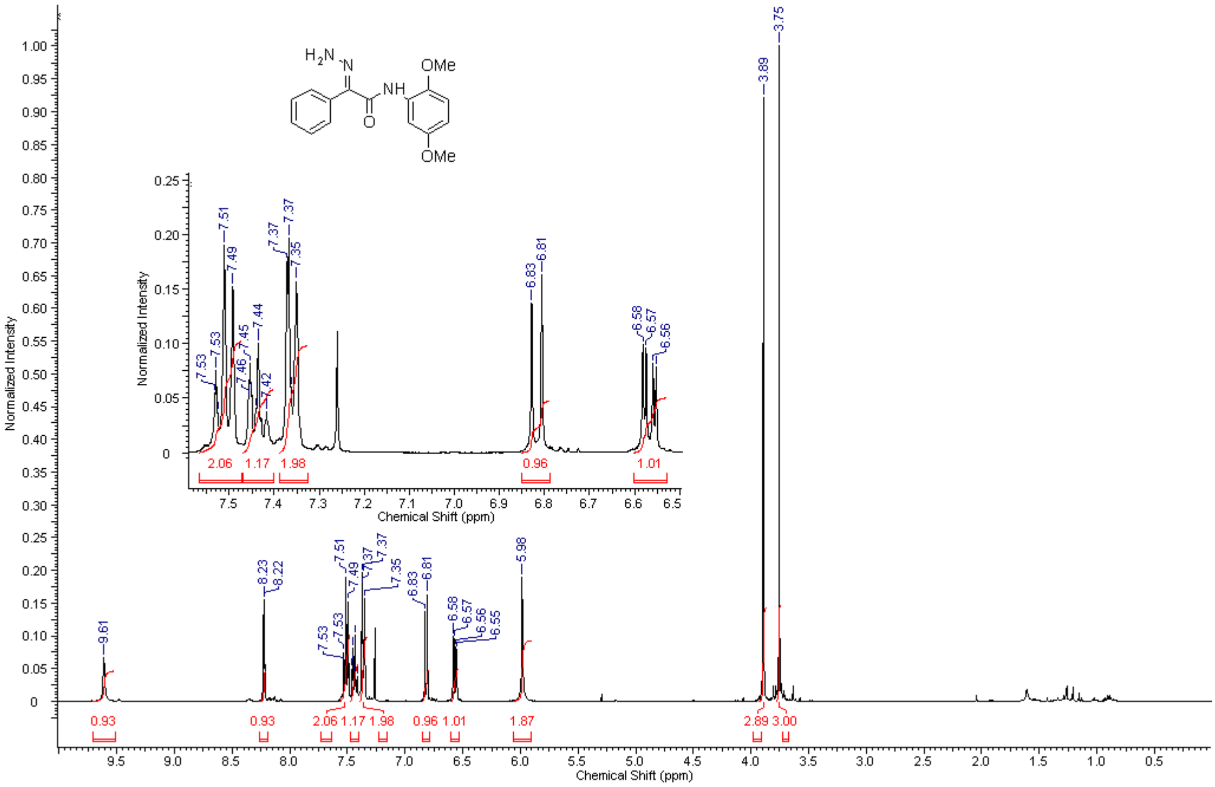
H2N N
NH
OMe
O
OMe
Normalized Intensity
Chemical Shift (ppm)
9.61
8.23
8.22
7.53
7.53
7.51
7.49
7.37
7.37
7.35
6.83
6.81
6.58
6.57
6.56
6.55
5.98
3.89
3.75
0.93
0.93
2.06
1.17
1.98
0.96
1.01
1.87
2.89
3.00

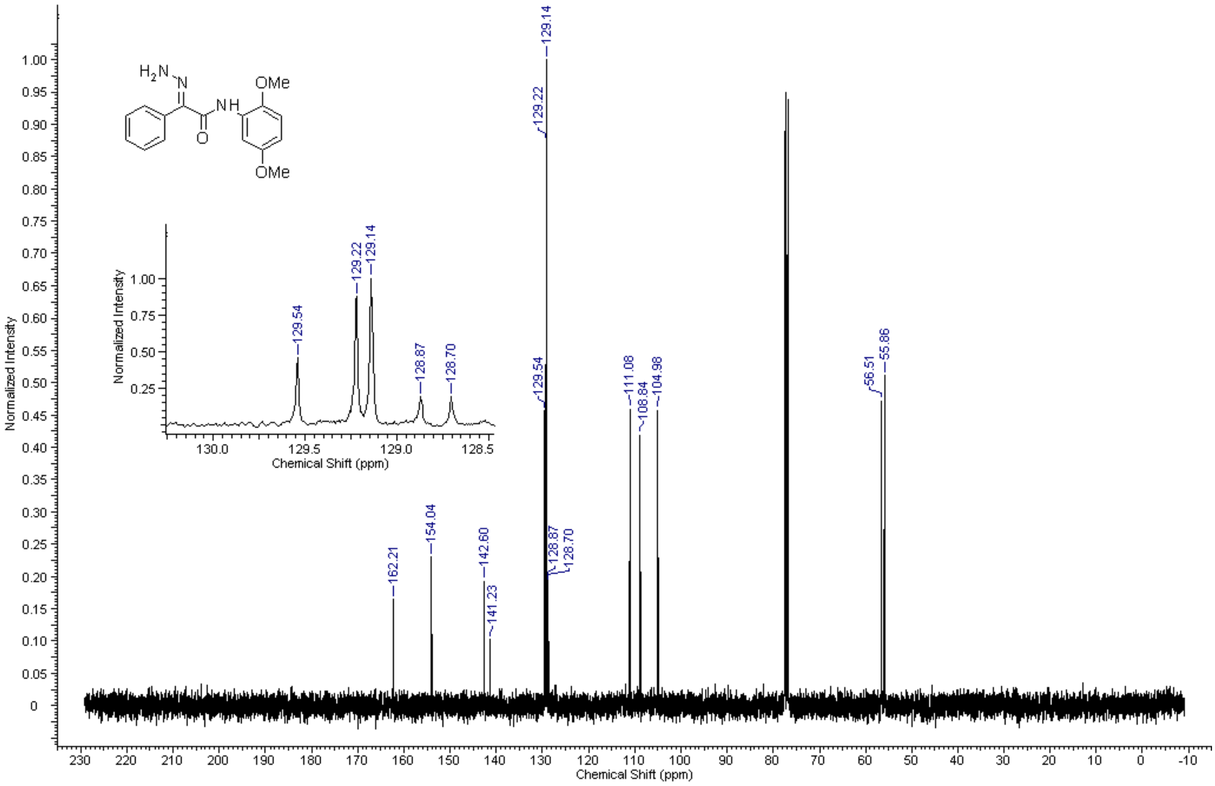
H2N N
NH
OMe
O
OMe
Normalized Intensity
Chemical Shift (ppm)
162.21
154.04
142.60
141.23
129.54
129.22
129.14
128.87
128.70
111.08
108.84
104.98
56.51
55.86

(*E*)- and (*Z*)-*N*-(2-Furylmethyl)-2-hydrazono-2-phenylacetamide 6r

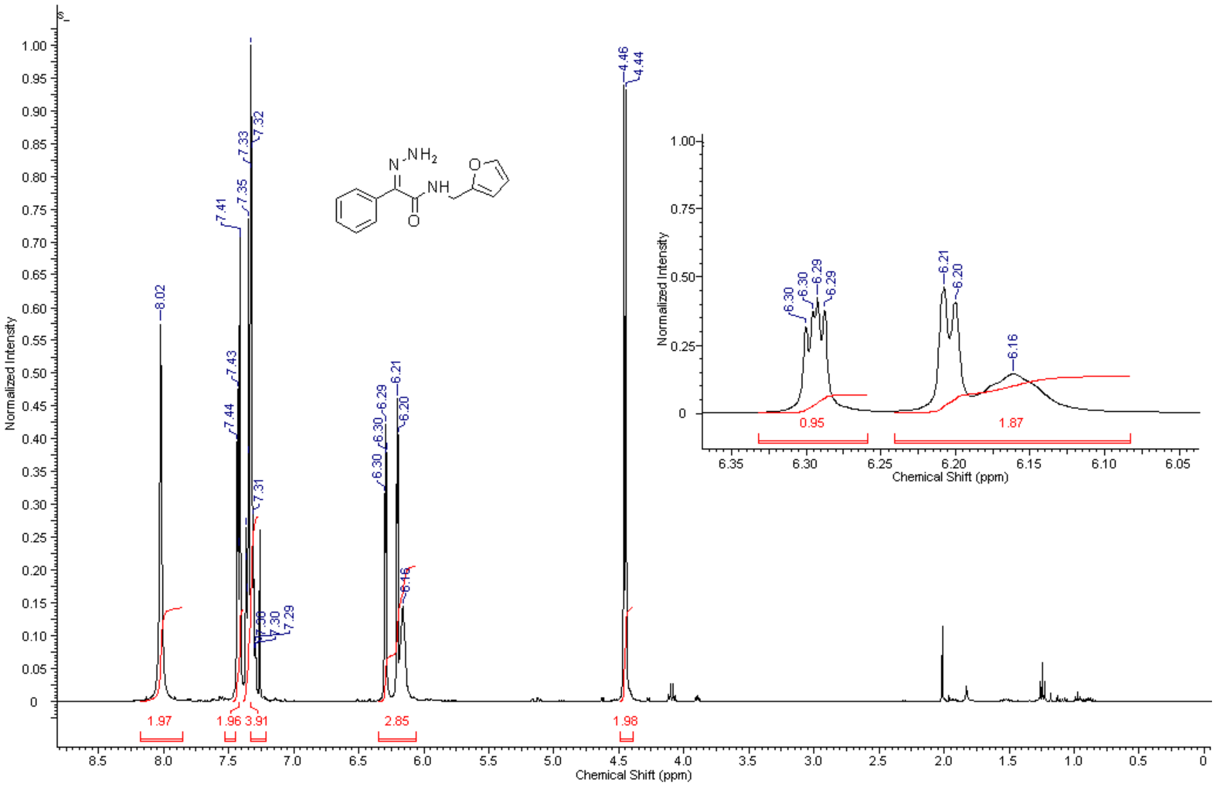

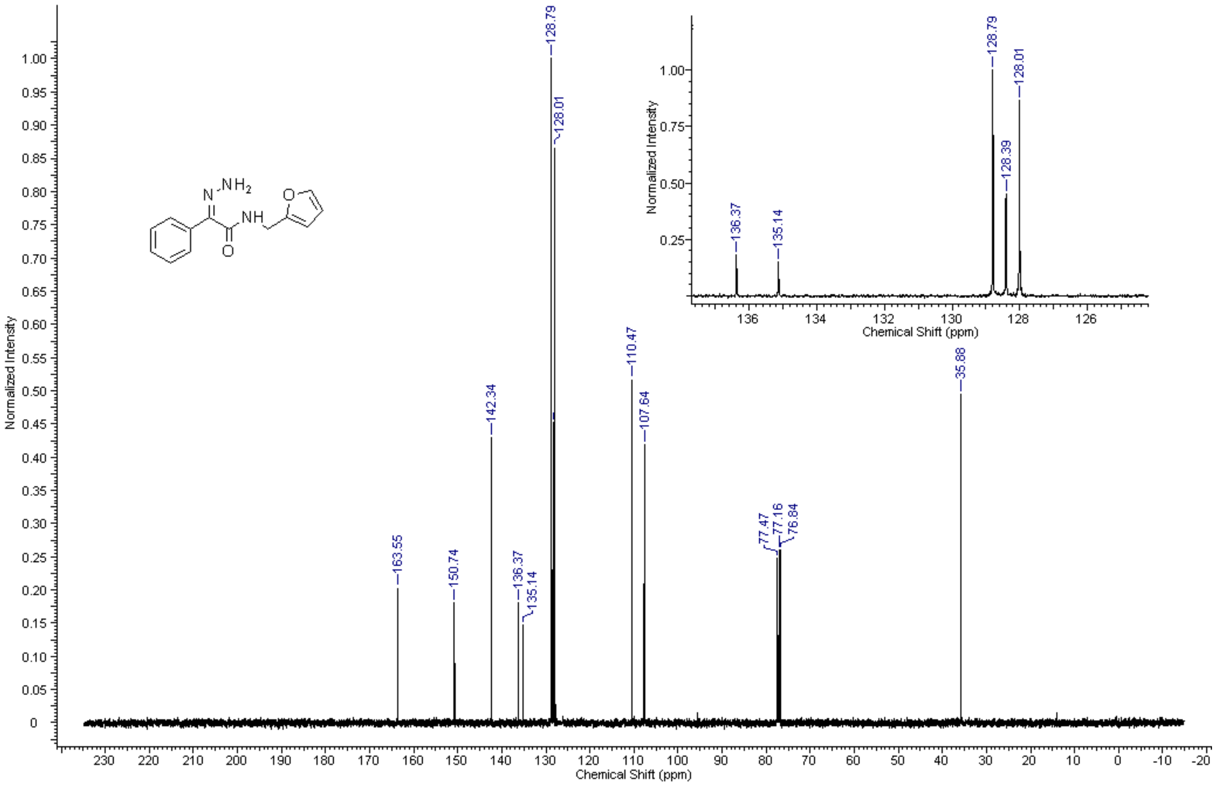

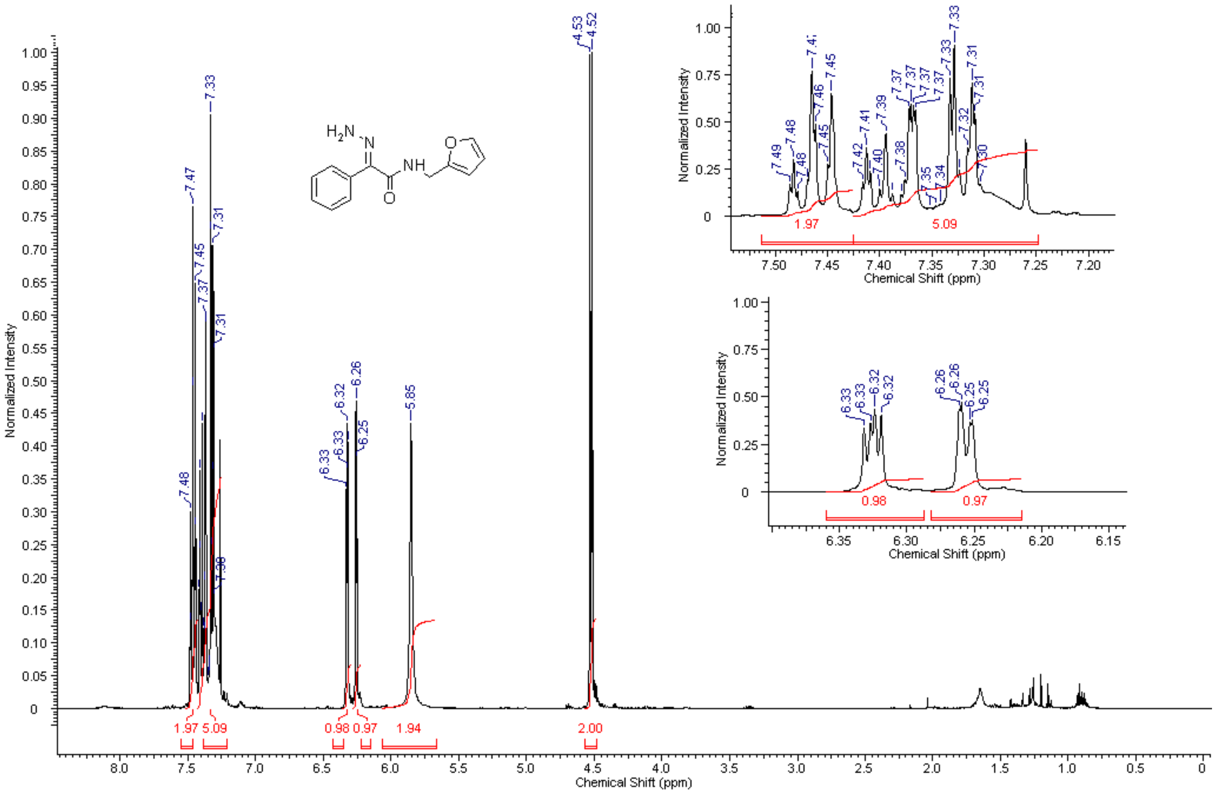

H2N
N
NH
O
O
Normalized Intensity
Chemical Shift (ppm)
7.47
7.45
7.37
7.33
7.31
7.48
6.33
6.32
6.26
6.25
5.85
4.53
4.52
1.97
5.09
0.98
0.97
1.94
2.00

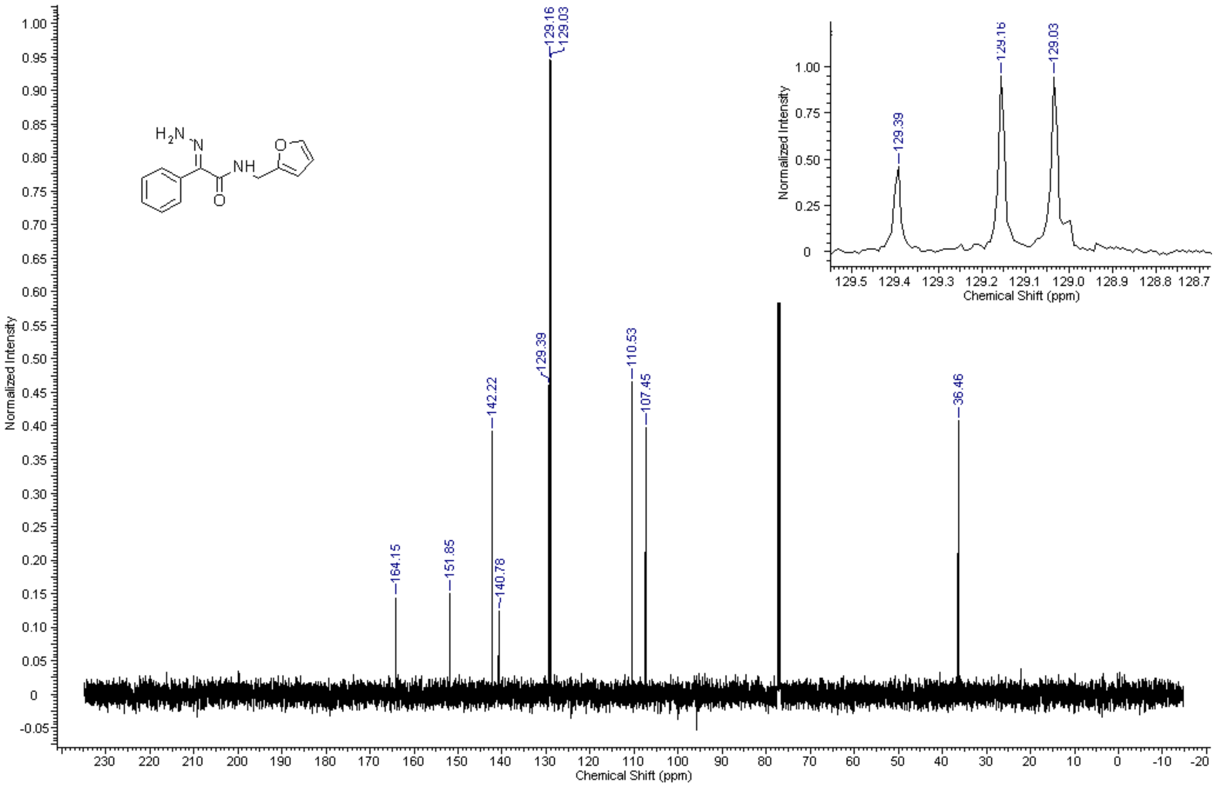

H2N
N
NH
O
O
Normalized Intensity
Chemical Shift (ppm)
164.15
151.85
142.22
140.78
129.39
129.16
129.03
110.53
107.45
36.46

2-Hydrazono-1-phenylethanone 8

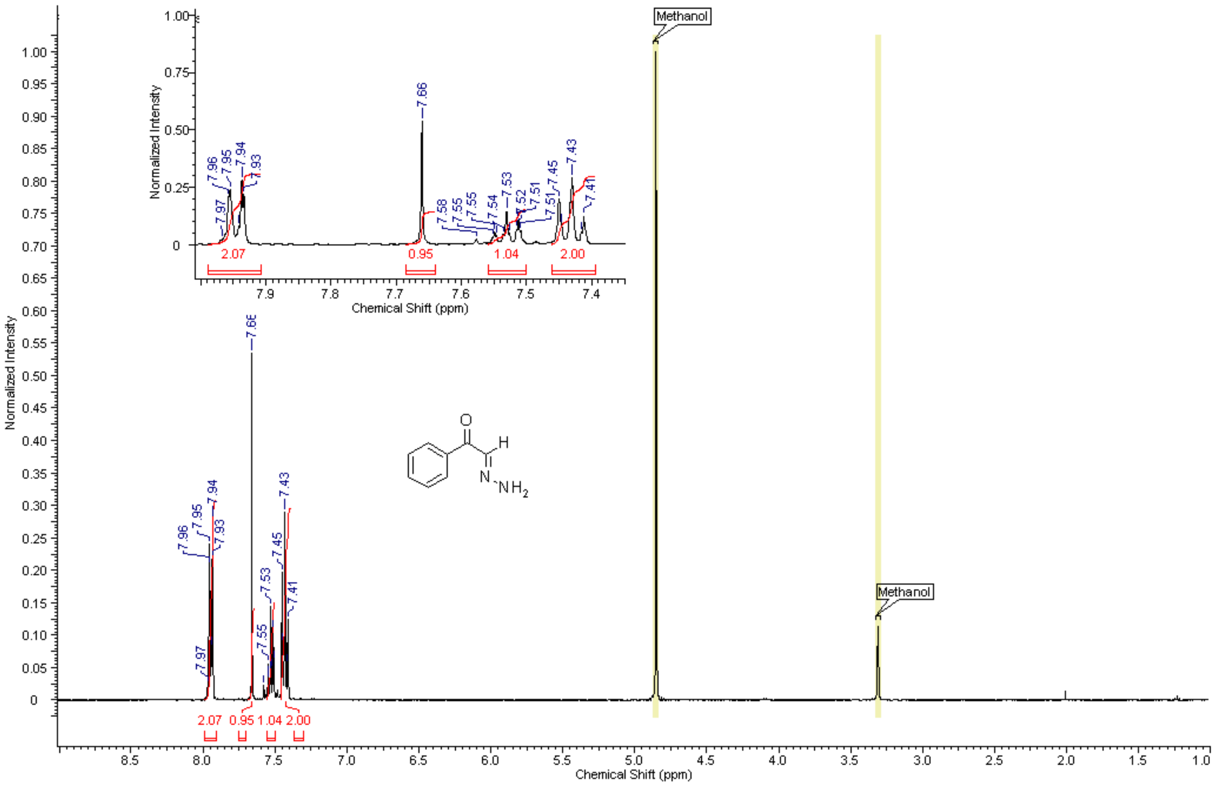

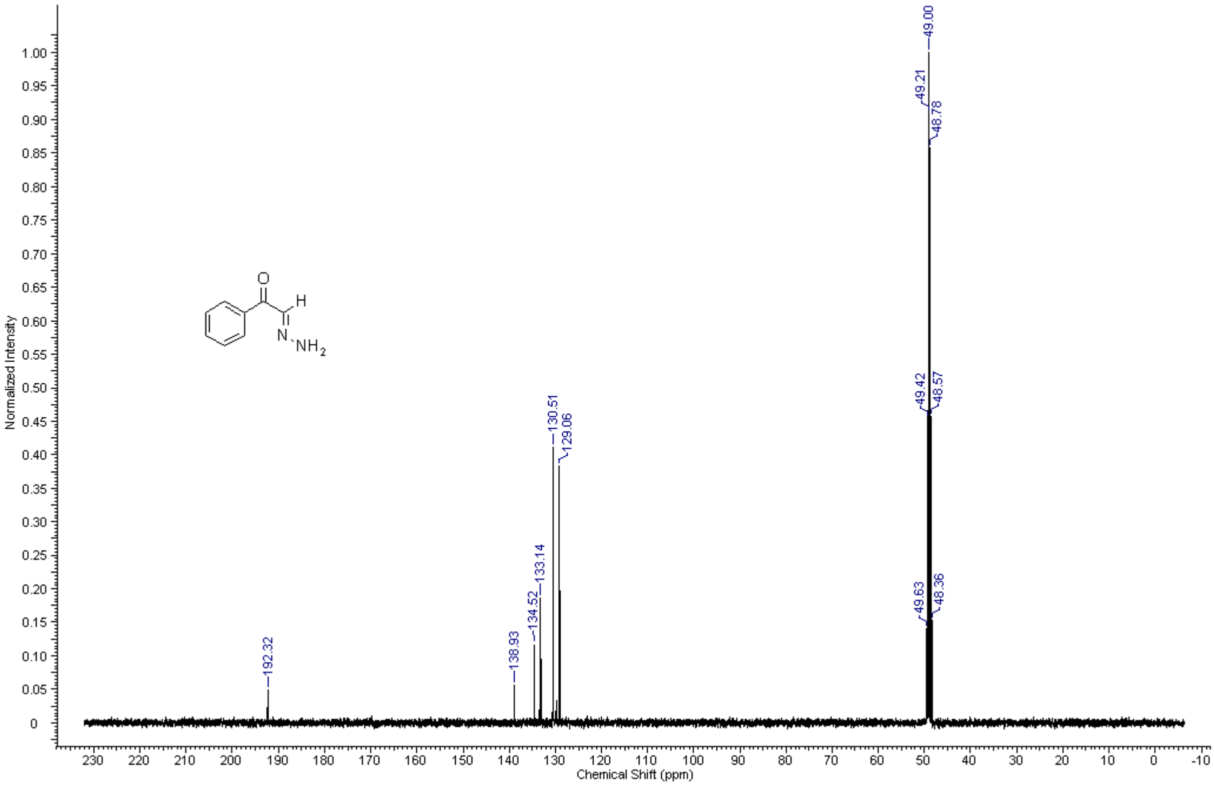

Dimethyl 1-hydrazonoethylphosphonate 7a

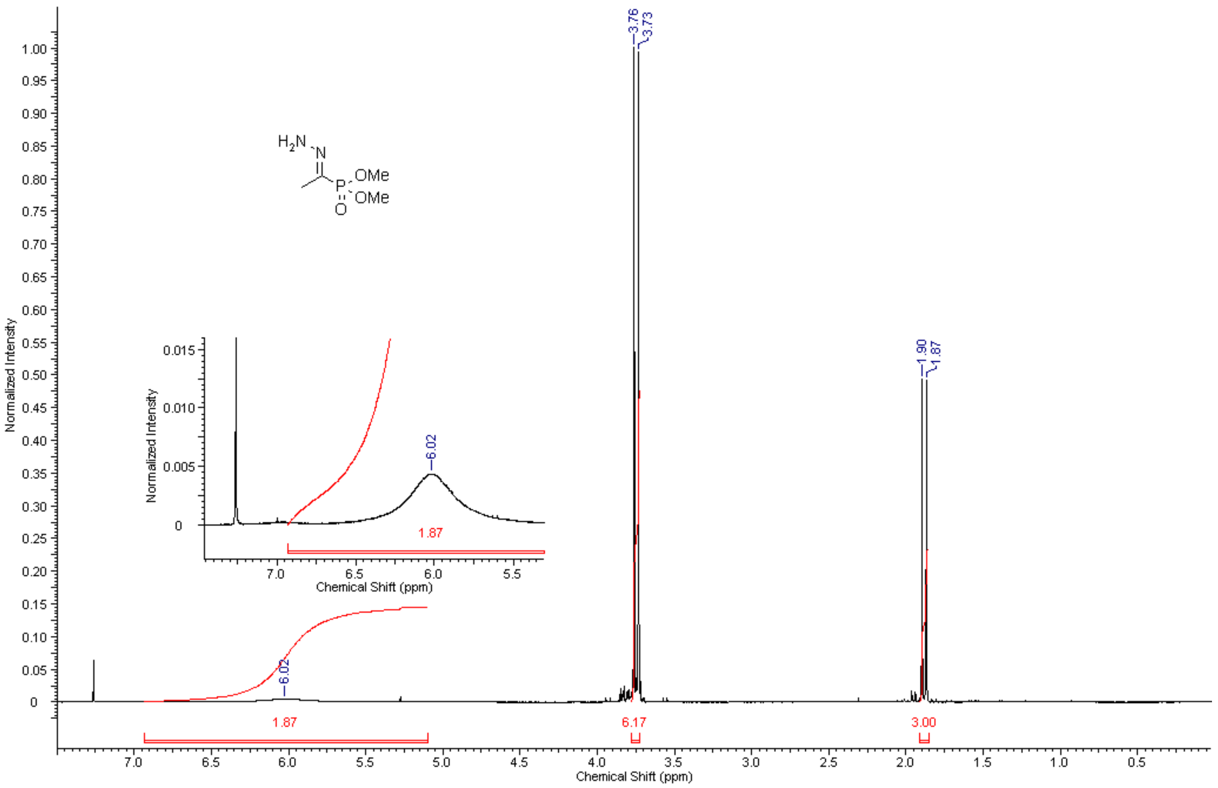

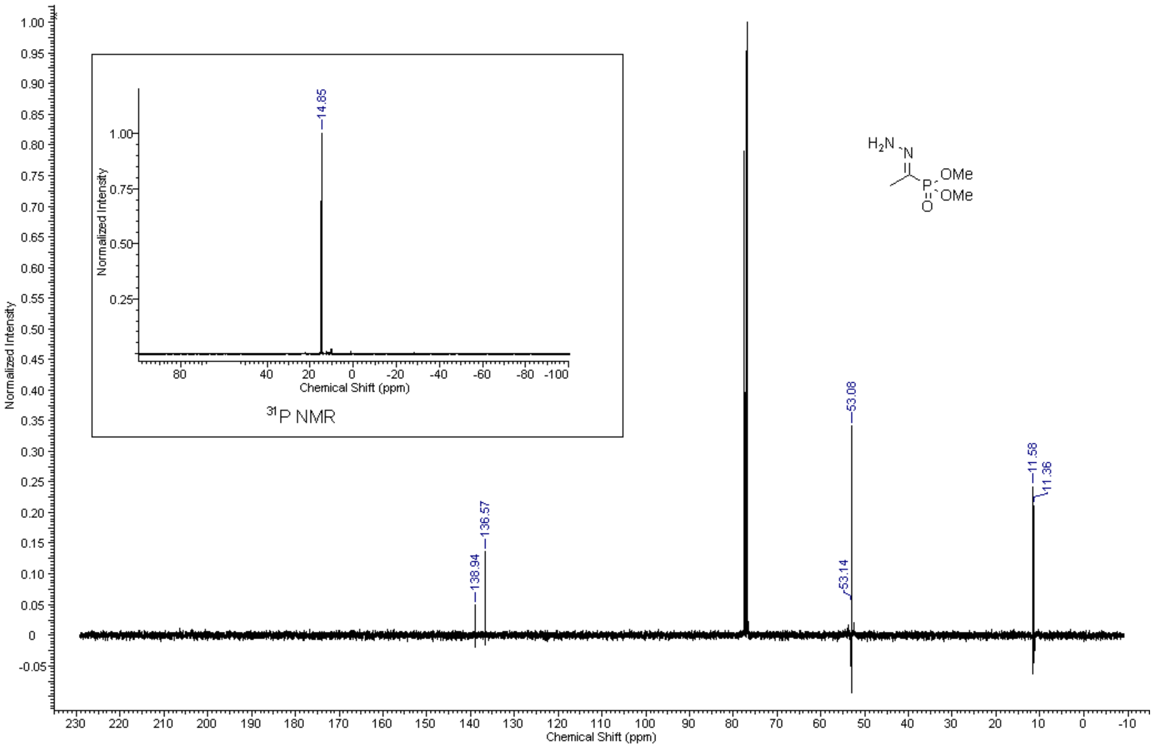

(*E*)- and (*Z*)-Diethyl 1-hydrazono-2-phenylethylphosphonate 7b

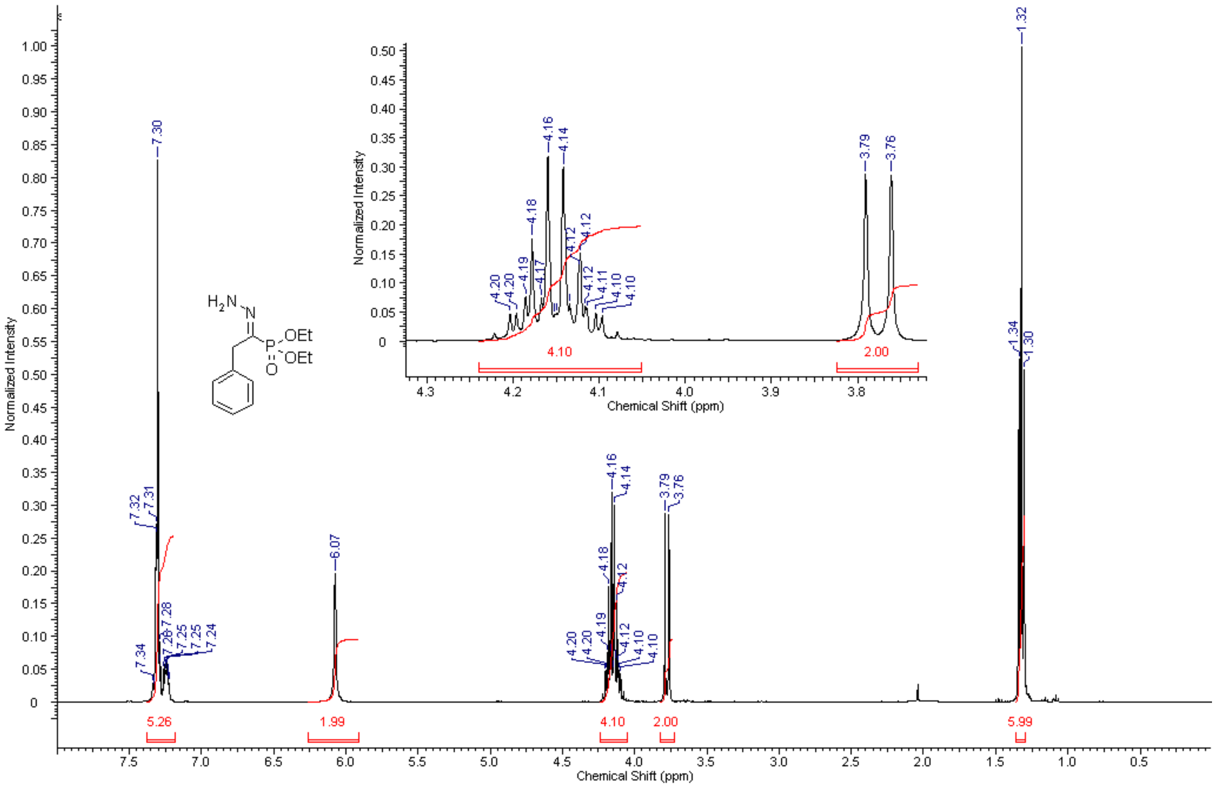

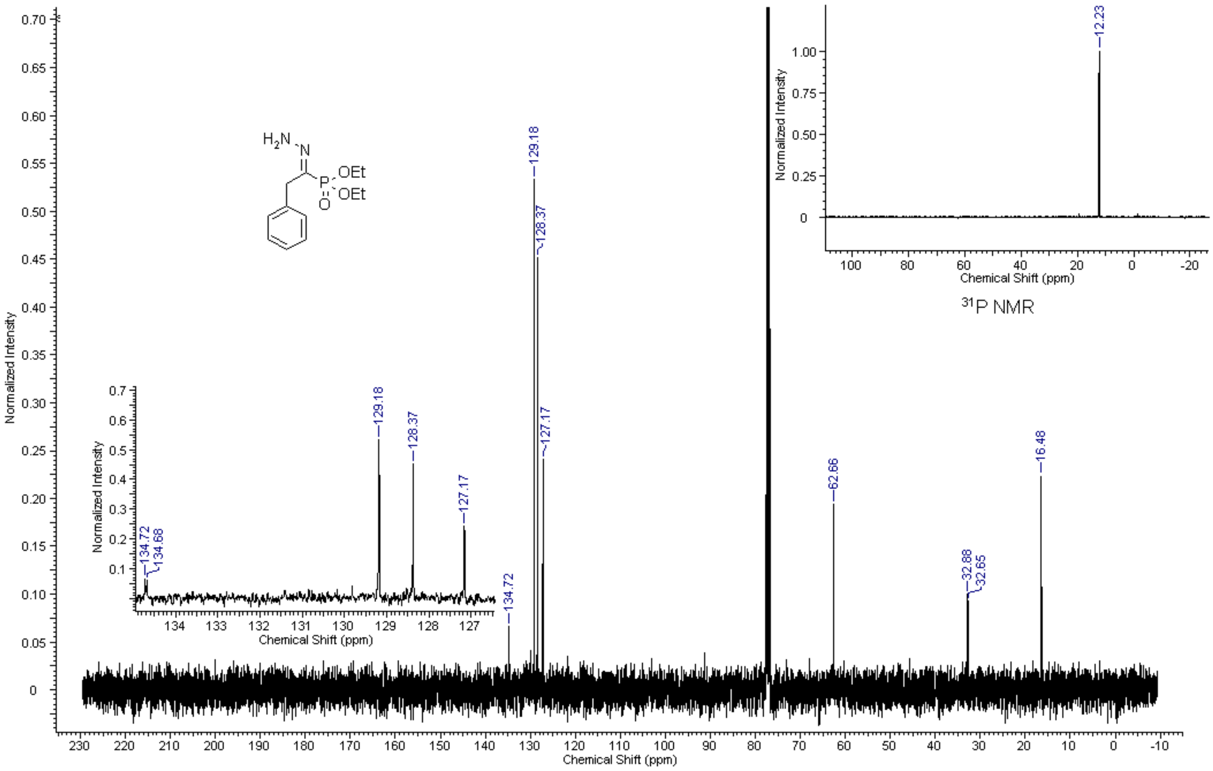

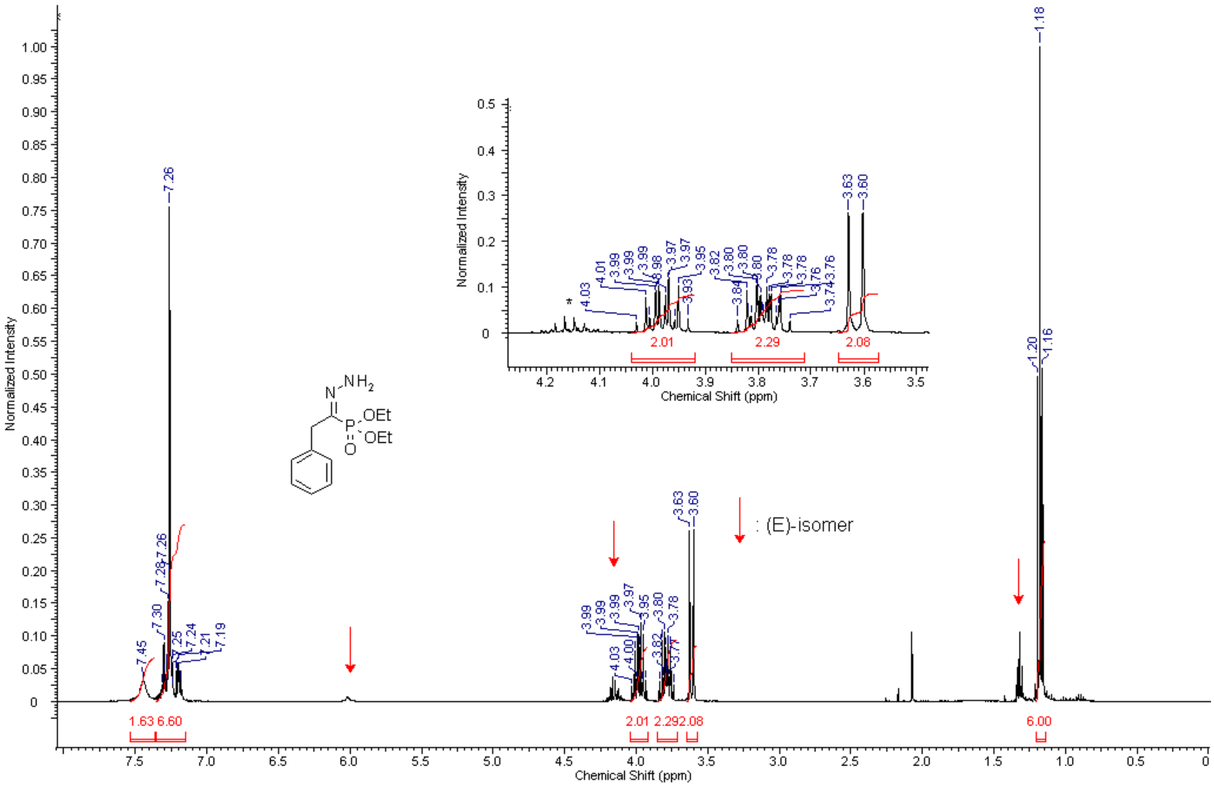
Normalized Intensity
Chemical Shift (ppm)
: (E)-isomer
OEt
OEt
NH2
1.63 6.60
2.01 2.29 2.08
6.00
2.01
2.29
2.08

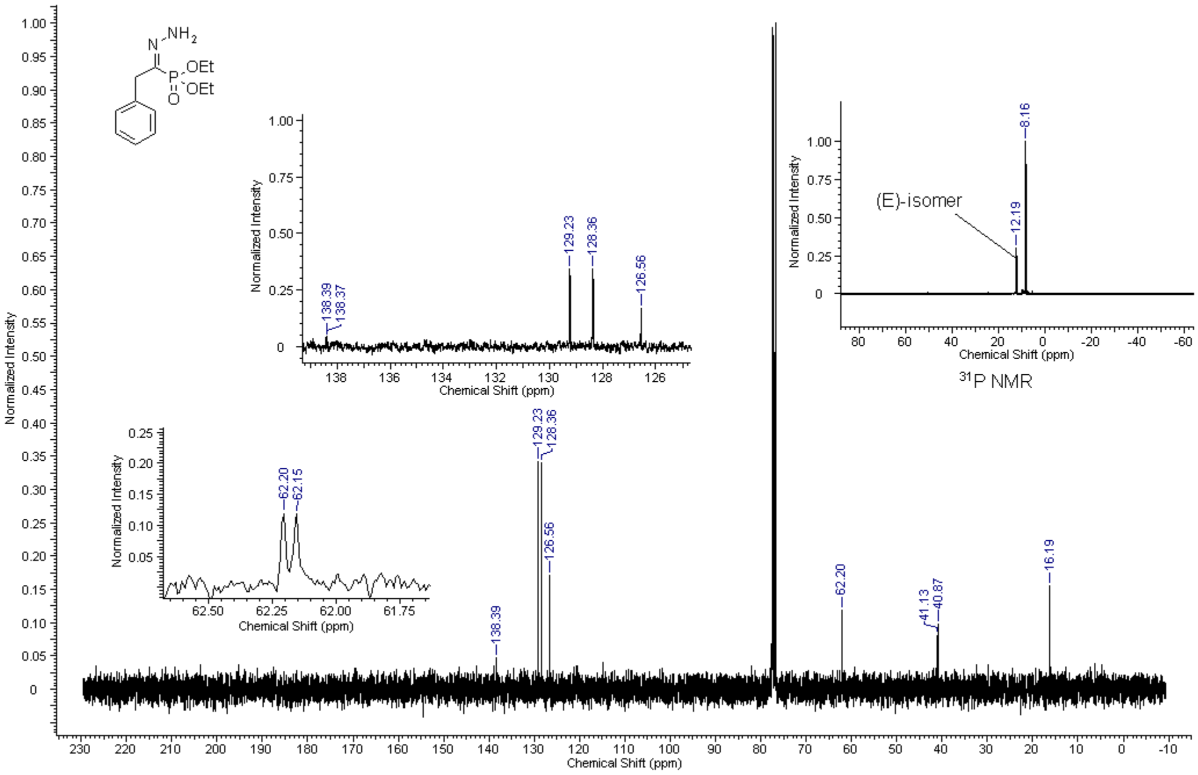
Normalized Intensity
Chemical Shift (ppm)
(E)-isomer
31P NMR
OEt
OEt
NH2

Diphenyldiazomethane 2

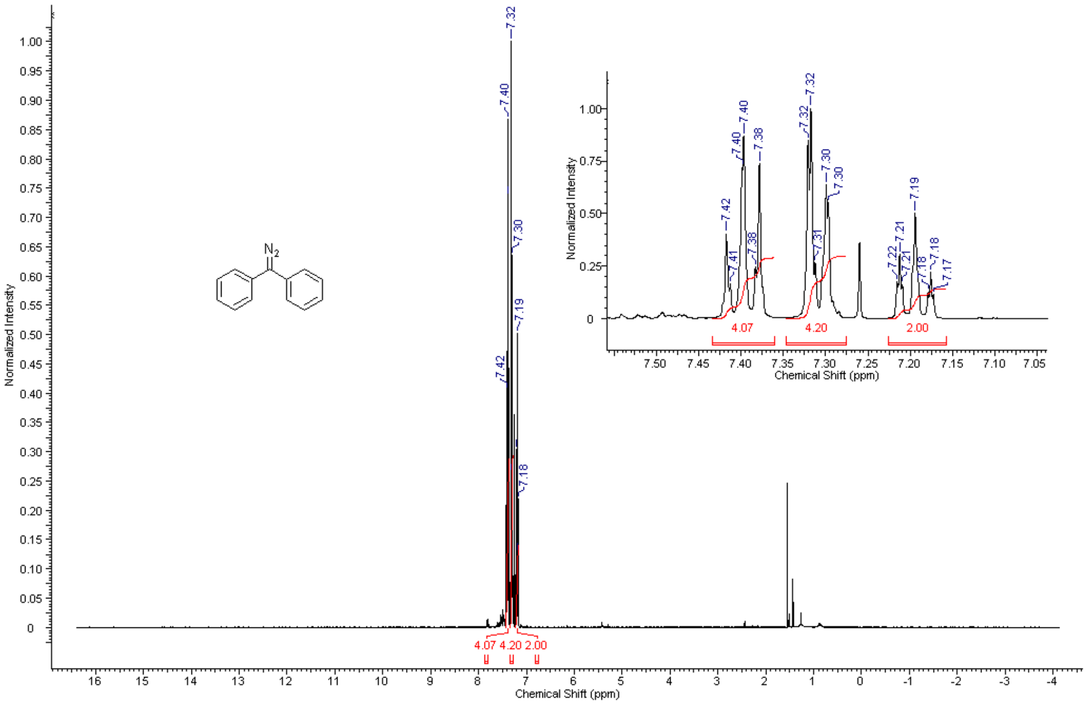

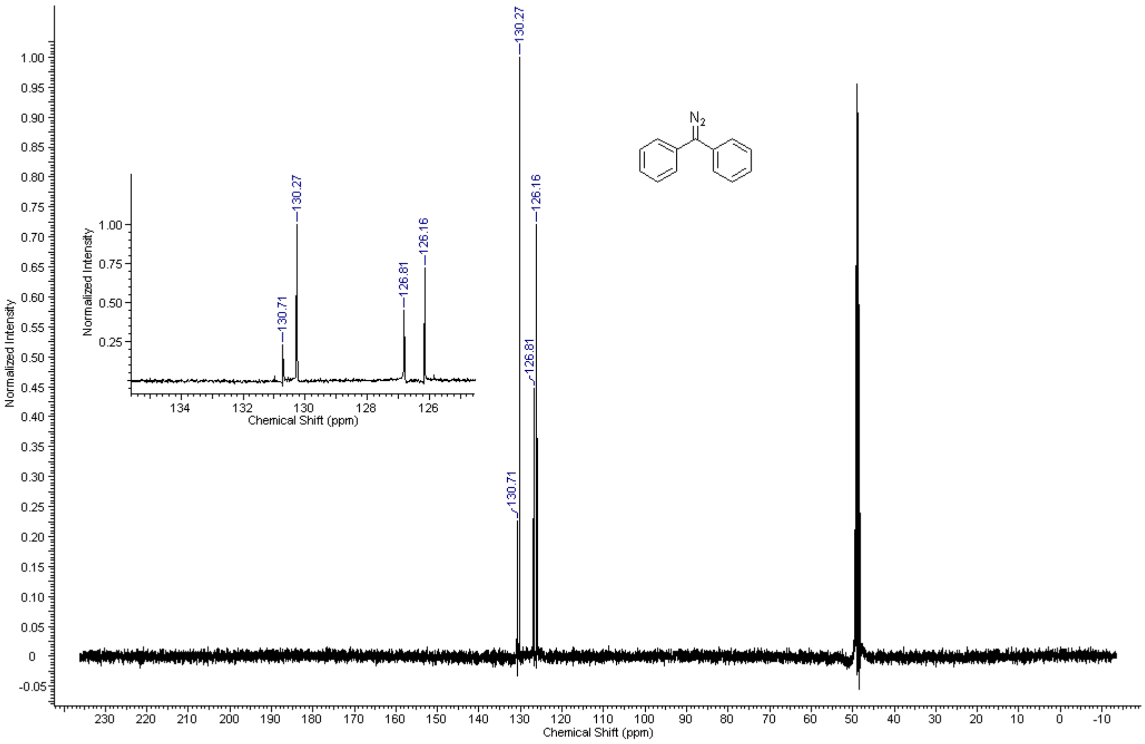

2-Diazo-1,2-diphenylethanone 10

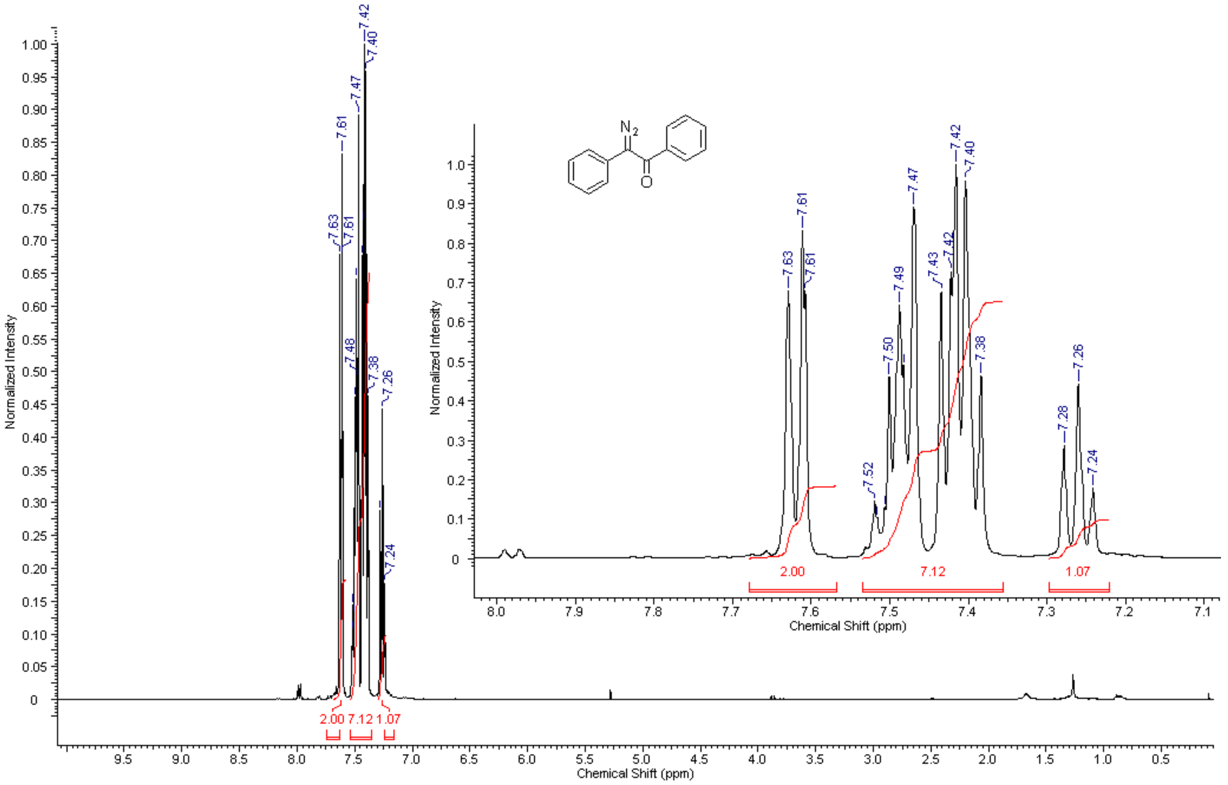

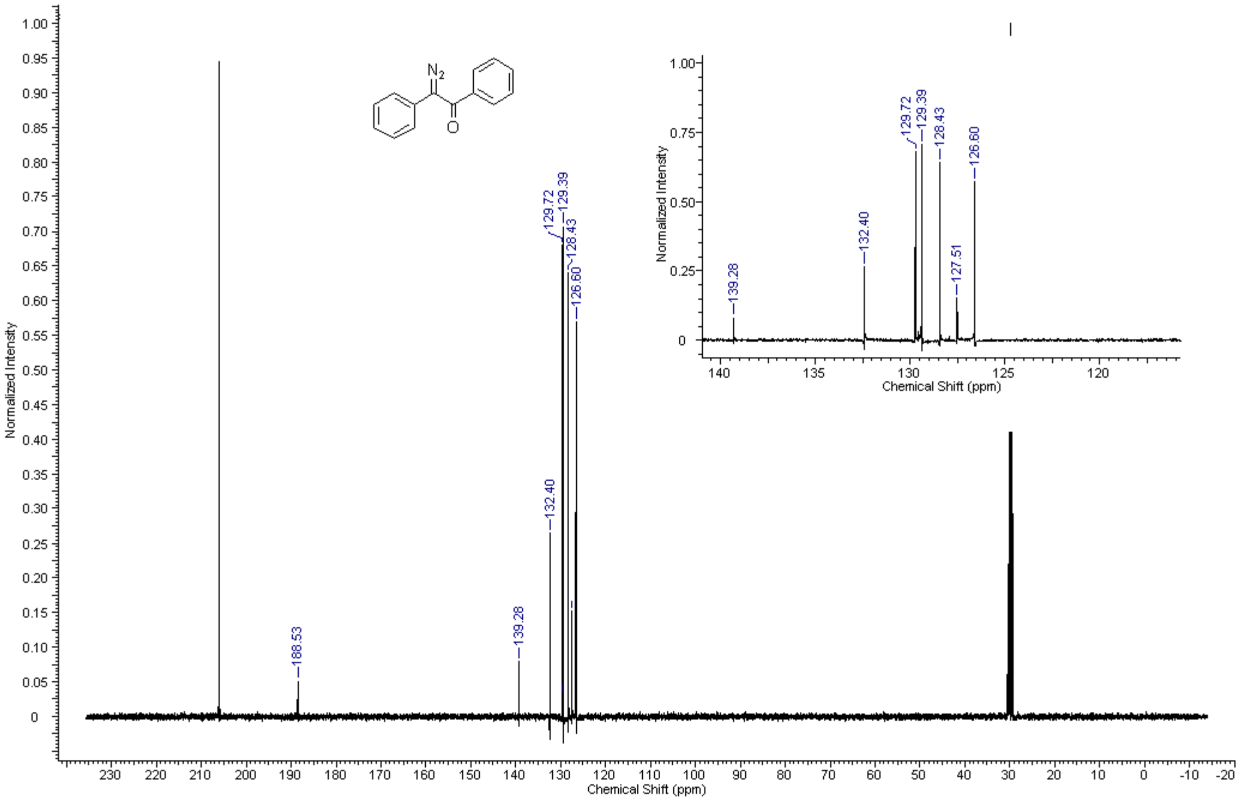

Ethyl 2-diazo-2-phenylacetate 11a

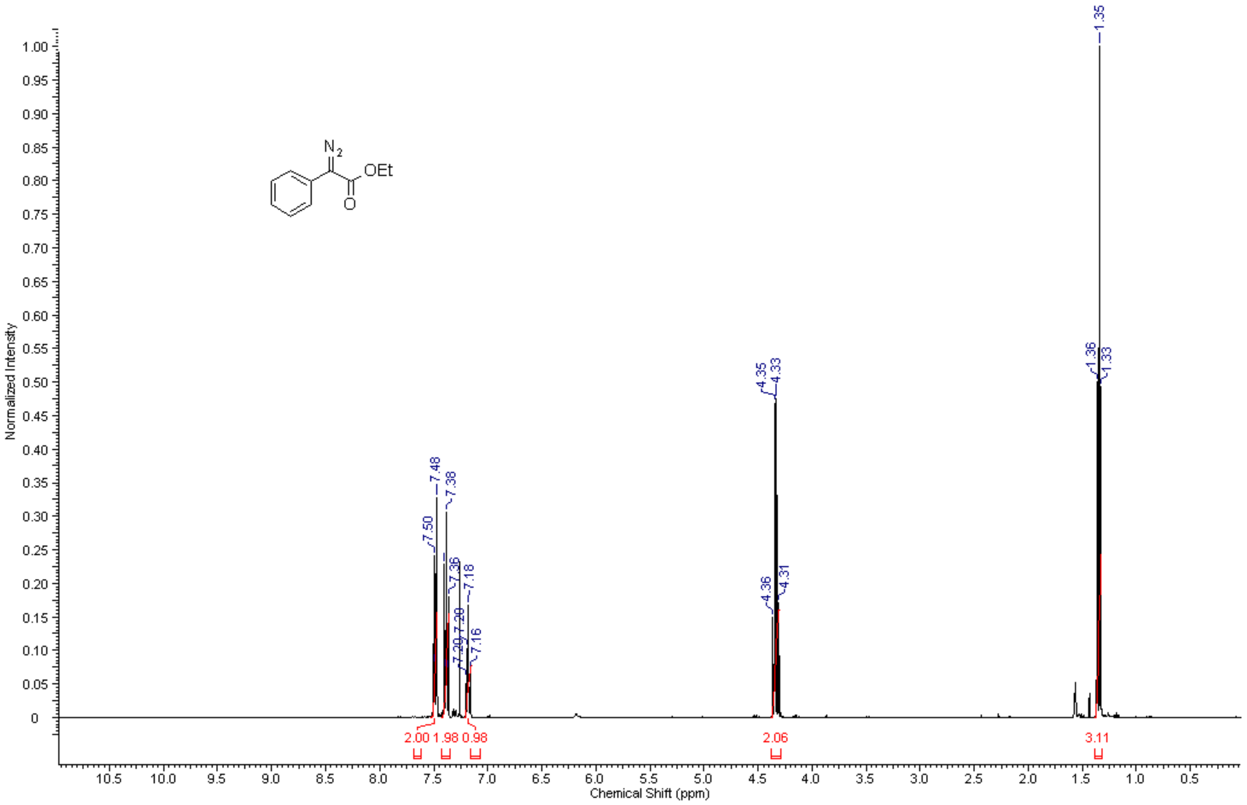

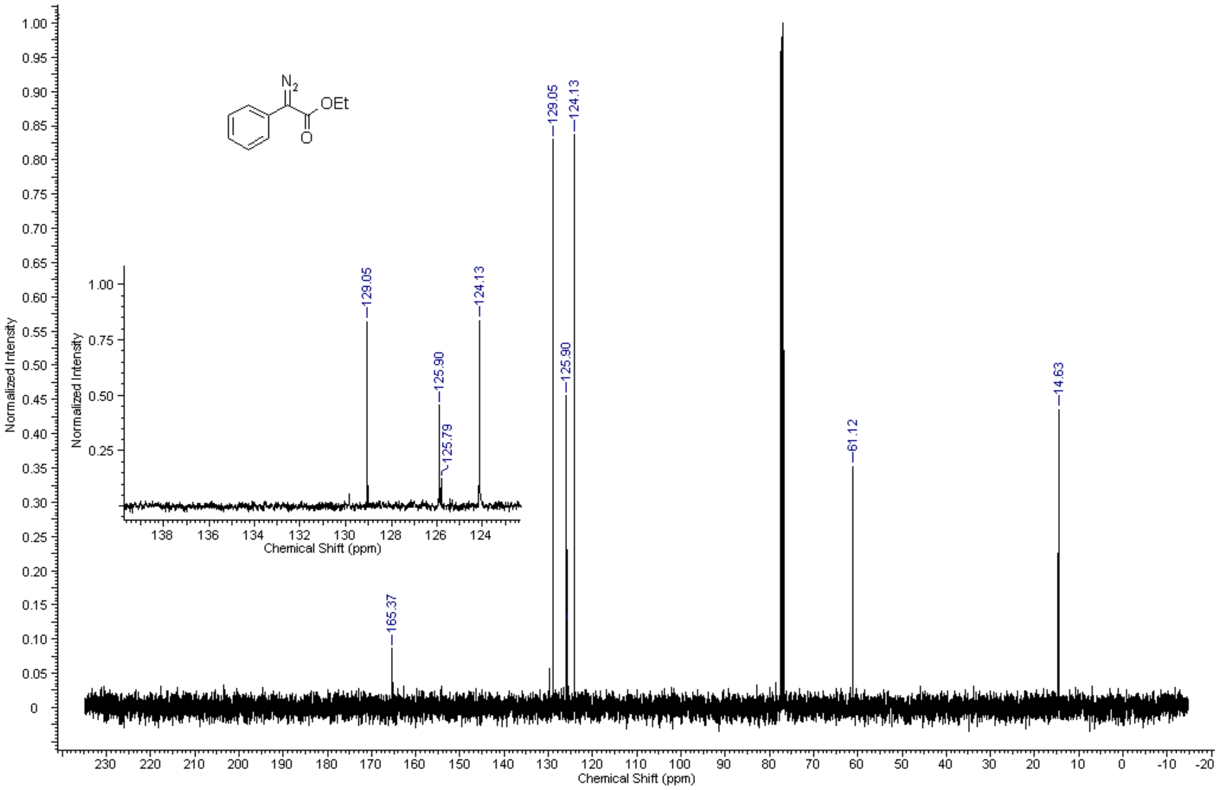

Ethyl 2-diazo-2-(4-methoxyphenyl)acetate 11b

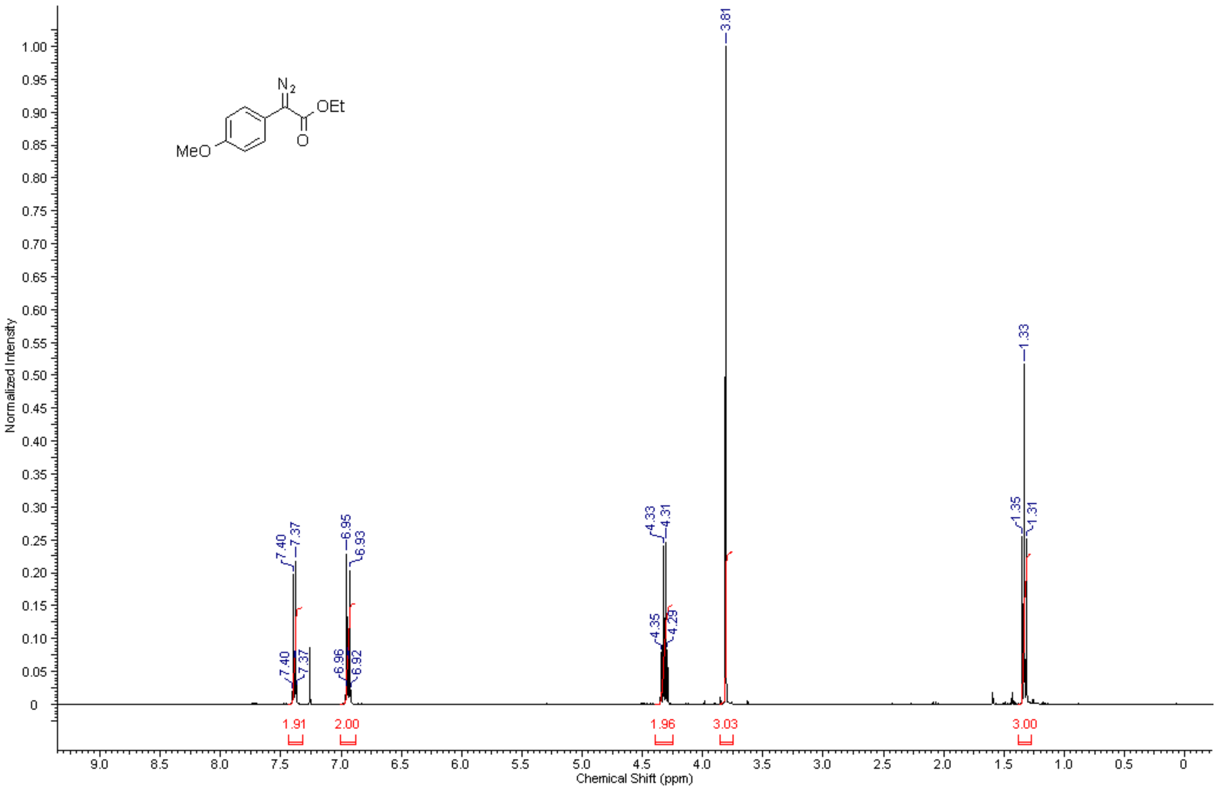

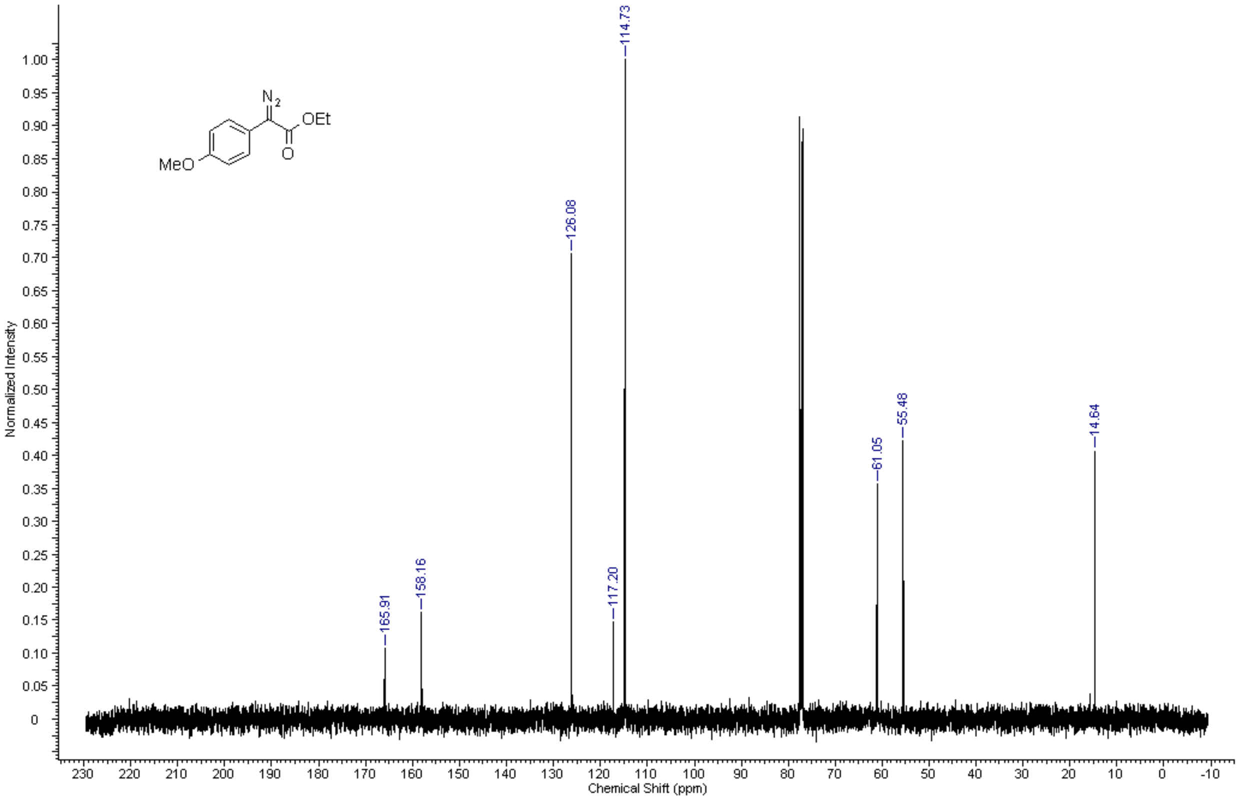

Ethyl 2-diazo-2-(2-methoxyphenyl)acetate 11c

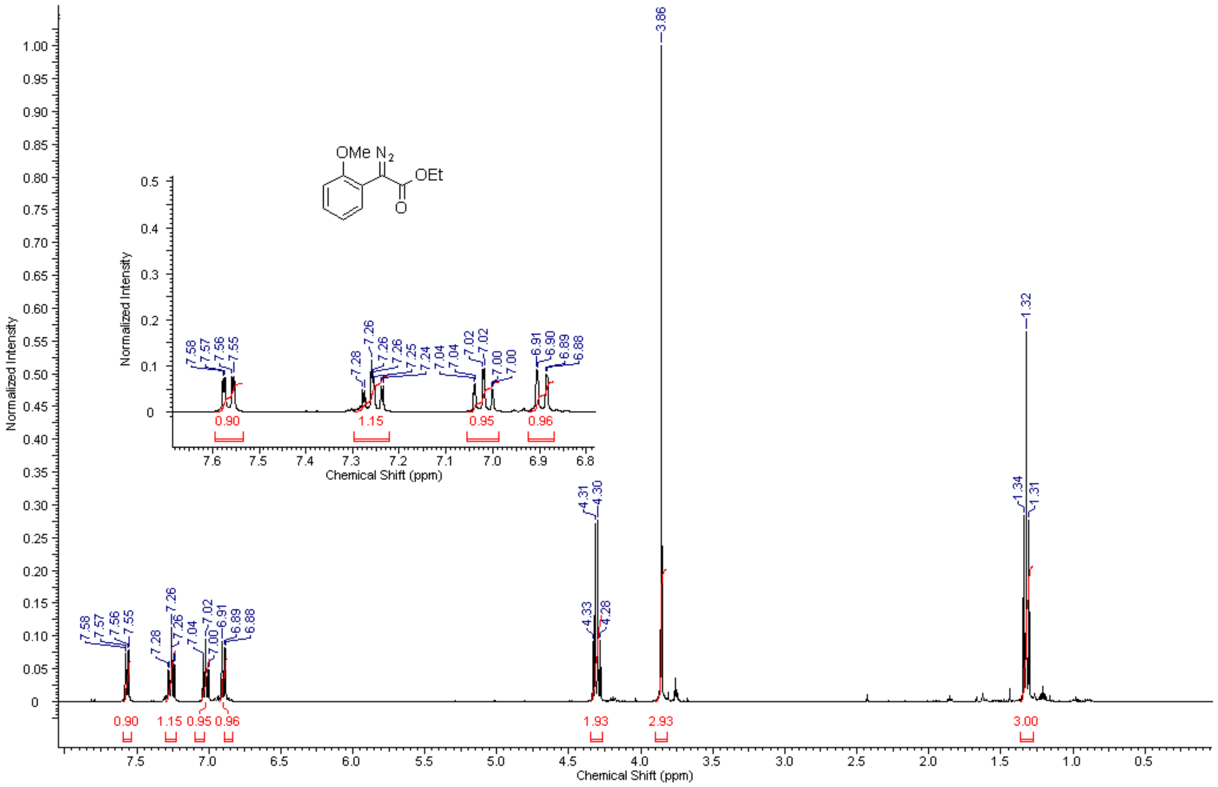

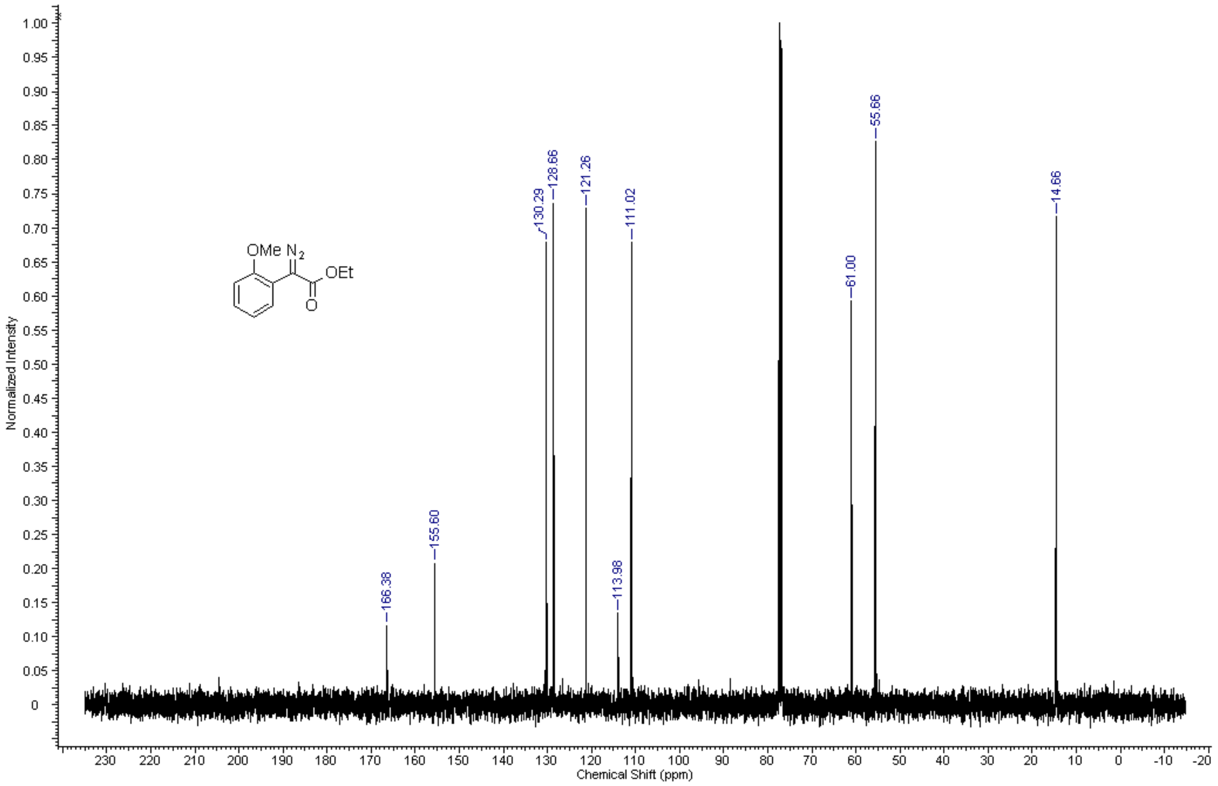

Ethyl 2-(4-bromophenyl)-2-diazoacetate 11d

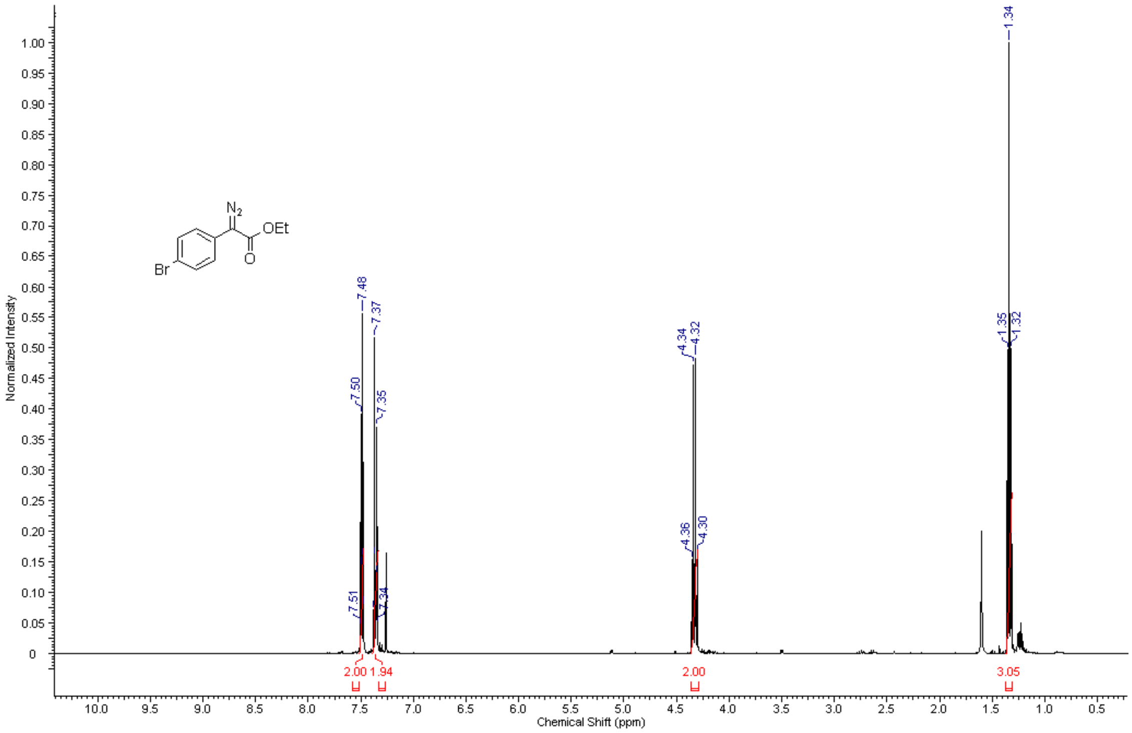

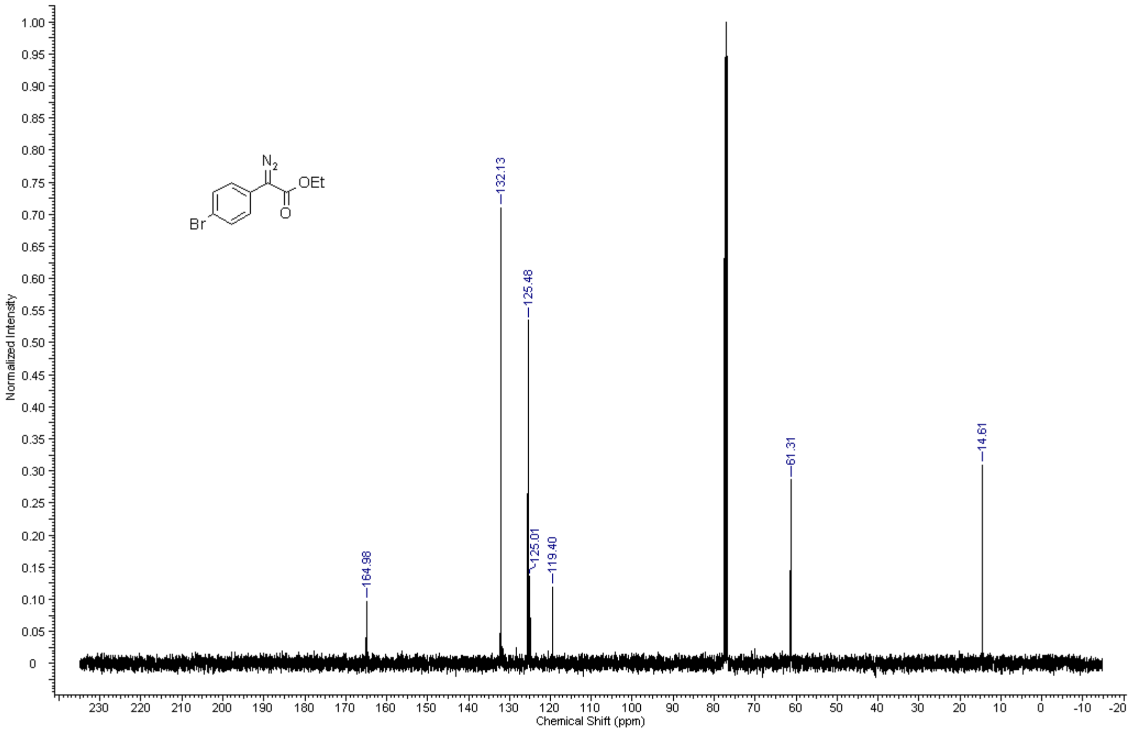

Ethyl 2-diazo-3-methylbutanoate 11e

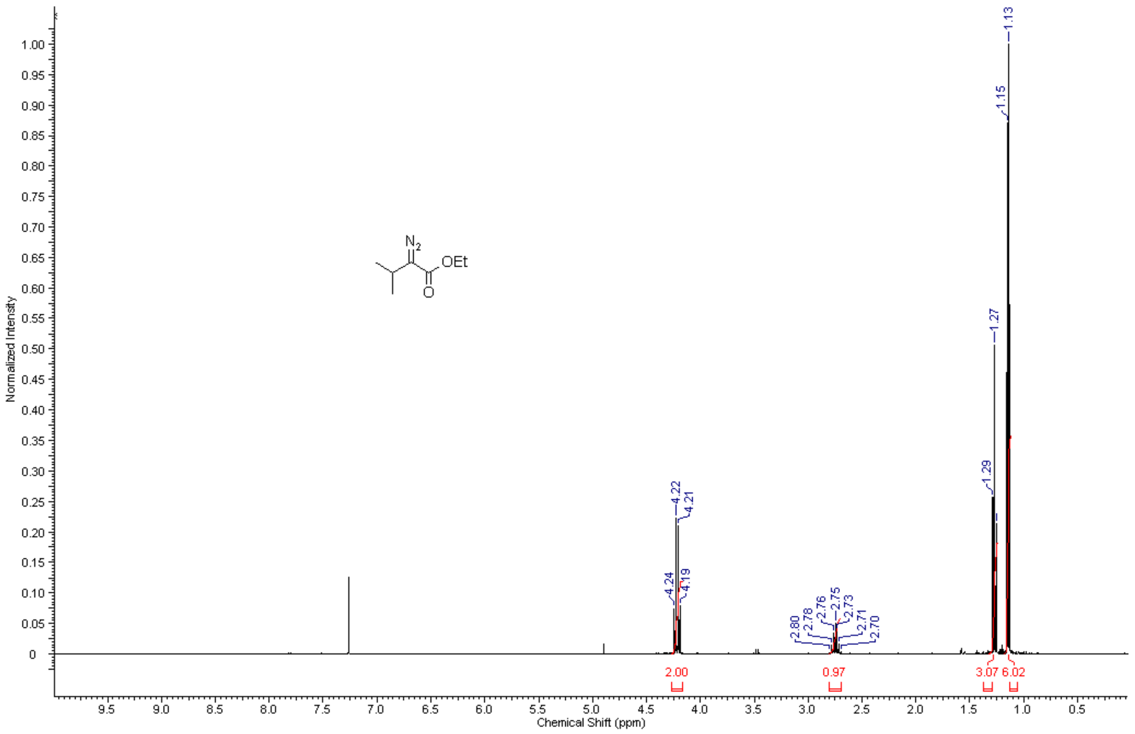

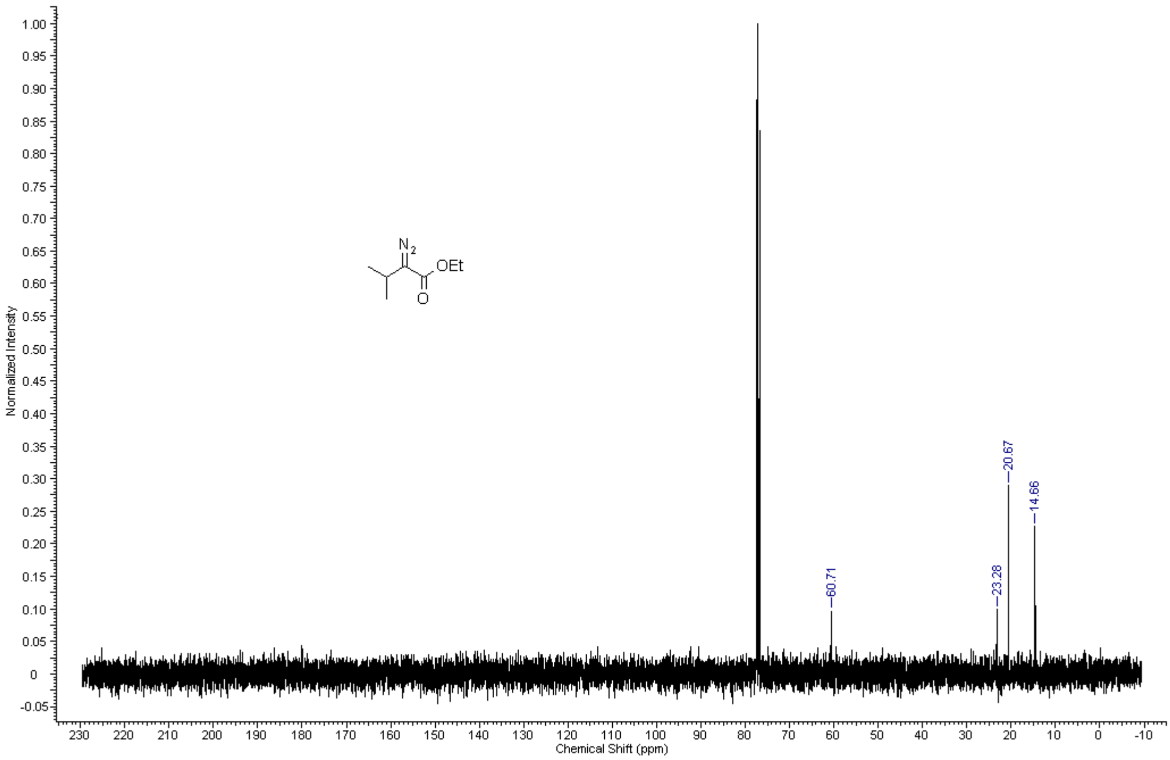

Ethyl 2-diazopropanoate 11f

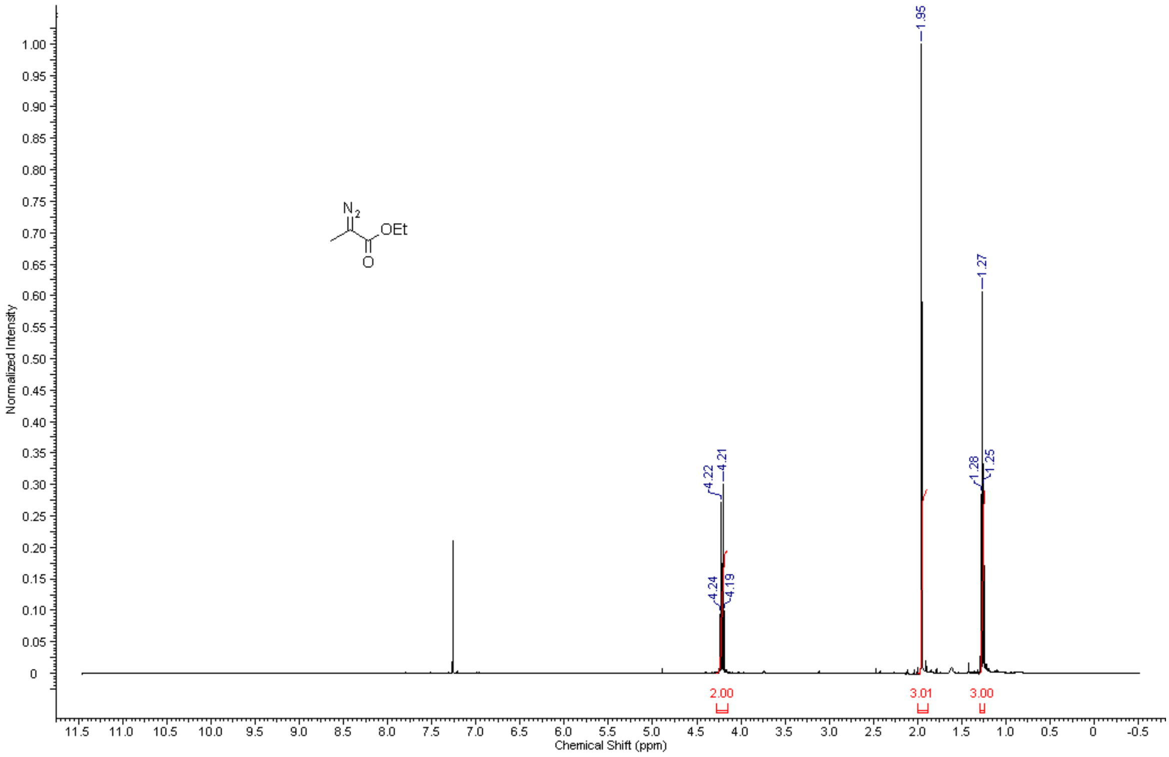

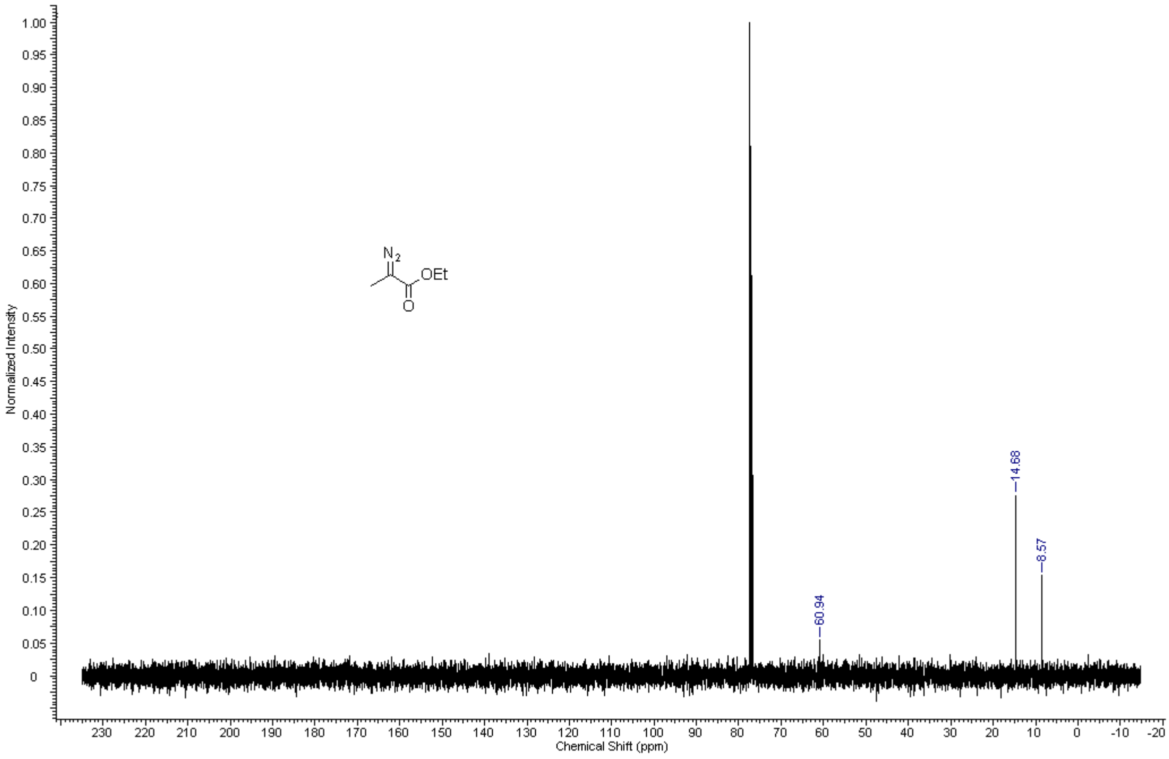

Ethyl 2-diazo-4-phenylbutanoate 11g

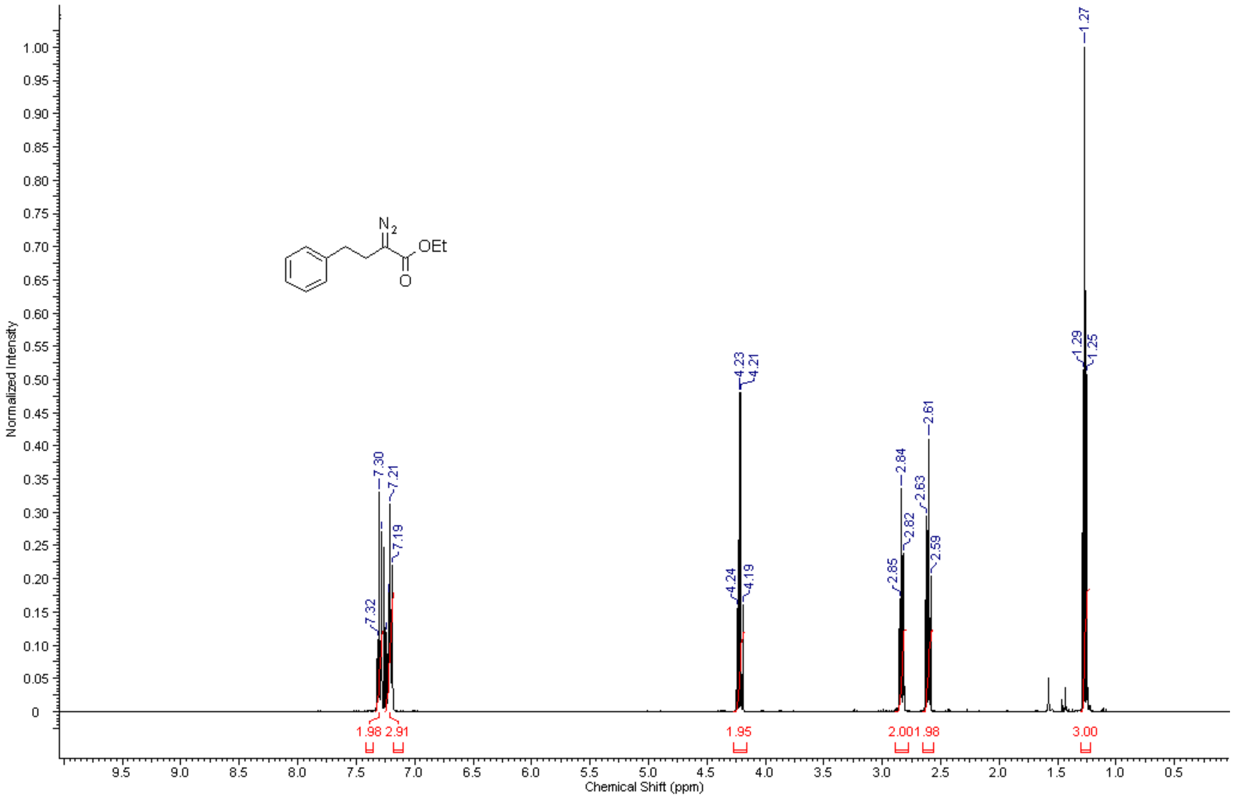

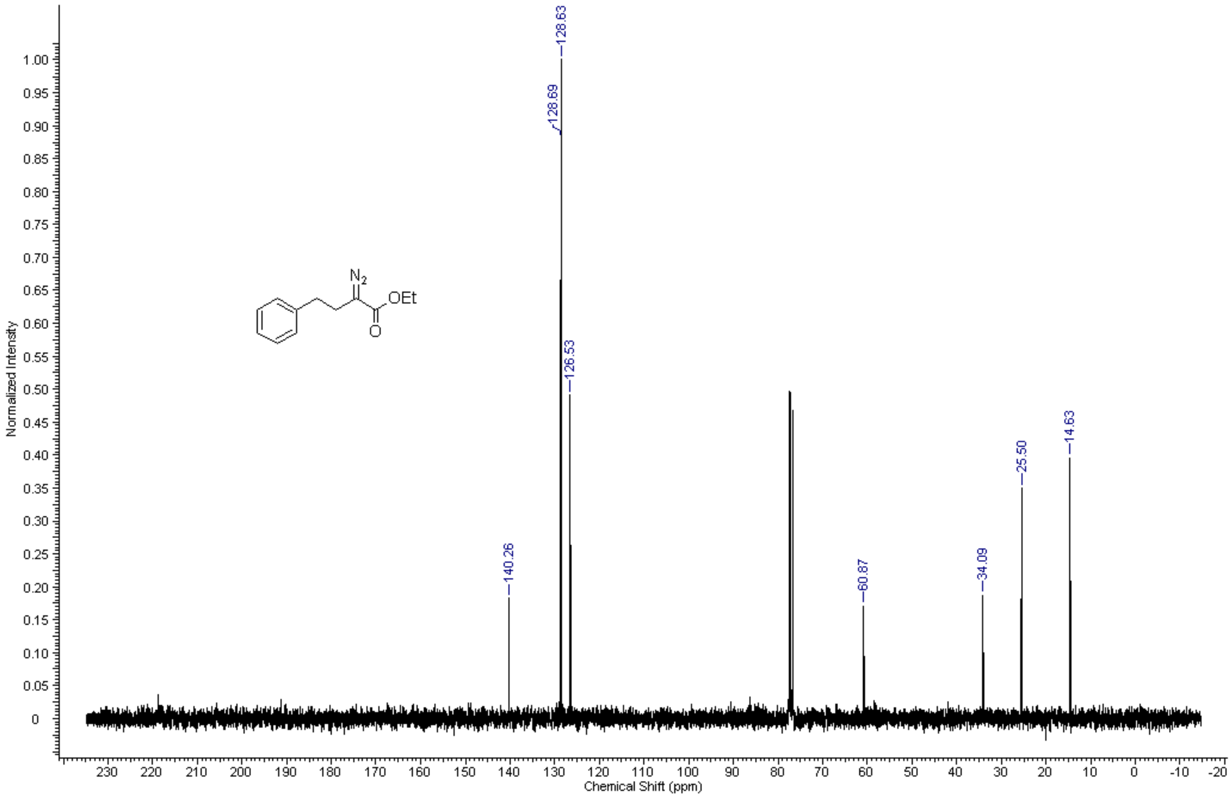

Allyl 2-diazo-2-phenylacetate 11h

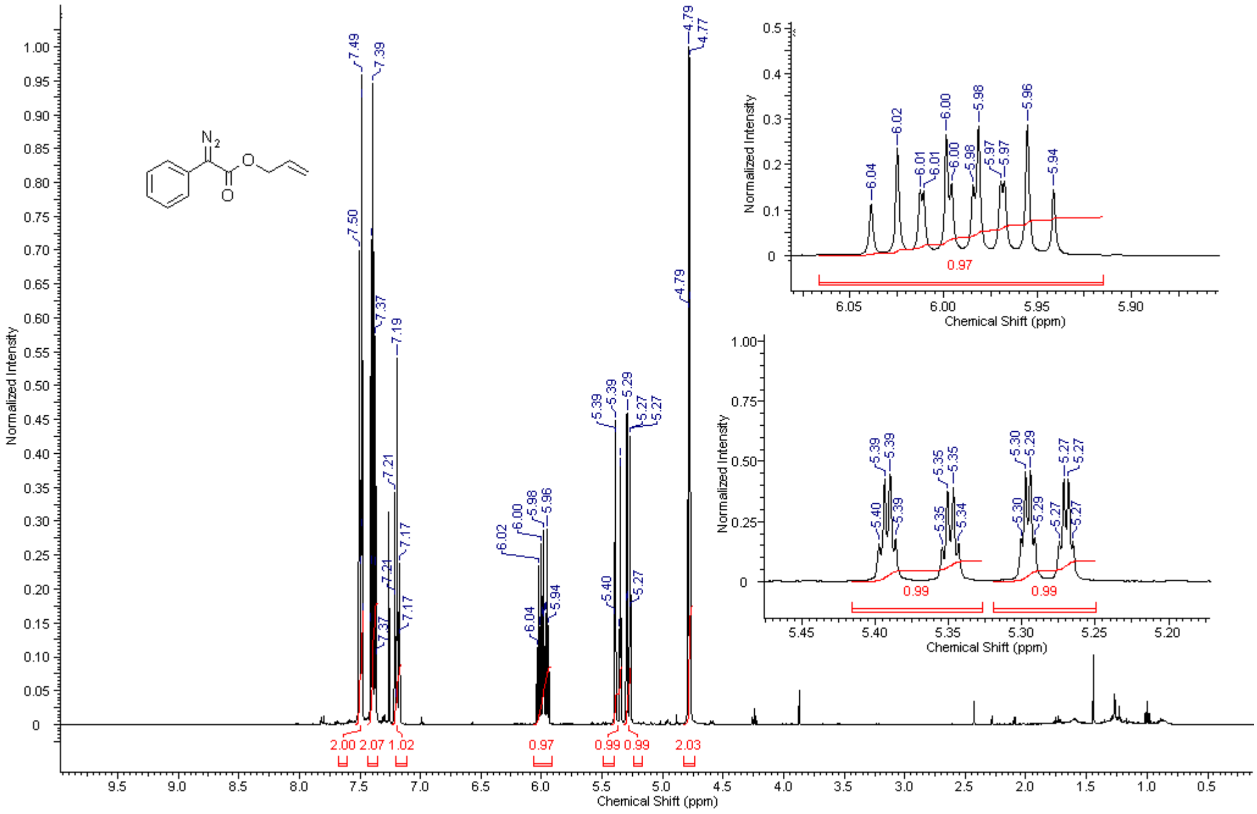

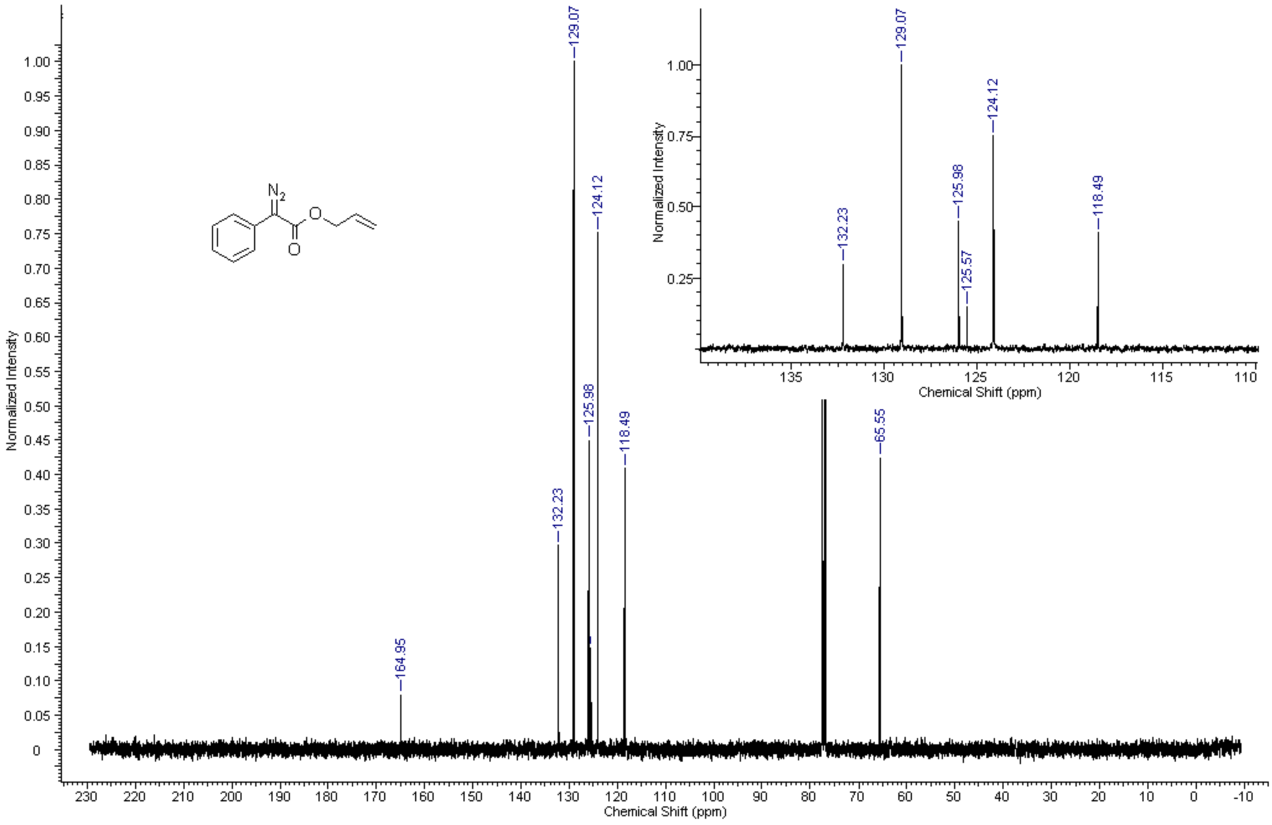

Cyclohexyl 2-diazo-2-phenylacetate 11i

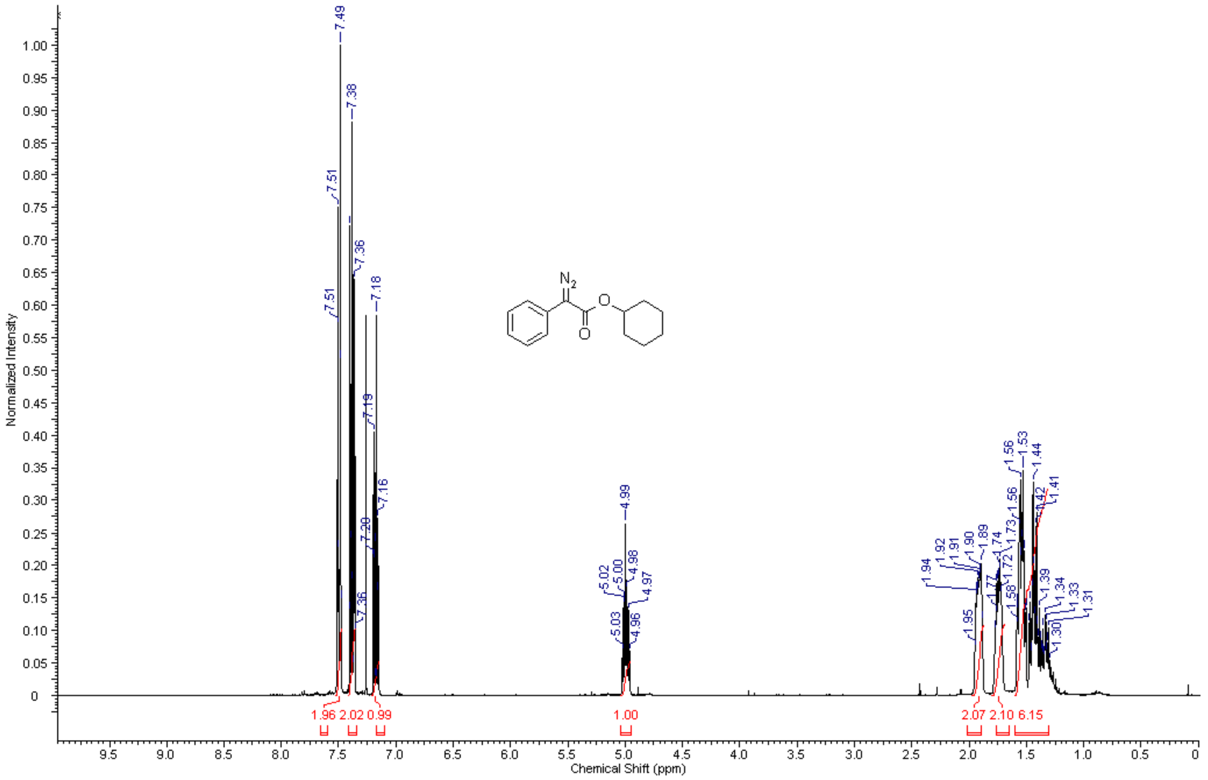

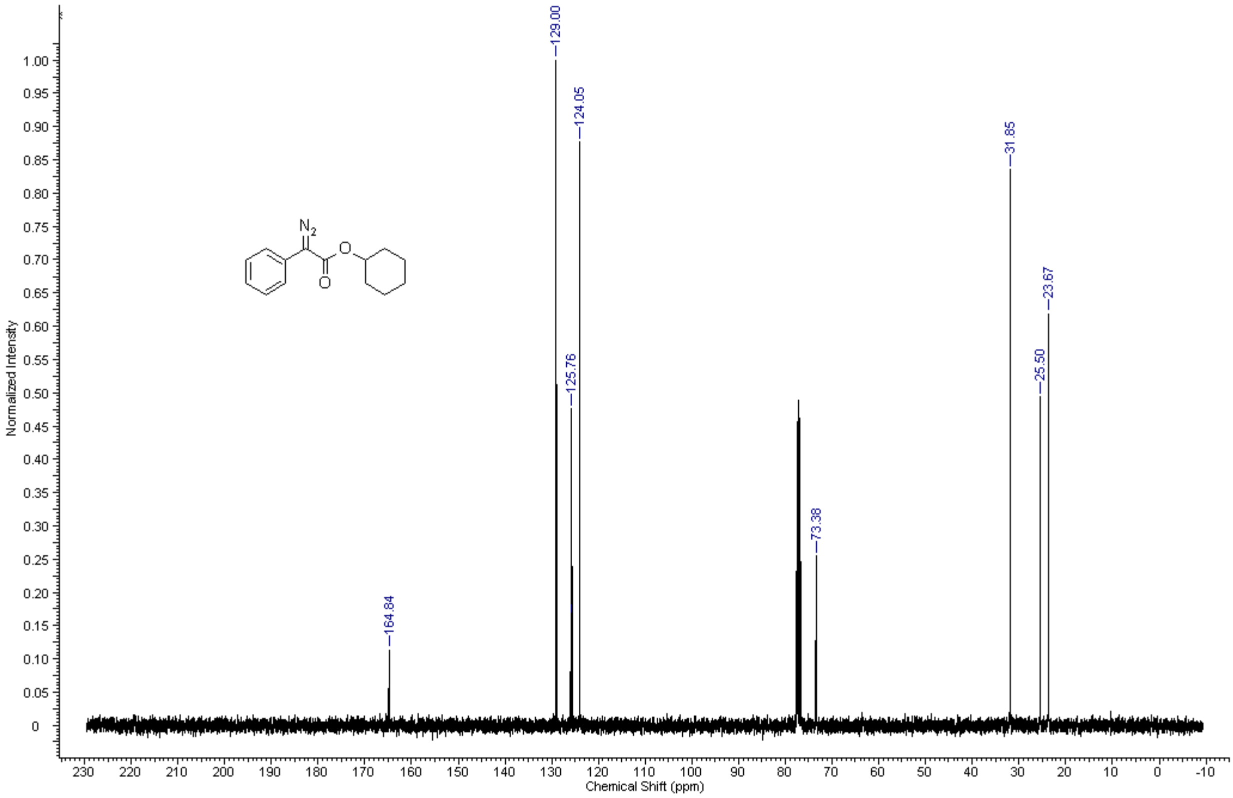

3-Diazo-4,4-dimethyldihydrofuran-2(*3H*)-one 11j

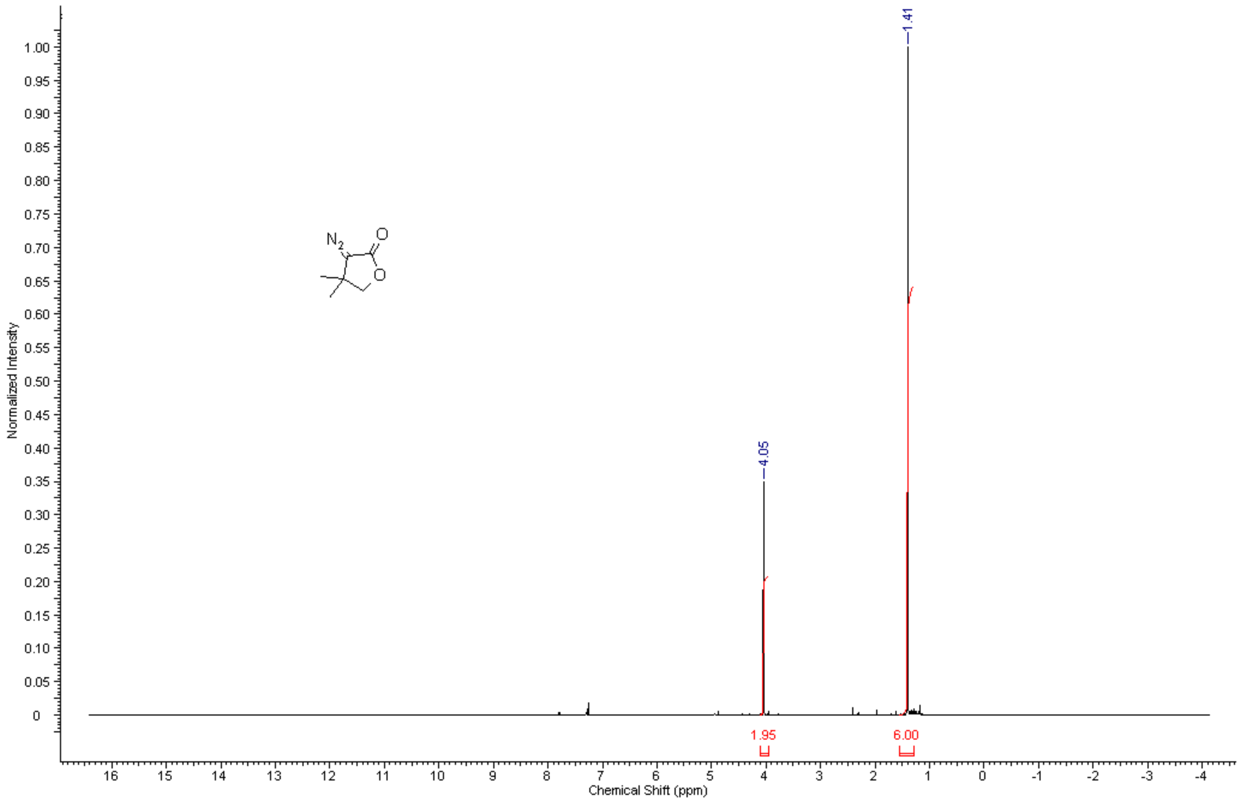

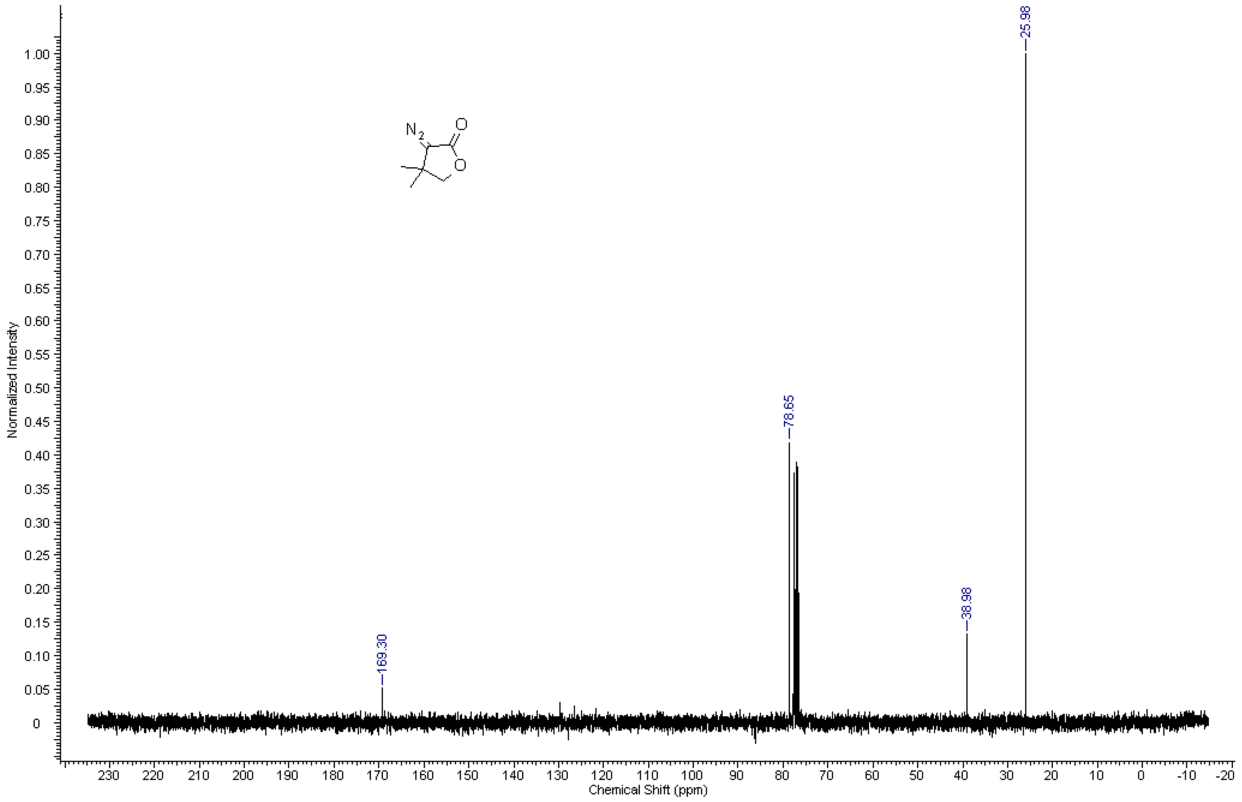

3-Diazoindolin-2-one 11k

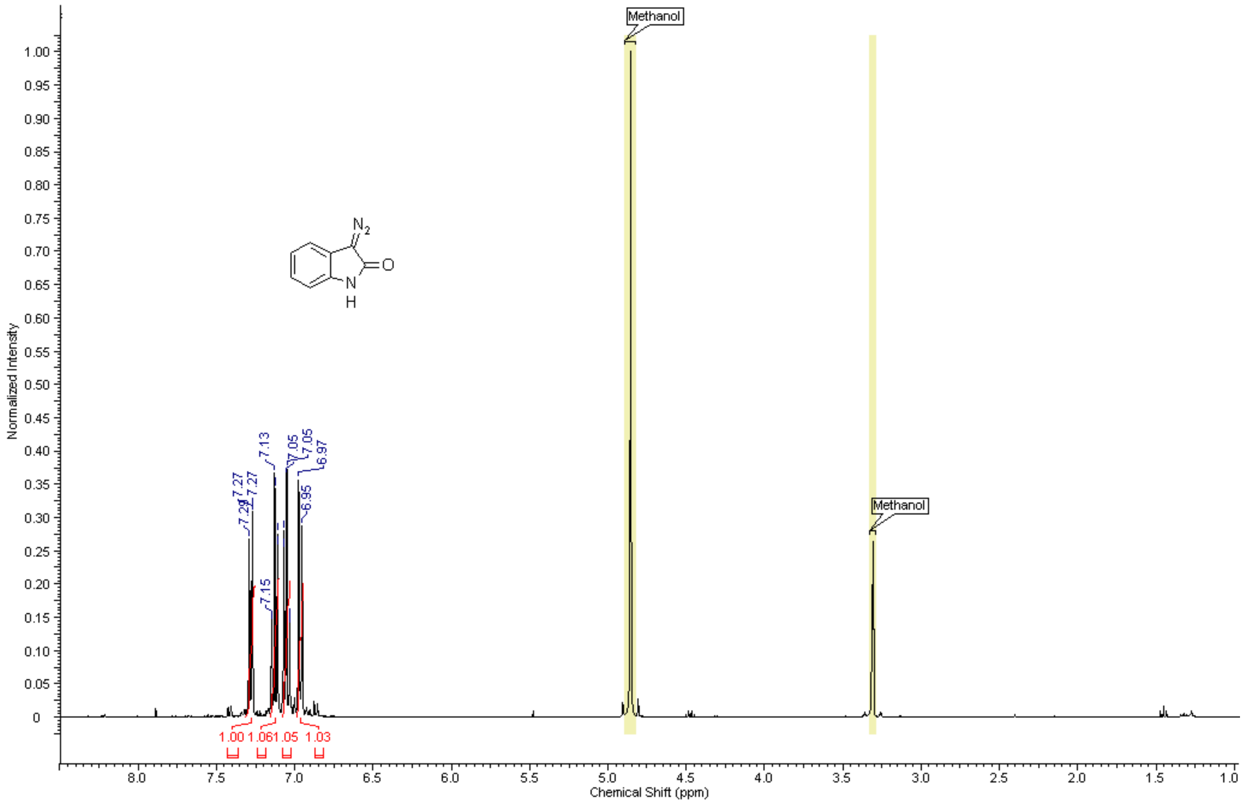

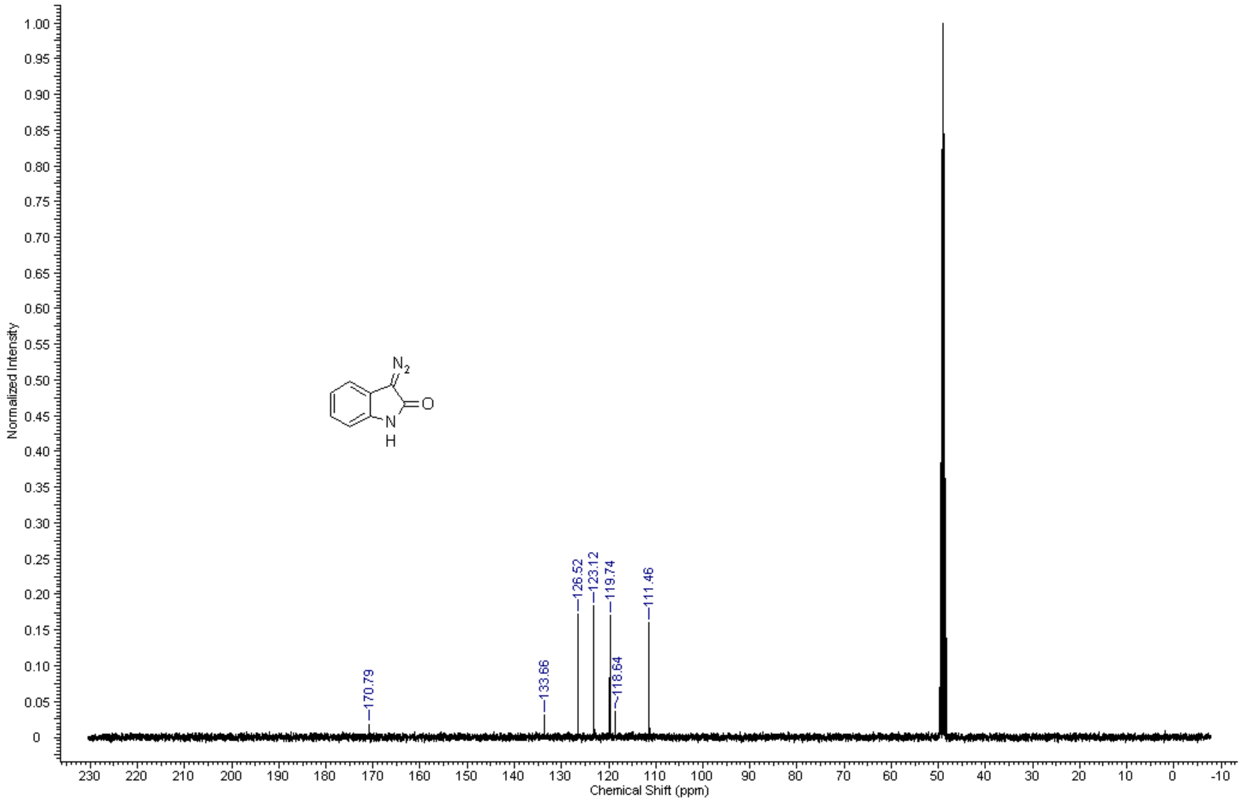

3-Diazo-1-methylindolin-2-one 11l

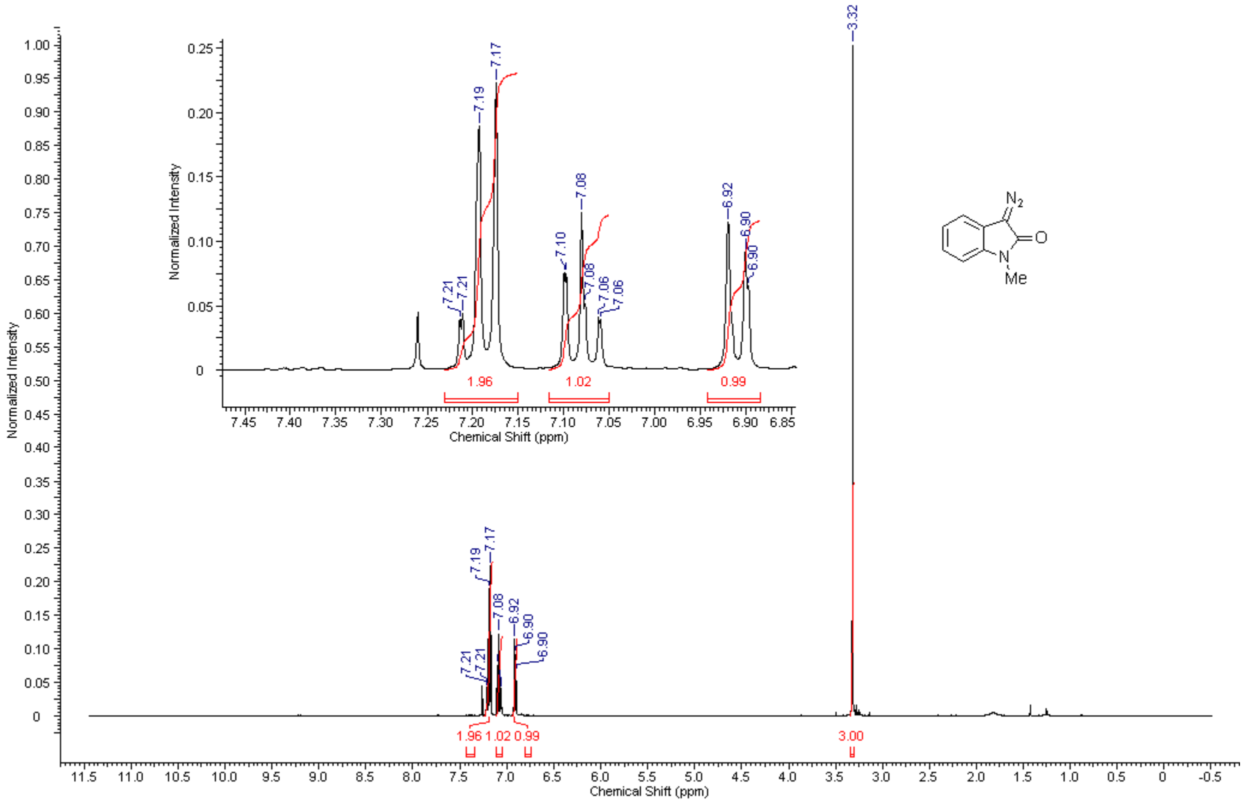

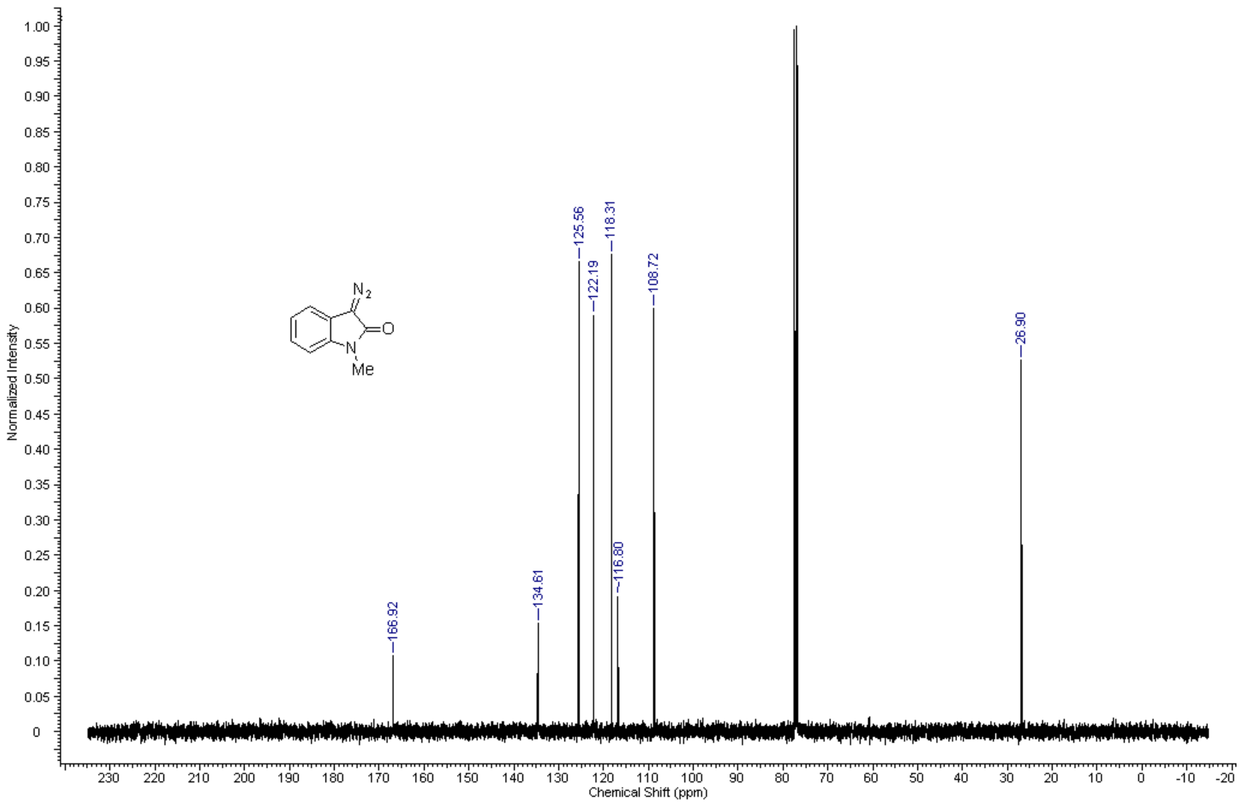

Ethyl 2-diazo-2-(2-thienyl)acetate 11m

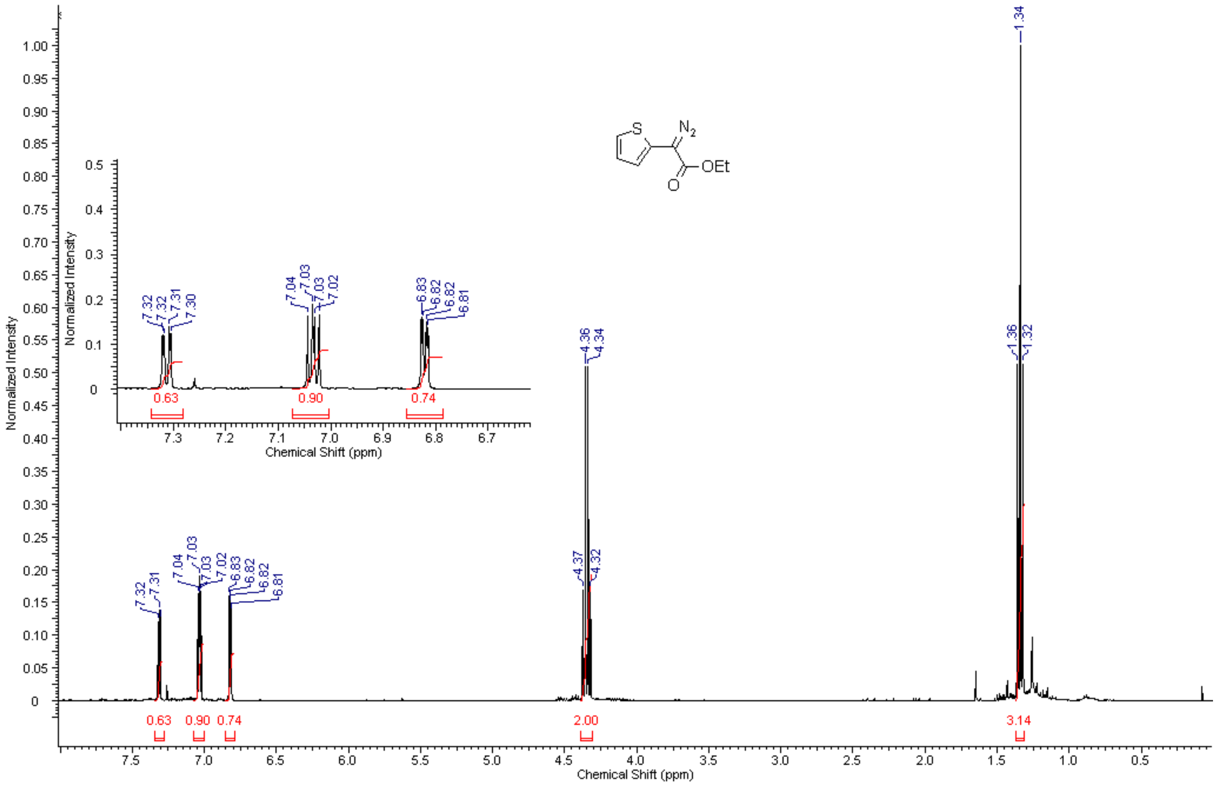

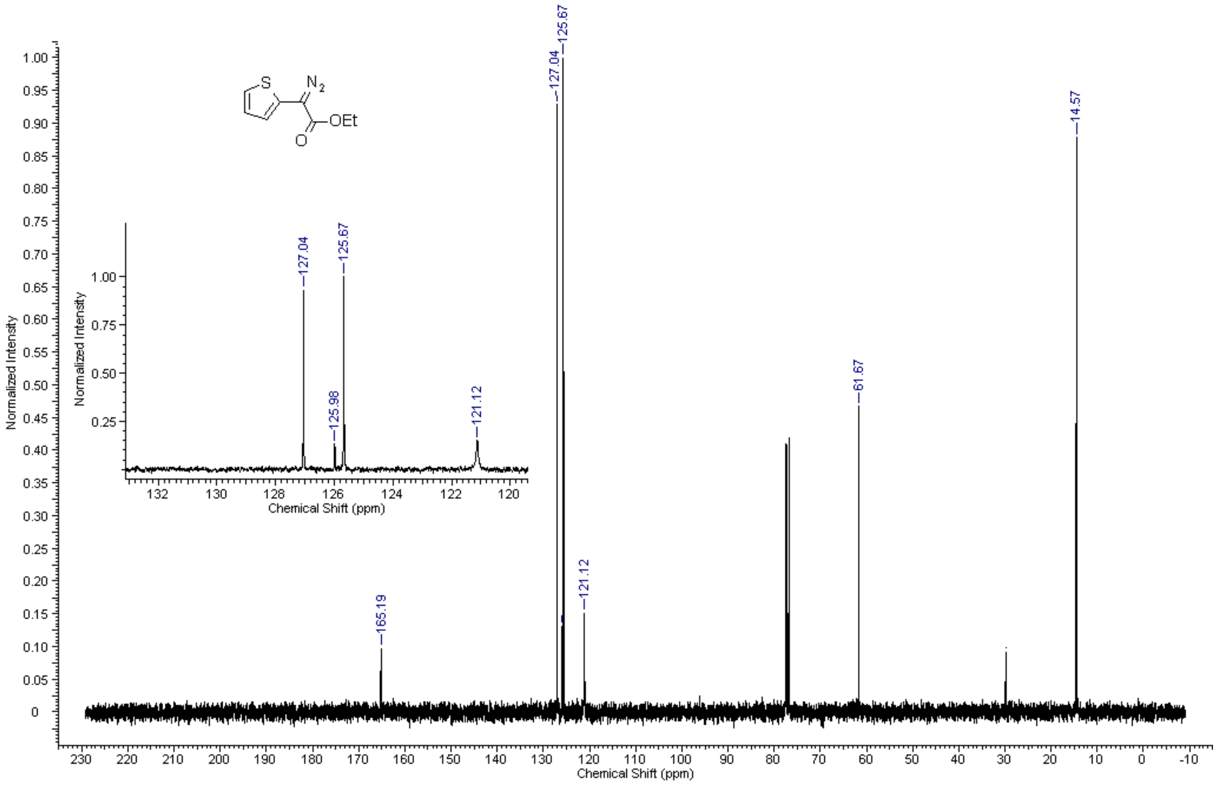

Ethyl [1,2,3]triazolo[1,5-*a*]pyridine-3-carboxylate 11n

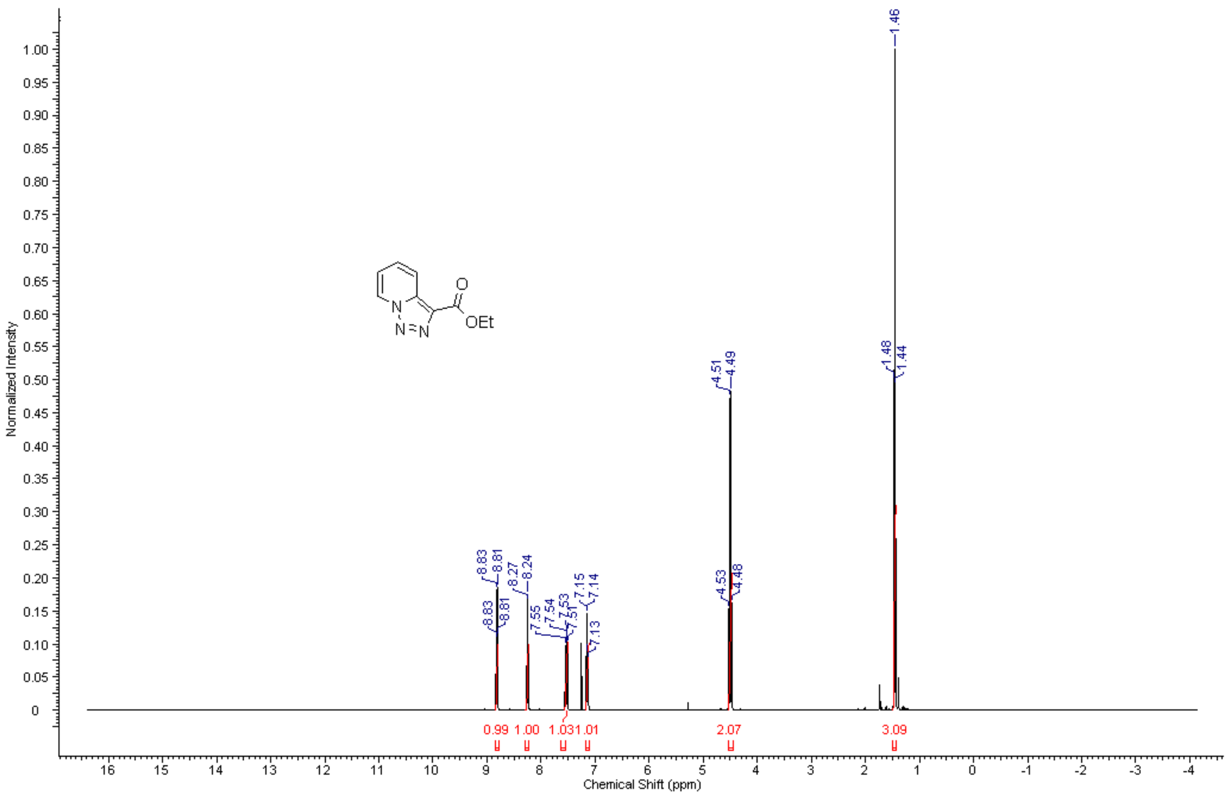

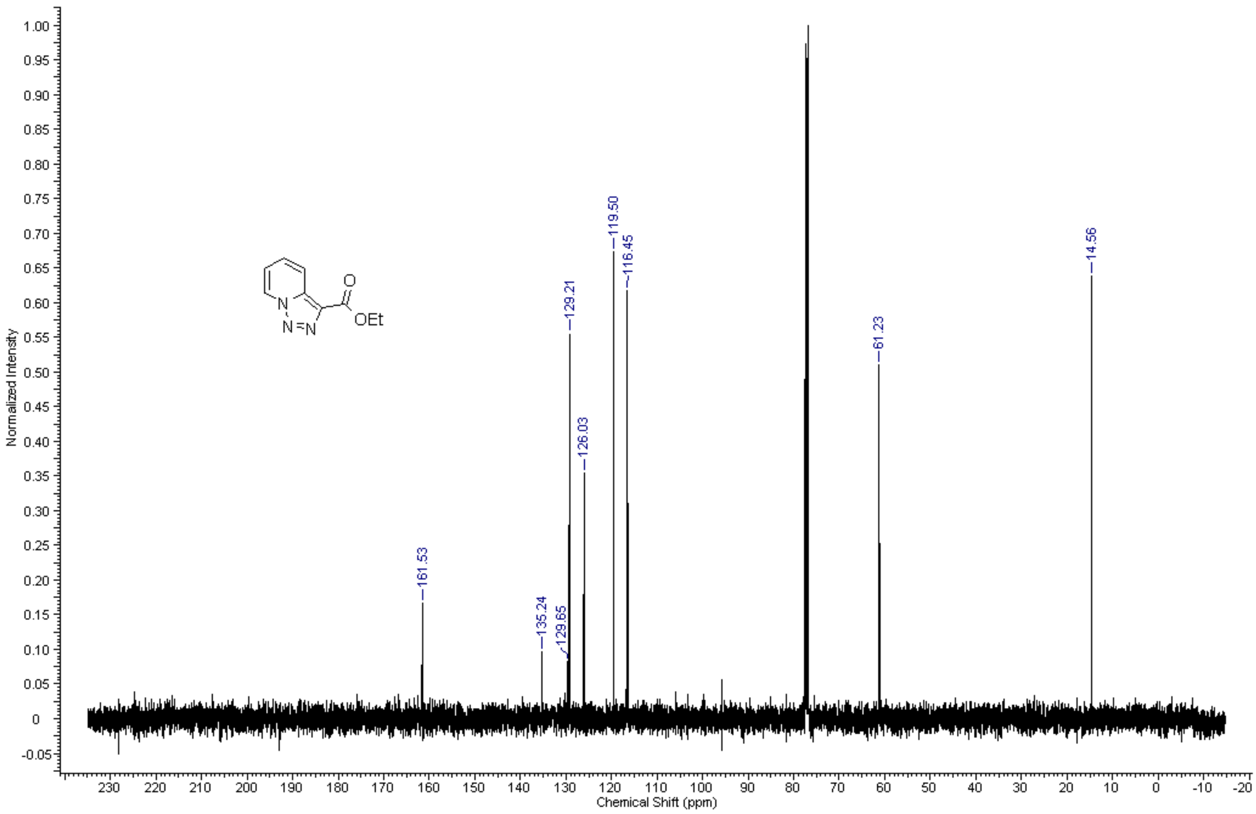

tert-Butyl 3-(1-diazo-2-methoxy-2-oxoethyl)-_1H_-indole-1-carboxylate 11o

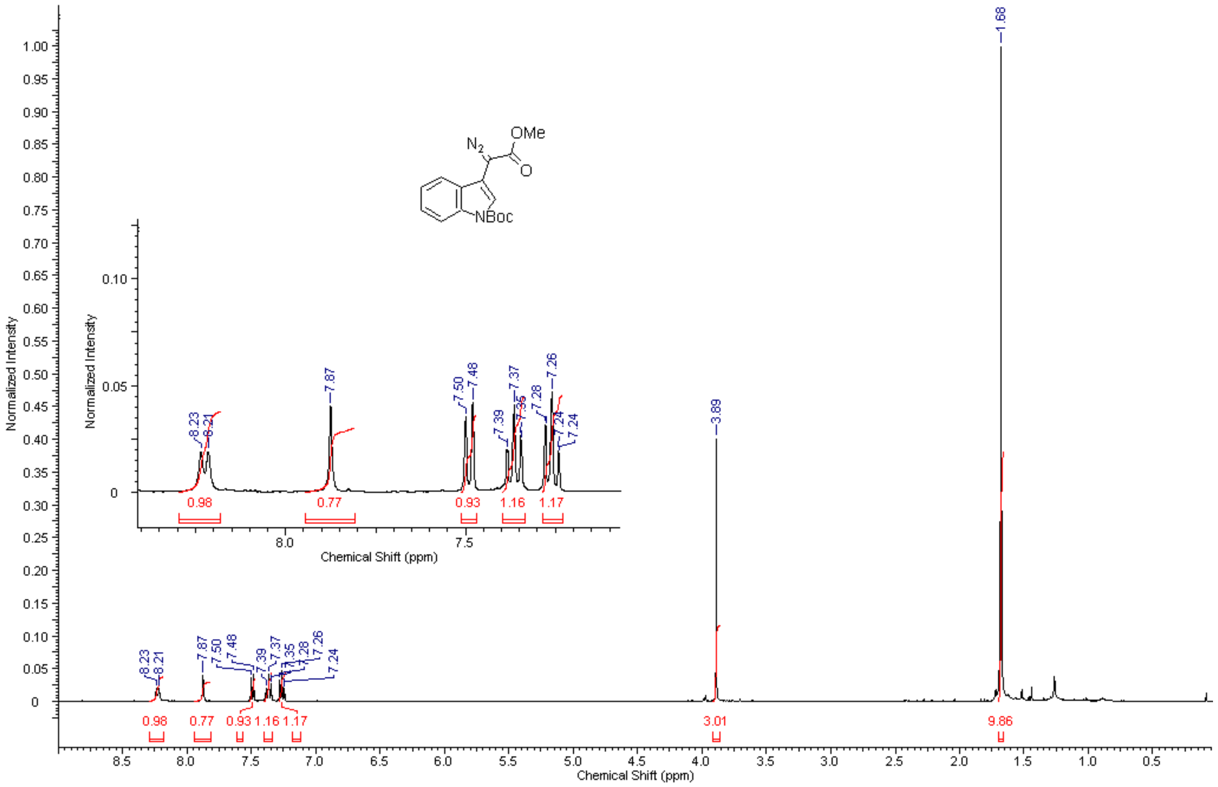

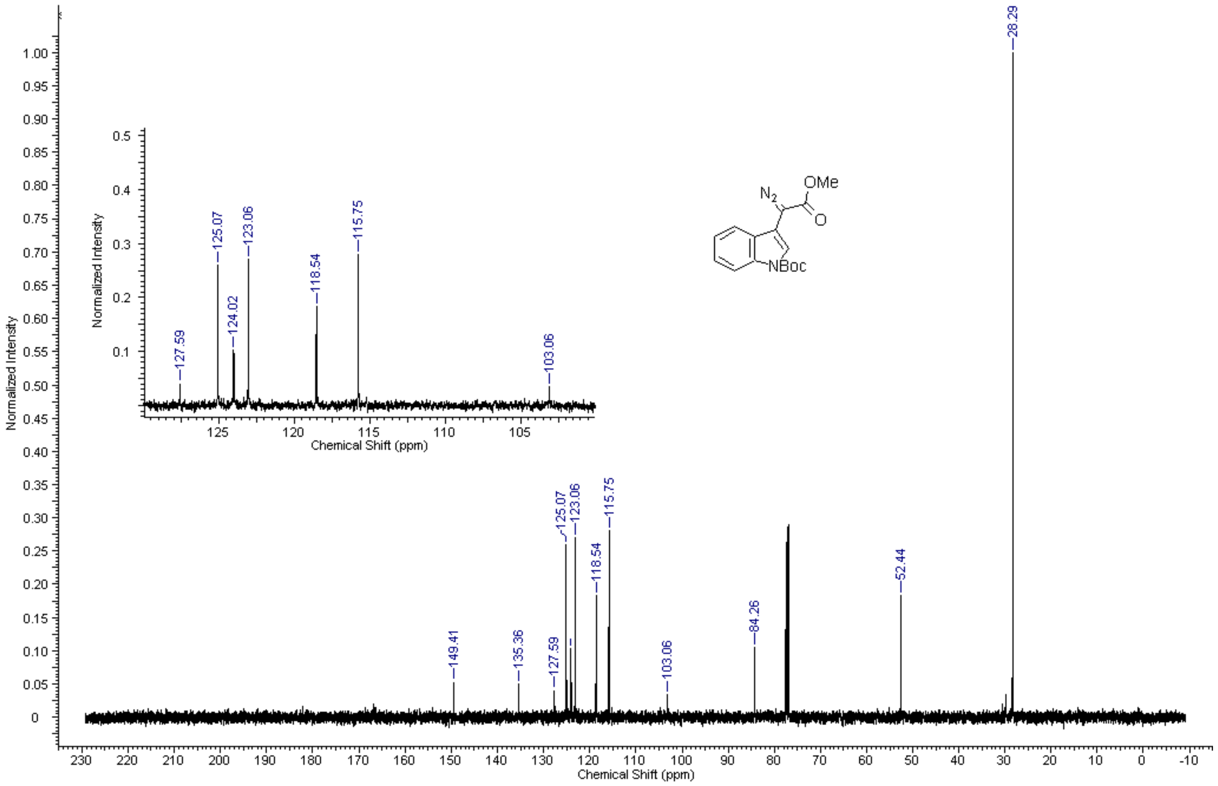

1-Allyl-4-phenyl-*1H*-1,2,3-triazol-5-ol 11p

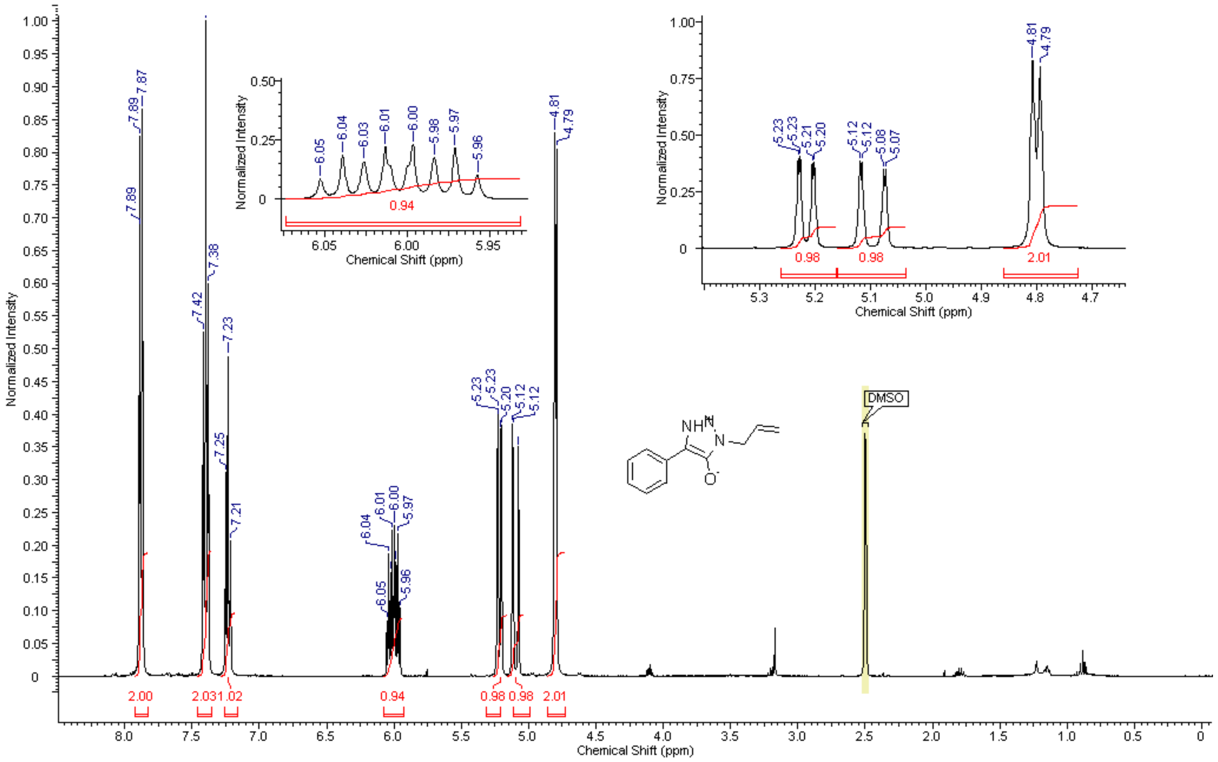

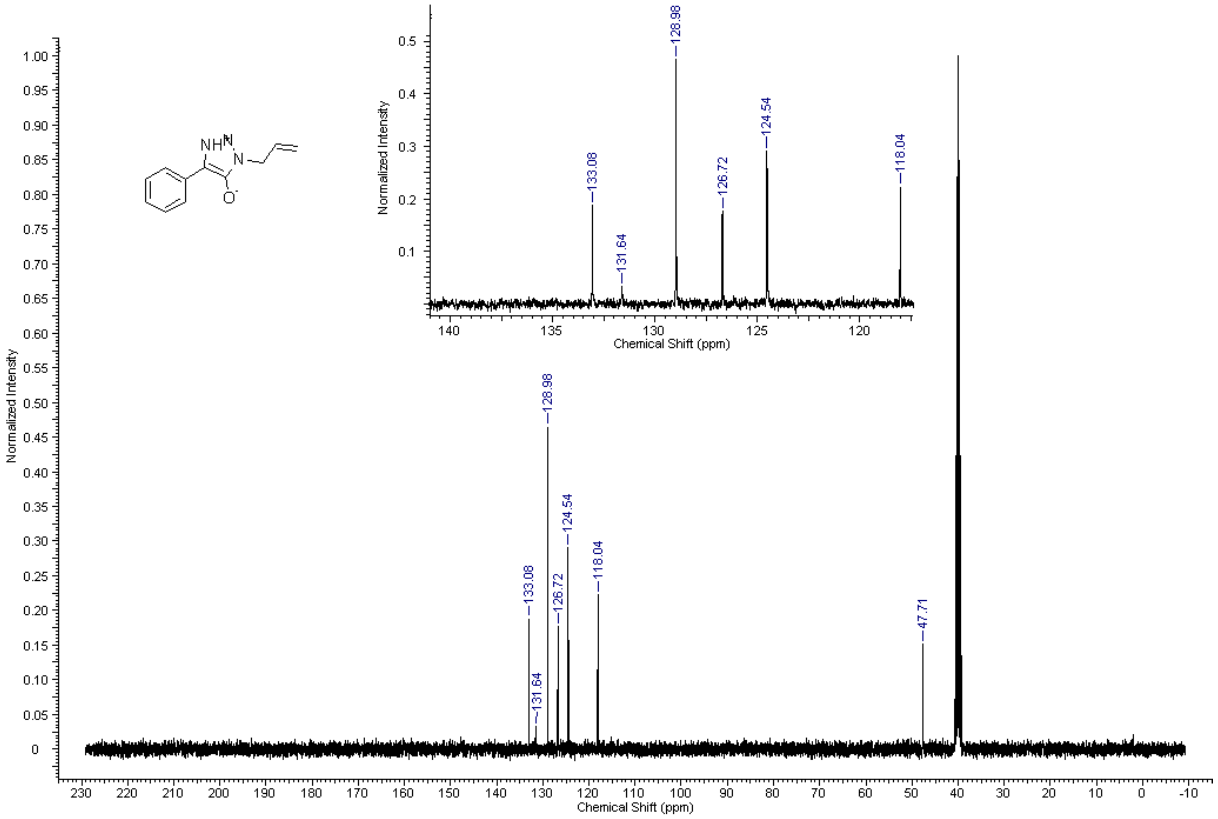

1-(2,5-Dimethoxyphenyl)-4-phenyl-1H-1,2,3-triazol-5-ol 11q

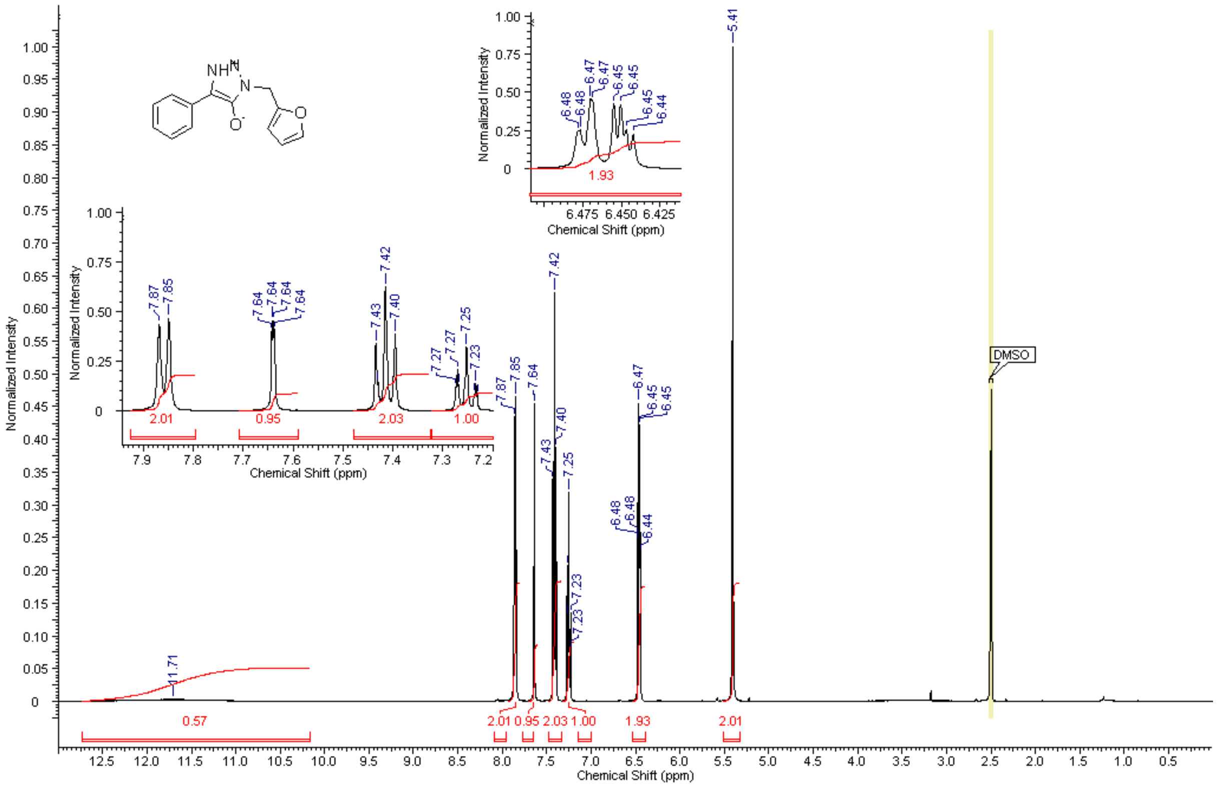

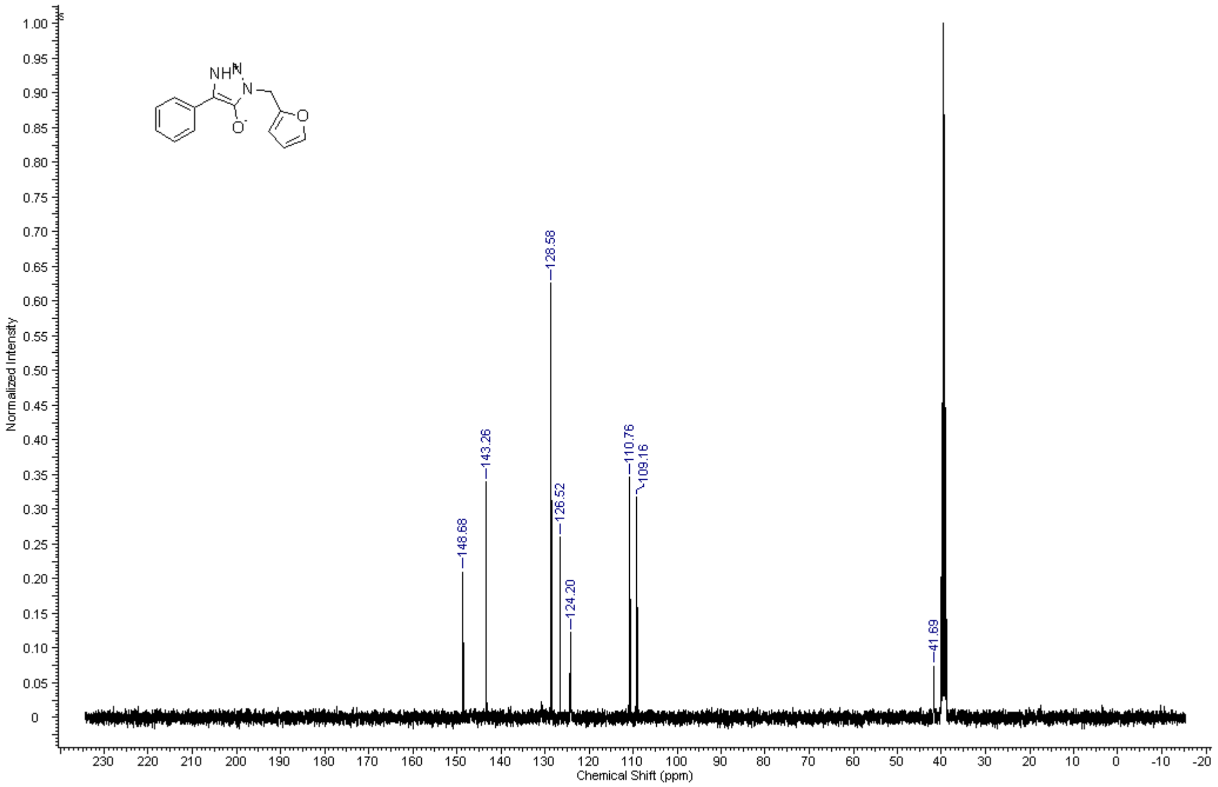

1-(2-Furylmethyl)-4-phenyl-1H-1,2,3-triazol-5-ol 11r

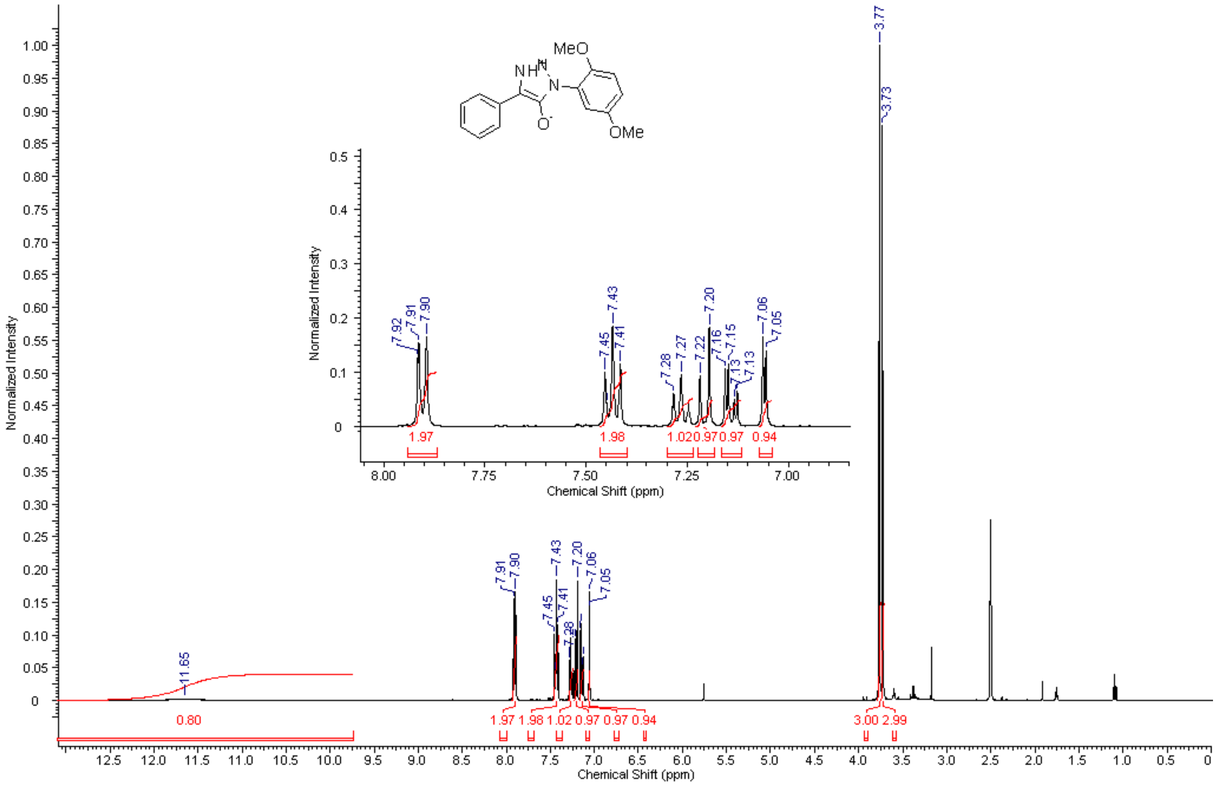

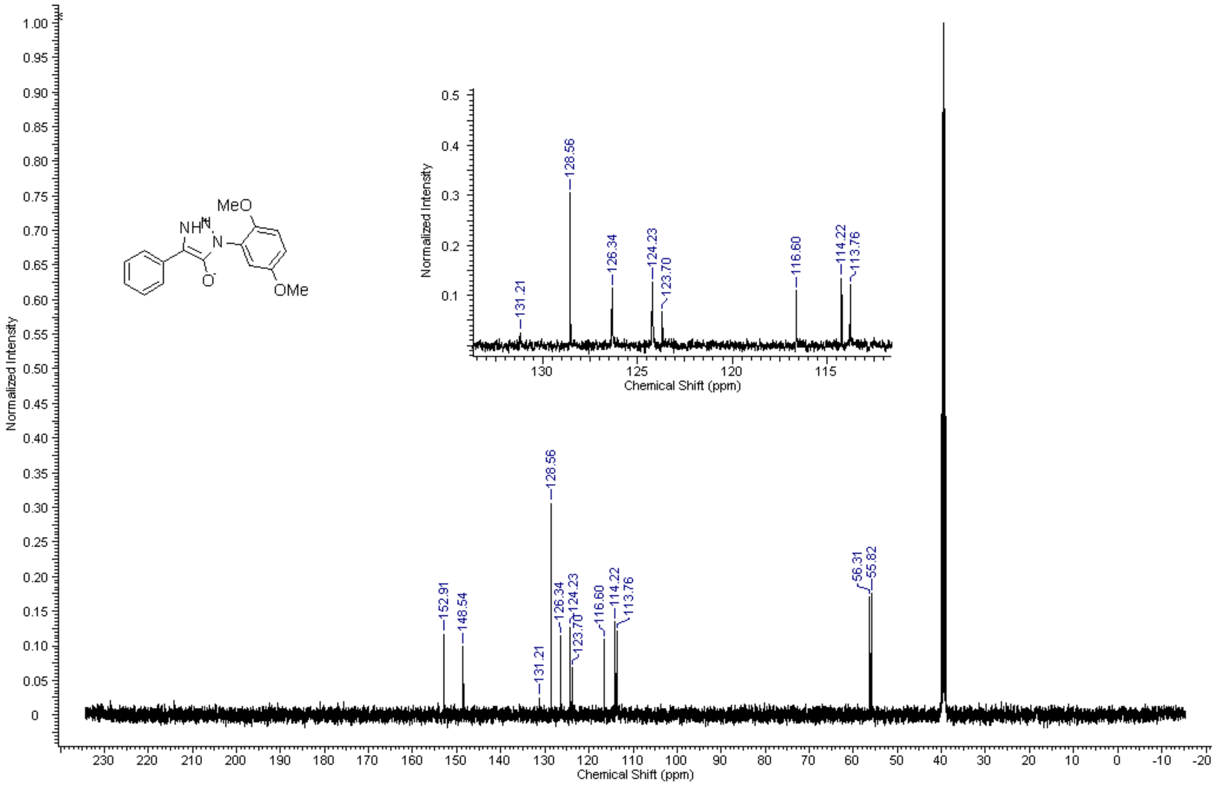

Dimethyl 1-diazoethylphosphonate 12a

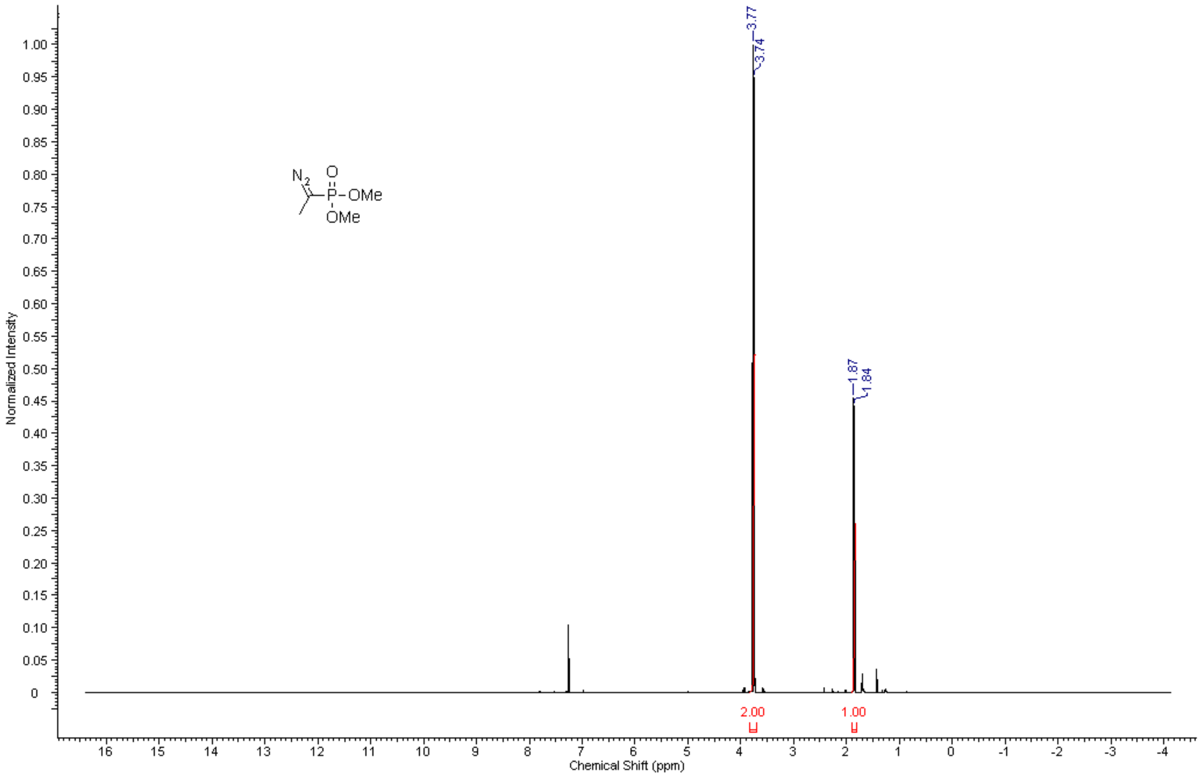

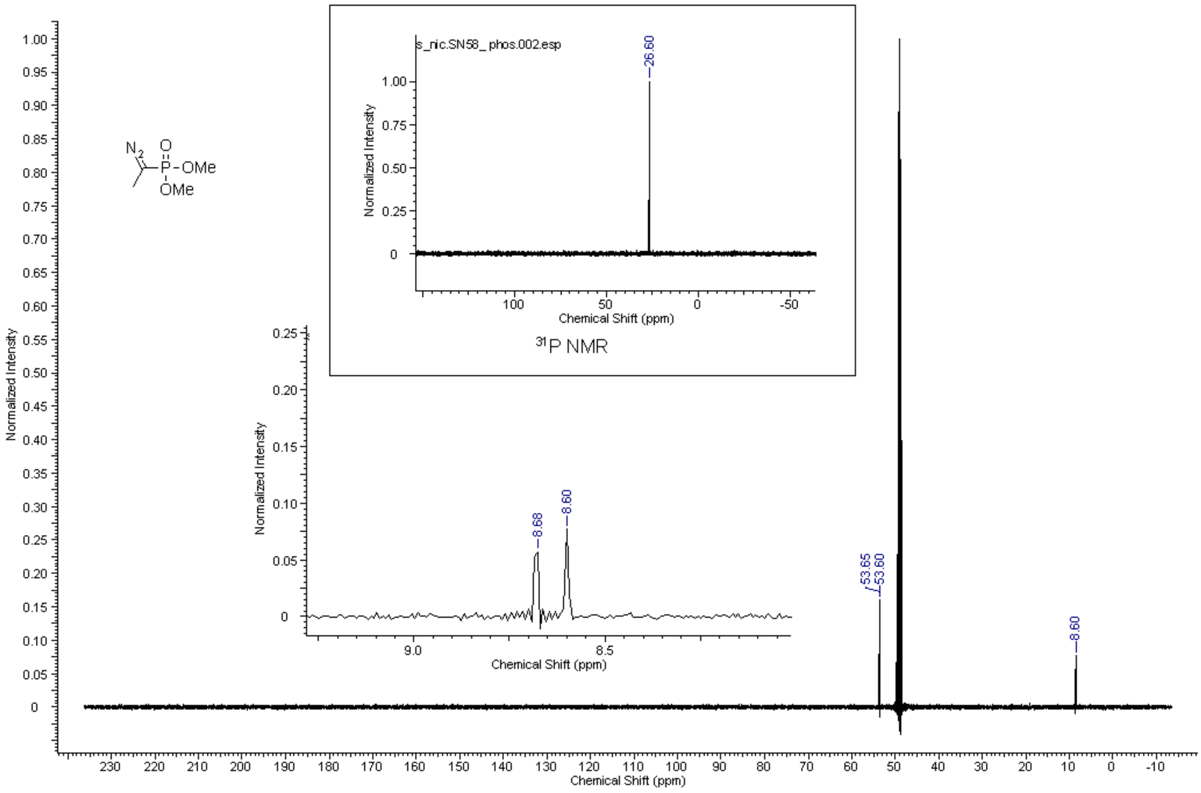

Diethyl 1-diazo-2-phenylethylphosphonate 12b

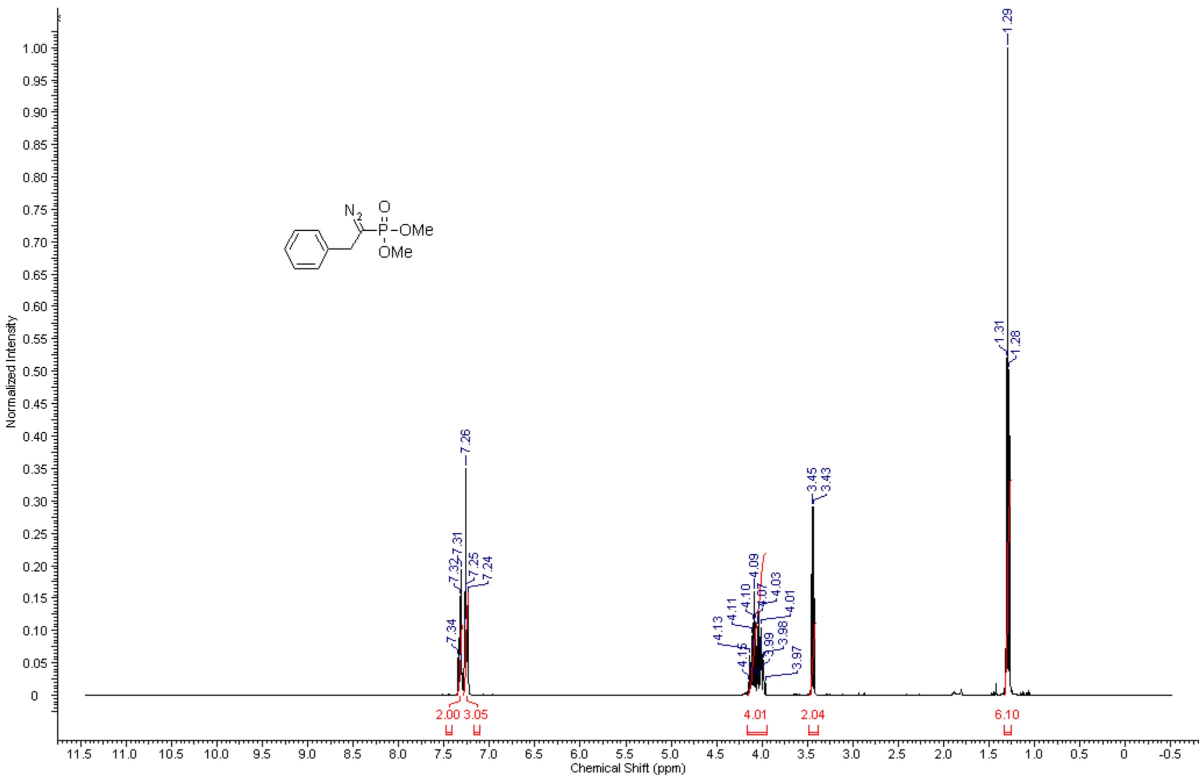

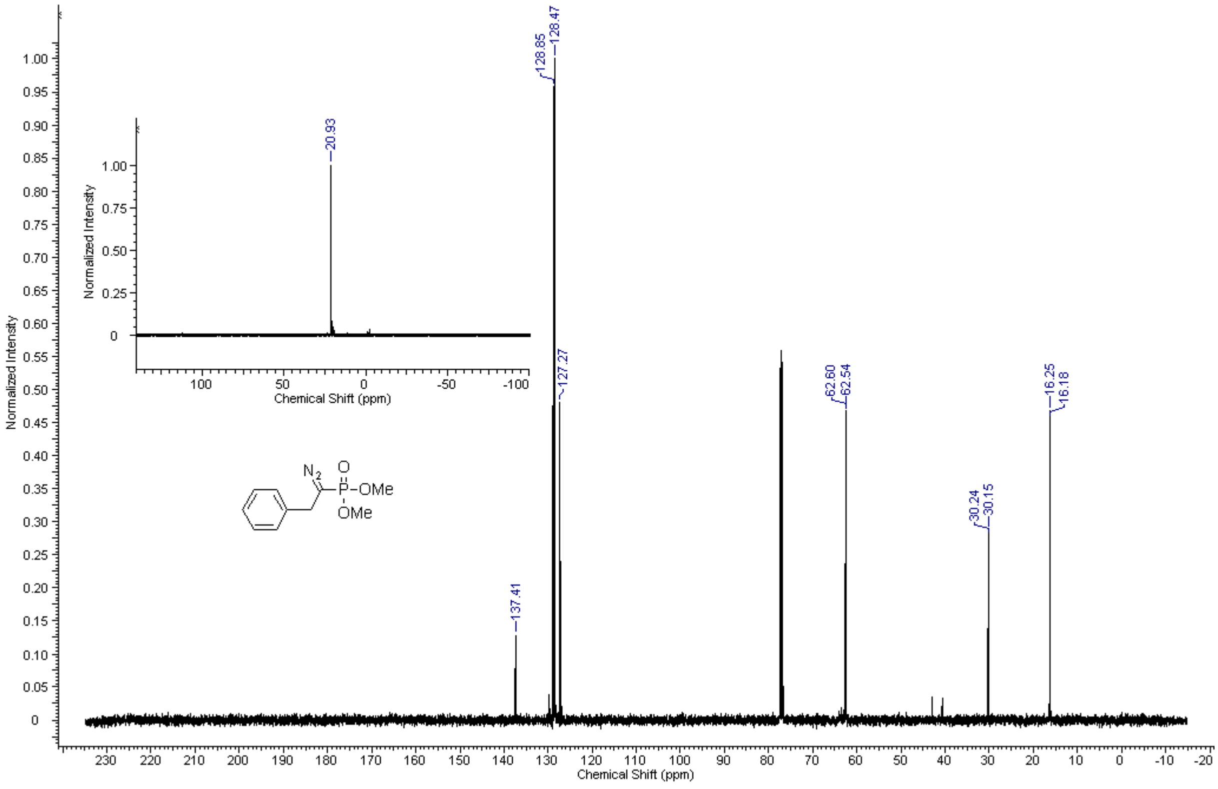

2-Diazo-1-phenylethanone 13

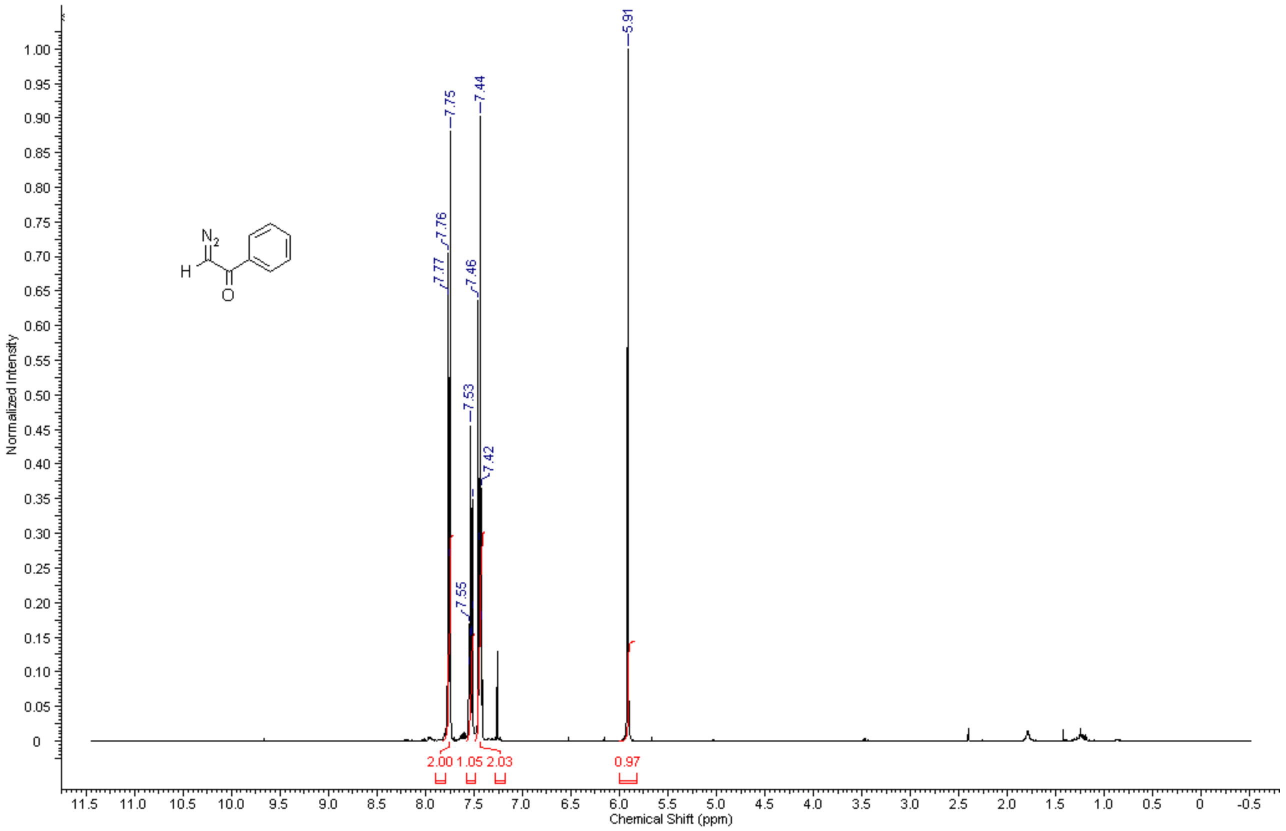

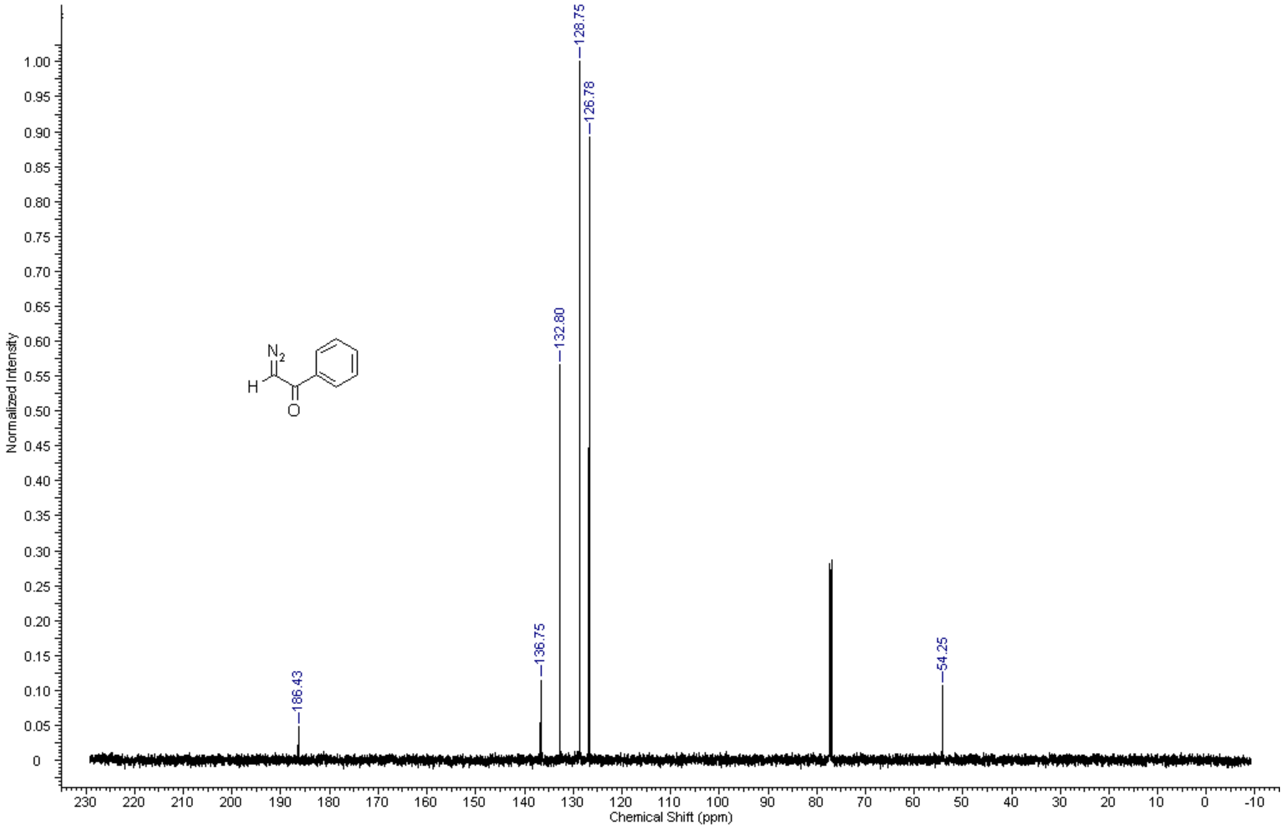

Supplement: Supplementary file 1 [file chem0020-4420-sd1.pdf]
